# The AHCY–adenosine complex rewires mRNA methylation to enhance fatty acid biosynthesis and tumorigenesis

**DOI:** 10.1038/s41422-025-01213-5

**Published:** 2026-01-19

**Authors:** Kun Liao, Fen Cao, Chen Wei, Zheng-Yu Qian, Hong-Rong Hu, Wen-Feng Pan, Zi-Qing Feng, Sen-mao Lian, Zi-Xuan Xiao, Hui Sheng, Hai-Yu Mo, Yi-Xuan Zhao, Qi-Nian Wu, Zhao-Lei Zeng, Bo Li, Rui-Hua Xu, Huai-Qiang Ju

**Affiliations:** 1 State Key Laboratory of Oncology in South China, Collaborative Innovation Center for Cancer Medicine, Sun Yat-sen University Cancer Center, Sun Yat-sen University, Guangzhou, Guangdong China; 2 Department of Urology, Guangdong Provincial People’s Hospital, Southern Medical University, Guangzhou, Guangdong China; 3 Department of Biochemistry, Zhongshan School of Medicine, Sun Yat-sen University, Guangzhou, Guangdong China; 4 Department of Biochemistry and Molecular Biology, Cancer Research Institute, School of Basic Medical Sciences, Southern Medical University, Guangzhou, Guangdong China; 5 Research Unit of Precision Diagnosis and Treatment for Gastrointestinal Cancer, Chinese Academy of Medical Sciences, Guangzhou, Guangdong China; 6 Experimental Education and Administration Center, School of Basic Medical Sciences, Southern Medical University, Guangzhou, Guangdong China; 7 Department of Medical Oncology, Sun Yat-sen University Cancer Center, State Key Laboratory of Oncology in South China, Guangdong Provincial Clinical Research Center for Cancer, Sun Yat-sen University, Guangzhou, Guangdong China

**Keywords:** Cancer metabolism, RNA modification

## Abstract

Methionine metabolism generates the substrate S-adenosylmethionine (SAM), which regulates epigenetic modifications crucial for various cellular processes, particularly tumorigenesis. However, whether methionine metabolism involves epigenetic mechanisms independent of SAM and what roles such mechanisms play in tumorigenesis remain unclear. We show here that the adenosylhomocysteinase (AHCY)–adenosine complex increases mRNA m^6^A levels in a non-global manner, promoting fatty acid synthesis and tumorigenesis. Adenosine increases mRNA m^6^A levels by binding to the methionine metabolism enzyme AHCY to form a complex, rather than depending on adenosine receptors. The AHCY–adenosine complex facilitates AHCY dimerization, with adenosine being crucial for dimer stability. AHCY dimers hinder the binding of fat mass and obesity-associated protein (FTO) at the Q86 site to RNA containing the VWDRACH motif, increasing m^6^A levels and upregulating lipogenesis genes, especially ACACA and SCD1, thus leading to reprogramming of lipid metabolism. Conversely, AHCY mutants that have lost dimerization or FTO-binding ability but retain hydrolase activity suppress lipogenesis and tumor growth without significantly affecting methionine catabolism mediated by AHCY. Loss of AHCY in mice and disruption of AHCY dimerization in tumor cells and patient-derived xenograft models restricted tumor growth. Our findings demonstrate a key SAM-independent link between methionine metabolism and mRNA m^6^A modification that affects demethylase substrate specificity. This novel link between the methionine cycle and lipid metabolism suggests new strategies for anticancer therapy.

## Introduction

Metabolism is an essential component of biology, enabling rapid and extensive reprogramming through the transcription and translation processes of RNA.^1–3^ Metabolism also controls the activity of enzymes responsible for reversible RNA modifications such as N6-methyladenosine (m^6^A).^3–5^ m^6^A is the most prevalent internal modification of messenger RNA (mRNA) in higher eukaryotes. m^6^A has widespread effects, having been shown to participate in the control of RNA stability, splicing, and translation, and the dysregulation of m^6^A alterations is closely tied to various developmental diseases, as well as cancer development.^6–10^ This chemical modification is catalyzed by the METTL3–METTL14 complex, an S-adenosylmethionine (SAM)-dependent RNA methyltransferase (MTase).^9,10^ The discovery of fat mass and obesity-associated protein (FTO) as the first RNA demethylase highlights the reversible and dynamic nature of m^6^A modification, which is similar to that of extensively studied reversible DNA and histone modifications.^11^ Metabolism has an important role in influencing epigenetics and cell fate decisions.^3,5,12–15^ However, certain aspects of metabolic enzymes and metabolites, as well as their regulatory mechanisms in RNA epigenetics, remain unclear.

Methionine metabolism, together with the availability of related environmental nutrients, plays a crucial role in RNA epigenetics, thereby regulating a variety of cellular processes.^5,16,17^ Methionine is converted into SAM in an MTase-catalyzed reaction that yields methylated substrates with roles in histone, DNA, and RNA methylation.^5,18,19^ We previously found that SAM, a product of methionine metabolism, facilitates m^6^A methylation in tumor cells.^13^ Although SAM supplementation can rescue histone methylation caused by methionine deficiency, it is unable to fully restore the downregulation of mRNA m^6^A (Supplementary information, Fig. S1a–c). Methionine metabolism and its key metabolic genes have been implicated in cancer development and metastasis through epigenetic changes related to methylation,^5,13,16^ highlighting the potential of targeting methionine metabolism for cancer therapy. However, it remains to be determined whether SAM-independent mechanisms play a role in the regulation of mRNA m^6^A modification by methionine metabolic genes and thus affect tumor biology.

In addition to resulting from concomitant aberrations in signaling pathways, alterations in metabolites can also be key factors influencing physiological and pathological processes.^1,12,20^ Adenosine (ADO) is a metabolite of the methionine cycle and an endogenous nucleoside that functions as both an intermediate metabolite and an extracellular signaling molecule.^21^ It is primarily produced intracellularly through the degradation of AMP or the reversible hydrolysis of S-adenosylhomocysteine (SAH) catalyzed by the methionine metabolism enzyme adenosylhomocysteinase (AHCY). ADO is then transported extracellularly by bidirectional nucleoside transporters or released through nucleotide metabolic pathways into the extracellular environment. ADO signaling controls several physiological and pathological processes,^21–23^ such as sleep–wake regulation, vasodilation, epilepsy, chronic inflammation, fibrosis, diabetes, and cancer development, via the ADO receptors. However, the importance of intracellular ADO metabolism and receptor-independent ADO signaling mechanisms remains unclear.^21^

Here, we demonstrate that the methionine metabolism enzyme AHCY specifically regulates mRNA methylation through a SAM-independent pathway by complexing with ADO. Mechanistically, the AHCY–ADO complex significantly enhances the direct physical interaction of AHCY with FTO by increasing AHCY dimerization. This interaction inhibits the binding of FTO to specific RNA molecules containing m^6^A motifs, which preferentially bind to the FTO Q86 site, thus increasing m^6^A levels and upregulating lipogenesis genes such as *ACACA* and *SCD1*, ultimately promoting fatty acid biosynthesis, tumor cell proliferation and tumorigenesis.

## Results

### AHCY increases mRNA m^6^A methylation

**Fig. 1:**
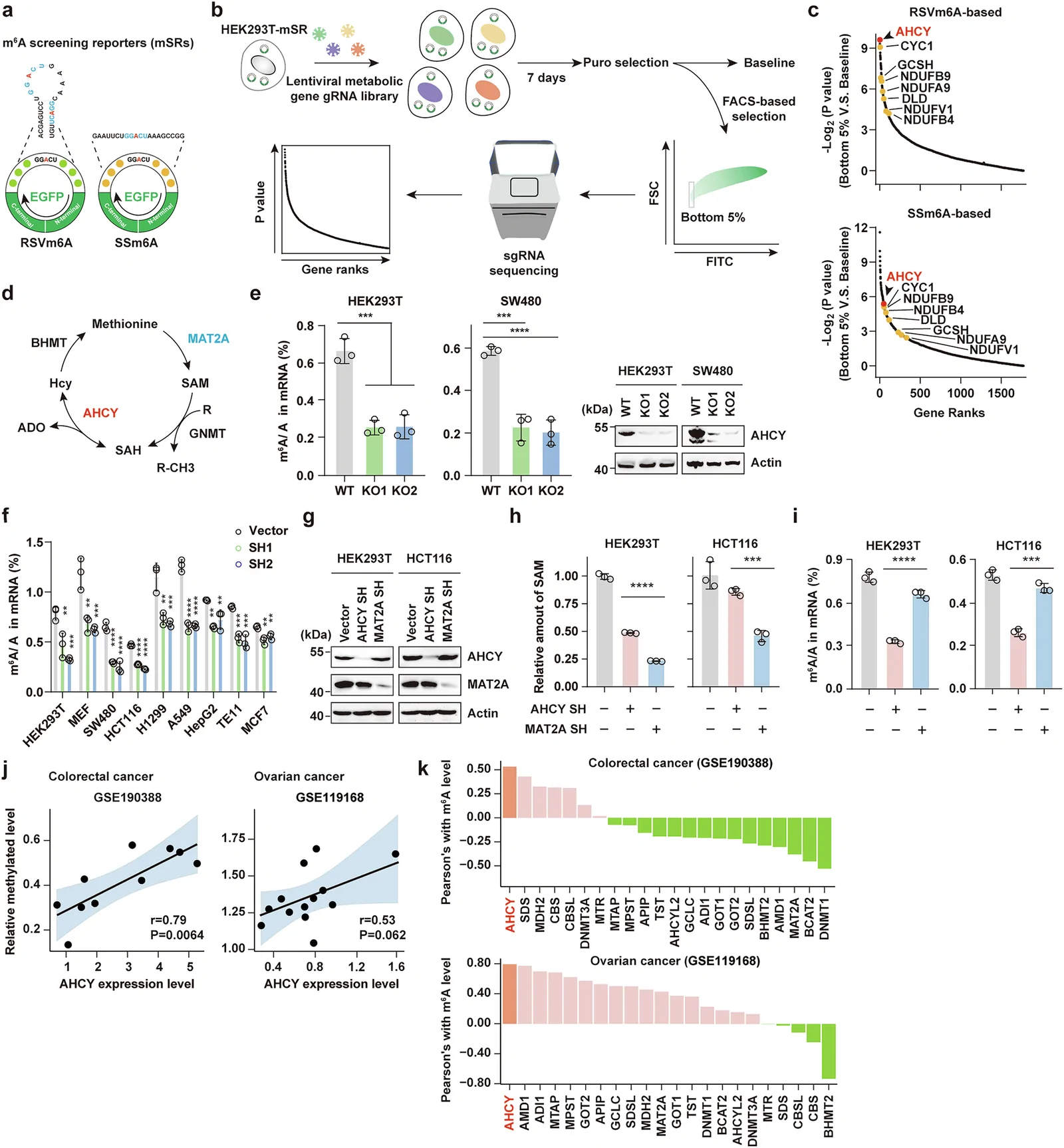
AHCY specifically increases mRNA m^6^A methylation. **a** Schematic diagram of two circular RNA (circRNA) translational reporters containing a GGACU motif that can be backspliced to generate circRNAs that drive GFP translation. One reporter had a linear structure (SSm6A), and the other had a hairpin structure (RSVm6A). **b** Overview of the CRISPR screen performed under conditions in which the medium was supplemented with 50 μM S-adenosylmethionine (SAM). **c** Positive regulators of m^6^A identified in the screen using the SSm6A and RSVm6A reporters. The displayed genes are associated with significantly enriched pathways, and AHCY is highlighted in red. **d** Schematic diagram of the methionine cycle. **e** Liquid chromatography-mass spectrometry (LC-MS/MS) quantification of the mRNA m^6^A/A ratio in WT and AHCY KO clones of HEK293T and SW480 cells (left). Immunoblot analysis of AHCY and β-actin (Actin) in HEK293T and SW480 cells upon KO of AHCY (right). **f** LC-MS/MS quantification of the mRNA m^6^A/A ratio in the indicated cells expressing vector or AHCY shRNAs. **g** Immunoblot analysis of AHCY, MAT2A, and Actin in HEK293T and HCT116 cells with AHCY or MAT2A knockdown. **h**–**k** SAM content (**h**) and LC-MS/MS quantification of the mRNA m^6^A/A ratio (**i**) in the indicated cells expressing vector, AHCY shRNAs, or MAT2A shRNAs. Scatterplots showing positive correlations between RNA methylation and AHCY mRNA expression (**j**) and between RNA methylation and the expression of genes related to one-carbon metabolism (**k**) in tissues from colorectal cancer (GSE190388) and ovarian cancer patients (GSE119168). Pearson’s correlation test. Data are presented as mean ± S.D. (*n* = 3, unless otherwise specified). Two-tailed unpaired Student’s *t*-test (**h**, **i**). One-way ANOVA with least significant difference *t*-test (LSD-*t*) (**e**, **f**). **P < 0.01, ***P < 0.001, ****P < 0.0001.

To thoroughly evaluate methionine metabolism and its associated metabolic pathways, we used an established metabolic library to perform CRISPR screening,^24^ focusing on the effects of m^6^A modification. Given the complexity of RNA structures, we selected two fluorescent reporter plasmids containing a GFP circular RNA with a GGACU consensus sequence: one with the m^6^A motif in a linear context (SSm6A)^25,26^ and the other with the motif in a hairpin structure (RSVm6A).^25,26^ The GFP pre-mRNA transcript in both reporter systems is assembled by backsplicing, generating a circular RNA that connects two GFP exon fragments (Fig. 1a). m^6^A methylation of the GGACU motif in the circular RNA can drive translation initiation of the GFP transcript, resulting in a GFP fluorescence signal. The RNA m^6^A modification is dynamically and reversibly regulated by the MTases METTL3 and METTL14 and the demethylases FTO and ALKBH5 (Supplementary information, Fig. S1d–i). Consequently, the GFP signals from both the SSm6A and RSVm6A reporters can be used as a readout of m^6^A methylation.

To identify SAM-independent RNA m^6^A regulatory mechanisms, we performed a lentiviral CRISPR-Cas9 gene knockout screen in HEK293T cells cultured in SAM-rich medium (Fig. 1b). The library included 50 negative control sgRNAs to ensure screening accuracy. By examining the enriched genes from two independent m^6^A reporter systems, we identified 37 genes enriched in both systems (Supplementary information, Fig. S1j). KEGG enrichment analysis revealed significant enrichment in “oxidative phosphorylation & NADH metabolism” and “one-carbon metabolism” (Supplementary information, Fig. S1k). Importantly, substances associated with NADH metabolism have been shown to directly influence demethylase activity,^27^ supporting the reliability of our screening method. In the one-carbon metabolism pathway, we identified AHCY, a key enzyme in the methionine cycle that substantially regulates mRNA m^6^A levels and serves as an important effector regulating mRNA m^6^A modification (Fig. 1c, d). However, other key genes in the methionine metabolic pathway, such as methionine adenosyltransferase (*MAT2A*), were not significantly enriched in both screening systems (Supplementary information, Tables S1, S2), suggesting that AHCY may regulate m^6^A levels in a SAM-independent manner. AHCY facilitates the reversible conversion of SAH to ADO and l-homocysteine (Hcy), supporting the one-carbon-unit transfer essential for biosynthesis, amino acid homeostasis, redox balance, and epigenetic regulation.^22^ In mammals, AHCY is uniquely responsible for this conversion. Although the essential metabolic roles of AHCY have been partially characterized,^17,22,28–30^ the potential moonlighting functions of AHCY and its involvement in SAM-independent methylation regulation remain to be discovered.

To further demonstrate that AHCY increases mRNA m^6^A methylation, we generated AHCY-knockout (KO) HEK293T and SW480 cells. The mRNA m^6^A content was significantly lower in AHCY KO cells than in control clones (Fig. 1e). The tight link between AHCY and mRNA m^6^A levels was confirmed in multiple cell lines with AHCY depletion (Fig. 1f; Supplementary information, Fig. S1l). Furthermore, ectopic overexpression of AHCY markedly increased mRNA m^6^A levels (Supplementary information, Fig. S1m). However, AHCY KO did not significantly affect expression of the MTases METTL3 and METTL14 or the demethylases FTO and ALKBH5 (Supplementary information, Fig. S1n, o). Methionine is rapidly converted to SAM, a principal methyl donor, via MAT2A in mammals^3,5^ (Fig. 1d). Intriguingly, although MAT2A depletion significantly reduced intracellular SAM levels compared with AHCY depletion, the reduction in mRNA m^6^A levels was more pronounced upon AHCY depletion (Fig. 1g–i). In addition, m^6^A RNA immunoprecipitation followed by high-throughput sequencing (MeRIP-seq) analysis of colorectal^31^ and ovarian cancer^32^ patient samples demonstrated a stronger positive correlation of mRNA m^6^A levels with AHCY than with other one-carbon metabolism genes (e.g., *MAT2A*) (Fig. 1j, k), suggesting the existence of a SAM-independent pathway through which AHCY regulates mRNA m^6^A modification. Furthermore, compared with other methionine metabolism enzymes, AHCY exhibited substantially greater upregulation in most tumor tissues, especially colorectal and lung cancer tissues, than in their normal counterparts (Supplementary information, Fig. S1p).

### Adenosine regulates mRNA m^6^A modification by binding AHCY

**Fig. 2:**
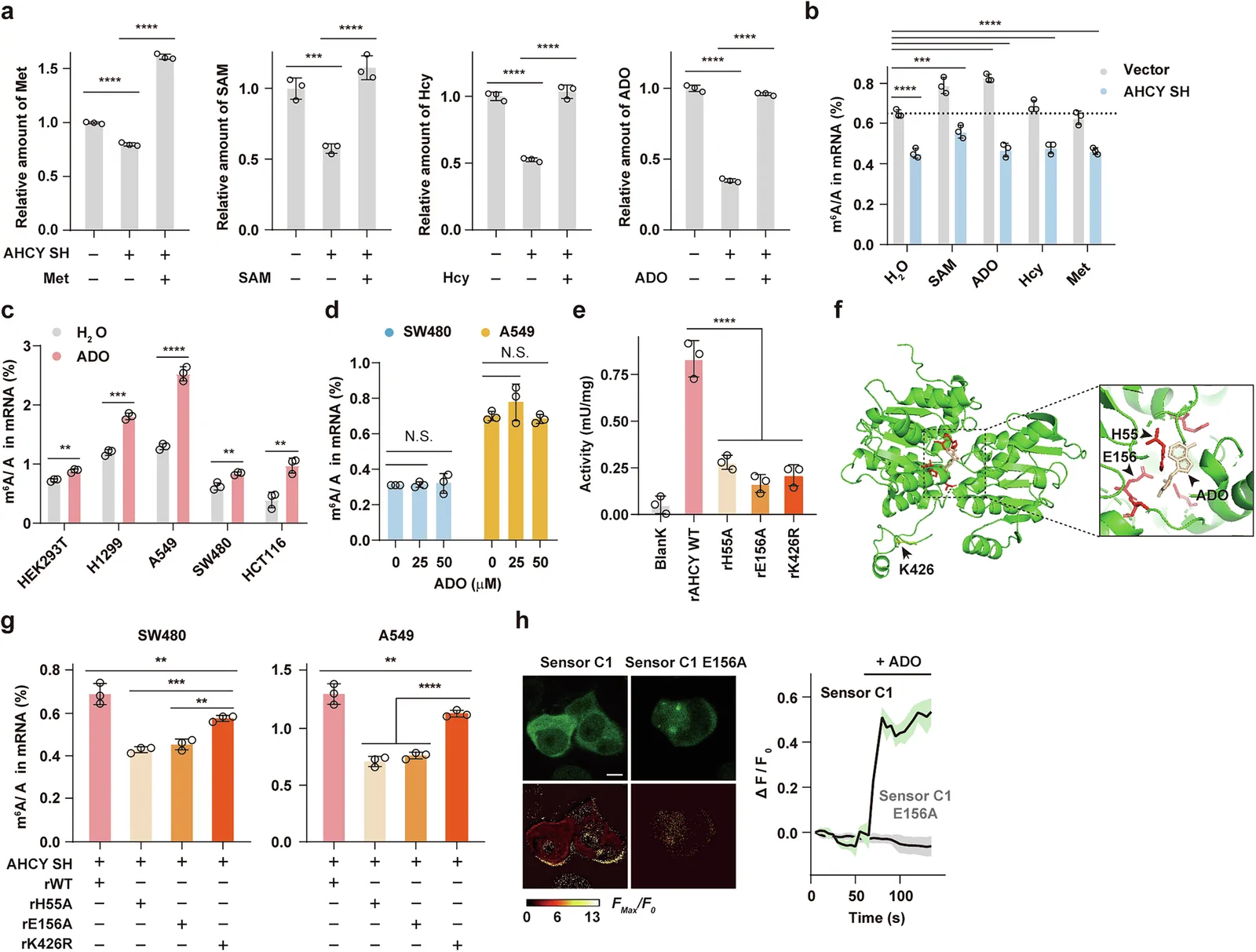
Adenosine regulates mRNA m^6^A by binding AHCY. **a** Quantitative analysis of metabolites in the methionine cycle measured in SW480 cells expressing vector or AHCY shRNA and treated with or without the indicated metabolites. **b** LC-MS/MS quantification of the mRNA m^6^A/A ratio in SW480 cells expressing vector or AHCY shRNA after treatment with metabolites in the methionine cycle for 12 h. **c** LC-MS/MS quantification of the mRNA m^6^A/A ratio in the indicated cells treated with or without adenosine (ADO) for 12 h. **d** LC-MS/MS quantification of the mRNA m^6^A/A ratio in SW480 and A549 AHCY shRNA-expressing cells treated with different concentrations of ADO for 12 h. **e** AHCY enzymatic activity in AHCY KO HEK293T cells re-expressing AHCY or the indicated mutants. **f** Molecular interactions within the ADO-binding pocket of the AHCY protein (PDB: 4PGF). Interacting residues are shown as sticks, with side chains colored red. **g** LC-MS/MS quantification of the mRNA m^6^A/A ratio in AHCY-depleted SW480 and A549 cells re-expressing AHCY WT and mutants. **h** Expression and responses of the AHCY-based ADO sensor (Sensor C1) and mutants in HEK293T cells. Images of sensor fluorescence before and after the application of 100 μM ADO (left). Representative traces and group analysis of fluorescence changes in cells expressing Sensor C1 in response to 100 μM ADO (right). F_max_, maximum fluorescence value; ΔF = F_t_ − F_0_; F_t_, fluorescence value at time t; F_0_, initial fluorescence value. Data are presented as mean ± S.D. (*n* = 3). The scale bar represents 10 μm. Two-tailed unpaired Student’s *t*-test (**c**). One-way ANOVA with LSD-*t* (**a**, **b**, **d**, **e**, **g**). **P < 0.01, ***P < 0.001, ****P < 0.0001, N.S., not significant.

To investigate the SAM-independent mechanism by which AHCY increases mRNA m^6^A levels, we first examined whether interference with methionine-cycle metabolites by AHCY depletion would affect mRNA m^6^A levels. Consistent with previous studies,^28,30,33^ AHCY knockdown significantly reduced the levels of methionine and its catabolic products (SAM, Hcy, and ADO) (Supplementary information, Fig. S2a). Interestingly, replenishment of methionine and its catabolic products to physiological concentrations following AHCY knockdown did not completely restore m^6^A levels (Fig. 2a, b). The SAM:SAH ratio influences the activity of m^6^A methyltransferases.^5^ Although SAM supplementation could fully restore the reduced SAM/SAH ratio and completely reverse the methylation levels resulting from AHCY depletion, it could not fully rescue the downregulated mRNA m^6^A levels caused by AHCY depletion (Supplementary information, Fig. S2b–d). This result suggests that, in addition to the possibility that reduced SAM levels may partially inhibit the activity of methyltransferases (e.g., METTL3/METTL14),^5,22^ other mechanisms may contribute to the regulation of mRNA m^6^A modifications. Notably, we unexpectedly found that ADO could significantly increase mRNA m^6^A levels in various cell lines (Fig. 2b, c). ADO metabolism involves both intracellular and extracellular metabolic pathways.^21^ Knockdown of the extracellular ADO metabolic enzymes CD39 and CD73 slightly reduced mRNA m^6^A levels, but interference with ADO receptors did not affect m^6^A levels (Supplementary information, Fig. S2e). In addition, we discovered that ADO deaminase (ADA), an intracellular ADO metabolism-dependent enzyme that irreversibly catalyzes the deamination of ADO to inosine, can significantly affect intracellular ADO and mRNA m^6^A levels (Supplementary information, Fig. S2f–k). These results suggest that the stimulatory effect of ADO on mRNA m^6^A modification is mediated through an ADO receptor-independent pathway.

To investigate the relationship between ADO-mediated increases in mRNA m^6^A and AHCY levels, we treated AHCY-depleted cells with ADO and found that ADO did not significantly increase mRNA m^6^A levels (Fig. 2d). However, ADO still significantly increased mRNA m^6^A levels in ADA-depleted cells (Supplementary information, Fig. S2l). Expression of shRNA-resistant AHCY (rAHCY) fully reversed the reduction in m^6^A levels induced by AHCY knockdown, excluding the possibility of off-target effects of AHCY shRNA (Supplementary information, Fig. S2m, n). Using the structure of AHCY (PDB: 4PGF),^29^ we analyzed the binding pocket for ADO binding to the AHCY protein (Supplementary information, Fig. S2o). We then constructed AHCY mutants with mutations in known ADO-binding residues (H55, E59, E156, and T157)^29,34^; the hydrolase activity of these mutants was lost (Supplementary information, Fig. S2p, q), and their expression failed to fully reverse the reduced m^6^A levels in AHCY-depleted cells (Supplementary information, Fig. S2n). Considering that mutations in the ADO-binding site of the AHCY protein also affected its activity, we constructed another AHCY mutant (K426R)^35^ that lacked activity but retained ADO-binding capability (Fig. 2e, f; Supplementary information, Fig. S2q, r) to confirm that ADO regulates mRNA m^6^A modification by binding AHCY (Fig. 2g).

The AHCY–ADO complex was first recognized in 1978, when the mutual high binding affinity of the two molecules was observed.^36^ Given that AHCY catalyzes the reversible hydrolysis of SAH to ADO and Hcy (Fig. 1d), we designed a sensor that can instantaneously track the AHCY–ADO complex. The sensor was developed through an established ADO sensor development pipeline.^23^ We initially used linker peptides to insert a conformation-sensitive circularly permuted enhanced GFP (cpEGFP) sequence into the specific loop region of AHCY, which undergoes the most pronounced changes upon ADO binding (Supplementary information, Fig. S2s). We then further optimized the linker peptides on the basis of the well-developed GRABAdo 1.0 sensor (Ado.1)^23^ and identified the sensor with the greatest fluorescence response, which we named Sensor C1 (Supplementary information, Fig. S2t, u and Data S1). Sensor C1, but not a non-ligand-binding mutant (Sensor C1 E156A), generated a peak response of ~40% in HEK293T cells treated with saturating concentrations of ADO (Fig. 2h; Supplementary information, Video S1), suggesting that Sensor C1 met the requirements for visualization of the AHCY–ADO complex.

### The AHCY–adenosine complex regulates mRNA m^6^A by binding to FTO

**Fig. 3:**
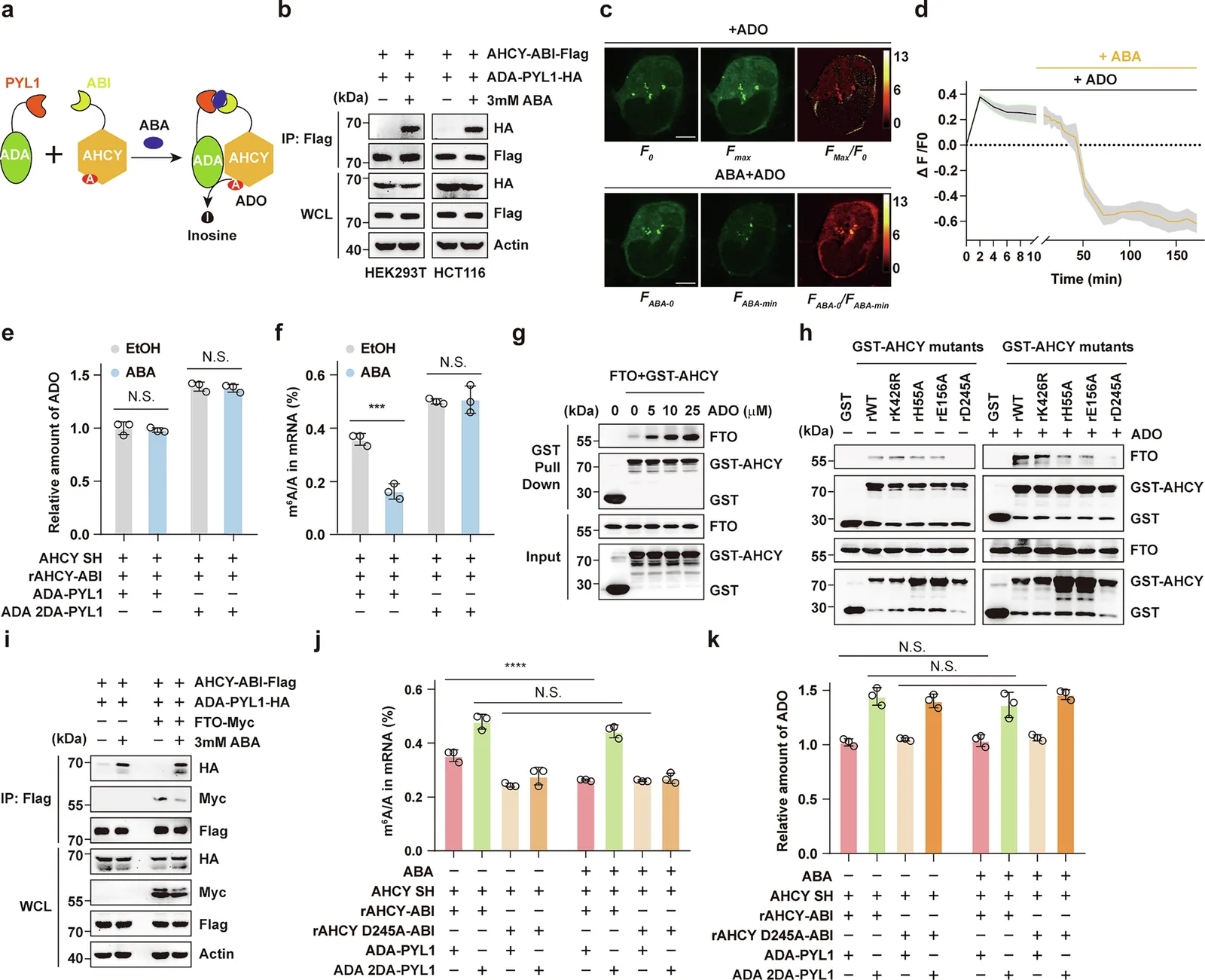
The AHCY–adenosine complex regulates mRNA m^6^A modification by binding FTO. **a** Schematic diagram of the ABA-inducible ABI/PYL1 system for targeting the AHCY–ADO complex through co-expression of ABI-AHCY and PYL1-ADA. **b** Western blot analysis of whole cell lysates (WCLs) and anti-Flag immunoprecipitates from HEK293T and HCT116 cells expressing the indicated proteins and treated with or without ABA. **c** Expression and responses of the AHCY-based ADO sensor (Sensor C1) in HEK293T cells. Images of sensor fluorescence before and after application of 100 μM ADO (upper) followed by 3 mM ABA (bottom). F_max_, maximum fluorescence value; F_0_, initial fluorescence value; F_min_, minimum fluorescence value. The scale bars represent 5 μm. **d** Representative traces and group analysis of fluorescence changes in cells expressing Sensor C1 in response to 100 μM ADO followed by 3 mM ABA. ΔF = F_t_ − F_0_; F_t_, fluorescence value at time t; F_0_, initial fluorescence value. **e** Quantitative analysis of intracellular ADO in HCT116 cells expressing the indicated proteins and treated with or without 3 mM ABA for 24 h. **f** LC-MS/MS quantification of the mRNA m^6^A/A ratio in HCT116 cells expressing the indicated proteins and treated with or without 3 mM ABA for 24 h. **g** Pull-down assays were performed by mixing purified recombinant His-FTO (2 μg), GST-AHCY (2 μg), and increasing concentrations of ADO, followed by incubation for 4 h. **h** Purified recombinant GST-AHCY (WT) or GST-AHCY mutants (2 μg) were incubated with His-FTO (2 μg) for 4 h in the absence or presence of 10 μM ADO, followed by pull-down assays. **i** Western blot analysis of WCLs and anti-Flag immunoprecipitates from A549 cells expressing the indicated proteins and treated with or without ABA for 3 h. **j** LC-MS/MS quantification of the mRNA m^6^A/A ratio in AHCY-depleted HCT116 cells re-expressing the indicated proteins and treated with or without 3 mM ABA for 12 h. **k** Quantitative analysis of intracellular ADO in AHCY-depleted HCT116 cells re-expressing the indicated proteins and treated with or without 3 mM ABA for 12 h. Data are presented as mean ± S.D. (*n* = 3). Two-tailed unpaired Student’s *t*-test (**e**, **f**). One-way ANOVA with LSD-*t* (**j**, **k**). ***P < 0.001, ****P < 0.0001, N.S., not significant.

To determine whether the AHCY–ADO complex is a pivotal determinant in regulating mRNA m^6^A levels, we engineered a chemically induced proximity system for transient regulation of the AHCY–ADO complex, specifically an abscisic acid (ABA)-triggered ABI/PYL1 system (Fig. 3a; Supplementary information, Data S2). ABA, a small molecule present in plants, significantly enhances the interaction between PYL1 and ABI.^37–40^ ADA and the AHCY protein did not interact in cells (Fig. 3b). Remarkably, upon treatment with ABA, the ADA–PYL1 and AHCY–ABI fusion proteins exhibited a significant interaction, and the fluorescence response induced by the AHCY–ADO complex decreased progressively (Fig. 3b–d; Supplementary information, Video S2). To confirm the ability of the chemically induced proximity system to reduce the abundance of ADO in complex with AHCY, we used a well-documented, validated ADO sensor (GRABAdo.1) (Supplementary information, Fig. S3a). As reported previously, GRABAdo.1^23^ exhibited a robust GRAB–ADO complex-induced fluorescence response upon ADO addition (Supplementary information, Fig. S3b, c). As expected, the ADO-induced fluorescence response decreased with ABA addition (Supplementary information, Fig. S3b, c and Video S3), suggesting the feasibility and effectiveness of the chemically induced proximity system coupled with the ADO sensor. Although ABA treatment did not affect the overall ADO level (Fig. 3e), ABA-induced degradation of ADO significantly reduced mRNA m^6^A levels in the AHCY–ADO complex proximity system (Fig. 3f) but not in the GRAB–ADO complex proximity system (Supplementary information, Fig. S3d). By contrast, m^6^A levels did not change significantly when ABA was added to the ADA inactive mutant fusion system (Fig. 3f). These findings indicate that the AHCY–ADO complex may be the key factor regulating mRNA m^6^A levels, rather than the overall ADO level in cells.

In vitro m^6^A modification assays excluded intrinsic MTase activity in AHCY–ADO complexes (Supplementary information, Fig. S3e). Proteomic analysis of AHCY-associated proteins suggested a potential interaction with the demethylase FTO, rather than with the MTases METTL3/METTL14 or the demethylase ALKBH5 (Supplementary information, Fig. S3f–h). Moreover, purified recombinant glutathione *S*-transferase (GST)-tagged AHCY bound directly to purified recombinant His-tagged FTO (Supplementary information, Fig. S3i). In cohorts of patients with colorectal^31^ and ovarian cancer,^32^ a more significant negative correlation was observed between FTO expression levels and m^6^A levels after standardizing both cohorts on the basis of AHCY (Supplementary information, Fig. S3j, k). Furthermore, FTO overexpression reversed the increase in mRNA m^6^A levels induced by AHCY re-expression (Supplementary information, Fig. S3l). However, the absence of FTO did not affect the hydrolase activity of AHCY (Supplementary information, Fig. S3m). Consistent with previous studies,^22,41^ both AHCY and FTO were distributed in the cytoplasm and nucleus (Supplementary information, Fig. S3n). We also detected the AHCY–FTO interaction in both compartments (Supplementary information, Fig. S3n–p).

To confirm that AHCY modulates mRNA m^6^A modification through its interaction with FTO, we analyzed the AHCY–FTO interaction sites. Analysis of the AHCY protein, which is divided into four functional domains,^22,34^ revealed that only the AHCY AA1–183 and AA184–356 regions bind to the FTO protein (Supplementary information, Fig. S3q, r). We then used SPPIDER (http://sppider.cchmc.org/) to identify the surface-exposed residues within this region and systematically mutated each residue to investigate potential alterations in protein‒protein binding affinity.^42^ AHCY D245, H162, and Q251 were identified as crucial residues (Supplementary information, Fig. S3s). Notably, only the D245A mutation did not impede the enzymatic activity of AHCY (Supplementary information, Fig. S3t). However, the AHCY D245A mutant could not interact with FTO or restore the reduced m^6^A levels in AHCY-depleted cells (Supplementary information, Fig. S3u, v). These data suggest that AHCY may increase mRNA m^6^A modification through its interaction with the demethylase FTO.

Next, to examine the role of ADO in the AHCY–FTO interaction, we added various concentrations of ADO to mixtures of purified recombinant AHCY and FTO proteins in vitro. ADO markedly increased the interaction between AHCY and FTO (Fig. 3g). By contrast, supplementation with SAH, another substrate of AHCY, did not significantly alter this interaction (Supplementary information, Fig. S3w). Furthermore, ADO substantially increased the interactions of wild-type (WT) and K426R-mutant AHCY with FTO but not the interactions of the H55A, E156A, and D245A mutants with FTO (Fig. 3h), suggesting that formation of the AHCY–ADO complex significantly increases the interaction of AHCY with FTO. Intriguingly, ABA treatment reduced the abundance of the AHCY–ADO complex in the proximity system (Fig. 3a–d), thereby significantly reducing the AHCY–FTO interaction and mRNA m^6^A levels. By contrast, neither the AHCY–FTO interaction nor m^6^A levels were altered in the ADA 2DA–PYL1 group (Fig. 3i, j; Supplementary information, Fig. S3x). Indeed, the chemically inducible proximity system considerably reduced the abundance of the AHCY–ADO complex (Fig. 3a–d) but did not affect overall ADO content (Fig. 3k). These findings confirm that the interaction between AHCY–ADO complexes and FTO increases RNA m^6^A modification.

### The AHCY–adenosine complex increases AHCY dimerization to inhibit FTO activity

**Fig. 4:**
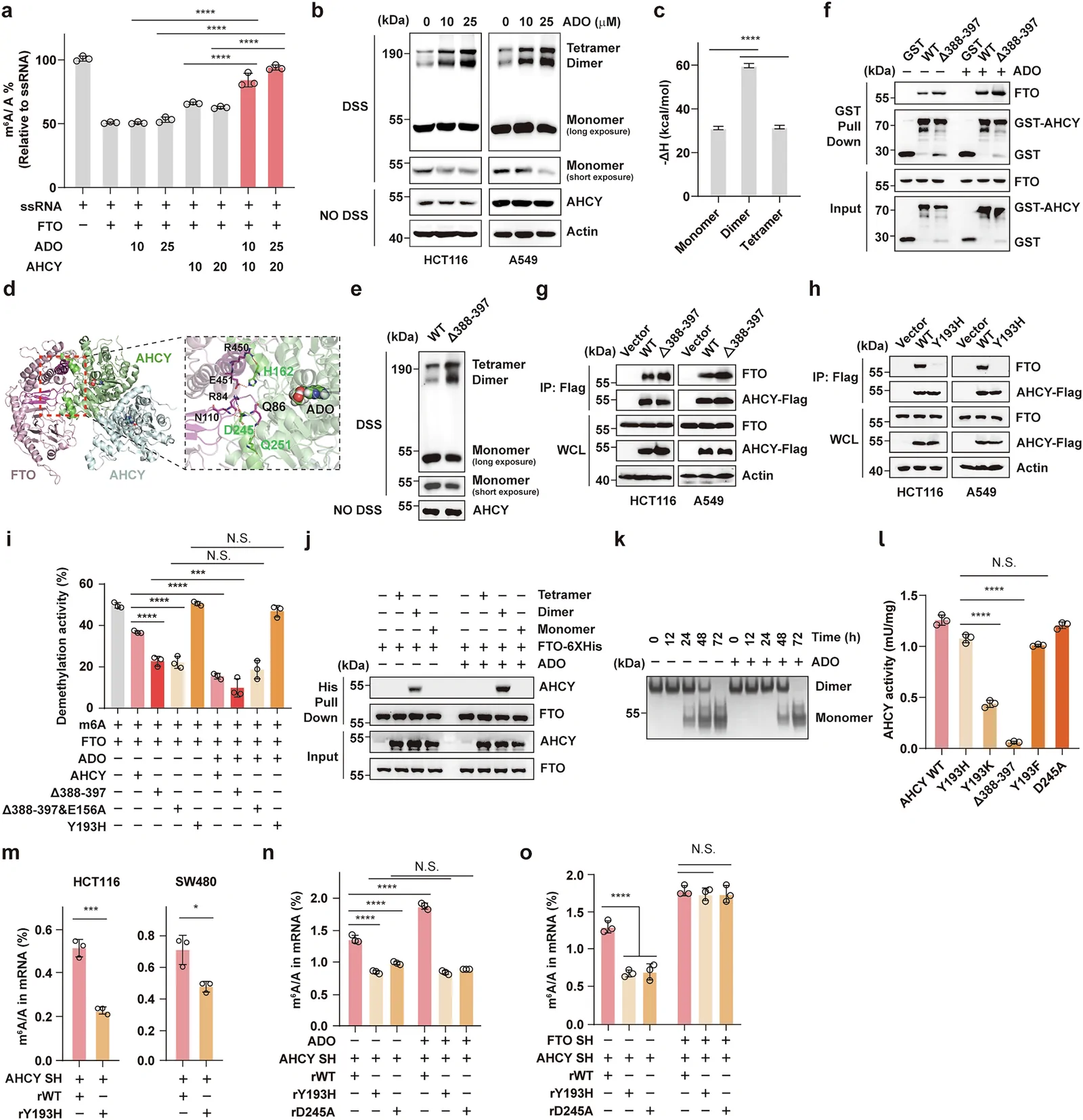
The AHCY–adenosine complex increases AHCY dimerization to inhibit FTO activity. **a** Demethylation of m^6^A in post-FTO reaction mixtures in vitro. With H_2_O as a mock control, purified recombinant AHCY and/or ADO was added to the standard reaction mixture to the indicated concentrations, and the mixtures were then analyzed by LC-MS/MS. **b** HCT116 and A549 cells were cultured in complete medium (CM) and then transferred to media containing different concentrations of ADO for an additional 4 h. The oligomerization state of endogenous AHCY in cells was analyzed using disuccinimidyl suberate for protein cross-linking. **c** Molecular docking of FTO (PDB: 3LFM) with various AHCY oligomers (PDB: 3NJ4 and 1LI4). The binding enthalpies (ΔH, kcal/mol) of FTO with AHCY in various oligomerization states, as determined by molecular docking, are shown. **d** FTO and AHCY dimers are shown as cartoons and are colored purple and green or blue, respectively. The critical residues in AHCY and FTO involved in their interaction are shown in stick representation and colored black and green, respectively. **e** Analysis of the oligomerization state of AHCY WT or the Δ388–397 mutant using disuccinimidyl suberate for protein cross-linking. **f** Pull-down assays were performed by mixing purified recombinant His-FTO (2 μg) and GST-AHCY (2 μg) or the GST-AHCY Δ388–397 mutant (2 μg) and incubating the mixture with or without 10 μM ADO for 4 h. **g** Western blot analysis of co-immunoprecipitates of Flag-tagged AHCY or the Δ388–397 mutant with endogenous FTO in the indicated cells. **h** Western blot analysis of co-immunoprecipitates of Flag-tagged AHCY or the indicated mutants with endogenous FTO in the indicated HCT116 and A549 cells. **i** Demethylation of m^6^A in the post-FTO reaction mixtures in vitro. With H_2_O as a mock control, purified recombinant AHCY or the indicated mutant was added to the standard reaction mixture for FTO demethylation in the presence or absence of 10 μM ADO, and the reaction products were then analyzed by LC-MS/MS. **j** Pull-down assays were performed by mixing recombinant His-FTO (2 μg) and AHCY monomers (2 μg), dimers (2 μg), or tetramers (2 μg) obtained by separation via gel filtration and incubating the mixture with or without 10 μM ADO for 4 h. **k** Recombinant AHCY dimers were purified by gel filtration with or without ADO, incubated at 37 °C for the indicated times, and then analyzed by native PAGE. **l** Enzymatic activity of purified recombinant AHCY WT and the indicated mutants in vitro (*n* = 6). **m** LC-MS/MS quantification of the mRNA m^6^A/A ratio in AHCY-depleted HCT116 and SW480 cells re-expressing WT or Y193H mutant AHCY. **n**, **o** LC-MS/MS quantification of the mRNA m^6^A/A ratio in the indicated A549 cells cultured in the presence or absence of 25 μM ADO for 12 h (**n**) and with or without FTO shRNA transduction (**o**). Data are presented as mean ± S.D. (*n* = 3). Two-tailed unpaired Student’s *t*-test (**c**, **m**). One-way ANOVA with LSD-*t* (**a**, **i**, **l**, **n**, **o**). *P < 0.05, ***P < 0.001, ****P < 0.0001, N.S., not significant.

To investigate whether the AHCY–ADO complex interacts with FTO to directly influence its demethylase activity, we used liquid chromatography‒tandem mass spectrometry (LC-MS/MS) to test FTO activity in vitro.^11^ The retention-time peak and secondary mass spectral peak of ssDNA m^6^A decreased gradually upon FTO treatment (Supplementary information, Fig. S4a). In addition, FTO activity was significantly inhibited by combined treatment with ADO and AHCY compared with either treatment alone (Fig. 4a), suggesting that the AHCY–ADO complex considerably suppresses FTO demethylase activity and upregulates m^6^A modification (Fig. 3j). AHCY proteins exist as monomers and as various oligomers, including dimers and tetramers (Fig. 4b; Supplementary information, Fig. S4b), with tetramers being the most active.^22,33^ Notably, we observed that ADO substantially reduced the abundance of AHCY monomers, thereby increasing the abundance of AHCY dimers and tetramers, whereas SAH, another AHCY substrate, had no effect on these forms (Fig. 4b; Supplementary information, Fig. S4c). Indeed, methionine restriction significantly reduced intracellular ADO levels (Supplementary information, Fig. S1a) and impeded AHCY dimer and tetramer formation (Supplementary information, Fig. S4d).

To investigate whether different AHCY oligomers exhibit different binding affinities for FTO, we performed molecular dynamics analyses of the interactions between various AHCY oligomers and FTO (Supplementary information, Fig. S4e). The binding between FTO and AHCY dimers was significantly stronger than that between FTO and AHCY monomers or tetramers, implying that the interface with AHCY dimers could be critical for the interaction with FTO (Fig. 4c; Supplementary information, Fig. S4e). Furthermore, consistent with the co-immunoprecipitation results (Supplementary information, Fig. S3s), molecular dynamics analysis revealed that AHCY residues Q251, H162, and D245 were located at the site of the AHCY–FTO interaction (Fig. 4d). The AHCY Δ184–187 and Δ190–207 mutations^34^ disrupted the dimerization and tetramerization of AHCY, weakened its interaction with FTO, and failed to reverse the reduction in mRNA m^6^A levels of tumor cells caused by AHCY knockdown (Supplementary information, Fig. S4f–j). Compared with AHCY WT, the Δ388–397 mutant^22,34^ exhibited substantial increases in dimerization and interaction with FTO, both in vitro (Fig. 4e, f) and in tumor cells (Fig. 4g; Supplementary information, Fig. S4k). By contrast, the AHCY Y193H mutant^22,34^ exhibited considerably reduced dimerization and interaction with FTO in tumor cells, but its tetramerization was maintained (Fig. 4h; Supplementary information, Fig. S4l). Strikingly, ADO significantly enhanced the dimerization of purified recombinant AHCY WT, increased its interaction with purified recombinant FTO protein, and inhibited FTO activity (Fig. 4f, i; Supplementary information, Fig. S4m). Furthermore, ADO still significantly increased the interaction between AHCY dimers and FTO and suppressed FTO activity (Fig. 4i, j; Supplementary information, Fig. S4n). However, it did not enhance the interaction between the AHCY Δ388–397&E156A mutant and FTO or inhibit FTO activity (Fig. 4i; Supplementary information, Fig. S4m). This effect may be attributed to the increased affinity between AHCY and FTO facilitated by the AHCY–ADO complex, which also contributes to AHCY dimer stability (Fig. 4k; Supplementary information, Fig. S4o, p).

The AHCY Y193H mutation slightly impaired AHCY activity, with an effect comparable to that of the AHCY Y193F mutation, which is not genetically linked to hypermethioninemia (https://www.ebi.ac.uk/ProtVar/); however, the AHCY Δ388–397 mutation significantly impaired AHCY hydrolase activity (Fig. 4l). Notably, the AHCY Δ388–397 mutant still restored mRNA m^6^A levels in tumor cells to AHCY WT levels, significantly higher than those of the AHCY E156A mutant (Supplementary information, Fig. S4q). However, FTO depletion nearly eliminated these differences between the AHCY Δ388–397 mutant and other mutants (AHCY E156A or K426R) (Supplementary information, Fig. S4q). Similarly to the AHCY rD245A mutant (Supplementary information, Fig. S3v), the AHCY Y193H mutant retained hydrolase activity and maintained the SAH/SAM ratio (Fig. 4l; Supplementary information, Fig. S4r) but failed to fully rescue the AHCY depletion-induced reduction in mRNA m^6^A levels (Fig. 4m). Therefore, to eliminate potential interference from AHCY activity on FTO/m^6^A signaling regulation, the Y193H and D245A mutants were selected for subsequent experiments. The AHCY Y193H and D245A mutations abolished the ADO-induced increase in mRNA m^6^A modification (Fig. 4n; Supplementary information, Fig. S4s). Similarly, depletion of FTO abolished, whereas loss of ALKBH5 failed to eliminate, the differences in mRNA m^6^A levels between AHCY WT and its Y193H and D245A mutants (Fig. 4o; Supplementary information, Fig. S4t–v), suggesting that the AHCY–ADO complex influences mRNA m^6^A modification through the binding of the AHCY dimer to FTO.

### The AHCY–adenosine complex disrupts the binding of FTO to RNA

**Fig. 5:**
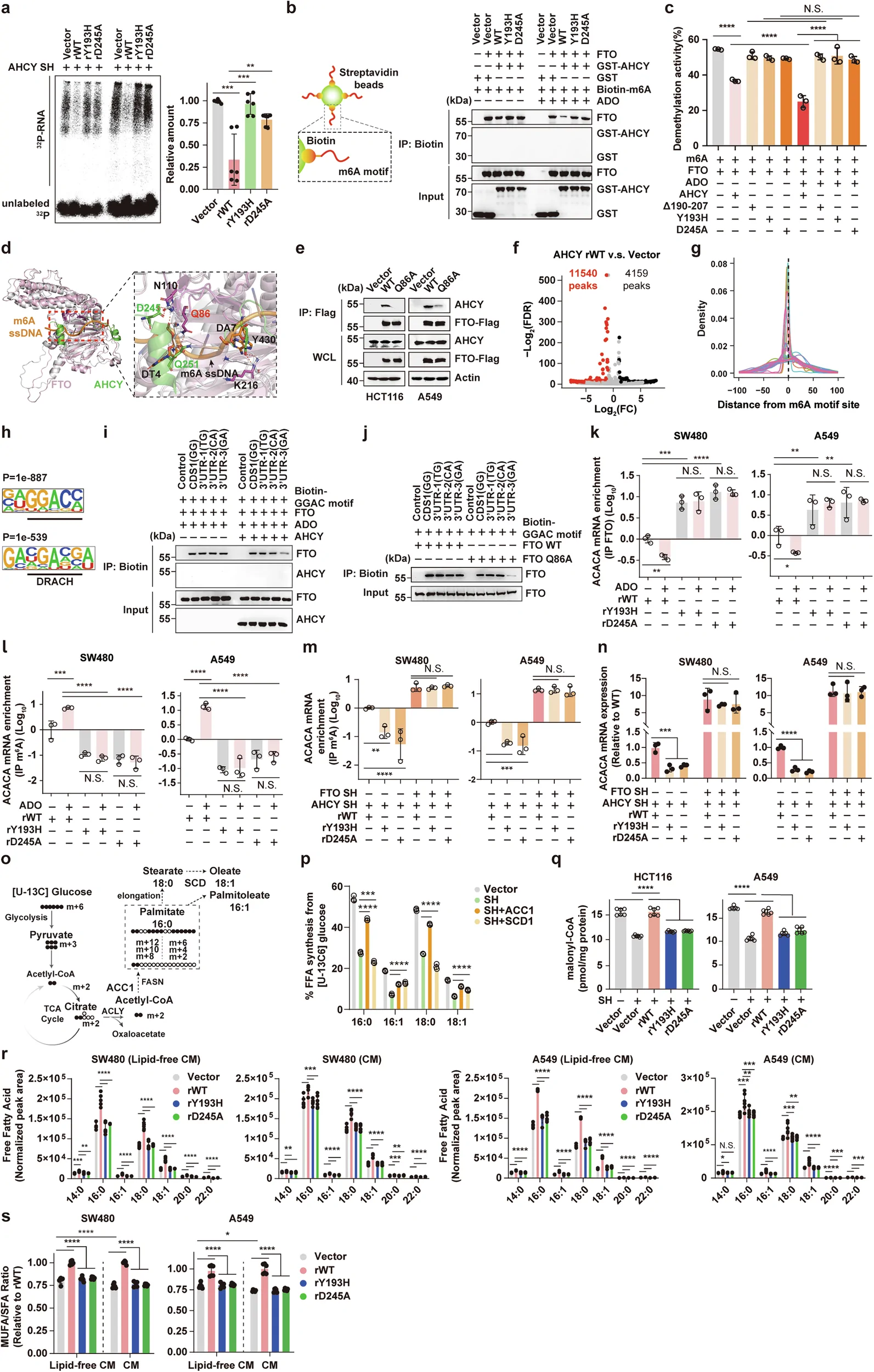
The AHCY–adenosine complex disrupts the binding of FTO to RNA. **a** Two representative ^32^P autoradiograms from CLIP of FTO-HA pulled down from AHCY KO HCT116 cells re-expressing AHCY WT or the indicated mutants using a monoclonal anti-HA antibody (*n* = 6). **b** Purified recombinant His-FTO and GST-AHCY WT or the indicated mutants (5 μg) were incubated overnight with the biotin-m^6^A motif fragment (200 nM) in the presence or absence of 10 μM ADO. Pulldown assays were performed with streptavidin beads. **c** Demethylation of m^6^A in the post-FTO reaction mixtures in vitro. With H_2_O as a mock control, purified recombinant AHCY or the indicated mutant was added to the standard reaction mixture for FTO demethylation in the presence or absence of 10 μM ADO, and the reaction products were then analyzed by LC-MS/MS. **d** Alignment of the FTO–ssDNA complex (PDB: 5ZMD) and the FTO–AHCY dimer complex in Fig. 4f. FTO proteins, ssDNA, and AHCY proteins are shown as cartoons and are colored purple, yellow, and green, respectively. **e** Western blot analysis of co-immunoprecipitation of FTO-Flag or the indicated mutants with endogenous AHCY in the indicated HCT116 and A549 cells. **f** Significantly decreased (red dot) or increased (black dot) FTO binding peaks were identified in AHCY-depleted A549 cells upon AHCY re-expression (false discovery rate (FDR) < 0.0001 and FC > 2). **g** A density plot shows the distribution of motifs enriched in the Vector group from FTO CLIP-seq, together with their respective distances from the m^6^A motif DRACH. Each color corresponds to a motif. **h** Motifs harboring the DRACH sequence identified by HOMER de novo analysis from FTO CLIP-seq peaks enriched in the Vector group. **i** Purified recombinant FTO (5 μg) and AHCY (5 μg) proteins were incubated overnight with different sequences of biotin-GG(m^6^A)C motif fragments (200 nM; GGGGAC, TGGGAC, CAGGAC, or GAGGAC). Pull-down assays were performed with streptavidin beads. **j** Purified recombinant FTO (5 μg) and the indicated mutant proteins (5 μg) were incubated overnight with different sequences of biotin-GG(m^6^A)C motif fragments (200 nM). **k**, **l** AHCY-depleted A549 or SW480 cells re-expressing WT or mutant AHCY were treated with or without 25 μM ADO for 12 h. Relative precipitated RNA levels were normalized to those in non-ADO-treated cells expressing WT AHCY. **m** AHCY-depleted SW480 and A549 cells re-expressing WT or mutant AHCY with or without FTO shRNA transduction. Relative precipitated RNA levels were normalized to those in cells expressing WT AHCY. RNA immunoprecipitation assays were performed in these cells using anti-FTO (**k**) or anti-m^6^A (**l**, **m**) antibodies, and qPCR analysis of precipitated *ACACA* mRNA was then performed. **n** The mRNA levels of *ACACA* in AHCY-depleted SW480 and A549 cells re-expressing WT or mutant AHCY with or without FTO shRNA transduction were examined by qPCR. **o** Schematic  diagram depicting the incorporation of carbons from uniformly-labeled ^13^C-glucose (U-13C-glucose, indicated by the black filled circles) into the TCA cycle, as well as palmitate, stearate, palmitoleate, and oleate. Representative palmitate isotopologs are depicted. **p** Steady-state palmitic acid (16:0), palmitoleic acid (16:1), stearic acid (18:0), and oleic acid (18:1) labeling from U-^13^C-glucose in the indicated A549 tumor cells cultured in lipid-free CM for 24 h. FA taken up exogenously were also incorporated into cellular FFAs but were not labeled. **q** Quantification of malonyl-CoA in AHCY-depleted HCT116 and A549 cells re-expressing WT AHCY or the indicated mutants. **r** GC-MS/MS quantification of the normalized abundances of FFAs in AHCY-depleted A549 and SW480 cells re-expressing WT or mutant AHCY cultured in lipid-free CM or regular CM (*n* = 6). **s** The ratios of MUFAs (C16:1 and C18:1) to SFAs (C16:0 and 18:0), representing SCD1 activity in AHCY-depleted A549 and SW480 cells re-expressing WT or mutant AHCY cultured in lipid-free CM or regular CM, as measured by GC-MS/MS (*n* = 6). Data are prese*n*ted as mean ± S.D. (*n* = 3, unless otherwise specified). One-way ANOVA with LSD-*t* (**a**, **c**, **k**–**n**, **p**–**s**). *P < 0.05, **P < 0.01, ***P < 0.001, ****P < 0.0001, N.S., not significant.

To mechanistically delineate how the AHCY–ADO complex interacts with FTO to influence its demethylase activity, we performed cross-linking immunoprecipitation (CLIP) of radiolabeled [γ-^32^P] RNA with HA-FTO in tumor cells. Only AHCY WT, not AHCY Y193H or D245A, inhibited the RNA binding of FTO (Fig. 5a). Moreover, in the presence of purified recombinant AHCY protein, ADO enhanced the disruptive effect of FTO on m^6^A ssRNA binding and suppressed FTO activity (Fig. 5b, c). However, the purified Y193H and D245A mutants did not exert these effects (Fig. 5b, c), suggesting that the AHCY–ADO complex enhances the AHCY–FTO interaction via dimerization of AHCY, thus blocking its association with FTO and mRNA m^6^A substrates and ultimately repressing its demethylase activity. Molecular dynamics analysis revealed that the AHCY dimer could submerge the RNA-binding domain of FTO, thus inhibiting its binding to RNA (Fig. 5d). Interestingly, Q86 of FTO, rather than the classical m^6^A binding and catalytic sites R96, Y108, and E234, bound to the AHCY dimers (Fig. 5d, e; Supplementary information, Fig. S5a). Previous studies have reported an association between the FTO residue Q86 and ssRNA.^43^ In addition, the FTO Q86A mutation did not affect FTO activity in specific cells,^43^ indicating the potential interference of AHCY with the substrate preference of FTO.

To test this hypothesis, we performed MeRIP-seq and FTO CLIP-seq analysis. The MeRIP-seq data confirmed the previously established core motif (DRACH; D = A, G, or U; R = A or G; A = m^6^A; H = A, C, or U) through clustering of significantly enriched sequences,^9,10,44^ suggesting that AHCY has no significant bias in affecting the FTO binding core motif (Supplementary information, Fig. S5b). Most of the changed peaks exhibited notably reduced FTO binding upon AHCY re-expression (Fig. 5f). We observed that AHCY significantly inhibited the FTO CLIP-seq peaks, which were notably upregulated in the corresponding MeRIP-seq peaks (Supplementary information, Fig. S5c, d). Next, we performed a motif search for FTO CLIP-seq peaks. Intriguingly, we observed a significant reduction in motif enrichment upon AHCY re-expression, particularly at the 2–5 nucleotide region preceding the m^6^A motif (DRACH) (Fig. 5g; Supplementary information, Fig. S5e), which aligns with the FTO Q86 binding site (Fig. 5d). Unexpectedly, we identified a specific VWDRACH RNA motif (V = G, A, C; W = A, T), especially GADRACH, as highly enriched at the FTO binding site, where AHCY plays a regulatory role (Fig. 5h; Supplementary information, Fig. S5f). Using two random forest models in the SRAMP prediction tool, we identified four canonical m^6^A modification sites within the mRNA, characterized by the DRACH motif and the GGAC consensus sequence, but with distinct upstream sequences (GG, TG, CA, or GA) (Supplementary information, Table S3). Interestingly, purified recombinant AHCY distinctly disrupted the binding of purified recombinant FTO to 3′ UTR fragment 3 (GADRACH) but not to the other fragments (Fig. 5i; Supplementary information, Table S3). Moreover, the purified recombinant FTO Q86A mutant lost its interaction with AHCY (Fig. 5e) and exhibited lower binding affinity for 3′ UTR fragment 3 but not for the other fragments (Fig. 5j), highlighting potential interference of AHCY with the binding of FTO to specific substrate sequences that contain identical m^6^A motifs, such as GADRACH, which preferentially bind to the FTO Q86 site.

KEGG pathway enrichment from MeRIP-seq analysis revealed that AHCY-disrupted FTO substrates modulate numerous oncogenic pathways, including PI3K-Akt, EGFR tyrosine kinase, AMPK signaling, and fatty acid (FA) biosynthesis pathways (Supplementary information, Fig. S5g). Subsequent analyses revealed that AHCY influences the expression of a subset of genes involved in fatty acid (FA) biosynthesis, including *ACACA*, *FASN*, *OLAH*, *SCD1*, *ACSL3*, *ELOVL1*, and *ELOVL7*, with *ACACA* and *SCD1* being predominantly affected. Conversely, the upregulation of exogenous FA transporters (e.g., CD36, FABP5) induced by AHCY depletion likely reflects a feedback mechanism compensating for reduced endogenous FA synthesis.^45,46^ Sterol regulatory element-binding proteins (SREBPs) are a family of transcription factors that play a crucial role in regulating lipogenesis.^46,47^ Notably, this influence appears to be independent of changes in SREBP1 or SREBP2 expression (Supplementary information, Fig. S5h–k). ACACA (encoded by *ACC1*), a cytosolic enzyme critical for de novo FA synthesis, and ACACB (encoded by *ACC2*), which is localized in the mitochondrial outer membrane, catalyze the production of malonyl-CoA, which inhibits carnitine palmitoyltransferase I, a key enzyme regulating mitochondrial FA uptake and oxidation.^47,48^ Interestingly, *AHCY* mRNA expression was more strongly correlated with *ACACA* than with *ACACB* mRNA expression across various tumor types (Supplementary information, Data S3). Gene set enrichment analysis (GSEA) confirmed that AHCY significantly reduced the binding of FTO to genes involved in FA biosynthesis (Supplementary information, Fig. S5l). Manual inspection of the FTO CLIP-seq gene-browser tracks revealed instances of FA-synthesis-related transcripts with reduced peak intensity in cells re-expressing AHCY, enabling us to easily identify the signature sequence preference of AHCY disrupting FTO-RNA binding, GADRACH (Supplementary information, Fig. S5m). Nevertheless, MeRIP-seq profiles revealed greater peak intensities for *ACACA* and *SCD1* in these cells, in contrast to the FTO CLIP-seq results (Supplementary information, Fig. S5m). Furthermore, compared with WT FTO, the FTO Q86A mutant exhibited significantly reduced binding to *ACACA* and *SCD1* mRNA (Supplementary information, Fig. S5n, o).

### The AHCY–adenosine complex promotes fatty acid biosynthesis and tumor growth

We next explored how the AHCY–ADO complex regulates FA biosynthesis genes and their associated functions through its interaction with FTO. ADO increased the capacity of purified recombinant AHCY to inhibit the interaction between purified recombinant FTO and the ACACA GG(m^6^A)C motif fragment, but this effect was not extended to the purified recombinant AHCY D245A and Y193H mutants (Supplementary information, Fig. S5p), indicating that the AHCY–ADO complex attenuates the affinity of FTO for mRNA transcripts of FA biosynthesis genes. Furthermore, in AHCY-depleted tumor cells, ADO reduced the interaction of FTO with *ACACA* and *SCD1* mRNA and increased m^6^A modification and expression of these mRNAs upon AHCY WT re-expression, but these effects were negligible in cells re-expressing the AHCY mutants (Fig. 5k, l; Supplementary information, Fig. S5q–s). FTO depletion abrogated the differences observed between WT and mutant AHCY (Fig. 5m, n), emphasizing that the AHCY–ADO complex significantly reduces the interaction of FTO with lipogenesis-related mRNAs and increases their m^6^A modification.

Acetyl-CoA is carboxylated by acetyl-CoA carboxylase (ACC) to form malonyl-CoA. Fatty acid synthase (FASN) then catalyzes a condensation reaction that converts malonyl-CoA to palmitic acid (16:0), the primary product of fatty acid synthesis. This 16-carbon saturated fatty acid (16:0) undergoes elongation and desaturation, during which stearoyl-CoA desaturase (SCD) desaturates saturated fatty acids (SFAs) to generate monounsaturated fatty acids (MUFAs). These reactions yield fatty acids of various chain lengths and saturation levels, such as palmitoleate (16:1), stearate (18:0), and oleate (18:1).^47,48^ To further assess the effect of AHCY on the de novo FA synthesis pathway, we traced the fate of glucose, a major substrate for FA biosynthesis in cancer (Fig. 5o). Increased ACC1 expression partially reversed the reduction in ^13^C-glucose-derived free fatty acids (FFAs) caused by AHCY knockdown (Fig. 5p; Supplementary information, Fig. S5t, u), and SCD1 expression led to an increase in MUFA levels (16:1 and 18:1) (Fig. 5p; Supplementary information, Fig. S5u), underscoring the metabolic significance of AHCY for FFA homeostasis.

The AHCY Y193H and D245A mutants retained AHCY enzymatic activity (Fig. 4l; Supplementary information, Fig. S5v). Notably, AHCY WT re-expression rescued AHCY depletion-induced reductions in malonyl-CoA, FFA levels, and MUFA:SFA ratios, but the Y193H and D245A mutants were ineffective (Fig. 5q–s). However, there were no significant differences in acetyl-CoA levels between WT and mutant AHCY variants (Supplementary information, Fig. S5w). In addition, a ^13^C-labeled glucose tracer analysis demonstrated that both Y193H and D245A mutants significantly suppressed de novo FA synthesis compared with AHCY rWT re-expression (Supplementary information, Fig. S5x). Interestingly, AHCY depletion markedly impaired FA biosynthesis in lipid-free medium relative to regular complete medium (Fig. 5r; Supplementary information, Fig. S5t). Conversely, MUFA proportions decreased substantially under both conditions (Fig. 5s). These results confirm that the AHCY–ADO complex governs the expression of FA biosynthetic genes through the FTO/m^6^A signaling pathway, thereby maintaining FFA homeostasis. This regulatory mechanism is closely linked to the utilization of exogenous fatty acids by tumor cells, while operating independently of precursor acetyl-CoA.

**Fig. 6:**
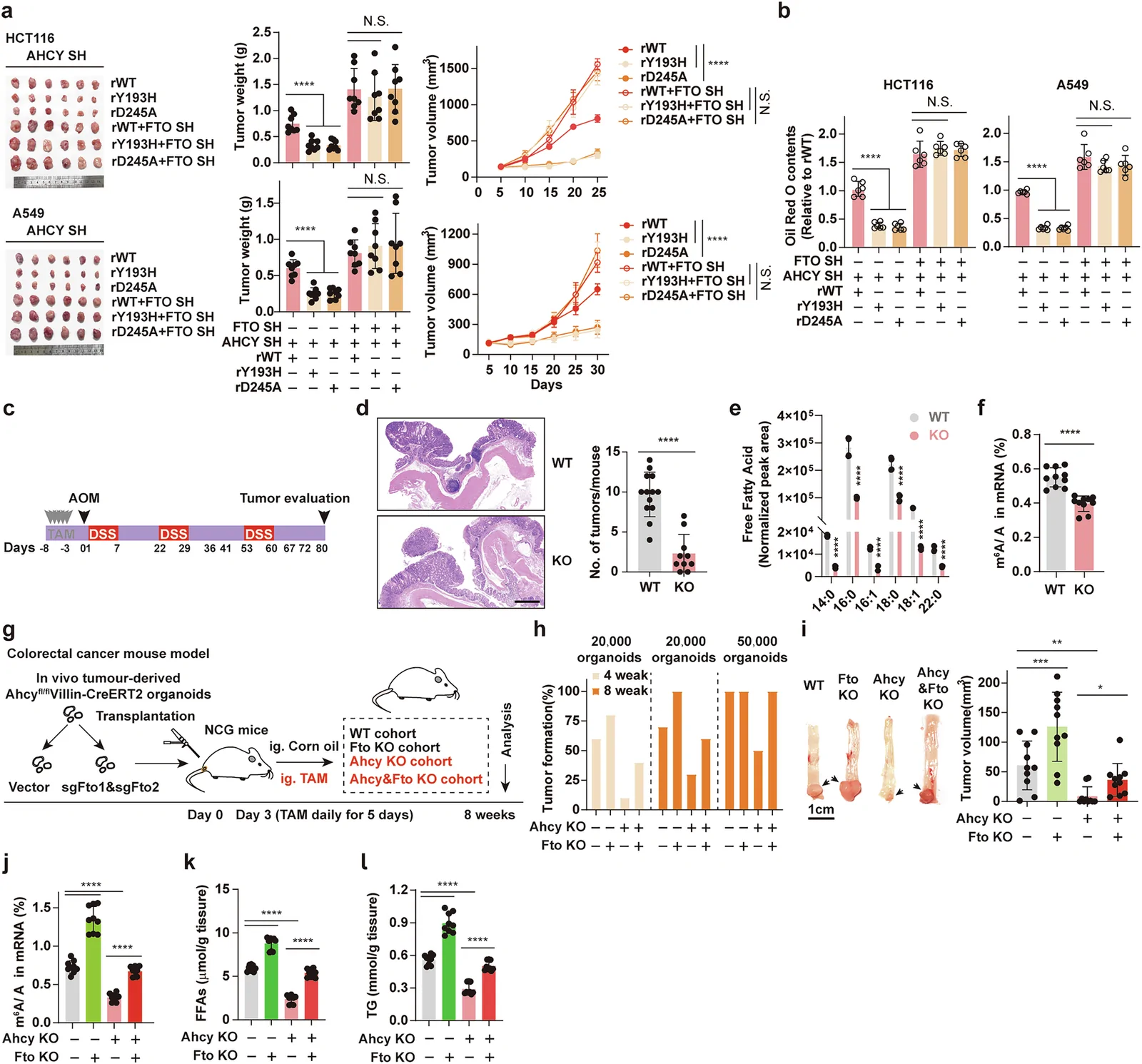
The AHCY-FTO axis promotes tumor growth through lipid metabolic reprogramming. **a** Representative images, weights, and volumes of xenograft tumors formed by AHCY-depleted HCT116 and A549 cells re-expressing AHCY WT or the indicated mutants with or without FTO shRNA transduction in immunocompromised mice (*n* = 7–9). **b** Quantitation of the Oil Red O content in AHCY-depleted HCT116 and A549 cells re-expressing WT AHCY or the indicated mutants with or without FTO shRNA transduction (*n* = 6). **c** Schematic design of the model of azoxymethane (AOM)/DSS-induced colon cancer. **d** Representative hematoxylin and eosin (H&E) staining of mouse COAD tumor tissues collected from the WT and KO cohorts. The scale bar represents 500 μm. Numbers of tumors harvested from the WT and KO cohorts (*n* = 10–15 mice per group). **e**, **f** GC-MS/MS quantification of the normalized abundance of FFAs (**e**) and LC-MS/MS quantification of the mRNA m^6^A/A ratio (**f**) of tumors harvested from colorectal cancer tissues of mice in the WT and KO cohorts (*n* = 6–10 mice per group). **g** Schematic representation of naive colorectal cancer organoids, derived from spontaneous tumors in *Ahcy*^*fl/fl*^ Villin-CreERT2 genotype mice induced by AOM-DSS, infected with or without lentivirus carrying Fto sgRNA (sgFto1&sgFto2) and intestinally inoculated in situ by colonoscopy-based orthotopic transplantation. **h**, **i** Tumor formation rate (**h**), representative images (**i**, left), and tumor size (**i**, right) after orthotopic transplantation of sgVector and sgFto1&sgFto2 colorectal cancer organoids into the colons of NCG mice. **j**–**l** LC-MS/MS quantification of the mRNA m^6^A/A ratio (**j**), FFA content (**k**), and triglyceride (TG) content (**l**) in colorectal tumor tissues from mice in the of WT, *Ahcy* KO, *Fto* KO, and *Ahcy* & *Fto* KO cohorts (*n* = 8–10 mice per group). Data are presented as mean ± S.D., with the exception of tumor volumes, which are presented as mean ± SEM. Two-tailed unpaired Student’s *t*-test (**d**–**f**). One-way ANOVA with LSD-*t* (**a**, **b**, **i**–**l**). Two-way ANOVA with LSD-*t* (**a**). *P < 0.05, **P < 0.01, ***P < 0.001, ****P < 0.0001, N.S., not significant.

Alterations in lipid metabolism are among the most pronounced metabolic changes in cancer cells, and increased lipogenesis contributes to rapid proliferation and tumorigenesis.^49–52^ To further explore whether the effect of AHCY on tumor lipogenesis promotes tumor growth, we assessed tumor growth following re-expression of genes involved in lipogenesis, such as ACACA. Re-expression of the AHCY D245A and Y193H mutants did not counteract the effects of AHCY depletion, including reductions in ACC1 protein expression, lipid droplet abundance, and tumor cell proliferation and tumorigenesis in vivo (Supplementary information, Fig. S6a–c). However, when ACC1 protein expression was restored in AHCY-depleted cells re-expressing the AHCY D245A or Y193H mutant, the reductions in lipid droplet number and tumor growth were partially reversed in vivo (Supplementary information, Fig. S6a–c). To further characterize the role of FTO in lipid metabolism and tumor growth, we observed the effects of AHCY mutants in FTO-depleted tumor cells. Notably, differences in lipid droplet abundance and tumor growth between AHCY WT and the mutants (AHCY Y193H and D245A) nearly disappeared upon FTO depletion (Fig. 6a, b). These results underscore the significance of the FTO/m^6^A signaling pathway for regulation of lipid metabolism and tumor growth by the AHCY–ADO complex.

### The AHCY-FTO axis enhances tumorigenesis in vivo

We sought to determine the role of AHCY in tumor growth and tumorigenesis using an AOM/DSS-induced colorectal cancer model. We engineered mice with conditional knockout of the *Ahcy* gene and crossed them into a pVillin-CreERT2 background to establish a model (*Ahcy* CKO) with conditional knockout of *Ahcy* in colorectal epithelial cells induced via tamoxifen (TAM) treatment (*Ahcy* KO) (Supplementary information, Fig. S6d). Compared with littermate controls treated with corn oil, *Ahcy* CKO mice treated with TAM exhibited a reduced tumor burden (Fig. 6c, d; Supplementary information, Fig. S6e–g), supporting the potential of AHCY as a therapeutic target in colorectal cancers.^28^ As anticipated, immunohistochemical (IHC) staining in the AOM/DSS-induced colorectal cancer model revealed significantly greater AHCY and ACC1 protein levels in tumor tissues than in normal tissues (Supplementary information, Fig. S6h, i). In the colorectal cancer models, we observed significant reductions in ACC1 and Ki67 protein levels in TAM-treated mice compared with corn-oil-treated mice (Supplementary information, Fig. S6j, k).

AHCY plays a pivotal role in one-carbon metabolism, and its metabolic intermediates contribute to homeostasis of several amino acids (cysteine, serine, and methionine).^22^ We observed that TAM-induced KO of the *Ahcy* gene resulted in dysregulation of methionine catabolism and a reduction in FFAs (Fig. 6e; Supplementary information, Fig. S6l), likely reflecting the reduced availability of exogenous lipids accessible to tumor cells in vivo.^53^ This reduction in FFAs may result from downregulation of mRNA m^6^A modification levels (Fig. 6f). Notably, in spontaneous colorectal cancer models, SFAs and MUFAs showed greater downregulation than PUFAs upon *Ahcy* KO (Supplementary information, Fig. S6m). Moreover, *Ahcy* KO significantly reduced the proportional abundance of monounsaturated FFAs (Supplementary information, Fig. S6n). These observations imply that AHCY may play a role in regulating FA biosynthesis and MUFA composition, thereby contributing to tumorigenesis and sustaining tumor growth. To further explore the role of FTO in mediating AHCY-promoted tumorigenesis in mouse orthotopic tumors, we established orthotopic inoculation models of colorectal cancer organoids (Fig. 6g; Supplementary information, Fig. S6o). Consistent with prior findings, the tumor burden was significantly reduced in *Ahcy* KO mice compared with control mice, and *Fto* KO could at least partially reverse the reduction in tumor burden caused by *Ahcy* KO (Fig. 6h, i). Furthermore, *Fto* KO counteracted the reductions in mRNA m^6^A levels and total FFAs and TG caused by *Ahcy* KO (Fig. 6j–l). These results provide further evidence that the AHCY-FTO axis enhances lipid metabolism reprogramming and tumorigenesis in vivo.

### The AHCY–ADO complex is associated with poor prognosis in tumor patients

**Fig. 7:**
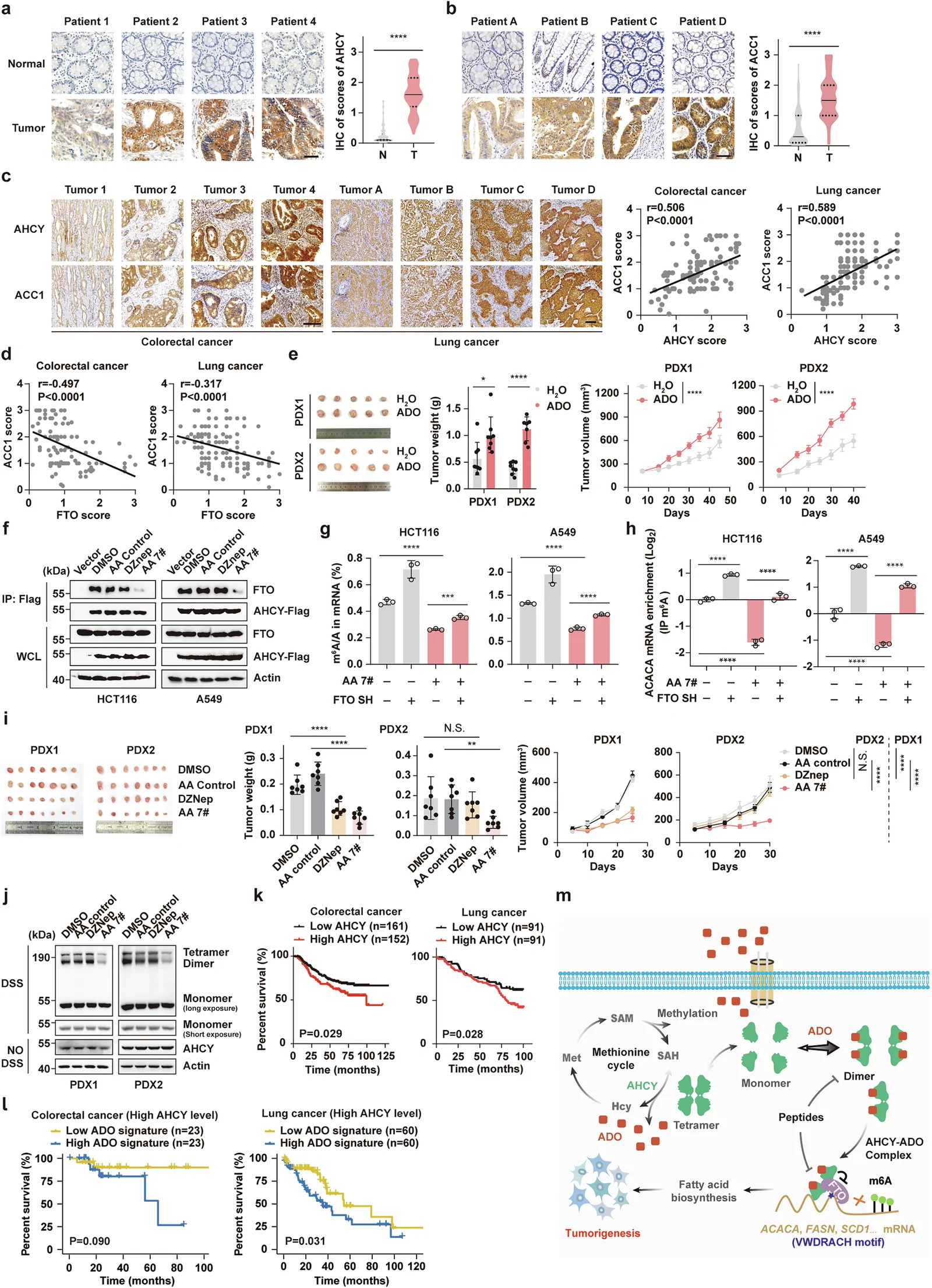
Adenosine and AHCY dimers promote tumor growth, and AHCY correlates with poor prognosis in tumor patients. **a**, **b** Representative IHC staining (left) and statistical analysis (right) of AHCY (**a**) and ACC1 (**b**) protein expression in colorectal cancer tissues and corresponding paracancerous tissues (*n* = 101). The scale bars represent 50 μm. **c** Representative images of IHC staining in four colorectal cancer and lung cancer specimens are shown (left). The scale bars represent 200 μm. Semi-quantitative AHCY and ACC1 scoring of colorectal cancer (*n* = 101) and lung cancer (*n* = 151) tissues was performed (using a scale from 0 to 3) via non-parametric Spearman’s rank correlation test (right). **d** Semi-quantitative FTO and ACC1 scoring of colorectal cancer (*n* = 101) and lung cancer (*n* = 149) tissues was performed (using a scale from 0 to 3) via non-parametric Spearman’s rank correlation test. **e** Representative images, weights, and volumes of PDX tumors derived from colorectal cancer patients treated with H_2_O or 5 mg/kg ADO three times per week (*n* = 6–7). **f** Western blot analysis of WCLs and anti-Flag immunoprecipitates from HCT116 and A549 cells expressing FTO and Flag-AHCY and treated with DMSO, 200 μM control peptide (AA control), 25 μM DZNep, or 100 μM of an AHCY-dimer-perturbing peptide (AA #7) for 24 h. **g**, **h** LC-MS/MS quantification of the mRNA m^6^A/A ratio (**g**) and MeRIP-qPCR analysis of *ACACA* mRNA (**h**) were performed in HCT116 and A549 cells with or without FTO shRNA transduction treated with 100 μM of an AHCY-dimer-perturbing peptide (AA #7) for 24 h. **i** Representative images, weights, and volumes of colorectal cancer PDX tumors treated with DMSO (twice weekly), the control peptide (15 mg/kg, daily), DZNep (2.5 mg/kg, twice weekly), or AA #7 (15 mg/kg, daily) for 30 days (*n* = 6–7). **j** Analysis of the oligomerization state of endogenous AHCY in the PDX1 and PDX2 models following treatment with the perturbing peptide AA #7, using disuccinimidyl suberate for protein cross-linking. **k** Kaplan–Meier overall survival curves for colorectal and lung cancer patients in the local cohort stratified by tumor AHCY protein level (high vs low). **l** Kaplan–Meier overall survival curves for colorectal cancer and lung cancer patients with high AHCY levels in the TCGA tissue cohort stratified by the ADO gene signature. **m** Schematic model showing that the AHCY–ADO complex drives AHCY dimerization to inhibit FTO activity and increase mRNA m^6^A levels, ultimately promoting FA synthesis and tumorigenesis. Data are presented as mean ± S.D., with the exception of tumor volumes, which are presented as mean ± SEM. Two-tailed unpaired Student’s *t*-test (**a**, **b**, **e**). One-way ANOVA with LSD-*t* (**g**–**i**). Two-way ANOVA with LSD-*t* (**e**, **i**). *P* values were obtained by the log-rank *t*-test (**k**, **l**). *P < 0.05, **P < 0.01, ***P < 0.001, ****P < 0.0001, N.S., not significant.

IHC analysis was performed on primary colorectal cancer tissues and adjacent normal tissues, as well as primary lung cancer tissues. This analysis consistently revealed an upregulation of both AHCY and ACC1 in colorectal cancer tissues compared with their adjacent normal counterparts (Fig. 7a, b) and a positive correlation between the expression levels of these markers in resected lung and colorectal cancer tissues (Fig. 7c). Conversely, FTO expression was reduced in colorectal and lung cancer samples compared with adjacent normal tissues (Supplementary information, Fig. S7a, b). Furthermore, FTO and ACC1 expressions were negatively correlated in surgically resected lung and colorectal cancer tissues (Fig. 7d). To examine the effect of ADO on human tumor progression, we established colorectal cancer patient-derived xenograft (PDX) models in immunodeficient mice via subcutaneous implantation of primary tumor fragments with high levels of AHCY protein expression obtained from untreated patients, ensuring preservation of the original tissue architecture (Supplementary information, Fig. S7c, d). Notably, ADO significantly increased ACC1 and Ki67 levels and tumor growth in the colorectal cancer PDX models (Fig. 7e; Supplementary information, Fig. S7e, f). To further confirm the role of increased AHCY dimerization mediated by the AHCY–ADO complex in promoting human tumor growth, we specifically designed a series of peptides to disrupt AHCY dimers (Supplementary information, Table S4). Harnessing insights from protein dimerization dynamics, we identified two peptides of interest (AA #6 and AA #7). Peptide AA #7 demonstrated the greatest potential for disrupting AHCY dimerization, yet AHCY inhibitors failed to affect these dimers (Supplementary information, Fig. S7g, h). Remarkably, peptide AA #7 also successfully disrupted the interaction between FTO and AHCY that was induced by the AHCY–ADO complex (Fig. 7f) and inhibited cell growth in cancer cell lines (Supplementary information, Fig. S7i). In addition, treatment with AA #7 significantly reduced both mRNA m^6^A levels and the m^6^A modification levels of mRNAs associated with lipogenesis genes (*ACACA* and *SCD1*) (Fig. 7g, h; Supplementary information, Fig. S7j). Most importantly, compared with the control peptide, peptide AA #7 significantly suppressed AHCY dimerization and tumor growth (Fig. 7i, j), suggesting that targeting AHCY dimers could be a promising strategy for cancer treatment.

To further characterize the relationship between the anti-tumor action of peptide AA #7 and the FTO/m^6^A signaling pathway, we evaluated the behavior of FTO-depleted cell lines treated with AA #7. The ability of AA #7 to inhibit tumor cell growth and lipid droplet abundance was markedly lower in FTO-depleted lines than in control colorectal and lung cancer cell lines (Supplementary information, Fig. S7k, l). Similarly, FTO depletion also at least partially reversed the inhibitory effects of AA #7 on mRNA m^6^A levels and m^6^A modification levels of lipogenesis-related genes (*ACACA* and *SCD1*) (Fig. 7g, h; Supplementary information, Fig. S7j). However, FTO depletion did not reverse the downregulation of histone methylation induced by AA #7 (Supplementary information, Fig. S7m). Therefore, these results suggest that the FTO/m^6^A signaling pathway plays a critical role in mediating the anti-tumor effects of AA #7.

We next examined the survival times of patients with lung cancer and colorectal cancer and found that high AHCY levels were correlated with significantly shorter median survival times (Fig. 7k). Similarly, analyses of TCGA data across various cancers revealed that high AHCY expression was associated with reduced overall survival in patients with low-grade glioma (LGG), liver cancer (LIHC), and lung adenocarcinoma (LUAD) (Supplementary information, Fig. S7n). By contrast, low FTO levels were correlated with significantly shorter median survival times in colorectal and lung cancers (Supplementary information, Fig. S7o). These results indicate that AHCY-mediated biological events influence the clinical aggressiveness of human tumors, particularly colorectal and lung tumors. Intriguingly, high expression of genes in the ADO-related gene signature was associated with a greater reduction in overall survival in colorectal cancer, lung cancer, LGG, and skin cutaneous melanoma (SKCM) patients with high AHCY expression levels, but this correlation did not extend to patients with low AHCY expression levels (Fig. 7l; Supplementary information, Fig. S7p, q), highlighting the critical effect of the AHCY–ADO complex on tumor progression and clinical aggressiveness.

## Discussion

Tumor cells possess the potential for unlimited replication and rapid growth, with metabolic reprogramming playing an integral part in this cascade.^54,55^ The methionine cycle has a vital role in the metabolic reprogramming of tumor cells, influencing tumor growth and proliferation.^5,16^ However, the regulatory mechanisms by which the methionine cycle contributes to these processes remain largely unclear. AHCY and ADO are a crucial enzyme and a metabolite, respectively, in the methionine cycle. Here, we report the unexpected intrinsic and broad cancer-promoting activity of the AHCY–ADO complex. Mechanistically, this complex initiates AHCY dimerization, which disrupts binding of the demethylase FTO to VWDRACH motifs in mRNA and reduces FTO activity by enhancing the interaction between AHCY and FTO. Consequently, genes involved in lipid metabolism that promote lipogenesis and tumor cell growth are upregulated (Fig. 7m).

The methyl donor SAM is derived from methionine, and investigations into methionine metabolism and its impact on epigenetic processes have become important in epigenetics research.^3,5^ Our studies revealed a SAM-independent regulatory mechanism of the methionine pathway in which the AHCY–ADO complex regulates mRNA m^6^A levels by inhibiting FTO activity, further emphasizing the importance of methionine metabolism in epigenetic regulation. Although the high affinity of the AHCY–ADO complex has been noted previously,^36^ the function of this complex has been largely overlooked. Using an AHCY-based ADO sensor, we dynamically observed its presence and confirmed its crucial role in regulating mRNA m^6^A levels through chemically induced proximity systems that do not affect the overall ADO content.

In addition to its involvement in fundamental biological processes, FTO demonstrates significant yet paradoxical roles in cancer biology,^7,14,15,56^ exhibiting both tumor-promoting and tumor-suppressing functions. Notably, there have been conflicting reports on FTO’s carcinogenic vs tumor-suppressive activities, even within the same cancer type, including colon cancer^57,58^ and lung cancer.^59,60^ This apparent contradiction may arise from heterogeneity in molecular expression among different tissue origins and cancer subtypes, as well as the diversity of FTO substrates and alterations in metabolic regulation or the tumor microenvironment. Our study revealed that the AHCY–ADO complex inhibits FTO activity, thereby increasing the m^6^A levels of lipogenesis genes such as *ACACA* and *SCD1* and promoting tumor growth. We found that AHCY dimerization mediated by the AHCY–ADO complex does not globally influence the binding of FTO to its substrates but may instead have a preferential effect on binding to specific substrate sequences, such as RNA containing the VWDRACH motif, which preferentially binds to the FTO Q86 site. Crucially, because the efficiency of AHCY-mediated lipogenesis depends on the availability of exogenous lipids, the degree of external lipid utilization may critically modulate the tumor-suppressive functions of FTO in AHCY-overexpressing cancers (e.g., lung and colorectal cancer). These findings emphasize the necessity for comprehensive evaluation of tumor metabolic dynamics and microenvironmental interactions when developing FTO-targeted therapies.

Metabolic products include small-molecule substrates, intermediate products, and end products within metabolic pathways,^3^ and the interactions of these factors with proteins convey metabolic-state information to various cellular processes.^15,20,61^ Metabolite binding plays a pivotal role in regulating the assembly and functioning of protein complexes and high-molecular-weight protein assemblies.^20^ Most research on AHCY has focused predominantly on its enzymatic activity, revealing that it exhibits the highest activity in the tetrameric state.^17,22,28,30,33,34^ The content of AHCY dimers surpasses that of tetramers under physiological conditions, but the functional role of AHCY dimers remains unclear. Our results indicate that the AHCY–ADO complex promotes and stabilizes AHCY dimers, which lack hydrolase activity, but still enhance ADO signaling beyond mere substrate binding. Activation of methionine metabolism in tumor cells via the AHCY dimer signal mediated by ADO stimulates FA biosynthesis and alters intracellular FFA homeostasis, primarily by altering endogenous FA synthesis and MUFA composition.

To better distinguish the contributions of the moonlighting functions of AHCY, we used the Y193H and D245A AHCY mutants, which retain enzymatic activity without affecting SAM levels. Under these conditions, the AHCY–ADO-complex-FTO axis was found to upregulate FFAs independently of the precursor substrate acetyl-CoA. Furthermore, the AHCY–ADO complex, in association with the FTO axis, not only regulates the FA biosynthesis gene *ACACA* but also enhances the activity of SCD1, thereby increasing the proportion of MUFAs. This process may involve the desaturation of newly synthesized or internalized SFAs to prevent lipotoxicity and cell death induced by endoplasmic reticulum stress during rapid tumor proliferation.^48,51^ FA synthesis is implicated in cancer and various physiological and pathological processes, including embryonic development.^46,62^ Mice that lack the key enzymes Acc1 or Fasn experience early embryonic death. Our study offers a possible explanation for why AHCY deficiency, but not that of other methionine metabolism enzymes (e.g., Mat1a, Mat2a, Gnmt, Cbs, Bhmt), leads to embryonic mortality in mice.^22,63^ These findings further imply the potential importance of the nonenzymatic form of AHCY in pathological and physiological processes.

We obtained genetic evidence that AHCY acts as a bona fide metabolic oncogenic activator in colorectal cancers. Using in situ tumor models in genetically modified mice, we observed a significant reduction in the burden of colorectal tumors resulting from *Ahcy* deletion. These observations are consistent with previous studies,^28^ which indicated that pharmacological inhibition of Ahcy reduced the intestinal tumor burden in *Apc*^*Min/+*^ mice. Impeding AHCY activity, leading to blockade of methionine catabolic processes and nucleotide metabolism, prevents the self-renewal and differentiation of crypt progenitor and cancerous stem cells, events driven by *Apc* loss that are believed to be precursors to cancer.^28^ However, the inhibition of noncanonical AHCY activities, such as those involving activation of the lipid metabolic pathway initiated by AHCY–ADO-complex-induced AHCY dimers, affects the malignant progression and proliferation of cancer cells. In orthotopic colorectal cancer organoid models, *Fto* KO partially reversed the *Ahcy* KO-induced reductions in total FFAs, TG, and tumor burden, suggesting that the AHCY-FTO axis promotes reprogramming of lipid metabolism and tumorigenesis in vivo. Notably, peptide AA #7, which we developed on the basis of AHCY dimer formation, effectively disrupted the dimerization of AHCY and inhibited the proliferation of diverse tumor cell types in vitro and in vivo. These findings support the observation that high expression of ADO-synthesis signature genes is associated with reduced overall survival in patients with colorectal and lung cancers with high AHCY expression, highlighting the critical role of the AHCY–ADO complex in tumor progression and clinical outcomes in cancer patients and underscoring the therapeutic potential of targeting AHCY dimers.

## Materials and methods

### Cell culture

HEK293T human embryonic kidney cells, the human lung cancer cell lines A549 and H1299, the human liver cancer cell line HepG2, and the human breast cancer cell line MCF7 were cultured in DMEM supplemented with 10% fetal bovine serum (FBS) and 1% penicillin–streptomycin (Gibco, 15140163). The human colorectal cancer cell lines HCT116 and SW480, NCM460 colonic epithelial cells, and the human esophageal cancer cell line TE11 were cultured in RPMI-1640 medium supplemented with 10% FBS and 1% penicillin–streptomycin. Mouse embryonic fibroblasts (MEFs) were cultured in MEM supplemented with 10% FBS, 1% penicillin–streptomycin, and growth factors. HEK293T, NCI-H1299, A549, SW480, HCT116, HepG2, MCF7, and MEF cell lines were obtained from the American Type Culture Collection. The TE11 cell line was a kind gift from Dr. Hiroshi Nakagawa and Dr. Anil K. Rustgi at the Columbia University Irving Medical Center, USA. All cells were maintained at 37 °C in a 5% CO_2_ humidified incubator. Cells were authenticated by analysis of short tandem repeats, and mycoplasma tests were performed bi-weekly throughout the experiments. AHCY KO cell clones were generated by CRISPR-Cas9 gene editing. In brief, lentiviruses carrying LentiCRISPRv2 (Addgene, 52961) constructs with guide sequences (KO1, GAT GAT GTC AAT ACA GCC TG; KO2, GCG GGC TCC GAA ACC CCG CA) were used to infect HEK293T and SW480 cells. Virus infection was performed using 8 μg/mL polybrene for 24 h, followed by medium replenishment. Antibiotics were used to select the virus-infected cells, and single clones were propagated for subsequent experiments.

### Construction of relevant plasmids and lentivirus

Protein expression plasmids were generated by PCR amplification of the respective cDNA open reading frames. Primers were designed to include protein tag sequences (such as Flag, Myc, or HA tags) and were recombined into double-enzyme-digested vectors following the instructions of the ClonExpress Ultra One Step Cloning Kit (Vazyme, C115). AHCY mutants, ADA mutants, and FTO mutants were generated using PCR-based site-directed mutagenesis and DpnI digestion (NEB, R0176). The relevant primer sequences are provided in Supplementary Information, Table S5. shRNAs targeting were designed using the website https://portals.broadinstitute.org/gpp/public/gene/search and cloned into the pLKO.1 plasmid (Addgene, 10878); the relevant primer sequences are provided in Supplementary information, Table S6. To circumvent shRNA-mediated degradation of exogenously expressed cDNAs, we introduced synonymous mutations to the shRNA targeting sequence of expression constructs. Lentiviral particles were produced in HEK293T cells using a third-generation packaging system. This system involved the use of pLKO.1 or pCDH plasmids, as well as psPAX2 (Addgene, 12260) and pCMV-VSV-G (Addgene, 8454) plasmids. The supernatant of transfected HEK293T cells was harvested ~48–72 h later and filtered through a 0.45 μm pore size to obtain viral stock. The stock was kept at 4 °C for short-term use (1–5 days) or −80 °C for long-term storage.

### CRISPR/Cas9 metabolic enzyme knockout screening

Two types of circRNA incorporating split GFP together with an m^6^A motif were obtained as described previously.^25,26^ The CRISPR-Cas9 knockout library contained 7142 sgRNAs targeting genes encoding 1773 classical metabolic enzymes, with four sgRNAs designed per gene. The m^6^A GFP reporter gene plasmid was packaged into lentivirus, and the obtained virus was used to infect HEK293T cells. After 48 h, puromycin selection was performed. To establish a homogeneous fluorescent cell line for subsequent experiments, single clones were selected. CRISPR-Cas9-mediated knockout of metabolic enzyme-related genes was performed in HEK293T single-clone cells infected with the lentiviral library. The transfection efficiency was maintained at around 30%. Seven days after viral addition, flow cytometry was performed after puromycin selection to isolate cells that exhibited the weakest fluorescence intensity (5%). Genomic DNA was extracted from these cells, libraries were constructed, and sequencing analysis was performed. The sgRNA read count and hit calling were analyzed using MAGeCK v0.5.6. The MAGeCK scores and lists of ranked genes for CRISPR-mediated knockout screens are provided in Supplementary information, Tables S1 and S2. The raw sequencing data have been deposited in the BioProject database under the ID PRJNA1077874.

### Quantitative assessment of m^6^A levels in mRNA using LC-MS/MS

Total RNA was extracted using the AxyPrep Multisource Total RNA Miniprep Kit (AXYGEN, AP-MN-MS-RNA-250), and mRNA was enriched using the Hieff NGS mRNA Isolation Master Kit (YEASEN, 12603ES96). The enriched mRNA was digested with RNase enzyme P1 (NEB, M0660S) to break down RNA from single-stranded to single-nucleotide form. This process involved incubation at 37 °C for 1 h, followed by inactivation at 75 °C for 10 min. Alkaline phosphatase (Beyotime, D7027) and sodium bicarbonate were then added, followed by another incubation at 37 °C for 1 h and inactivation at 75 °C for 10 min. The solution was centrifuged, degassed by ultrasound in a mobile phase, and injected into a liquid chromatography instrument, proceeding to tandem mass spectrometry analysis (triple quadrupole liquid chromatograph, Agilent 1290 Infinity; mass spectrometer, Agilent 6495). Single ribonucleotides were ionized, and precursor/fragment ion pairs were derived from nucleoside molecular information. In the mass spectra, the precursor/fragment ion pair for m^6^A was identified as 282.1 Da/150.1 Da. The peak area for m^6^A was calculated on the basis of retention time. Finally, the overall methylation intensity of m^6^A on mRNA was determined by calculating the proportion of m^6^A relative to total adenosine.

### Extraction and detection of target metabolites

Metabolite extraction procedures varied between tissue and cell samples. In brief, tissue samples weighing ~50–100 mg were cut into small pieces and placed in a grinding tube. Metabolites were extracted using 1.2 mL of low-temperature HPLC-grade methanol and water in a 1:1 volume ratio. Each sample was homogenized using a tissue crusher (Xinzhi Biotechnology Scientz-IID) and centrifuged at 14,000 rpm for 10 min at 4 °C; 960 μL of the supernatant was transferred to a new EP tube. The sample was then vacuum dried and dissolved in 200 μL of pre-cooled methanol, acetonitrile, and water in a 5:3:2 volume ratio for detection of hydrophilic metabolites related to methionine circulation. Methionine-related metabolites were analyzed using LC-MS (Agilent 1290 Infinity liquid chromatography system and Agilent 6495 mass spectrometer). Standard curves were constructed using known concentrations of methionine (Sigma, M9625), S-adenosylmethionine (MACKLIN, S997346), *S*-adenosylhomocysteine (MCE, HY-19528), adenosine (Sigma-Aldrich, V900417), and homocysteine (MCE, HY-19528) to determine metabolite contents in the samples. Data were normalized to protein content as determined with a Bradford protein assay kit (Beyotime, P0006C). The precipitate from the previous centrifugation step was used for extraction of hydrophobic metabolites such as lipids. Lipid metabolites were extracted using 1.28 mL of pre-cooled HPLC-grade methanol and chloroform in a 1:3 volume ratio. After homogenization with a tissue crusher, each sample was centrifuged at 14,000 rpm for 10 min at 4 °C, and 900 μL of the chloroform layer was transferred to a new EP tube. Nitrogen was blown in a fume hood for subsequent lipid detection using GC-MS (Shimadzu GCMS-QP2020 NX gas chromatography-mass spectrometry system).

### Measurement of total free fatty acids, triglycerides, acetyl-CoA, and malonyl-CoA

In brief, cells were digested, washed once with PBS, and lysed in the reagent (5 × 10^5^ cells per 100 μL) using ultrasound (200 W, 2 s on, 3 s off for a total of 10 min). The lysates were then centrifuged at 12,000× *g* and 4 °C for 20 min. Culture medium and serum were directly used for testing. Frozen tissues were ground in reagent (10 mL/g tissue).

Free fatty acids were quantified using the Amplex Red Free Fatty Acids Assay Kit (Beyotime S0215S). Triglycerides were measured using the Amplex Red Triglyceride Assay Kit (Beyotime, S0219M). Following the manufacturer’s instructions, direct testing was performed for culture medium and serum triglycerides. For tissue samples, 100 μL isopropanol was added per 10 mg tissue, and the mixture was homogenized at 4 °C. A 20 μL volume of lysate supernatant was transferred to a 96-well plate, and assay buffer or buffer with isopropanol was added to 50 μL. Blank controls included only assay buffer or buffer with isopropanol. Absorbance was read at 570 nm using a SpectraMax M microplate reader. The corresponding protein concentration or tissue weight was used for normalization.

Cells were collected into centrifuge tubes, washed once with PBS, and dissolved in extraction solution (5 × 10^5^ cells per 100 μL). After sonication for 30 min on ice, the extraction solution was centrifuged at 12,000× *g* for 20 min, and the supernatant was collected and placed on ice for testing. Acetyl-CoA levels were measured using an Acetyl-Coenzyme A ELISA Research Kit (MB 3258 A) according to the manufacturer’s protocol. Absorbance was measured at 450 nm using a SpectraMax M microplate reader. Malonyl-CoA was detected using a human malonyl-CoA ELISA kit (Maisha MS1933-A), and absorbance was measured at 450 nm after a series of incubation and reaction steps. The corresponding protein concentration was used for normalization.

### Separation of AHCY tetramers, dimers, and monomers

Purified GST-AHCY protein was digested with HRV 3 C protease (TAKARA, 7360) at 4 °C for 16 h, and the GST tag was removed using glutathione MagBeads (GenScript, L00895-10). AHCY monomers, dimers, and tetramers were separated using AKTA Purify (Cytiva) with pre-chilled buffers (20 mM HEPES pH 8.0, 150 mM NaCl). The gel filtration column used was a SEPHACRYL S-200 HR, T 10×300 GL (Cytiva, 28956916). After the column was installed, the system was washed with loading buffer (20 mM HEPES pH 8.0, 150 mM NaCl). Then, 100 µL of the prepared 20 µM AHCY protein, with or without adenosine, was loaded into the chromatography system by syringe. The program was set as follows: flow rate 0.5 mL/min, pressure 2.5 MPa, accumulation volume 24 mL. Elution of the sample was monitored using an ultraviolet (UV = 280 nm) detector. The eluted AHCY tetramer, dimer, and monomer were further identified by non-denaturing gel electrophoresis (RTD, 61380416) for use in subsequent experiments.

### Western blotting assay

Cells were washed three times with pre-chilled PBS and then lysed on ice for 30 min with RIPA lysis buffer containing a protease inhibitor cocktail (MCE, HY-K0010). The lysates were centrifuged at 12,000× *g* for 30 min, and the supernatant was collected for protein quantification using a Bradford protein assay kit (Beyotime, P0006C). The samples were mixed with 2× loading buffer and incubated at 95 °C for 5–10 min for SDS-PAGE. The proteins in the gel were transferred onto a PVDF membrane (Bio-Rad, 1620177), which was then blocked with 5% skimmed milk at room temperature for 1 h. The membrane was incubated with the primary antibody overnight in TBST at 4 °C. After three washes with TBST, the membrane was incubated with an HRP-conjugated anti-rabbit (CST, 7074 V, 1:2000) or anti-mouse (CST, 7076 V, 1:2000) IgG secondary antibody at room temperature for 1 h, followed by detection using a chemiluminescence imaging system (Bio-Rad). The antibodies used in this study were anti-AHCY (Proteintech, 10757-2-AP, 1:1000), anti-MAT2A (CST, 84478, 1:1000), anti-Histone H3 (CST, 4499, 1:2000), anti-H3K4me3 (CST, 9751, 1:2000), anti-H3K9me3 (CST, 13969, 1:2000), anti-H3K27me3 (CST, 9733, 1:2000), anti-H3K36me3 (CST, 4909, 1:2000), anti-H3K79me3 (Abways, AB3548, 1:1000), anti-FTO (CST, 45980, 1:1000), anti-actin (CST, 93473, 1:1000), anti-GAPDH (CST, 92310, 1:1000), anti-PARP (CST, 9542, 1:1000), anti-flag (Sigma, F3165, 1:1000), anti-HA (CST, 2367, 1:1000), anti-Myc (CST, 5605, 1:1000), anti-SAHH (Abcam, ab134966, 1:1000), anti-ACC1 (Proteintech, 21923-1-AP, 1:1000), anti-GST (Proteintech, 66001-2-Ig, 1:2000), FASN (Acmec, AC50671, 1:1000), anti-SCD1 (Proteintech, 28678-1-AP, 1:1000), anti-SREBP1 (Santa Cruz, sc-13551, 1:1000), SREBP2 (Santa Cruz, sc-13552, 1:1000), anti-p-AMPK (CST, 9957, 1:1000), anti-AMPK (CST, 9957, 1:1000), anti-p-PI3K (GeneTex, GTX100462S, 1:1000), anti-Akt (CST, 927, 1:10002), anti-EGFR (Proteintech, 66455-1-Ig, 1:1000), anti-Ki67 (Proteintech, 27309-1-AP, 1:1000), anti-ALKBH5 (Proteintech, 16837-1-AP, 1:1000), anti-METTL3 (Proteintech, 15073-1-AP, 1:1000), and anti-METTL14 (Proteintech, 26158-1-AP, 1:1000).

### Co-immunoprecipitation

IP lysis buffer (20 mM Tris-HCl pH 7.4, 1% NP-40, 10% glycerol, 137 mM NaCl, 2 mM EDTA) was supplemented with 1 mM dithiothreitol (DTT), 1 mM phenylmethylsulfonyl fluoride (PMSF) (Beyotime, ST506), and a protease inhibitor cocktail (MCE, HY-K0010). Cells were lysed on ice for 30 min, and the supernatant was collected after centrifugation. A portion of the supernatant was used as the input control. A 500 μL volume of the supernatant was immunoprecipitated with the corresponding antibody and then added to protein A/G magnetic beads (MCE, HY-K0202), or with anti-HA magnetic beads (MCE, HY-K0201) and anti-Flag magnetic beads (MCE, HY-K0207). The immunoprecipitates were washed four times with IP lysis buffer and eluted with 50 μL of 2× loading buffer, then heated at 98 °C for 10 min. Immunoblotting was performed together with the input control sample.

### Quantitative RT-PCR (qPCR)

Total RNA was extracted using an RNA extraction kit (AXYGEN, AP-MN-MS-RNA-250), and RNA concentration was quantified using a Nanodrop UV spectrophotometer. A total of 1000 ng of RNA was used for reverse transcription with a reverse transcription kit (Takara, RR036A). Real-time quantitative PCR was performed using the TransGen Biotech AQ101 kit and a Bio-Rad CFX 96 fluorescence quantitative PCR instrument. GAPDH or 18S was used as the reference gene for normalization, and primer sequences were obtained from PrimerBank (https://pga.mgh.harvard.edu/primerbank) as shown in Supplementary information, Table S7.

### Proximity ligation assay (PLA) and confocal microscopy

The interaction between AHCY and FTO in cells was detected by PLA with Duolink In Situ Orange Starter Kit Mouse/Rabbit (Sigma, DUO92102-1KT) according to the manufacturer’s instructions. In brief, cells were fixed in 4% paraformaldehyde at room temperature and permeabilized with 0.5% Triton X-100. After blocking, cells were incubated with primary antibodies (anti-AHCY, Proteintech, 10757, 1:50; anti-FTO, Abcam, ab92821, 1:50) overnight at 4 °C, then with PLUS (Sigma, DUO92001) and MINUS (Sigma, DUO92005) PLA probes for 1 h at 37 °C. Cells were then sequentially incubated in ligation solution for 30 min and amplification solution for 100 min, both at 37 °C. After nuclear staining with DAPI, PLA samples were observed under a confocal microscope (Nikon, CSU-W1). For the sfGFP(1–10) and sfGFP11 system, cells co-expressing AHCY-sfGFP(1–10) and FTO-sfGFP11 were seeded at a density of 5 × 10^4^ cells in a confocal dish and cultured for 48 h. The nuclei were stained with Hoechst 33342 live-cell staining solution (Beyotime, C1028, 1:200). Imaging was performed using a high-resolution laser confocal microscope (Zeiss, LSM 880) to study the co-localization of AHCY and FTO.

### Confocal imaging of adenosine sensor

Cells expressing an AHCY-based adenosine sensor were examined by confocal imaging (Supplementary information, Data S1). For the ABA-triggered ABI/PLY1 system, cells expressed ADA-PLY1 and Sensor C1-ABI fusion proteins (Supplementary information, Data S2). Cells were first bathed in Tyrode’s solution (150 mM NaCl, 4 mM KCl, 2 mM MgCl_2_, 2 mM CaCl_2_, 10 mM HEPES pH 7.3–7.4) and 10 mM glucose and imaged before and after addition of 100 μM adenosine or 3 mM ABA. Live cells expressing the AHCY-based adenosine sensor were screened and imaged using a spinning disk confocal microscope (Nikon, CSU-W1). To image GFP fluorescence, we used a 475/30 nm laser for excitation and a 535/50 nm emission filter to collect the signals.

### AHCY activity assay

About 1 μg of protein, either directly purified or extracted from cells stably expressing AHCY or mutants, was used for the in vitro enzyme activity assay, which was performed according to the instructions of the Adenosylhomocysteinase (AHCY) Activity Assay Kit using the fluorescence method (Abcam, ab197002). The fluorescence intensity was measured using excitation and emission wavelengths of 535 nm and 587 nm with a multimode plate reader (Victor Nivo 5S) at 37 °C for 30 min per well. AHCY enzyme activity was calculated from a standard curve using the enzyme reaction kinetics formula.

### Recombinant protein purification

Human FTO was subcloned into the pET28a vector (Novagen) to generate a 6× His-tagged fusion protein. Human AHCY and its mutants were subcloned into the pGEX-GST vector. Both FTO and AHCY protein plasmids were transformed into BL21-DE3 chemically competent cells (TransGen, CD701-03). Cultures were shaken at 37 °C in liquid LB medium supplemented with the corresponding antibiotics until the OD600 reached 0.6–1.0. IPTG (Sangon Biotech, A600168) was added to a final concentration of 0.1–0.5 mM, and the cultures were shaken at 20 °C and 200 rpm for 22 h. All cultures were harvested and centrifuged at 8000 rpm and 4 °C for 10 min to obtain bacterial pellets. The pellets were washed with PBS and lysed in lysis buffer (50 mM Tris-HCl, 300 mM NaCl, 1 mM PMSF, and 1 mM protease inhibitor cocktail) before sonication. GST-tagged proteins were purified using GST agarose purification resin (Sangon Biotech, C600913), and His-tagged proteins were purified using the 6× His-Tagged Protein Purification Kit (Cwbio, CW0893).

### Surface plasmon resonance (SPR)

AHCY protein and its mutant variants (~15 μg) purified from prokaryotes were immobilized on a Series S Sensor chip CM7 (Cytiva, 29147020). The chips were placed in the Biacore T200 instrument to wash away non-specifically bound substances using a flowing buffer solution. A gradient-diluted solution of adenosine (Sigma-Aldrich, V900417) was injected simultaneously. Resonance curve analysis was performed to evaluate the affinity, binding constants, and other related parameters between adenosine and AHCY or its mutant variants.

### GST pull-down assay

The GST-tagged AHCY fusion protein and its mutant variants, as well as 6× His-tagged FTO, were purified after prokaryotic expression. Glutathione MagBeads (GenScript, L00895-10) were washed three times with lysis buffer, and 500 μL of the same buffer containing 2 μg AHCY and 2 μg FTO proteins was added. The quantity of proteins was increased when the concentration of adenosine (Sigma-Aldrich, V900417) was elevated. The mixture was rotated at 4 °C for 4 h. The beads were washed three times with lysis buffer, the bound proteins were eluted with 50 μL 2× loading buffer at 98 °C for 10 min, and the supernatant, which contained the target proteins, was collected. Samples were then subjected to SDS-PAGE or stored at −80 °C for future use.

### Separation of cytoplasmic and nuclear proteins

Cytoplasmic and nuclear proteins were separated using the cytoplasmic and nuclear extraction reagent kit (Beyotime, P0028). In brief, after the cells were washed with PBS, 200 μL of cell lysis protein extraction reagent A supplemented with PMSF was added to 20 μL of cell pellet and incubated on ice for 10 min. Then, 10 μL of cell lysis protein extraction reagent B was added and incubated on ice for 1 min. The mixture was centrifuged at 4 °C and 12,000 × *g* for 5 min, and the supernatant, which contained cytoplasmic proteins, was collected. The pellet was washed with PBS, and 50 μL of cell nucleus protein extraction reagent with PMSF was added. The mixture was incubated on ice for 30 min, then centrifuged at 4 °C and 12,000 × *g* for 10 min. The supernatant contained the extracted nuclear proteins. The isolated cytoplasmic and nuclear proteins were used for subsequent immunoprecipitation experiments.

### MeRIP-seq and analysis

Total RNA was extracted according to the instructions of the AXYGEN AP-MN-MS-RNA-250 kit, and samples with concentration > 50 ng/μL, RIN value > 7.0, and OD260/280 > 1.8 were used for downstream experiments. mRNA was specifically captured and purified twice using oligo(dT) magnetic beads, then fragmented using a magnesium ion fragmentation reagent kit (NEB, E6150S). Fragmented mRNA was pre-mixed with m^6^A-immunomagnetic beads, and the IP product was reverse transcribed into stable cDNA. The fragments were screened and purified through magnetic beads, and a sequencing library was constructed by PCR and sequenced (LC-Bio). Low-quality data were filtered out, and the mapping reads were analyzed and counted using HISAT2 software, mainly counting the distribution of uniquely mapped reads. ExomePeak2 was used for differential peak analysis. Peaks with diff.log_2_FC > 0 and fdr < 0.01 were considered upregulated, and peaks with diff.log_2_FC < 0 and fdr < 0.01 were considered downregulated. Motif analysis was performed on the differential peaks using the findMotifsGenome.pl function of HOMER with the parameters -size 200 and -len 5,6,7. The raw sequencing data have been deposited in the BioProject database with the ID PRJNA1079664.

For the selection of publicly available data, we performed independent cohort analyses rather than a combined analysis owing to the inherent technical and biological variation among diverse sequencing datasets (e.g., platform-specific biases, batch effects, and inter-individual heterogeneity). Cohorts were prioritized on the basis of the availability of paired MeRIP-seq and RNA-seq data and a minimum of 10 tumor samples. Data from GSE190388 and GSE119168 were downloaded from the GEO database via prefetch, subjected to quality control with FastQC, and aligned using HISAT2 to the human reference genome (GRCh38) with annotations from Homo_sapiens.GRCh38.84.gtf (Ensembl). Data were converted to the required formats using Samtools, and MACS2 was used for peak calling. The obtained narrow peaks were merged using bedtools and then intersected with the hg38 annotated genome using bedtools intersect to map peaks to annotated genes and to integrate different samples. Coverage was calculated using bedtools multicov for the IP product and input. The ratio between the IP RPKM value and the input RPKM value for each m^6^A RNA coverage in each tissue sample was considered to be the m^6^A modification level. Peaks of maximum coverage value for the same gene and the same region were retained for further analysis. Protein-coding genes were filtered, and input RPKM values were treated as mRNA expression levels. The median level of m^6^A was considered to be the overall sample m^6^A level. Correlation coefficients between AHCY mRNA expression and overall sample m^6^A level were calculated for each dataset. Cysteine and methionine metabolism pathways were used for calculation of the correlation between expression levels of enzymes and overall sample m^6^A levels.

### Analysis of AHCY–adenosine complex scores

The AHCY–ADO complex level was calculated using the normalized AHCY level and normalized adenosine level. In brief, we first subtracted the minimum AHCY expression value in each cohort, then took the logarithm and normalized it to a value between 0 and 1 (formulas 1 and 2). Intracellular ADO is primarily derived from three mechanisms^21^: (1) transport from the microenvironment into tumor cells via the SLC39A1 and SLC28A1 transporters; (2) hydrolysis of SAH in the methionine cycle catalyzed by AHCY; and (3) the nucleotide metabolism pathway, which involves genes such as NT5C1A, ADA, CECR1, and ADK. SLC39A1, SLC28A1, AHCY, and NT5C1A contribute to increased intracellular ADO levels, whereas ADA, CECR1, and ADK are associated with reducing ADO levels. Building on previously established methodologies for assessing metabolic pathways,^19,64^ including urea cycle disorders and ammonia levels, we developed an ADO signature score to quantify intracellular ADO levels on the basis of gene expression. Adenosine levels were then calculated using formula 3 and underwent the same normalization process (formula 4). The normalized adenosine level was multiplied by the normalized AHCY level to represent the AHCY–ADO complex level (formula 5).

1 $$\log {{{\rm{AHCY}}}}=\log ({{{\rm{AHCY}}}}-{{{{\rm{AHCY}}}}}_{\min }+1)$$

2 $${{{\rm{Norm}}}}.{{{\rm{AHCY}}}}=\frac{\log {{{\rm{AHCY}}}}-{\log {{{\rm{AHCY}}}}}_{\min }}{{\log {{{\rm{AHCY}}}}}_{\max }-{\log {{{\rm{AHCY}}}}}_{\min }}$$

3 $${{{\rm{Adenosine}}}}= \, {{{\rm{NT}}}}5{{{\rm{C}}}}1{{{\rm{A}}}}+{{{\rm{AHCY}}}}+{{{\rm{SLC}}}}39{{{\rm{A}}}}1+{{{\rm{SLC}}}}28{{{\rm{A}}}}1\\ -{{{\rm{ADA}}}}-{{{\rm{CECR}}}}1-{{{\rm{ADK}}}}$$

4 $${{{\rm{Norm}}}}.{{{\rm{Adenosine}}}}=\frac{{{{\rm{Adenosine}}}}-{{{{\rm{Adenosine}}}}}_{\min }}{{{{{\rm{Adenosine}}}}}_{\max }-{{{{\rm{Adenosine}}}}}_{\min }}$$

5 $${{{\rm{AHCY}}}}-{{{\rm{Adenosine\; Complex}}}}={{{\rm{Norm}}}}.{{{\rm{AHCY}}}}\times {{{\rm{Norm}}}}.{{{\rm{Adenosine}}}}$$

### In vitro FTO activity assay

Purified recombinant FTO proteins (1 μg) were mixed with m^6^A ssRNA substrate (1 nmol) in a buffer containing 2 mM l-ascorbic acid, 300 μM α-ketoglutarate, 283 μM (NH_4_)_2_Fe(SO_4_)_2_·6H_2_O, 50 μg/mL BSA, and 50 mM HEPES buffer (pH 7.0), supplemented with or without purified recombinant AHCY proteins and adenosine (Sigma, V900417), and incubated for 3 h at room temperature. The reaction was quenched by addition of 5 mM EDTA and heating at 95 °C for 5 min. After phenol–chloroform extraction and ethanol precipitation, the isolated RNA was digested with RNase P1 (NEB, M0660S) and alkaline phosphatase (Beyotime, D7027). Finally, the m^6^A levels were quantified by LC-MS/MS (Triple Quadrupole Liquid Chromatograph: Agilent 1290 Infinity; Mass Spectrometer: Agilent 6495) to assess the demethylation activity of FTO.

### Protein cross-linking and oligomerization-detection assay

Cells cultured in a 6-well plate were collected and washed twice with pre-chilled PBS; then, 200 μL of polymeric lysis buffer (20 mM HEPES pH 8.0, 0.5% NP-40, 10% glycerol, 137 mM NaCl) supplemented with 1 mM PMSF (Beyotime, ST506), protease inhibitor cocktail (MCE, HY-K0010) and 3 mM DSS cross-linking reagent (Invitrogen, 21655) was added. The samples were incubated at room temperature for 5 min, and the reaction was terminated with 20 mM Tris-HCl pH 7.5. After this step, the samples were lysed on ice for 30 min and centrifuged at 12,000× *g* for 30 min, and the supernatant was transferred to a new EP tube for use in SDS-PAGE or storage at −80 °C. In vitro-purified AHCY and its mutant variants were digested with HRV 3 C protease (TAKARA, 7360) at 4 °C for 16 h, the GST tag was removed using Glutathione MagBeads (GenScript, L00895-10), and 3 mM DSS cross-linking reagent was added and incubated at room temperature for 5 min. The reaction was terminated by addition of 20 mM Tris-HCl pH 7.5, and the samples were used for non-denaturing gel staining or SDS-PAGE.

### ^32^P-labeled RNA cross-linking immunoprecipitation (CLIP)

Cells were transfected with PCDH-FTO-3×HA and/or AHCY WT or mutant (Y193H or D245A) constructs. The cells were collected 48 h post transfection, 8–10 mL PBS was added to the dish, and the cells were irradiated at 400 mJ/cm^2^ and then again at 200 mJ/cm^2^ in a UV crosslinker on ice. After UV crosslinking, the cells were collected into a 50-mL conical tube and centrifuged at 200× *g* for 5 min at 4 °C. The cell pellet was washed in 1 mL cold PBS and centrifuged at 1000× *g* for 5 min at 4 °C. The cell pellet was lysed in 1 mL 1× PXL (1× PBS (cell culture grade), 1% NP40, 0.5% sodium deoxycholate, and 0.1% SDS) and incubated on ice for 10 min. RNase (NEB, M0660S) was used to fragment the RNA. The lysates were then centrifuged in a prechilled ultracentrifuge (using 11 mm × 34 mm polycarbonate tubes in a TLA120.2 rotor) at 32,000× *g* for 25 min at 4 °C. Meanwhile, HA-tagged magnetic beads (MCE, HY-K0201) were washed three times with BWB buffer (1× PBS (cell culture grade) and 0.02% Tween-20) and three times with 1× PXL. The supernatant from the ultracentrifuged lysates was incubated with the washed beads on a rotator at 4 °C for 2 h. The beads were then washed twice with 1× PXL and once each with high-salt wash buffer (1× PBS, 1 M NaCl (final concentration, including the ~140 mM NaCl present in PBS), 1% NP40, 0.5% sodium deoxycholate, and 0.1% SDS), high-stringency wash buffer (15 mM Tris-HCl pH 7.5, 5 mM EDTA pH 8.0, 2.5 mM EGTA pH 8.0, 1% NP40, 1% sodium deoxycholate, 0.1% SDS, 120 mM NaCl, and 25 mM KCl), and low-salt wash buffer (1 mM Tris-HCl pH 7.5 and 5 mM EDTA). The beads were then washed twice with 1× PNK buffer (50 mM Tris-HCl pH 7.5, 10 mM MgCl_2_, and 0.5% NP40), thoroughly resuspended in 80 µL of phosphorylation mix (T4 PNK Mix and ^32^P-γ-ATP (3000 Ci/mmoL)), and incubated in a Thermomixer R at 37 °C for 20 min. The beads were washed once with 1× PNK buffer, once with high-salt wash buffer, and twice with 1× PNK buffer. The beads were flash-spun, and the tubes were placed in a magnet. The supernatants were loaded on a Novex NuPAGE Bis-Tris gel and an autoradiogram was obtained using a Typhoon FLA 7000 IP biomolecular imager (GE).

### RNA immunoprecipitation

In brief, cells were lysed and immunoprecipitated using anti-HA or anti-m^6^A antibody, with normal mouse IgG as a negative control. The mRNA precipitated by anti-HA beads (MCE, HY-K0201), anti-m^6^A (Synaptic Systems, 202003, dilution 1:50), or normal IgG (CST, 5415, dilution 1:125) was converted to cDNA and analyzed by qPCR. The amount of precipitated mRNA was normalized to the input RNA fraction to eliminate possible differences in RNA sample preparation. The PCR primer sequences are provided in Supplementary information, Table S7.

### Biotin–m^6^A motif ssRNA fragment pull-down assays

Purified recombinant FTO protein was mixed with biotin-ssRNA in lysis buffer supplemented with or without adenosine, together with purified recombinant AHCY (WT or mutant) in a final volume of 500 μL. Streptavidin magnetic beads (Promega, Z5481) were added, and the mixture was rotated overnight at 4 °C. The beads were washed four times with lysis buffer and loaded with 50 μL of 2× loading buffer to elute the target protein at 98 °C for 10 min. The collected protein was subjected to SDS-PAGE.

### Monitoring de novo fatty acid synthesis by tracing ^13^C-labeled glucose

To monitor the path of glucose within the culture, cells were seeded in six-well plates at a density of 150,000 cells per well. After three washes with PBS, the cells were cultured in DMEM lacking glucose, glutamine, and sodium pyruvate. The medium was supplemented with 2.0 g/L of U-^13^C-labeled glucose, 2 mM glutamine, and 10% dialyzed FBS (C3820-0500, Holocene) or lipid-free FBS (AB-FBS-DL0500, ABW) and subjected to the specified treatment conditions for 24 h before metabolites were extracted. Cells were washed three times with pre-chilled physiological saline solution. To each well (containing 1.2 × 10^6^ cells), 500 μL of pre-chilled methanol and 200 μL of pre-chilled water were added. After scraping the cells, the suspension was transferred to new EP tubes, and 500 μL of pre-chilled chloroform was added. The mixture was shaken for 10 min, then centrifuged at 12,000 rpm for 10 min at 4 °C. The bottom layer (450 μL) was dried under nitrogen gas after extraction. The samples were then derivatized using 2% methanolic sulfuric acid and injected into a gas chromatography-mass spectrometry system (Shimadzu, GCMS-QP2020NX). Data were corrected for natural isotopes using the R package IsoCorrectoR.

### Oil Red O staining assay

First, 4 × 10^4^ cells were inoculated onto a 24-well cell culture plate. On the following day, the cell culture medium was carefully removed, and the cells were washed once with PBS. The cells were fixed with 4% PFA Fix Solution (Beyotime, P0099) for 10 min at room temperature and rinsed twice with PBS. Oil Red O staining was performed according to the manufacturer’s instructions (Beyotime, C0158M). Nuclei were stained with hematoxylin staining solution (Beyotime, C0107). After addition of Antifade Mounting Medium (Beyotime, P0126), cells were imaged using an automated upright fluorescence microscope (Olympus BX63). The Oil Red O images were analyzed using the “Analyze Particles” module in ImageJ/Fiji to count the number of red lipid droplets and the corresponding number of cells for each image, and the number of red lipid droplets per 100 cells was calculated. The experimental data were analyzed and graphically represented using GraphPad Prism 9.0 statistical software.

### Cell proliferation assay

About 2000 cells per well were seeded into a 96-well plate with 3 replicates for each condition and cultured for the indicated time. Once the cells adhered to the plate, the medium was replaced with fresh complete culture medium containing 1% FBS, or the respective drug treatments were performed. Cell proliferation was measured using CCK-8 reagent (Beyotime, C0040, 1:10). Absorbance was measured at 450 nm using a SpectraMax M microplate reader (Molecular Devices).

### Immunohistochemistry and immunofluorescence

Paraffin-embedded tissue sections were obtained from human or mouse colorectal and lung cancer and stained with antibodies as indicated. The use of human tumor tissues and paired adjacent normal tissues was approved by the institutional review board at Sun Yat-sen University Cancer Center and was in compliance with all relevant ethical regulations. For immunohistochemistry, the tissue sections were deparaffinized with dimethylbenzene, rehydrated in an alcohol gradient, and treated with 3% H_2_O_2_ in methanol to block endogenous peroxidase, followed by antigen-retrieval buffer (ZSGB-BIO, ZLI-9069). For immunofluorescence analysis, the sections were blocked in 10% FBS and incubated overnight at 4 °C with primary antibodies. After binding of secondary antibody coupled with horseradish peroxidase or fluorescein, the sections were stained with DAB (Dako, K5007), and counter-stained with hematoxylin (Servicebio, G1004) for IHC analysis. For immunofluorescence analysis, the cells were blocked and treated with permeabilization buffer consisting of PBS with 5% BSA and 1% Triton X-100. Alexa Fluor 488 (1:500, Invitrogen, A-21206) or Alexa Fluor 568 (1:500, Invitrogen, A-10042) fluorescent secondary antibodies were used. The nuclear staining reagent DAPI (Beyotime, P0131, 1:200) was also applied.

### Xenograft and PDX assay

For xenograft tumor models, 2.5 × 10^6^ tumor cells suspended in a mixture of DMEM and Matrigel (Corning, 356234) at a 1:1 ratio were subcutaneously injected into 4–6-week-old female immunocompromised athymic nude mice (BALB/c nude, Beijing Vital River Lab Animal Technology) in an SPF environment. Tumor size and mouse body weight were measured every 3 days. Tumor volumes were determined using the formula L × W^2^ × 0.5, where L was the longest diameter and W was the shortest diameter. At the endpoint, which occurred around 30–40 days after injection, mice were euthanized by cervical dislocation under sodium pentobarbital anesthesia. The xenograft tumors were dissected for subsequent analyses such as tumor weighing and paraffin sectioning. Primary colon cancer and lung cancer samples were obtained from the Sun Yat-sen University Cancer Center. Freshly resected tumor tissues were washed with sterile PBS, cut into uniform pieces, and transplanted onto the flanks of BALB/c nude mice and Severe Combined Immunodeficiency (SCID) mice. Adenosine, AHCY inhibitors, or AHCY-dimer-perturbing peptides were injected intraperitoneally or subcutaneously. The peptide control sequence was HKFSVSGEGEGDAT, derived from the EGFP protein. During synthesis of AA 7#, a cell-penetrating peptide (iRGD, CRGDKGPDC) was conjugated to the C terminus of the target sequence.^65^ Tumor size was measured and tumor volume calculated every 2 days. When the tumor volume reached 1000 mm^3^, mice were euthanized by cervical dislocation under sodium pentobarbital anesthesia. The xenograft tumors derived from patient samples were dissected for analyses such as tumor weighing and paraffin sectioning.

### Genetically engineered and autochthonous murine models

The *Ahcy* flox/flox (fl/fl) gene mice had LoxP elements inserted into the flanking exons 2 and 3 of the Ahcy gene in the same orientation and were generated from the C57BL/6 strain using CRISPR/Cas9-mediated gene editing (Jiangsu Jicui Yaokang). The resultant floxed allele was confirmed by Sanger sequencing and Southern blotting.

To establish the colorectal cancer model, we engineered mice with the *Ahcy* fl/fl gene and crossed them into a pVillin-CreERT2 background to create a model with *Ahcy* deletion specific to colorectal epithelial cells through TAM treatment (*Ahcy*^fl/fl^Villin-CreERT2). CreERT2 encodes a Cre recombinase fused to a mutant estrogen ligand-binding domain (ERT2) that requires the presence of TAM for activity. We chose 6–8-week-old female *Ahcy*^fl/fl^Villin-CreERT2 (*Ahcy* KO) and littermate control mice (*Ahcy* WT) to establish the autochthonous murine models for colorectal cancer. All mice were intraperitoneally injected once with 10 mg/kg azoxymethane (AOM, Sigma, A5486). The mice were then treated with 2% dextran sulfate sodium (DSS, Aladdin, D274248) in drinking water for 5 days, followed by regular water for 16 days, and three cycles of DSS water treatment. In the fifth week, tamoxifen was administered by gavage at a dose of 200 mg/kg for 5 consecutive days. After 3 months, mice were euthanized by cervical dislocation, and the colons and rectums were dissected for analysis.

To generate in vivo tumor-derived *Ahcy*^fl/fl^ Villin-CreERT2 organoids, tumors were harvested from autochthonous murine models, minced with surgical scissors, and digested in a digestion buffer containing 500 U/mL collagenase type I (Worthington, LS004196) at 37 °C for 40 min with gentle agitation. The cells were then washed, filtered through a 100-µm mesh, and centrifuged for 5 min at 300× *g*. After centrifugation, the cells were embedded in Matrigel and cultured in a medium consisting of 5% FBS and basal medium. Subsequently, the organoids were infected with viruses containing the vector and sgFto1 & sgFto2. Next, 20,000 or 50,000 organoids were inoculated into NCG mice. After 3–5 days of TAM treatment, the mice were divided into four groups: the WT cohort, *Fto* KO cohort, *Ahcy* KO cohort, and *Ahcy*&*Fto* KO cohort. Eight weeks later, the mice were euthanized via cervical dislocation, and the colons and rectums were dissected for analysis. Tumor number and size were recorded and photographed, and the samples were fixed and preserved in mouse tissue fixative solution. The tumor samples were used for m^6^A, metabolomics, and IHC analysis.

### Molecular dynamics simulations

The monomer, dimer, and tetramer structure models of AHCY were constructed on the basis of crystal structures in the Protein Data Bank (PDB) database. The monomer structure model was derived from PDB ID 1LI4, and the other multimer structure models were derived from PDB ID 3NJ4. The apo and FTO/RNA-complex structure models of FTO were also derived and built from the PDB database. Subsequently, the full-length structure of FTO (ID AF-Q9C0B1-F1-model_v4) from the AlphaFold protein structure database (https://alphafold.ebi.ac.uk/) was used as a template, and missing segments of the crystal structure of FTO were completed using MODELLER software (https://salilab.org/modeller/).

The protein–protein interactions between AHCY and FTO were predicted using the ZDOCK server (https://zdock.umassmed.edu/). The initial interactive complexes for different AHCY forms with FTO were constructed. For the output complex-state predictions of each AHCY system, the positions of these interface residues in AHCY/FTO complexes were checked using PyMOL software. The complex with the optimal interface-residue locations was chosen as the initial conformation for each complex model in the following molecular dynamics simulation and optimization.

The structural optimization and energy calculations of complex systems were performed using AMBER16 software. The protein parametrization was performed using the ff14SB force field. The complexes in each system were placed in a rectangular water box, ensuring that the closest distance between the protein and the water box boundary was greater than 10 Å. The TIP3P model was used for the parametrization of water molecules. Na^+^ ions were added to ensure the electroneutrality of the entire system. The molecular dynamics simulation included four stages: energy minimization, heating, pre-equilibration, and equilibration for trajectory production. In the energy minimization stage, both steepest descent and conjugate gradient methods were used for 5000 steps of optimization. During the heating stage, each system was gradually heated from 0 K to 300 K under the NVT ensemble, with all complex atoms restrained with a force constant of 5.0 kcal mol^−1^Å^−2^. In the pre-equilibration stage, each system underwent a 400-ps molecular dynamics simulation with a gradual reduction of the restraint force constant. Finally, each system underwent a 100-ns molecular dynamics simulation in the NPT ensemble (300 K, 1.0 atm), with structural snapshots saved every 10 ps. Subsequently, the molecular dynamics trajectories of the four systems were used for further structural and energy analysis.

For the 100-ns equilibrium trajectories of each system, we first analyzed the root mean square deviation (RMSD) of all Cα atoms of the complex, receptor, and ligand compared with the initial structure over time. We then uniformly selected 100–1000 frames from the molecular dynamics trajectories for MM/GBSA-binding free energy calculations. Four key energy terms (EVDW, EELE, EGB, ESURF), representing nonpolar interactions in the gas phase, polar interactions in the gas phase, polar interactions in the solvent phase, and nonpolar interactions in the solvent phase, were computed and then summed to obtain the binding free energy (ΔH) of the receptor–ligand interactions. The structural alignments of representative conformations and interactions were depicted using PyMOL software.

To investigate the effect of adenosine molecules on the stability of the AHCY dimer and its interaction with FTO, we constructed three more molecular dynamics simulation systems. The molecular charges of adenosine were calculated with Gaussian 09 software, and further parametrization was performed using the GAFF force field.

To rapidly identify peptides that disrupt the formation of AHCY dimers, we used the crystal structure of the AHCY dimer as a template to design and build 11 AHCY/peptide complex systems. The structure model of the AHCY monomer was derived from the A chain of PDB ID 3NJ4, and the structure models of the peptides were derived from the B chain of PDB ID 3NJ4, with the corresponding structural segments with different sequences extracted as the initial structures.

### TCGA data analysis

TCGA data were obtained from the UCSC TCGA Pan-Cancer dataset. The remaining datasets were obtained from https://portal.gdc.cancer.gov/. In the TCGA cohort analysis to assess the influence of AHCY on overall survival, the median value of AHCY expression was used as a cutoff to divide patients into high-AHCY and low-AHCY groups. In the TCGA cohort analysis for the AHCY–ADO complex, we used an optimal cutoff point to classify patients. Those in the top 40% and bottom 30% were defined as the AHCY-high and AHCY-low groups, respectively. In each subgroup, we further examined differences in survival prognosis of the top 15% and bottom 15% of individuals on the basis of AHCY–ADO complex scores. AHCY–ADO complex level was calculated as described above. The log-rank test was used to test for differences in overall survival between subgroups, with *P* < 0.05 considered to indicate a significant difference.

### Animal ethics

Animal experiments were performed in accordance with the guidelines outlined in the 8th edition of the Guide for the Care and Use of Laboratory Animals (NRC 2011). The study was conducted in compliance with the ethical regulations of the institution and the country and was approved by the Institutional Animal Care and Use Committee of Sun Yat-sen University Cancer Center.

### Histopathological analysis

Tissues were obtained and fixed in 10% neutral buffered formalin (Servicebio, G1101) for a period of 1–2 days. Subsequently, the tissues were embedded in paraffin blocks and sectioned at a thickness of 5 μm for H&E staining and immunohistochemistry. H&E images were captured using an Axio Scan Z1 slide scanner (Zeiss). Representative immunohistochemistry staining was quantified by multiplying the staining-intensity score (ranging from 0 to 3) by the percentage of positively stained cells in each representative field. All histopathological analysis was performed by a licensed pathologist.

### Statistics and reproducibility

All statistical data are represented as mean ± standard deviation (S.D.) or standard error of the mean (SEM), and comparisons between two groups were performed using Student’s *t*-test. For comparisons of more than two groups, one-way analysis of variance (One-Way ANOVA) was used, followed by the least significant difference *t*-test (LSD-*t*) for post hoc pairwise comparisons. The chi-square test (*χ*^*2*^) was used for comparisons of binary variables. Pearson’s correlations or non-parametric Spearman’s rank correlations were used to analyze correlations between pairs of continuous variables. All statistical analyses were performed using SPSS 21.0 software. Kaplan–Meier survival curves and log-rank tests were performed using GraphPad Prism 9.

All experimental subjects and quantitative measurements were retained for statistical evaluation. All western blot results, flow cytometry datasets, and cellular imaging findings were derived from at least two distinct experimental repetitions. Detailed documentation of independent biological iterations appears in the corresponding graphical annotations. Randomization protocols governed the distribution of test specimens and living models across comparative cohorts. Subject cohorts had comparable age distributions and gender proportions.

## Supplementary information

Supplementary information, Data S1

Supplementary information, Data S2

Supplementary information, Data S3

Supplementary information, Figure S1

Supplementary information, Figure S2

Supplementary information, Figure S3

Supplementary information, Figure S4

Supplementary information, Figure S5

Supplementary information, Figure S6

Supplementary information, Figure S7

Supplementary information, Table S1

Supplementary information, Table S2

Supplementary information, Table S3

Supplementary information, Table S4

Supplementary information, Table S5

Supplementary information, Table S6

Supplementary information, Table S7

Supplementary information, Video S1

Supplementary information, Video S2

Supplementary information, Video S3

Video legends

## Data Availability

All raw data and experimental materials in this study are available from the corresponding authors upon reasonable request. The raw sequencing data were deposited at the BioProject database under IDs PRJNA1077874, PRJNA1079664, and PRJNA1096464.
