# Supplementary material for: The AHCY–adenosine complex rewires mRNA methylation to enhance fatty acid biosynthesis and tumorigenesis
Source: Cell Res. 2026 Jan 19;36(2):152–72. doi: 10.1038/s41422-025-01213-5 (PMC12848013; doi:10.1038/s41422-025-01213-5)
Supplement: Supplementary file 1 — Supplementary information, Data S1 [file 41422_2025_1213_MOESM1_ESM.pdf]

**Supplementary information, Data S1: Sequence of the AHCY-based ADO sensor C1.**

Pink CMV promoter

Yellow IgK leader

Blue AHCY(1-173 AA)

Bright Green EGFP(149-238)

Green EGFP(1-144)

Pool blue AHCY(174-431 AA)

GTGATGCGGTTTTGGCAGTACATCAATGGGCGTGGATAGCGGTTTGACTCACGGGGATTT
CCAAGTCTCCACCCCATTGACGTCAATGGGAGTTTGTTTTGGCACCAAAATCAACGGGAC
TTTCCAAAATGTCGTAACAACTCCGCCCCATTGACGCAAATGGGCGGTAGGCGTGTACGG
TGGGAGGTCTATATAAGCAGAGCTCTCTGGCTAACTAGAGAACCCACTGCTTACTGGCTTA
TCGAAATTAATACGACTCACTATAGGGAGACCCAAGCTTGGTACCGAGCTCGGATCCACTA
GTAACGGCCGCCAGTGTGCTGGAATTCGGCTTGGGGATATCCACCATGGAGACAGACAC
ACTCCTGCTATGGGTACTGCTGCTCTGGGTTCCAGGTTCCACTGGTGACACAAGTTTGTA
CAAAAAAGTTGGCACCACCGGTATGTCTGACAAACTGCCCTACAAAGTCGCCGACATCG
GCCTGGCTGCCTGGGGACGCAAGGCCCTGGACATTGCTGAGAACGAGATGCCGGGCCT
GATGCGTATGCGGGAGCGGTACTCGGCCTCCAAGCCACTGAAGGGCGCCCGCATCGCT
GGCTGCCTGCACATGACCGTGGAGACGGCCGTCCTCATTGAGACCCTCGTCACCCTGG
GTGCTGAGGTGCAGTGGTCCAGCTGCAACATCTTCTCCACCCAGGACCATGCGGCGGC
TGCCATTGCCAAGGCTGGCATTCCGGTGTATGCCTGGAAGGGCGAAACGGACGAGGAGT
ACCTGTGGTGCATTGAGCAGACCCTGTACTTCAAGGACGGGCCCCTCAACATGATTCTG
GACGACGGGGGCGACCTCACCAACCTCATCCACACCAAGTACCCGCAGCTTCTGCCAG
GCATCCGAGGCATCTCTGAGGAGACCACGACTGGGGTCCACAACCTCTACAAGATGATG
GCCAATGGGATCCTCTGAAGCAGATGGAGAGCCAGCCTCCCAGCCGCCGGGGTCCGGA
CGCCGTCGCCGCGCCGGGAGGAAACGTCTATATCAAGGCCGACAAGCAGAAGAACGGC
ATCAAGGCGAACTTCCACATCCGCCACAACATCGAGGACGGCGGCGTGCAGCTCGCCTA
CCACTACCAGCAGAACACCCCCATCGGCGACGGCCCCGTGCTGCTGCCCGACAACCAC
TACCTGAGCGTGCAGTCCAAACTTTCGAAAGACCCCAACGAGAAGCGCGATCACATGGT
CCTGCTGGAGTTCGTGACCGCCGCCGGGATCACTCTCGGCATGGACGAGCTGTACAAG
GGCGGTACCGGAGGGAGCATGGTGAGAAAGGGCGAGGAGCTGTTCACCGGGGTGGTG
CCCATCCTGGTCGAGCTGGACGGCGACGTAAACGGCCACAAGTTCAGCGTGTCCGGCG
AGGGTGAGGGCGATGCCACCTACGGCAAGCTGACCCTGAAGTTCATCTGCACCACCGG
CAAGCTGCCCGTGCCCTGGCCCACCCTCGTGACCACCCTGACCTACGGCGTGCAGTGC

TTCAGCCGCTACCCCGACCACATGAAGCAGCACGACTTCTTCAAGTCCGCCATGCCCGA
AGGCTACATCCAGGAGCGCACCATCTTCTTCAAGGACGACGGCAACTACAAGACCCGCG
CCGAGGTGAAGTTCGAGGGCGACACCCTGGTGAACCGCATCGAGCTGAAGGGCATCGA
CTTCAAGGAGGACGGCAACATCCTGGGGCACAAGCTGGAGTACAACACCGGAGCAGCA
GCACGCTGGCGCGGGCGGCAGAACCGCGAGCTCCATGCTGCCAAGTCAACTCGGAAG
GTGCCTGCCATCAATGTCAATGACTCCGTCACCAAGAGCAAGTTTGACAACCTCTATGGC
TGCCGGGAGTCCCTCATAGATGGCATCAAGCGGGCCACAGATGTGATGATTGCCGGCAA
GGTAGCGGTGGTAGCAGGCTATGGTGATGTGGGCAAGGGCTGTGCCCAGGCCCTGCGG
GGTTTCGGAGCCCGCGTCATCATCACCGAGATTGACCCCATCAACGCACTGCAGGCTGC
CATGGAGGGCTATGAGGTGACCACCATGGATGAGGCCTGTCAGGAGGGCAACATCTTTG
TCACCACCACAGGCTGTATTGACATCATCCTTGGCCGGCACTTTGAGCAGATGAAGGATG
ATGCCATTGTGTGTAACATTGGACACTTTGACGTGGAGATCGATGTCAAGTGGCTCAACG
AGAACGCCGTGGAGAAGGTGAACATCAAGCCGCAGGTGGACCGGTATCGGTTGAAGAAT
GGGCGCCGCATCATCCTGCTGGCCGAGGGTCGGCTGGTCAACCTGGGTTGTGCCATGG
GCCACCCCAGCTTCGTGATGAGTAACTCCTTCACCAACCAGGTGATGGCGCAGATCGAG
CTGTGGACCCATCCAGACAAGTACCCCGTTGGGGTTCATTTCCTGCCCAAGAAGCTGGA
TGAGGCAGTGGCTGAAGCCCACCTGGGCAAGCTGAATGTGAAGTTGACCAAGCTAACTG
AGAAGCAAGCCCAGTACCTGGGCATGTCCTGTGATGGCCCCTTCAAGCCGGATCACTAC
CGCTACGTCTAG
