# Supplementary material for: The AHCY–adenosine complex rewires mRNA methylation to enhance fatty acid biosynthesis and tumorigenesis
Source: Cell Res. 2026 Jan 19;36(2):152–72. doi: 10.1038/s41422-025-01213-5 (PMC12848013; doi:10.1038/s41422-025-01213-5)
Supplement: Supplementary file 2 — Supplementary information, Data S2 [file 41422_2025_1213_MOESM2_ESM.pdf]

Supplementary information, Data S2: Sequences of the Sensor C1-ABI and ADA-PLY1.

Sequence of the Sensor C1-ABI:

Pink CMV promoter

Yellow IgK leader

Blue AHCY(1-173 AA)

Bright Green EGFP(149-238)

Green EGFP(1-144)

Pool blue AHCY(174-431 AA)

Violet ABI

DarkYellow flag tag

GTGATGCGGTTTTGGCAGTACATCAATGGGCGTGGATAGCGGTTTGACTCACGGGGATTT
CCAAGTCTCCACCCCATTGACGTCAATGGGAGTTTGTTTTGGCACCAAAATCAACGGGAC
TTTCCAAAATGTCGTAACAACTCCGCCCCATTGACGCAAATGGGCGGTAGGCGTGTACGG
TGGGAGGTCTATATAAGCAGAGCTCTCTGGCTAACTAGAGAACCCACTGCTTACTGGCTTA
TCGAAATTAATACGACTCACTATAGGGAGACCCAAGCTTGGTACCGAGCTCGGATCCACTA
GTAACGGCCGCCAGTGTGCTGGAATTCGGCTTGGGGATATCCACCATGGAGACAGACAC
ACTCCTGCTATGGGTACTGCTGCTCTGGGTTCCAGGTTCCACTGGTGACACAAGTTTGTA
CAAAAAAGTTGGCACCACCGGTATGTCTGACAAACTGCCCTACAAAGTCGCCGACATCG
GCCTGGCTGCCTGGGGACGCAAGGCCCTGGACATTGCTGAGAACGAGATGCCGGGCCT
GATGCGTATGCGGGAGCGGTACTCGGCCTCCAAGCCACTGAAGGGCGCCCGCATCGCT
GGCTGCCTGCACATGACCGTGGAGACGGCCGTCCTCATTGAGACCCTCGTCACCCTGG
GTGCTGAGGTGCAGTGGTCCAGCTGCAACATCTTCTCCACCCAGGACCATGCGGCGGC
TGCCATTGCCAAGGCTGGCATTCCGGTGTATGCCTGGAAGGGCGAAACGGACGAGGAGT
ACCTGTGGTGCATTGAGCAGACCCTGTACTTCAAGGACGGGCCCCTCAACATGATTCTG
GACGACGGGGGCGACCTCACCAACCTCATCCACACCAAGTACCCGCAGCTTCTGCCAG
GCATCCGAGGCATCTCTGAGGAGACCACGACTGGGGTCCACAACCTCTACAAGATGATG
GCCAATGGGATCCTCTGAAGCAGATGGAGAGCCAGCCTCCCAGCCGCCGGGGTCCGGA
CGCCGTCGCCGCGCCGGGAGGAAACGTCTATATCAAGGCCGACAAGCAGAAGAACGGC
ATCAAGGCGAACTTCCACATCCGCCACAACATCGAGGACGGCGGCGTGCAGCTCGCCTA
CCACTACCAGCAGAACACCCCCATCGGCGACGGCCCCGTGCTGCTGCCCGACAACCAC

TACCTGAGCGTGCAGTCCAAACTTTCGAAAGACCCCAACGAGAAGCGCGATCACATGGT
CCTGCTGGAGTTCGTGACCGCCGCCGGGATCACTCTCGGCATGGACGAGCTGTACAAG
GGCGGTACCGGAGGGAGCATGGTGAGAAAGGGCGAGGAGCTGTTCACCGGGGTGGTG
CCCATCCTGGTCGAGCTGGACGGCGACGTAAACGGCCACAAGTTCAGCGTGTCCGGCG
AGGGTGAGGGCGATGCCACCTACGGCAAGCTGACCCTGAAGTTCATCTGCACCACCGG
CAAGCTGCCCGTGCCCTGGCCCACCCTCGTGACCACCCTGACCTACGGCGTGCAGTGC
TTCAGCCGCTACCCCGACCACATGAAGCAGCACGACTTCTTCAAGTCCGCCATGCCCGA
AGGCTACATCCAGGAGCGCACCATCTTCTTCAAGGACGACGGCAACTACAAGACCCGCG
CCGAGGTGAAGTTCGAGGGCGACACCCTGGTGAACCGCATCGAGCTGAAGGGCATCGA
CTTCAAGGAGGACGGCAACATCCTGGGGCACAAGCTGGAGTACAACACCGGAGCAGCA
GCACGCTGGCGCGGGCGGCAGAACCGCGAGCTCCATGCTGCCAAGTCAACTCGGAAG
GTGCCTGCCATCAATGTCAATGACTCCGTCACCAAGAGCAAGTTTGACAACCTCTATGGC
TGCCGGGAGTCCCTCATAGATGGCATCAAGCGGGCCACAGATGTGATGATTGCCGGCAA
GGTAGCGGTGGTAGCAGGCTATGGTGATGTGGGCAAGGGCTGTGCCCAGGCCCTGCGG
GGTTTCGGAGCCCGCGTCATCATCACCGAGATTGACCCCATCAACGCACTGCAGGCTGC
CATGGAGGGCTATGAGGTGACCACCATGGATGAGGCCTGTCAGGAGGGCAACATCTTTG
TCACCACCACAGGCTGTATTGACATCATCCTTGGCCGGCACTTTGAGCAGATGAAGGATG
ATGCCATTGTGTGTAACATTGGACACTTTGACGTGGAGATCGATGTCAAGTGGCTCAACG
AGAACGCCGTGGAGAAGGTGAACATCAAGCCGCAGGTGGACCGGTATCGGTTGAAGAAT
GGGCGCCGCATCATCCTGCTGGCCGAGGGTCGGCTGGTCAACCTGGGTTGTGCCATGG
GCCACCCCAGCTTCGTGATGAGTAACTCCTTCACCAACCAGGTGATGGCGCAGATCGAG
CTGTGGACCCATCCAGACAAGTACCCCGTTGGGGTTCATTTCCTGCCCAAGAAGCTGGA
TGAGGCAGTGGCTGAAGCCCACCTGGGCAAGCTGAATGTGAAGTTGACCAAGCTAACTG
AGAAGCAAGCCCAGTACCTGGGCATGTCCTGTGATGGCCCCTTCAAGCCGGATCACTAC
CGCTACGTCTAGGTGCCTTTGTATGGTTTTACTTCGATTTGTGGAAGAAGACCTGAGATGG
AAGCTGCTGTTTCGACTATACCAAGATTCCTTCAATCTTCCTCTGGTTCGATGTTAGATGG
TCGGTTTGATCCTCAATCCGCCGCTCATTTCTTCGGTGTTTACGACGGCCATGGCGGTTC
TCAGGTAGCGAACTATTGTAGAGAGAGGATGCATTTGGCTTTGGCGGAGGAGATAGCTAA
GGAGAAACCGATGCTCTGCGATGGTGATACGTGGCTGGAGAAGTGGAAGAAAGCTCTTT
TCAACTCGTTCCTGAGAGTTGACTCGGAGATTGAGTCAGTTGCGCCGGAGACAGTTGGG
TCAACGTCGGTGGTTGCCGTTGTTTTCCCGTCCCACATCTTCGTCGCTAACTGCGGTGAC
TCTAGAGCCGTTCTTTGCCGCGGCAAAACTGCACTTCCATTATCCGTTGACCATAAACCG
GATAGAGAAGATGAAGCTGCGAGGATTGAAGCCGCAGGAGGGAAAGTGATTCAGTGGAA
TGGAGCTCGTGTTTTCGGTGTTCTCGCCATGTCGAGATCCATTGGCGATAGATACTTGAA
ACCATCCATCATTCCTGATCCGGAAGTGACGGCTGTGAAGAGAGTAAAAGAAGATGATTG
TCTGATTTTGGCGAGTGACGGGGTTTGGGATGTAATGACGGATGAAGAAGCGTGTGAGAT
GGCAAGGAAGCGGATTCTCTTGTGGCACAAGAAAAACGCGGTGGCTGGGGATGCATCGT
TGCTCGCGGATGAGCGGAGAAAGGAAGGGAAAGATCCTGCGGCGATGTCCGCGGCTGA
GTATTTGTCAAAGCTGGCGATACAGAGAGGAAGCAAAGACAACATAAGTGTGGTGGTGGT
TGATTTGAAGGATTACAAGGACGATGACGATAAGGTCTAG

Sequence of the ADA-PYL1:

Pink CMV promoter

Pool blue ADA

Bright Green PYL1

Blue HA tag

GTGATGCGGTTTTGGCAGTACATCAATGGGCGTGGATAGCGGTTTGACTCACGGGGATTT
CCAAGTCTCCACCCCATTGACGTCAATGGGAGTTTGTTTTGGCACCAAAATCAACGGGAC
TTTCCAAAATGTCGTAACAACTCCGCCCCATTGACGCAAATGGGCGGTAGGCGTGTACGG
TGGGAGGTCTATATAAGCAGAGCTCGTTTAGTGAACCGTCAGATCGCCTGGAGACGCCAT
CCACGCTGTTTTGACCTCCATAGAAGATTCTAGAGCTAGCGAATTCATGGCCCAGACGCC
CGCCTTCGACAAGCCCAAAGTGGAACTGCATGTCCACCTAGACGGATCCATCAAGCCTG
AAACCATCTTATACTATGGCAGGAGGAGAGGGATCGCCCTCCCAGCTAACACAGCAGAG
GGGCTGCTGAACGTCATTGGCATGGACAAGCCGCTCACCCTTCCAGACTTCCTGGCCAA
GTTTGACTACTACATGCCTGCTATCGCGGGCTGCCGGGAGGCTATCAAAAGGATCGCCTA
TGAGTTTGTAGAGATGAAGGCCAAAGAGGGCGTGGTGTATGTGGAGGTGCGGTACAGTC
CGCACCTGCTGGCCAACTCCAAAGTGGAGCCAATCCCCTGGAACCAGGCTGAAGGGGA
CCTCACCCCAGACGAGGTGGTGGCCCTAGTGGGCCAGGGCCTGCAGGAGGGGGAGCG
AGACTTCGGGGTCAAGGCCCGGTCCATCCTGTGCTGCATGCGCCACCAGCCCAACTGG
TCCCCCAAGGTGGTGGAGCTGTGTAAGAAGTACCAGCAGCAGACCGTGGTAGCCATTGA
CCTGGCTGGAGATGAGACCATCCCAGGAAGCAGCCTCTTGCCTGGACATGTCCAGGCCT
ACCAGGAGGCTGTGAAGAGCGGCATTCACCGTACTGTCCACGCCGGGGAGGTGGGCTC
GGCCGAAGTAGTAAAAGAGGCTGTGGACATACTCAAGACAGAGCGGCTGGGACACGGCT
ACCACACCCTGGAAGACCAGGCCCTTTATAACAGGCTGCGGCAGGAAAACATGCACTTC
GAGATCTGCCCCTGGTCCAGCTACCTCACTGGTGCCTGGAAGCCGGACACGGAGCATG
CAGTCATTCGGCTCAAAAATGACCAGGCTAACTACTCGCTCAACACAGATGACCCGCTCA
TCTTCAAGTCCACCCTGGACACTGATTACCAGATGACCAAACGGGACATGGGCTTTACTG
AAGAGGAGTTTAAAAGGCTGAACATCAATGCGGCCAAATCTAGTTTCCTCCCAGAAGATG
AAAAGAGGGAGCTTCTCGACCTGCTCTATAAAGCCTATGGGATGCCACCTTCAGCCTCTG
CAGGGCAGAACCTCAGCTCGAGAACTCAAGACGAATTCACCCAACTCTCCCAATCAATC
GCCGAGTTCCACACGTACCAACTCGGTAACGGCCGTTGCTCATCTCTCCTAGCTCAGCG
AATCCACGCGCCGCCGGAAACAGTATGGTCCGTGGTGAGGCGTTTCGATAGGCCACAGA
TTTACAAACACTTCATCAAAAGCTGTAACGTGAGTGAAGATTTCGAGATGCGAGTGGGAT
GCACGCGCGACGTGAACGTGATAAGTGGATTACCGGCGAATACGTCCCGAGAGAGATTA
GATCTGTTGGACGATGATCGGAGAGTGACTGGGTTTAGTATAACCGGTGGTGAACATAGG
CTGAGGAATTATAAATCGGTTACGACGGTTCATAGATTTGAGAAAGAAGAAGAAGAAA
GGATCTGGACCGTTGTTTTGGAATCTTATGTTGTTGATGTACCGGAAGGTAATTCGGAGGA

AGATACGAGATTGTTTGCTGATACGGTTATTAGATTGAATCTTCAGAAACTTGCTTCGATCA
CTGAAGCTATGAAC TACCCATACGATGTTCCAGATTACGCTTGA
