# Supplementary material for: The AHCY–adenosine complex rewires mRNA methylation to enhance fatty acid biosynthesis and tumorigenesis
Source: Cell Res. 2026 Jan 19;36(2):152–72. doi: 10.1038/s41422-025-01213-5 (PMC12848013; doi:10.1038/s41422-025-01213-5)
Supplement: Supplementary file 3 — Supplementary information, Data S3 [file 41422_2025_1213_MOESM3_ESM.pdf]

Supplementary information, Data S3: The correlations of *AHCY* mRNA expression with *ACACA* and *ACACB* mRNA expression were analyzed based on the TCGA database.

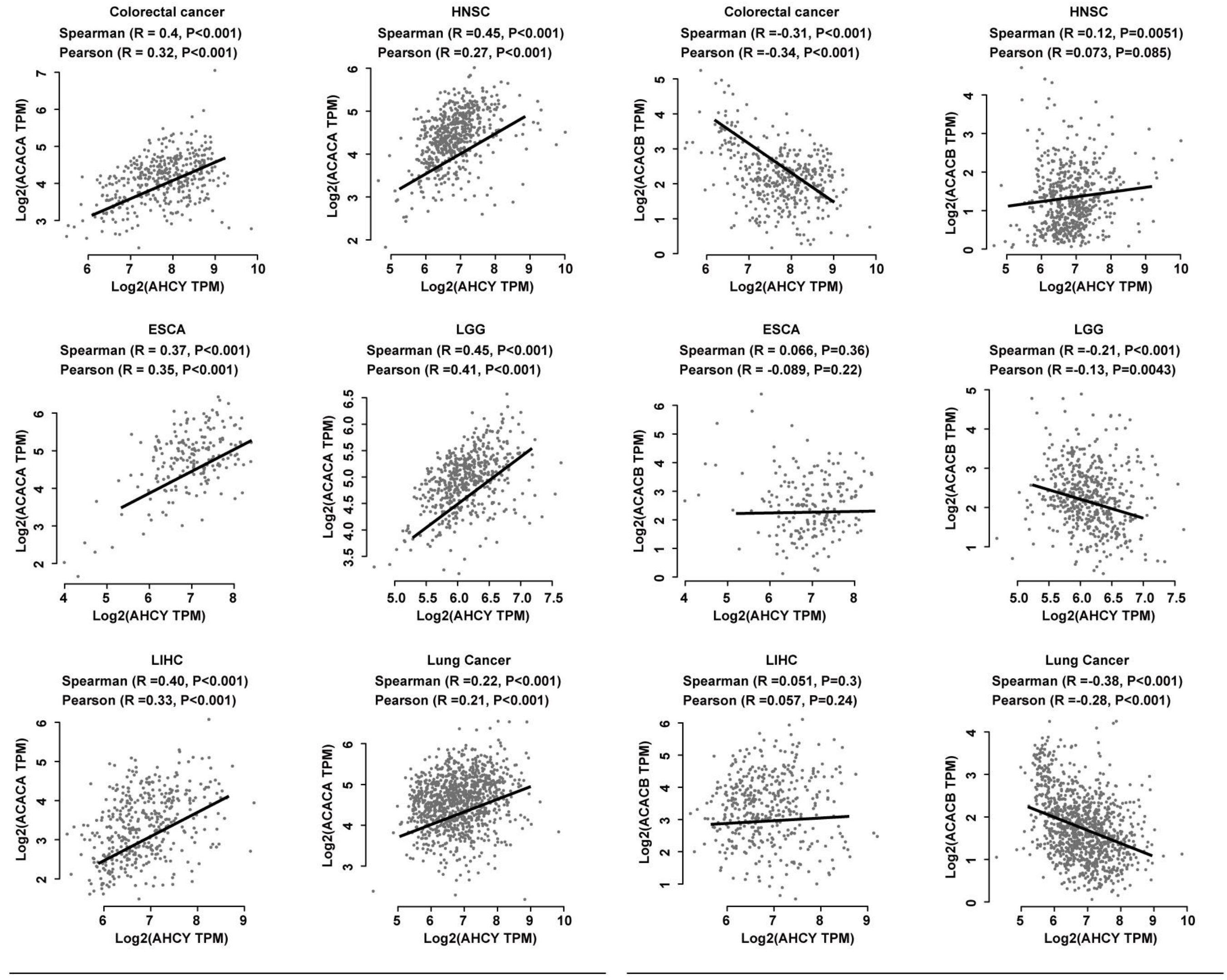

The correlation between *AHCY* and *ACACA*

The correlation between *AHCY* and *ACACB*
