# Supplementary material for: The AHCY–adenosine complex rewires mRNA methylation to enhance fatty acid biosynthesis and tumorigenesis
Source: Cell Res. 2026 Jan 19;36(2):152–72. doi: 10.1038/s41422-025-01213-5 (PMC12848013; doi:10.1038/s41422-025-01213-5)
Supplement: Supplementary file 4 — Supplementary information, Figure S1 [file 41422_2025_1213_MOESM4_ESM.pdf]

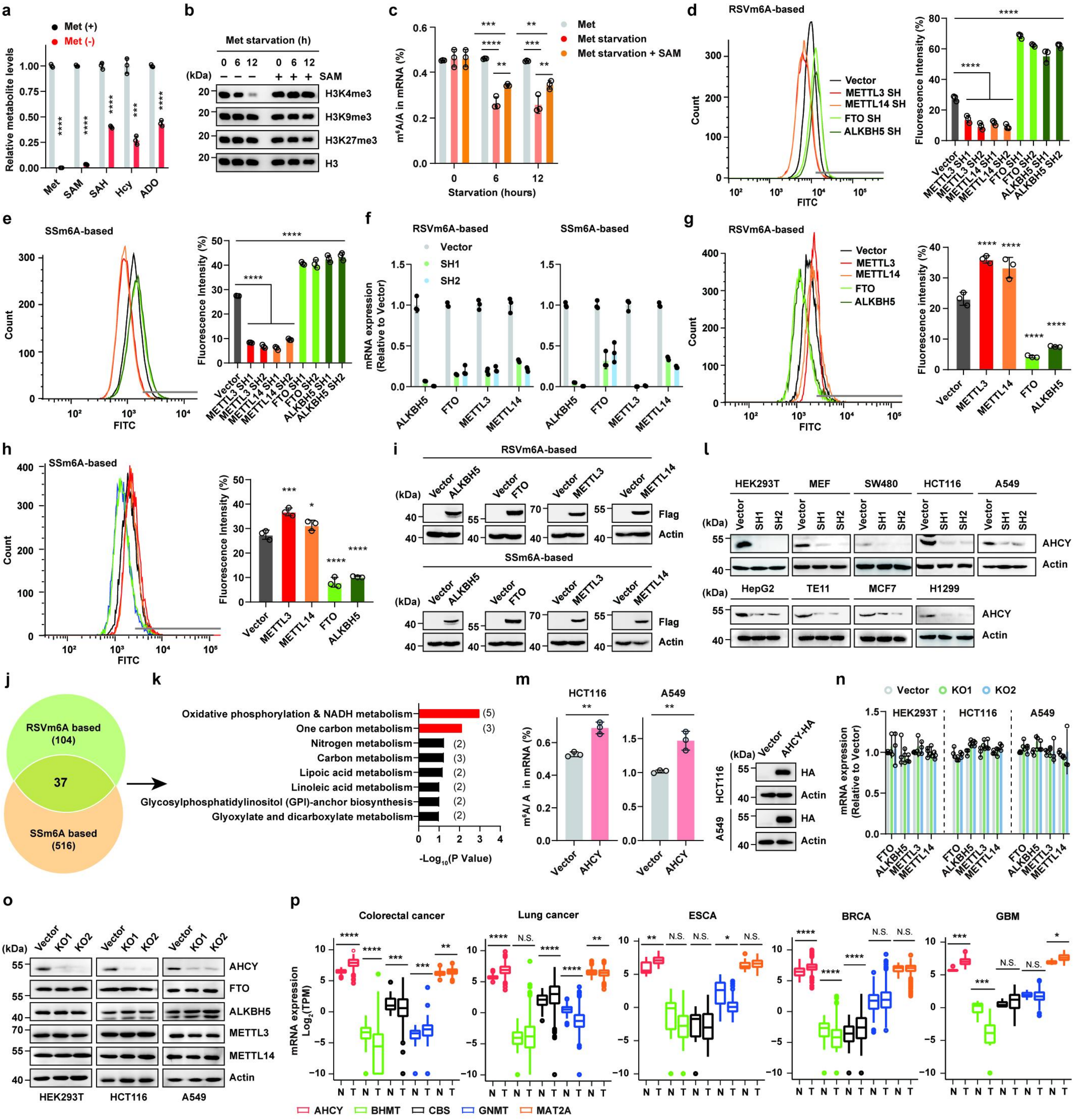

Fig. S1 SAM supplementation cannot completely rescue the decreased mRNA m^{6}A resulting from methionine deficiency. a Relative abundances of metabolites in the methionine cycle in HCT116 cells with or without methionine (Met) restriction. **b, c** Response of histone (**b**) and mRNA m^{6}A (**c**) methylation to methionine restriction in HCT116 cells with or without SAM supplementation. **d-i** Representative flow cytometric analyses of HEK293T SSm6A-based or RSVm6A-based reporter cells transfected as indicated for 48 hours (**d, e, g, h**). Quantitative polymerase chain reaction (qPCR) analysis of HEK293T SSm6A-based and RSVm6A-based reporter cells with or without knockdown of the indicated genes (**f**). Immunoblot analysis of HEK293T SSm6A-based and RSVm6A-based reporter cells overexpressing the indicated proteins (**i**). **j** Venn diagram showing the 37 overlapping genes among the effective candidate genes identified in two

CRISPR knockout screens in cells expressing the SSm6A and RSVm6A reporters. The parenthetical number in each circle indicates the count of enriched genes for that reporter system. **k** KEGG pathway analysis of these candidate genes in **j** using the DAVID online resource. The number in parentheses indicates the count of enriched genes for the corresponding pathway. **l** Immunoblots showing the AHCY protein levels in the indicated cells are presented. **m** LC–MS/MS quantification of the mRNA m^6A/A ratio in HCT116 and A549 cells ectopic expressing vector or AHCY-HA (left). Immunoblot analysis of HCT116 and A549 cells with or without over-expressing AHCY (right). **n, o** qPCR analysis of *FTO*, *ALKBH5, METTL3* and *METTL14* mRNA (**n**) and Immunoblot analysis of the expression of these proteins in wild-type (WT) and AHCY knockout (KO) clones of HEK293T, HCT116 and A549 cells (**o**). **p** Box plots showing the AHCY, BHMT, CBS, GNMT and MAT2A expression profiles in colorectal, lung, esophageal (ESCA), breast (BRCA) and brain (GBM) tumor tissues in TCGA. The medians (lines inside the boxes) and the 25th and 75th percentiles (box limits) are plotted. Data are presented as mean ± S.D. (n=3, unless otherwise specified). Two-tailed unpaired Student's t test (**a, m, p**). One-way ANOVA with LSD-t (**c-e, g, h**). *P < 0.05, **P < 0.01, ***P < 0.001, ****P < 0.0001, N.S., not significant.
