# Supplementary material for: The AHCY–adenosine complex rewires mRNA methylation to enhance fatty acid biosynthesis and tumorigenesis
Source: Cell Res. 2026 Jan 19;36(2):152–72. doi: 10.1038/s41422-025-01213-5 (PMC12848013; doi:10.1038/s41422-025-01213-5)
Supplement: Supplementary file 5 — Supplementary information, Figure S2 [file 41422_2025_1213_MOESM5_ESM.pdf]

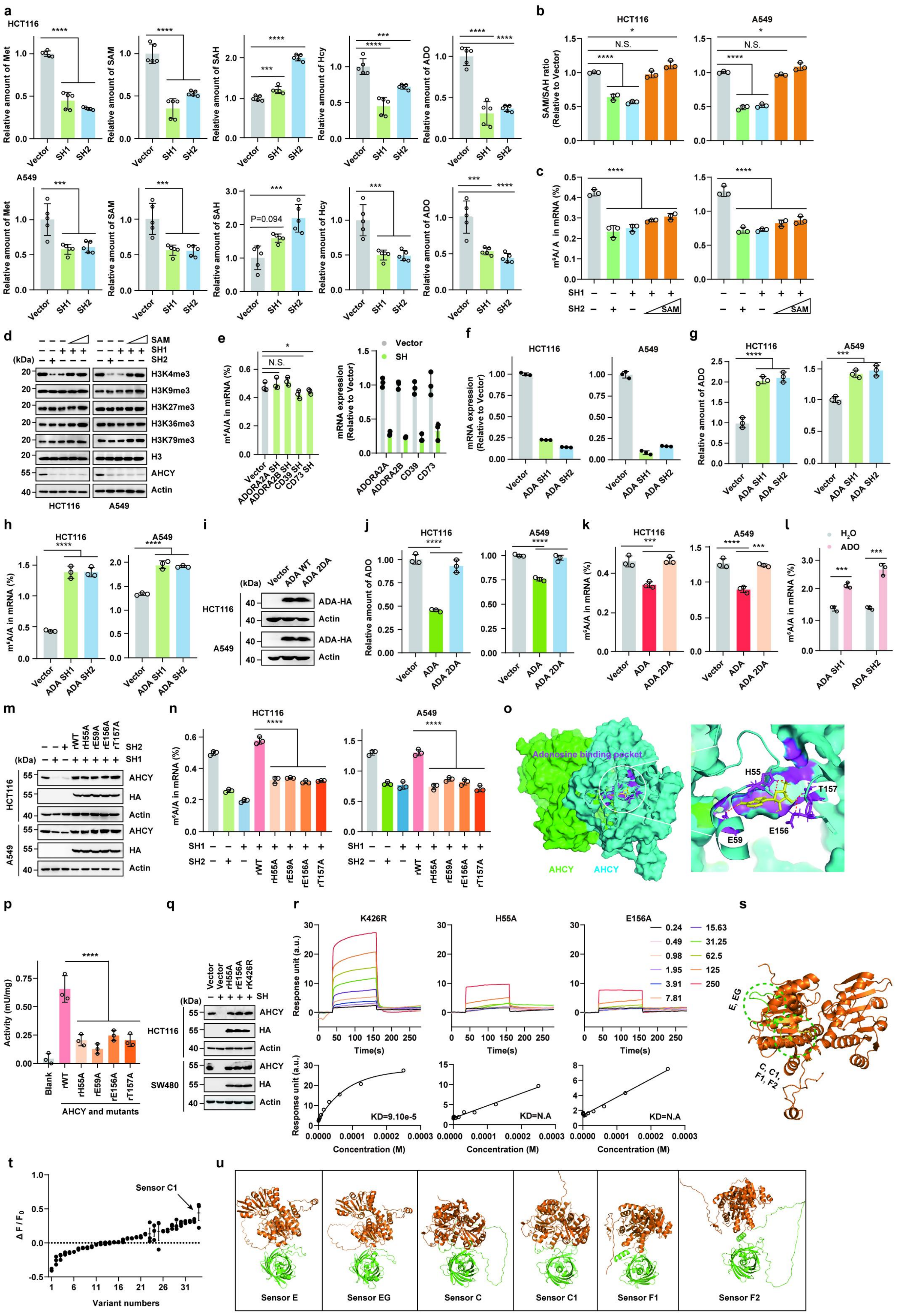
a
HCT116
A549
Relative amount of Met
Relative amount of SAM
Relative amount of SAH
Relative amount of Hcy
Relative amount of ADO
Vector
SH1
SH2
P=0.094
b
SAM/SAH ratio (Relative to Vector)
N.S.
c
m⁶A/ A in mRNA (%)
SAM
d
(kDa)
H3K4me3
H3K9me3
H3K27me3
H3K36me3
H3K79me3
H3
AHCY
Actin
e
m⁶A/A in mRNA (%)
ADORA2A SH
ADORA2B SH
CD39 SH
CD73 SH
mRNA expression (Relative to Vector)
ADORA2A
ADORA2B
CD39
CD73
f
ADA SH1
ADA SH2
g
h
i
ADA WT
ADA 2DA
ADA-HA
j
ADA
k
l
H₂O
ADO
m
rWT
rH55A
rE59A
rE156A
rT157A
HA
n
o
Adenosine binding pocket
H55
T157
E59
E156
AHCY
p
Activity (mU/mg)
Blank
AHCY and mutants
q
rK426R
SH
SW480
r
K426R
H55A
E156A
Response unit (a.u.)
Time(s)
Concentration (M)
KD=9.10e-5
KD=N.A
0.24
0.49
0.98
1.95
3.91
7.81
15.63
31.25
62.5
125
250
s
E, EG
C, C1, F1, F2
t
Sensor C1
Δ F / F₀
Variant numbers
u
Sensor E
Sensor EG
Sensor C
Sensor C1
Sensor F1
Sensor F2

Fig. S2 Intracellular adenosine increases mRNA m^{6}A methylation and AHCY-based adenosine sensor screening. a Quantitative analysis of metabolites involved in the methionine cycle in HCT116 and A549 cells expressing vector or AHCY shRNA (n=5). **b, c** LC-MS/MS quantification of the metabolites SAM/SAH ratio (**b**) and the mRNA m^{6}A/A ratio (**c**) in HCT116 and A549 AHCY shRNA-expressing cells treated with different concentrations (10 and 25 μM) of SAM for 24 hours. **d** The response of histone methylation to AHCY depletion was examined in HCT116 and A549 cells treated with different concentrations (10 and 25 μM) of SAM for 24 hours. **e** LC-MS/MS quantification of the mRNA m^{6}A/A ratio in HCT116 cells expressing vector or shRNAs targeting ADO-selective receptors and extracellular ADO metabolic enzymes (lift). qPCR analysis of HCT116 cells with or without knockdown of the indicated genes (right). **f-h** qPCR analysis (**f**), quantitative analysis of ADO levels (**g**) and LC-MS/MS quantification of the mRNA m^{6}A/A ratio (**h**) in HCT116 and A549 cells expressing vector or ADA shRNA. **i** Immunoblot analysis of HCT116 and A549 cells over-expressing the indicated proteins. **j, k** Quantitative analysis of the ADO level (**j**) and LC–MS/MS quantification of the mRNA m^{6}A/A ratio (**k**) in HCT116 and A549 cells expressing vector, WT ADA or an inactive mutant (ADA D19, 295A; ADA 2DA). **l** LC-MS/MS quantification of the mRNA m^{6}A/A ratio in A549 cells expressing vector or ADA shRNA and treated with 25 μM ADO for 12 hours. **m** Western blot analysis of vector- or AHCY shRNA-transduced HCT116 and A549 cells re-expressing the AHCY WT or alanine (A) substitution mutants at the ADO-binding sites (H55A, E59A, E156A, and T157A). **n** LC-MS/MS quantification of the mRNA m^{6}A/A ratio in AHCY-depleted HCT116 and A549 cells re-expressing the AHCY WT or alanine (A) substitution mutants at the ADO-binding sites (H55A, E59A, E156A, and T157A). **o** Overall view of the ADO-binding pocket in AHCY (PDB: 4PGF). The ADO is shown in yellow. The binding pocket is represented by a purple surface. **p** AHCY enzymatic activity in AHCY KO HEK293T cells re-expressing AHCY or the indicated mutants. **q** Western blot analysis of vector- and AHCY shRNA-transduced cells re-expressing the indicated proteins. **r** For SPR analysis, AHCY mutants were incubated with ADO at the indicated concentrations (upper). KD values were calculated using a steady-state affinity model (bottom). **s** Schematic illustration of the conformational-sensitive circularly permuted enhanced GFP (cpEGFP) inserted into a specific loop region of AHCY. The insertion sites correspond to the two green circled areas, which represent regions exhibiting significant conformational differences between the two states of AHCY before and after ADO binding. Representative candidate sites for cpEGFP insertion include sensor E (E), sensor EG (EG), sensor C (C), sensor C1 (C1), sensor F1 (F1), and sensor F2 (F2). **t** Fluorescence responses of variant-expressing cells in response to 100 μM ADO. $\Delta F = Ft - F0$; Ft, fluorescence value corresponding to time t; F0, initial fluorescence value. **u** The AlphaFold protein structure of the AHCY-based ADO sensor output and the AHCY-cpEGFP chimeric protein structure diagram (brown, AHCY protein structure; green, embedded cpEGFP structure). Data are presented as mean ± S.D. (n=3, unless otherwise specified). Two-tailed unpaired Student's t test (**l**). One-way ANOVA with LSD-t (**a-c, e, g, h, j, k, n, p**). *$P < 0.05$, **$P < 0.01$, ***$P < 0.001$, ****$P < 0.0001$, N.S., not significant.
