# Supplementary material for: The AHCY–adenosine complex rewires mRNA methylation to enhance fatty acid biosynthesis and tumorigenesis
Source: Cell Res. 2026 Jan 19;36(2):152–72. doi: 10.1038/s41422-025-01213-5 (PMC12848013; doi:10.1038/s41422-025-01213-5)
Supplement: Supplementary file 6 — Supplementary information, Figure S3 [file 41422_2025_1213_MOESM6_ESM.pdf]

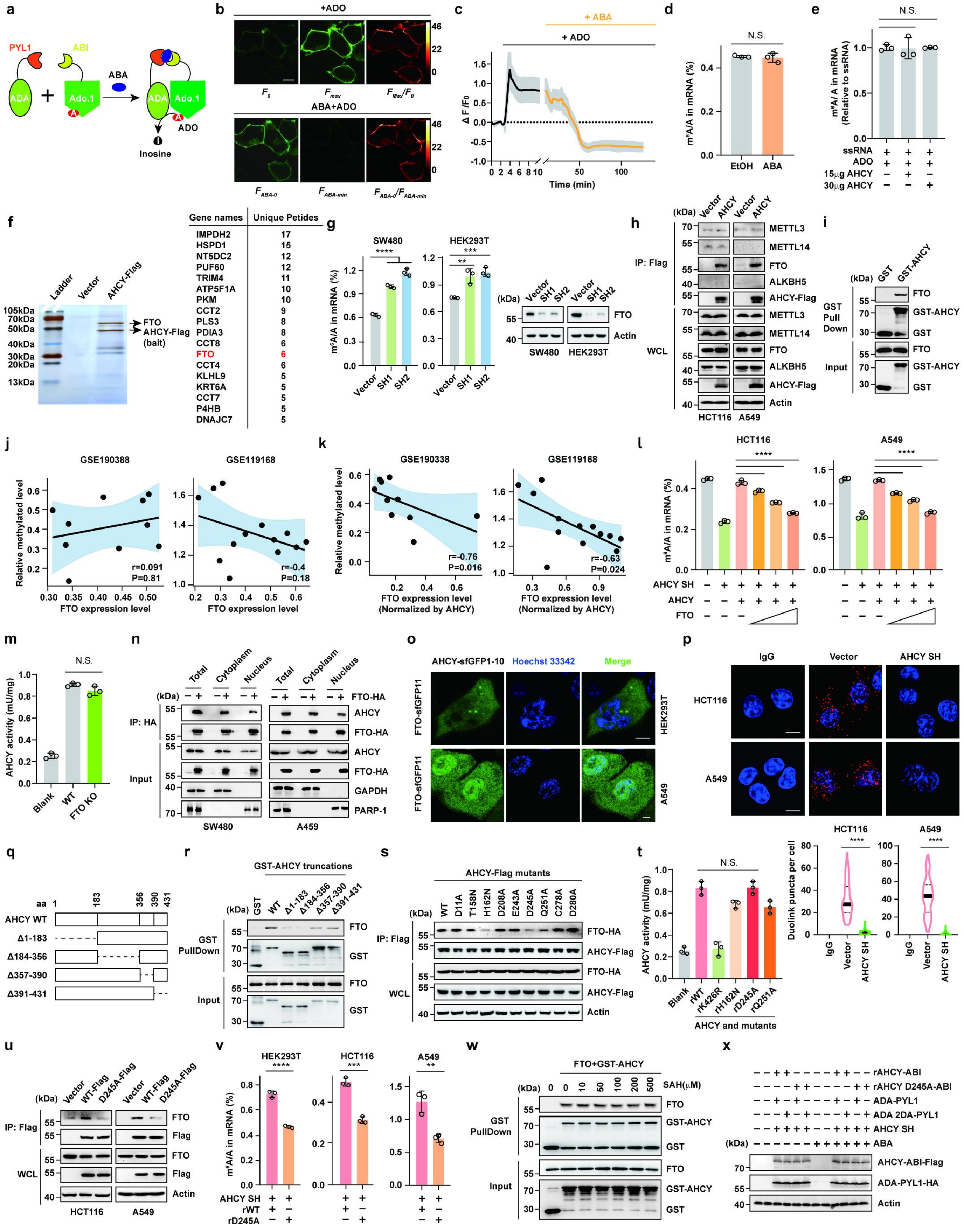

Fig. S3 The interaction of AHCY and FTO increases mRNA m^{6}A levels. a Schematic of the ABA-inducible ABI/PYL1 system for targeting the ADORA2A-ADO complex through co-expression of ABI-ADORA2A-cpEGFP (Ado.1) and PYL1-ADA. **b** Expression and responses of the ADORA2A-based ADO sensor (GRABAdo.1) in HEK293T cells. Images of

sensor fluorescence before and after application of 100 μM ADO (upper) followed by 3 mM ABA (bottom). F_{max}, maximum fluorescence value; F_0, initial fluorescence value; F_{min}, minimum fluorescence value. Scale bar: 10 μm. **c** Representative traces and group analysis of fluorescence changes in GRABAdo.1-expressing cells in response to 100 μM ADO followed by 3 mM ABA. ΔF=Ft-F0; Ft, fluorescence value corresponding to time t; F0, initial starting fluorescence value. **d** LC-MS/MS quantification of the mRNA m^6A/A ratio in HCT116 cells expressing the ADORA2A-ABI and ADA-PYL1 proteins and treated with or without 3 mM ABA for 48 hours. **e** After treating m^6A-modified ssRNA (1 nmoL) with 15 μg or 30 μg of recombinant human AHCY (pH 7.0) at room temperature (24 °C) overnight, LC-MS/MS was used to determine the m^6A/A ratio. **f** An immunoprecipitation assay was performed using an anti-Flag antibody, and AHCY-Flag immunoprecipitates were eluted with the Flag peptide and separated via SDS–PAGE. The gels were stained with Coomassie Brilliant Blue or silver. Selected peptide hits of proteins associated with AHCY-Flag identified through mass spectrometry are shown. **g** LC-MS/MS quantification of the mRNA m^6A/A ratio in SW480 and HEK293T cells transfected with FTO shRNA or vector. **h** Western blot analysis of co-immunoprecipitates of Flag-tagged AHCY and endogenous METTL3, METTL14, FTO and ALKBH5 in the indicated HCT116 and A549 cells. **i** Pull-down assays were performed by mixing purified recombinant His-FTO (2 μg) and GST-AHCY (2 μg) and incubating the mixture for 4 hours. **j, k** Scatterplot showing the correlations between RNA methylation and *FTO* mRNA expression (**j**) with or without normalized by *AHCY* mRNA (**k**) in the tissue of colorectal cancer (GSE190388) and ovarian cancer patients (GSE119168). Pearson's correlation test. **l** LC-MS/MS quantification of the mRNA m^6A/A ratio in AHCY-knockdown HCT116 and A549 cells re-expressing AHCY WT and increasing levels of FTO. **m** AHCY enzymatic activity in AHCY KO HEK293T cells with or without FTO knockout. **n** Whole-cell lysates (total) and the cytosolic and nuclear fractions were prepared from SW480 and A549 cells, and western blot analysis of HA-FTO and endogenous AHCY immunoprecipitates was performed. Cellular fractions generated from equal numbers of cells were analyzed by immunoblotting with the indicated antibodies. **o** Immunofluorescence analyses were performed on HEK293T and A549 cells expressing the AHCY-sfGFP1-10 and FTO-sfGFP11 proteins. The two non-fluorescent fragments of superfolder green fluorescent protein (sfGFP), sfGFP1-10 and sfGFP11, interact upon the binding of the fusion proteins AHCY-sfGFP1-10 and FTO-sfGFP11, resulting in the recombination of these fragments into a functional and fluorescent sfGFP protein. This process indicates the interaction region between AHCY and FTO. Scale bar: 5 μm. **p** Duo-Link II in situ proximity ligation assays (PLA) were performed with anti-AHCY and anti-FTO antibodies in HCT116 and A549 cells, with or without AHCY depletion. The quantification of the Duolink puncta (indicating PLA signals) per cell is provided on the bottom (n=50). Scale bar: 10 μm. **q** Schematic diagram depicting the domains of AHCY. **r** Pull-down assays were performed by mixing purified recombinant His-FTO (2 μg) and GST-AHCY (2 μg) or GST-AHCY exon truncations (2 μg) and incubating the mixture for 4 hours. **s** Western blot analysis of WCLs and anti-Flag immunoprecipitates from HEK293T cells expressing HA-tagged FTO and Flag-tagged AHCY or the indicated mutants. **t** AHCY enzymatic activity in AHCY KO HEK293T cells re-expressing AHCY or the indicated mutants. **u** Western blot analysis of co-immunoprecipitates of Flag-tagged AHCY or the indicated mutants and endogenous FTO in the indicated HCT116 and A549 cells. **v** LC-MS/MS quantification of the mRNA m^6A/A ratio in HCT116, SW480 and A549 cells

re-expressing AHCY WT or D245A mutant. **w** Pull-down assays were performed by mixing purified recombinant His-FTO (2 μg), GST-AHCY (2 μg) and increasing concentrations of SAH and incubating the mixture for 4 hours. **x** Immunoblot analysis showing the protein levels of Flag-tagged AHCY-ABI and HA-tagged ADA-PYL1 in the indicated HCT116 cells. Data are presented as mean ± S.D. (n=3). Two-tailed unpaired Student's t test (**d, m, p, t, v**). One-way ANOVA with LSD-t (**e, g, l**). **P < 0.01, ***P < 0.001, ****P < 0.0001, N.S., not significant.
