# Supplementary material for: The AHCY–adenosine complex rewires mRNA methylation to enhance fatty acid biosynthesis and tumorigenesis
Source: Cell Res. 2026 Jan 19;36(2):152–72. doi: 10.1038/s41422-025-01213-5 (PMC12848013; doi:10.1038/s41422-025-01213-5)
Supplement: Supplementary file 7 — Supplementary information, Figure S4 [file 41422_2025_1213_MOESM7_ESM.pdf]

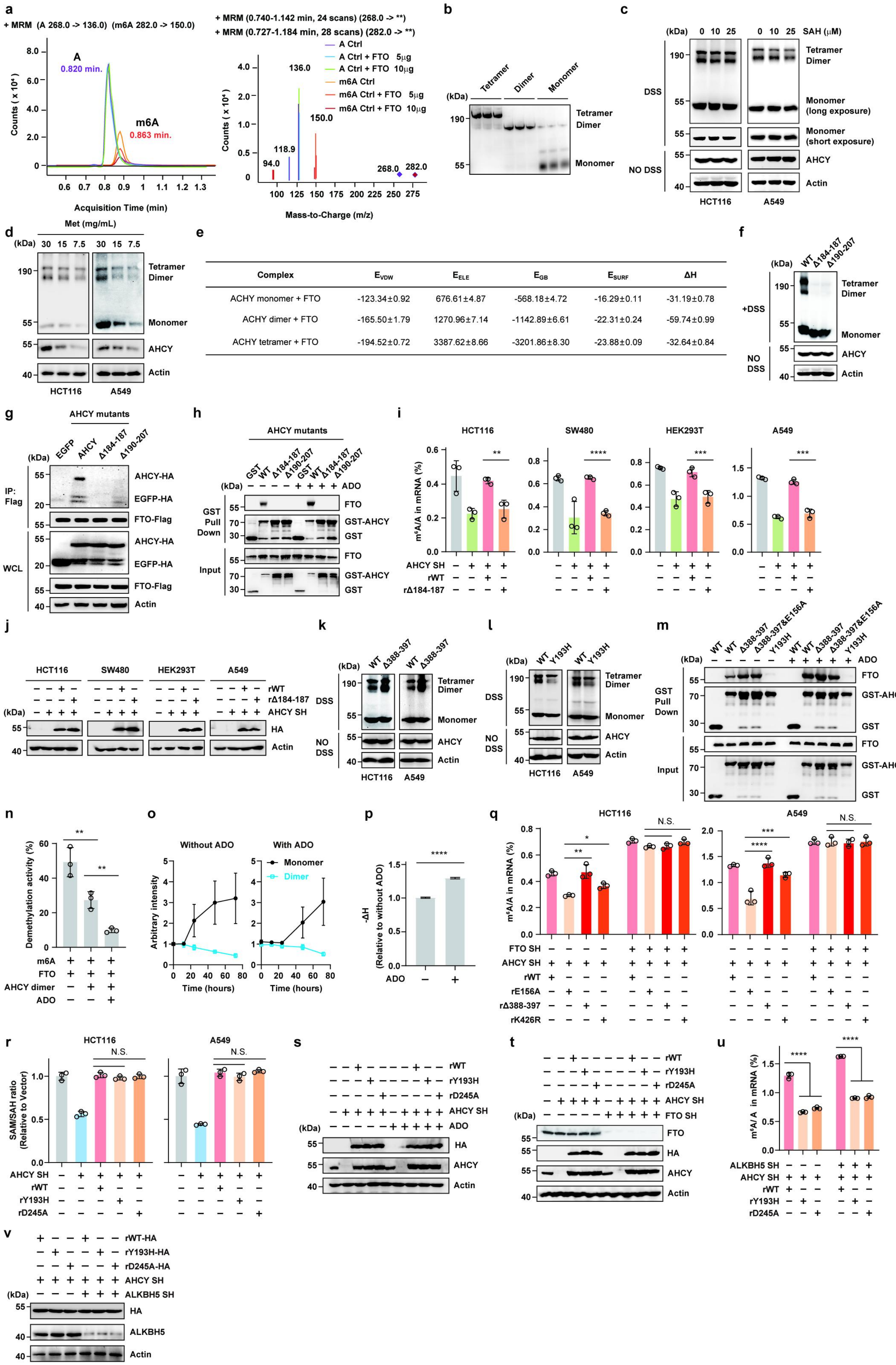

Complex	E_{VDW}	E_{ELE}	E_{GB}	E_{SURF}	ΔH
ACHY monomer + FTO	-123.34±0.92	676.61±4.87	-568.18±4.72	-16.29±0.11	-31.19±0.78
ACHY dimer + FTO	-165.50±1.79	1270.96±7.14	-1142.89±6.61	-22.31±0.24	-59.74±0.99
ACHY tetramer + FTO	-194.52±0.72	3387.62±8.66	-3201.86±8.30	-23.88±0.09	-32.64±0.84

Fig. S4 Methionine and adenosine facilitate AHCY dimerization, and AHCY dimers suppress FTO activity. a FTO efficiently catalyzes the demethylation of m^6A in ssRNAs at neutral pH, as shown by the HPLC profiles (left) and mass spectra (right). **b** Separated of AHCY monomer, dimer and tetramer fractions were analyzed on a native PAGE gel. **c, d** Analysis of the oligomerization state of endogenous AHCY in indicated cells treated with increasing concentrations of SAH (**c**) and methionine (**d**) using disuccinimidyl suberate for protein cross-linking. **e** The binding enthalpy (ΔH, kcal·mol−1), EVDW, EELE, EGB, and ESURF values of FTO with AHCY in various oligomerization states, as determined by molecular docking, are shown. For a binding process, ΔH, or the binding enthalpy, reflects the energy change of the system when the ligand binds to the protein. **f** Analysis of the oligomerization state of AHCY WT, the AHCY Δ184-187 mutant and the Δ190-207 mutant using disuccinimidyl suberate for protein cross-linking. **g** Western blot analysis of WCLs and anti-Flag immunoprecipitates from HCT116 cells expressing Flag-tagged FTO and HA-tagged EGFP, AHCY WT or the indicated mutants. **h** Pull-down assays were performed by mixing purified recombinant His-FTO (2 μg) and GST-AHCY (2 μg) or the indicated mutant (2 μg) and incubating the mixtures in the presence or absence of 10 μM ADO for 4 hours. **i, j** LC-MS/MS quantification of the mRNA m^6A/A ratio (**i**) and immunoblot analysis (**j**) of HCT116, SW480, HEK293T and A549 cells with or without AHCY depletion were re-expressed AHCY WT or the Δ184-187 mutant. **k, l** Analysis of the oligomerization state of AHCY WT or AHCY Δ388-397 mutant (**k**) and AHCY Y193H mutant (**l**) using disuccinimidyl suberate for protein cross-linking. **m** Pull-down assays were performed by mixing purified recombinant His-FTO (2 μg) and GST-AHCY (2 μg) or the indicated mutant (2 μg) and incubating the mixtures in the presence or absence of 10 μM ADO for 4 hours. **n** Demethylation of m^6A in the post-FTO reaction mixtures in vitro. With H_2O as a mock control, purified recombinant AHCY dimers obtained by gel filtration were added to the standard FTO demethylation reaction mixture in the presence or absence of 10 μM ADO. The resultant mixtures were incubated at 37 °C for 10 min and were then analyzed by LC-MS/MS. **o** Graphs showing quantification data for purified recombinant AHCY dimers with or without ADO incubated at 37 °C for the indicated times and then analyzed by native PAGE. **p** The binding enthalpy (ΔH, $kcal \cdot mol^{-1}$) of FTO (PDB: 3LFM) with AHCY dimers (PDB: 3NJ4) in the presence or absence of ADO, as determined by molecular docking. **q** LC-MS/MS quantification of the mRNA m^6A/A ratio in AHCY-depleted HCT116 and A549 cells re-expressing AHCY or AHCY mutants with or without FTO shRNA transduction. **r** LC-MS/MS quantification of the metabolites SAM/SAH ratio in AHCY-depleted HCT116 and A549 cells re-expressing AHCY WT or Y193H and D245A mutants. **s, t** Immunoblot analysis of the indicated A549 cells in the presence or absence of 25 μM ADO (**s**) and with or without FTO shRNA transduction (**t**). **u** LC-MS/MS quantification of the mRNA m^6A/A ratio in AHCY-depleted A549 cells re-expressing AHCY WT or the indicated mutants with or without ALKBH5 shRNA transduction. **v** Western blot analysis of the indicated A549 cells with or without ALKBH5 shRNA transduction. Data are presented as mean ± S.D. (n=3). Two-tailed unpaired Student's t test (**i, p**). One-way ANOVA with LSD-t (**n, q, r, u**). *P < 0.01, **P < 0.01, ***P < 0.001, ****P < 0.0001, N.S., not significant.
