# Supplementary material for: The AHCY–adenosine complex rewires mRNA methylation to enhance fatty acid biosynthesis and tumorigenesis
Source: Cell Res. 2026 Jan 19;36(2):152–72. doi: 10.1038/s41422-025-01213-5 (PMC12848013; doi:10.1038/s41422-025-01213-5)
Supplement: Supplementary file 8 — Supplementary information, Figure S5 [file 41422_2025_1213_MOESM8_ESM.pdf]

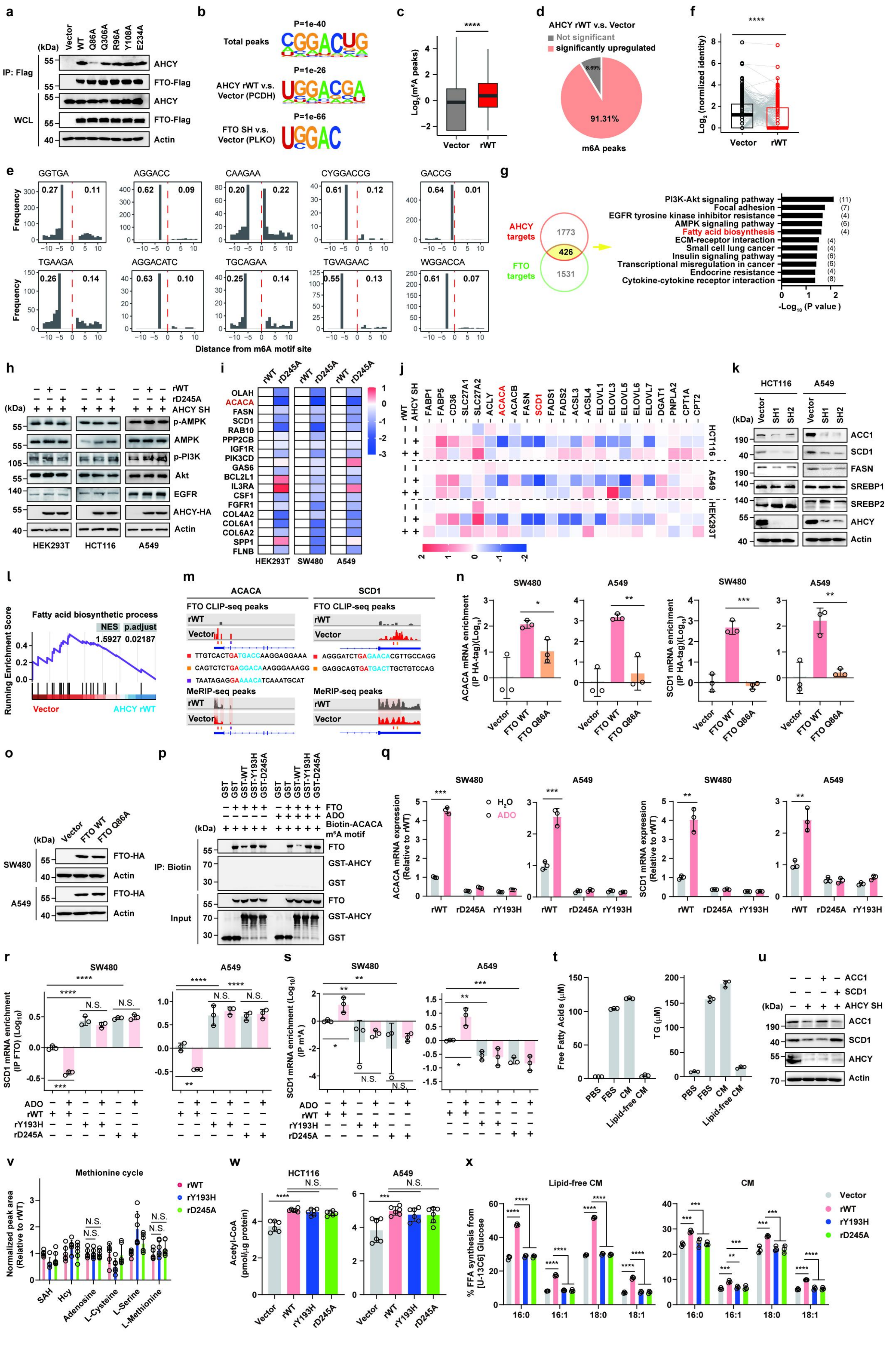

a
Vector WT Q86A Q306A R96A Y108A E234A
(kDa)
IP: Flag
WCL
AHCY
FTO-Flag
AHCY
FTO-Flag
Actin
b
P=1e-40
Total peaks
P=1e-26
AHCY rWT v.s. Vector (PCDH)
P=1e-66
FTO SH v.s. Vector (PLKO)
c
Log2(m6A peaks)
Vector rWT
d
AHCY rWT v.s. Vector
Not significant
significantly upregulated
8.69%
91.31%
m6A peaks
f
Log2 (normlized identity)
Vector rWT
e
GGTGA AGGACC CAAGAA CYGGACCG GACCG
TGAAGA AGGACATC TGCAGAA TGVAGAAC WGGACCA
Frequency
Distance from m6A motif site
g
AHCY targets
FTO targets
1773
426
1531
PI3K-Akt signaling pathway
Focal adhesion
EGFR tyrosine kinase inhibitor resistance
AMPK signaling pathway
Fatty acid biosynthesis
ECM-receptor interaction
Small cell lung cancer
Insulin signaling pathway
Transcriptional misregulation in cancer
Endocrine resistance
Cytokine-cytokine receptor interaction
-Log10 (P value)
h
rWT
rD245A
AHCY SH
p-AMPK
AMPK
p-PI3K
Akt
EGFR
AHCY-HA
Actin
HEK293T HCT116 A549
i
rWT rD245A
OLAH ACACA FASN SCD1 RAB10 PPP2CB IGF1R PIK3CD GAS6 BCL2L1 IL3RA CSF1 FGFR1 COL4A2 COL6A1 COL6A2 SPP1 FLNB
HEK293T SW480 A549
j
rWT
AHCY SH
FABP1 FABP5 CD36 SLC27A1 SLC27A2 ACLY ACACA ACACB FASN SCD1 FADS1 FADS2 ACSL3 ACSL4 ELOVL1 ELOVL3 ELOVL5 ELOVL6 ELOVL7 DGAT1 PNPLA2 CPT1A CPT2
HCT116 A549 HEK293T
k
HCT116 A549
Vector SH1 SH2
ACC1 SCD1 FASN SREBP1 SREBP2 AHCY Actin
l
Fatty acid biosynthetic process
NES p.adjust
1.5927 0.02187
Running Enrichment Score
Vector AHCY rWT
m
ACACA SCD1
FTO CLIP-seq peaks
MeRIP-seq peaks
rWT
Vector
TTGTCACTGATGACCAAGGAGGAAA
CAGTCTCTGAGGACAAAGGGAAAGG
TAATAGAGGAAAACATCAAATGCAT
AGGGATCTGAGAACACGTTGCCAGG
GAGGCAGTGATGACTTGCTGTCCAG
n
SW480 A549
ACACA mRNA enrichment (IP HA-tag)(Log10)
SCD1 mRNA enrichment (IP HA-tag)(Log10)
Vector FTO WT FTO Q86A
o
Vector FTO WT FTO Q86A
SW480 A549
FTO-HA
Actin
p
GST GST GST-WT GST-Y193H GST-D245A
FTO
ADO
Biotin-ACACA m6A motif
IP: Biotin
Input
FTO
GST-AHCY
GST
q
SW480 A549
H2O
ADO
ACACA mRNA expression (Relative to rWT)
SCD1 mRNA expression (Relative to rWT)
rWT rD245A rY193H
r
SW480 A549
SCD1 mRNA enrichment (IP FTO) (Log10)
N.S.
ADO
rWT
rY193H
rD245A
s
SW480 A549
SCD1 mRNA enrichment (Log10) (IP m6A)
t
Free Fatty Acids (μM)
TG (μM)
PBS FBS CM Lipid-free CM
u
ACC1
SCD1
AHCY SH
ACC1 SCD1 AHCY Actin
v
Methionine cycle
rWT rY193H rD245A
Normalized peak area (Relative to rWT)
SAH Hcy Adenosine L-Cysteine L-Serine L-Methionine
w
HCT116 A549
Acetyl-CoA (pmol/μg protein)
Vector rWT rY193H rD245A
x
Lipid-free CM CM
% FFA synthesis from [U-13C6] Glucose
16:0 16:1 18:0 18:1
Vector rWT rY193H rD245A

Fig. S5 The AHCY-adenosine complex increases lipogenesis and the expression of ACACA and SCD1. a Western blot analysis of WCLs and anti-Flag immunoprecipitates from HEK293T cells expressing AHCY and Flag-tagged FTO or the indicated mutants. **b** The motif search of the m^{6}A peaks revealed that they contain the m^{6}A consensus motif GGAC. **c** Analysis of the MeRIP-seq peaks associated with the significantly reduced peaks in FTO CLIP-seq in AHCY-depleted cells re-expressing AHCY. **d** MeRIP-seq peaks showed a significant upregulation in response to the inhibition of FTO CLIP-seq peaks by AHCY. **e** Frequency histogram of motifs, enriched in the Vector group from FTO CLIP-seq, positioned within -10 to +10 bp relative to the DRACH site. **f** Binding of FTO to the GADRACH motif sequence at a consistent RNA location in AHCY-depleted A549 cells with or without AHCY re-expression. **g** Venn diagram showing the overlapping mRNA m^{6}A substrate genes targeted by FTO and AHCY in HCT116 cells (left). The values in the intersecting regions represent the number of peaks shared among the comparison groups, while the values in the non-intersecting regions correspond to the number of peaks unique to each group. KEGG pathway analysis of these candidate genes using the DAVID online resource (right). The number in parentheses indicates the count of enriched genes for the corresponding pathway. **h** Immunoblot analysis of HEK293T, HCT116 and A549 cells with AHCY depletion and re-expression of AHCY WT or D245A. **i** Heatmaps reporting the fold change in the expression of genes in the top five enriched pathways in **g**, as determined by qPCR analysis. **j** Heatmap displaying the fold change in the expression of major fatty acid metabolism-related gene in indicated cells, detected by qPCR. The data presented in the heatmaps are Log_2(Fold change) (**i, j**). **k** Immunoblot analysis of HCT116 and A549 cells with or without AHCY depletion. **l** Gene set enrichment analysis (GSEA) of FTO CLIP-seq peaks corresponding gene in indicated groups. **m** The browser reveals representative transcripts that exhibited significant peaks of change in FTO CLIP-seq and MeRIP-seq. The called peaks are indicated with tick marks underneath. The sequences around each tick mark are shown at the bottom, and the consensus motif GADRACH is highlighted in color. **n** RNA immunoprecipitation assays were performed in the cells using anti-HA antibodies, and qPCR analyses of the precipitated *ACACA* and *SCD1* mRNA was then performed. HA-tagged FTO WT, the FTO Q86A mutant, or an empty vector control (Vector) were ectopically expressed in SW480 and A549 cells. Relative precipitated RNA levels were normalized to those in tumor cells expressing Vector. **o** Immunoblot analysis of SW480 and A549 cells over-expressing the indicated proteins. **p** Purified recombinant FTO (5 μg) and AHCY (5 μg) or the indicated mutant proteins were incubated with the biotin-ACACA m^{6}A motif fragment (3'UTR-3 fragment including GAGGAC sequence, 200 nM) in the presence or absence of 10 μM ADO overnight. Pull-down assays were performed with streptavidin agarose beads. **q** The mRNA levels of *ACACA* and *SCD1* in AHCY-depleted HCT116 and A549 cells re-expressing AHCY WT or mutants were treated with or without 25 μM ADO for 12 hours and examined by qPCR. **r, s** AHCY-depleted SW480 and A549 cells re-expressing AHCY WT or mutants were treated with or without 25 μM ADO for 12 hours. RNA immunoprecipitation assays were performed in the cells using anti-FTO (**r**) and anti-m^{6}A (**s**) antibodies, and qPCR analyses of the precipitated *SCD1* mRNA was then performed. Relative precipitated RNA levels were normalized to those in non-ADO-treated cells expressing AHCY WT. **t** FFAs and TG content in the regular complete culture medium (CM) or Lipid-free CM. **u** Immunoblot analysis of A549 cells with AHCY depletion and re-expression of ACC1 or SCD1. **v** Plots showing the normalized abundances of

methionine cycle intermediates in AHCY-depleted A549 cells re-expressing AHCY WT or the indicated mutants according to metabolomics analysis (n=6). **w** Quantification of Acetyl-CoA in AHCY-depleted HCT116 and A549 cells re-expressing AHCY WT or the indicated mutants (n=6). **x** Steady-state palmitic acid (16:0), palmitoleic acid (16:1), stearic acid (18:0) and oleic acid (18:1) labeling from U-^{13}C-glucose in AHCY-depleted A549 cells re-expressing AHCY WT or mutants cultured in regular complete medium (CM) or Lipid-free CM for 24 hours. Exogenously taken up fatty acids are also incorporated into cellular FFA but are not labelled. Data are presented as mean ± S.D. (n=3, unless otherwise specified). Two-tailed paired Student's t test (**f**). Two-tailed unpaired Student's t test (**c, n, q**). One-way ANOVA with LSD-t (**r, s, v-x**). *P < 0.05, **P < 0.01, ***P < 0.001, ****P < 0.0001, N.S., not significant.
