# Supplementary material for: The AHCY–adenosine complex rewires mRNA methylation to enhance fatty acid biosynthesis and tumorigenesis
Source: Cell Res. 2026 Jan 19;36(2):152–72. doi: 10.1038/s41422-025-01213-5 (PMC12848013; doi:10.1038/s41422-025-01213-5)
Supplement: Supplementary file 9 — Supplementary information, Figure S6 [file 41422_2025_1213_MOESM9_ESM.pdf]

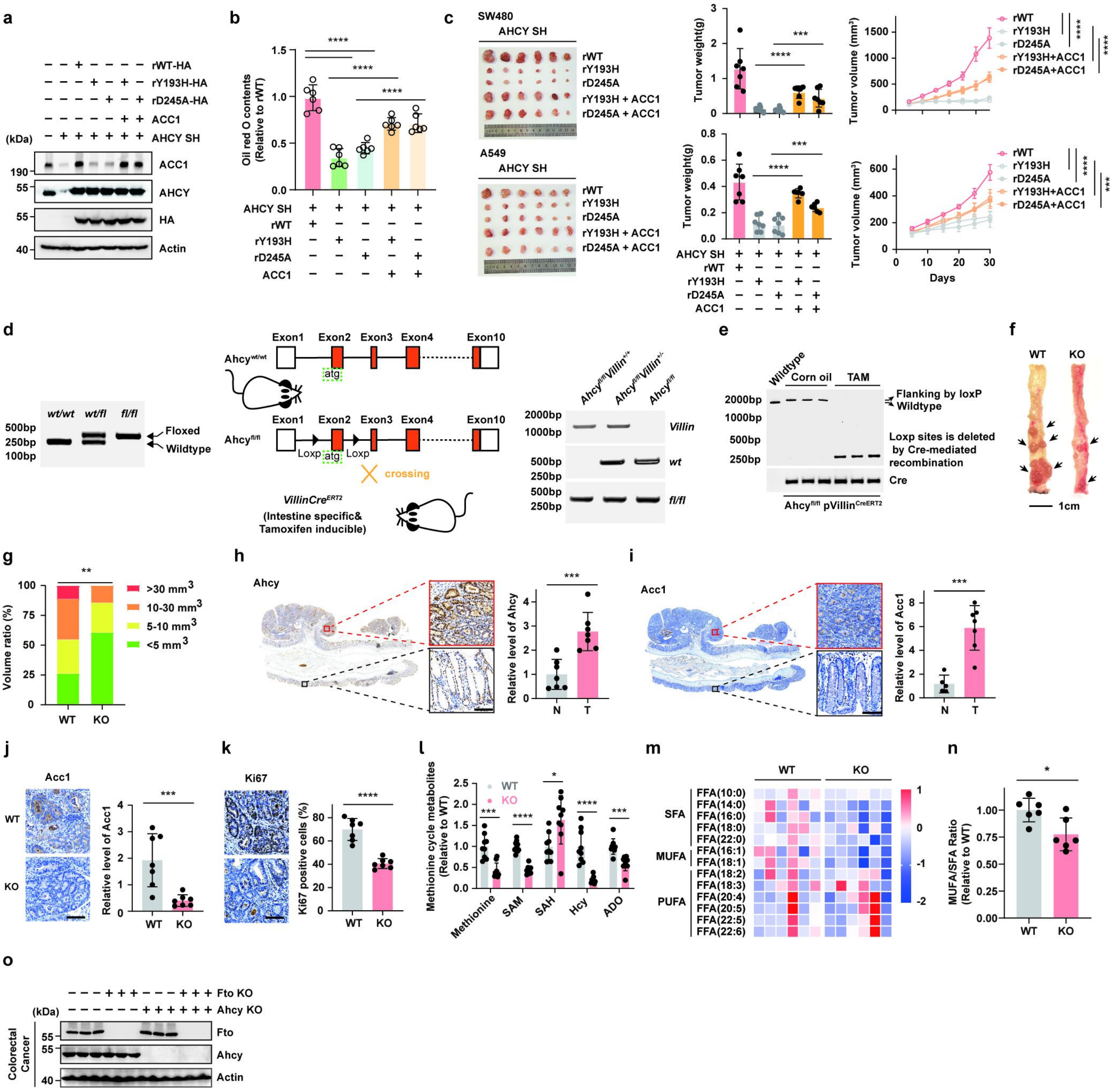

Fig. S6 AHCY enhances colorectal cancer tumorigenesis in vivo. a Immunoblot analysis of AHCY-depleted A549 cells re-expressing AHCY WT or the indicated mutants with or without rescue with ACC1 protein expression. **b** Quantitation of the lipid droplet abundance in the indicated depleted A549 cells (right, n=6). **c** Representative images, weights, and volumes of xenograft tumors formed by AHCY-depleted A549 cells re-expressing AHCY WT or the indicated mutants with or without rescue with ACC1 protein expression in immunocompromised mice (n=7-9). **d** PCR-based genotyping results for Ahcy$^{wt/wt}$, Ahcy$^{fl/fl}$, and heterozygous (Ahcy$^{wt/fl}$) mice (left). Villin^{CreERT2} mice carry a transgene for TAM-inducible Cre recombinase expression in the intestinal epithelium. Villin^{CreERT2} and Ahcy$^{fl/fl}$ mice were crossed to obtain Cre-loxp (fl) mice that express both Cre and a floxed gene segment for intestinal epithelium-specific deletion of the Ahcy gene (middle and right). **e** DNA PCR analysis of colon tissues harvested from mice given the indicated treatments. **f** Representative macroscopic images of longitudinally dissected colons from mice given the indicated treatments. The scale bar represents 1 cm. **g** Volumes

of colorectal tumors harvested from mice in the WT and KO cohorts (n=10-15 mice per group). P values were obtained by the chi-square test. **h, i** Representative IHC stainings and semiquantification of Ahcy (**h**) and Acc1 (**i**) expression in colorectal tumors and correspondent normal adjacent tissues from mice (n=7-10). The scale bars represent 100 μm. **j, k** Representative images of IHC staining and semiquantification of Acc1 (**j**) and Ki67 (**k**) expression in tumors harvested from mice in the WT and KO cohorts. The scale bars represent 50 μm. **l** Quantitative analysis of metabolites in the methionine cycle measured in tumor tissues of Ahcy WT and KO mouse cohorts by LC-MS/MS (n=7-10). **m** Heatmap of FFAs from metabolomics analysis in AHCY-knockout mouse spontaneous colorectal tumors. The color-coded scale on the left indicates the Log_2-transformed fold change after normalization relative to the mean of the corresponding WT group (n=6). **n** The ratios of MUFA (C16:1 and C18:1) to SFA (C16:0 and 18:0) in tumor tissues of Ahcy WT and KO mouse cohorts from **m**. **o** Immunoblot analysis of Fto and Ahcy in indicated colorectal cancer mouse model. Data are presented as mean ± S.D.. Two-tailed unpaired Student's t test (**h-l, n**). One-way ANOVA with LSD-t (**b, c**). Two-way ANOVA with LSD-t (**c**). *P < 0.05, **P < 0.01, ***P < 0.001, ****P < 0.0001.
