# Supplementary material for: The AHCY–adenosine complex rewires mRNA methylation to enhance fatty acid biosynthesis and tumorigenesis
Source: Cell Res. 2026 Jan 19;36(2):152–72. doi: 10.1038/s41422-025-01213-5 (PMC12848013; doi:10.1038/s41422-025-01213-5)
Supplement: Supplementary file 10 — Supplementary information, Figure S7 [file 41422_2025_1213_MOESM10_ESM.pdf]

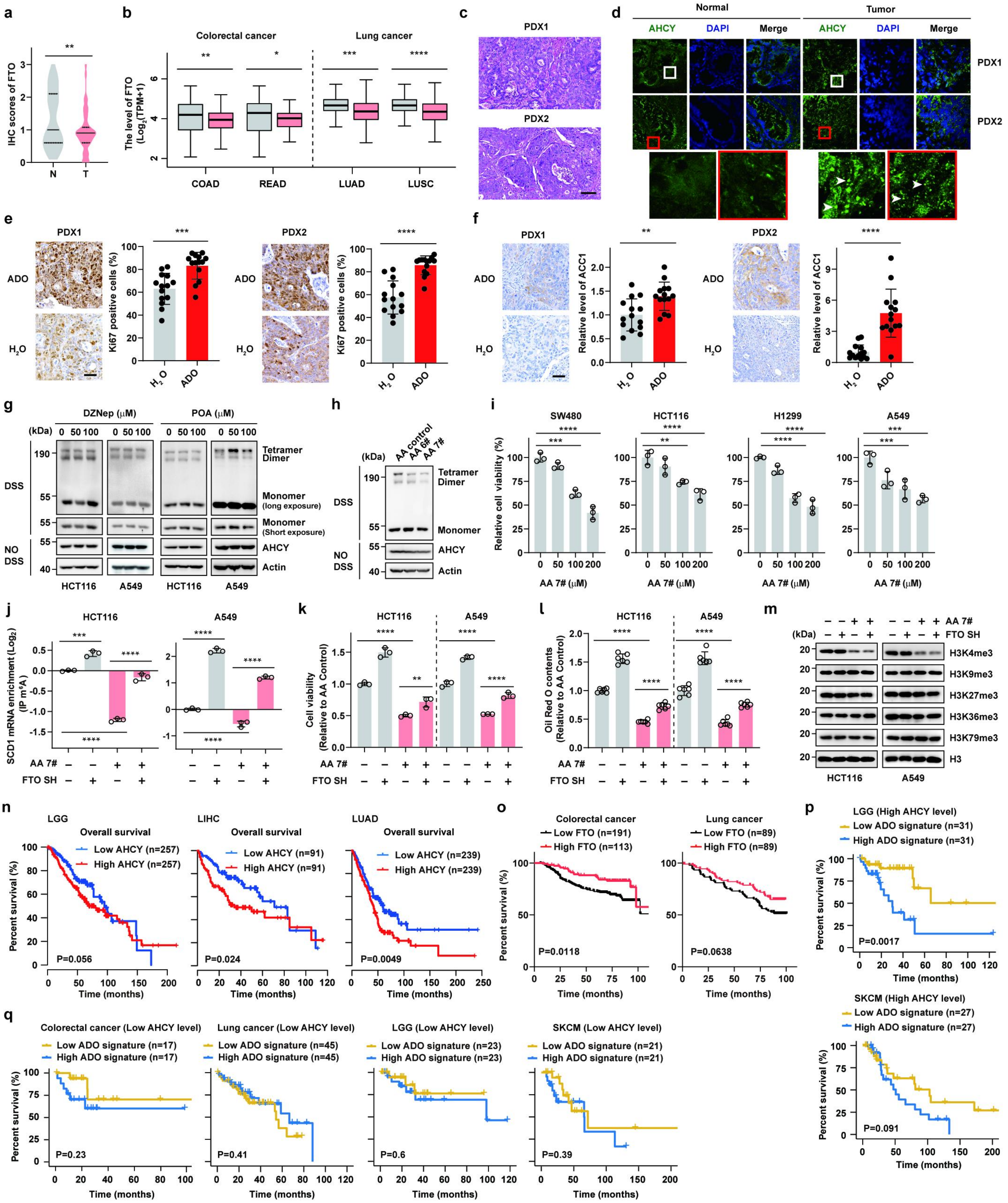

Fig. S7 AHCY dimers promote tumor growth and AHCY-adenosine complex correlates with poor prognosis in tumor patients. a Statistical analysis of FTO protein expression on primary colorectal cancer tissues alongside their adjacent normal tissues. **b** Box plots showing the *FTO* mRNA expression profiles in colon (COAD), rectal (READ), lung (LUAD, LUSC) tumor tissues in TCGA. The medians (lines inside the boxes) and the 25th and 75th percentiles (box limits) are plotted. **c** Representative H&E staining images of colorectal cancer PDX tumors. The scale bars represent 100 μm. **d** Immunofluorescence

analyses were performed on colorectal cancer tumors and the corresponding paracancerous tissues. Scale bar: 5 μm. **e, f** Representative images of IHC staining and semiquantification of Ki67 (**e**) and ACC1 (**f**) expression in harvested colorectal cancer PDX tumors. The scale bars represent 50 μm. **g, h** Analysis of the oligomerization state of endogenous AHCY in HCT116 and A549 cells treated with increasing concentrations of AHCY inhibitors (DZNep and POA) (**g**) and with the 100 μM AHCY dimer-perturbing peptide AA #6 or AA #7 (**h**) for 24 hours using disuccinimidyl suberate for protein cross-linking. **i** Viability of the indicated cells after treatment with increasing concentrations of the AHCY dimer-perturbing peptide (AA #7) for 72 hours. **j-l** MeRIP-qPCR analysis of *SCD1* mRNA (**j**), cells viability (**k**), and quantitation of the Oil Red O content (**l**) in HCT116 and A549 cells with or without FTO shRNA transduction treated with 100 μM of the AHCY dimer-perturbing peptide (AA #7) for 24 (**j**), 72 (**k**) and 48 (**l**) hours, respectively. **m** Western blot analysis of histone methylation levels in HCT116 and A549 cells with or without FTO shRNA transduction treated with 100 μM of the AHCY dimer-perturbing peptide (AA #7) for 24 hours. **n** Kaplan-Meier overall survival curves for patients in the LGG (brain lower grade glioma), LIHC (liver hepatocellular carcinoma) and LUAD (lung adenocarcinoma) cohorts in the TCGA database stratified by the tumor AHCY protein level (high vs. low). **o** Kaplan-Meier overall survival curves for colorectal and lung cancer patients in the local cohort stratified by the tumor FTO protein level (high vs. low). **p, q** Kaplan-Meier overall survival curves of patients in the colorectal, lung, LGG and SKCM (skin cutaneous melanoma) TCGA tissue cohorts with high (**p**) or low (**q**) AHCY level of the TCGA tissue cohort stratified by the ADO gene signature. Data are presented as mean ± S.D, except that tumor volumes are presented as mean ± SEM. Two-tailed unpaired Student's t test (**a, b, e, f**). One-way ANOVA with LSD-t (**i-l**). P values were obtained by the log-rank t test (**n-q**). *P < 0.05, **P < 0.01, ***P < 0.001, ****P < 0.0001.
