# Supplementary material for: The AHCY–adenosine complex rewires mRNA methylation to enhance fatty acid biosynthesis and tumorigenesis
Source: Cell Res. 2026 Jan 19;36(2):152–72. doi: 10.1038/s41422-025-01213-5 (PMC12848013; doi:10.1038/s41422-025-01213-5)
Supplement: Supplementary file 11 — Supplementary information, Table S1 [file 41422_2025_1213_MOESM11_ESM.pdf]

Table S1. Ranked list of genes identified in the positive screen performed in HEK293T cells expressing the RSVm6A reporter using the metabolic enzyme-related gene gRNA library.

Item	Description
**ID**	Gene or miRNA IDs.
**Num**	The number of targeting sgRNAs for this gene in the library.
**Pos|score**	The RRA lo value of this gene in positive selection.
**Pos|p-value**	The raw p-value of this gene for permutation test in positive selection.
**Pos|fdr**	The false discovery rate of this gene in positive selection.
**Pos|rank**	The ranking of this gene in positive selection.
**Pos|goodsgrna**	The number of "good" sgRNAs (sgRNAs whose ranking is below the selected alpha cutoff) in positive selection.
**Pos|lfc**	The log fold change of this gene in positive selection.

ID	Num	Pos|score	Pos|p-value	Pos|fdr	Pos|rank	Pos|goodsgrna	Pos|lfc
**AHCY**	**4**	**0.00028008**	**0.0012753**	**0.562981**	**1**	**3**	**0.99655**
ALG6	4	0.00036147	0.0015878	0.562981	2	4	1.3868
NDUFC1	4	0.00039839	0.0016827	0.562981	3	2	0.48928
CYC1	4	0.00045274	0.0018613	0.562981	4	4	0.83217
CPT1C	4	0.00046741	0.0019115	0.562981	5	3	1.0134
PIGW	4	0.00062712	0.0024697	0.562981	6	4	1.4947
PYGB	4	0.00069671	0.0028045	0.562981	7	2	0.25788
ACAD11	4	0.00076	0.0029943	0.562981	8	4	0.74957
RFK	4	0.00082716	0.0032231	0.562981	9	3	1.1473
PRDX3	4	0.00087927	0.0034464	0.562981	10	4	0.8696
GLT1D1	4	0.0009053	0.0035413	0.562981	11	4	0.65818
CA2	4	0.0011017	0.0042222	0.562981	12	3	1.0459
OXA1L	4	0.0011629	0.0044677	0.562981	13	4	0.93131
PDE12	4	0.001224	0.0046854	0.562981	14	4	0.72674
PLA2G4D	4	0.0013085	0.0049421	0.562981	15	3	0.78271
PIK3R4	4	0.0013998	0.0053328	0.562981	16	3	0.28975
ECH1	4	0.0014732	0.0056286	0.562981	17	3	1.0688
PGAM2	4	0.001493	0.0057123	0.562981	18	4	0.51807
CHST3	4	0.0018106	0.0069513	0.649036	19	2	0.67321
CYP4Z1	4	0.0019593	0.0074648	0.662129	20	2	0.30458
GCSH	4	0.002524	0.0094405	0.742758	21	3	1.0562
PYCR2	4	0.002729	0.010161	0.742758	22	2	0.18484
ALOX5AP	4	0.0028344	0.010484	0.742758	23	4	0.80244
DMGDH	4	0.0028564	0.01054	0.742758	24	3	0.8738
NDUFB9	4	0.00287	0.010574	0.742758	25	3	0.63451
PISD	4	0.0030258	0.011104	0.742758	26	3	0.70333
PCCB	4	0.0030777	0.011305	0.742758	27	1	-0.088616
HSDL2	4	0.0036365	0.013213	0.814131	28	2	0.23747
CYP51A1	4	0.0038285	0.013822	0.814131	29	3	0.76971
AMY2B	4	0.003911	0.014112	0.814131	30	3	0.95269

UQCRC1	4	0.0040362	0.01457	0.814131	31	3	0.93701
MGAT4A	4	0.0041951	0.015022	0.814131	32	1	-0.68138
COX6A1	4	0.004245	0.015144	0.814131	33	4	0.62383
ALDH3B1	4	0.0044056	0.015691	0.818725	34	4	0.49859
GUCY2D	4	0.0049881	0.017617	0.85999	35	4	0.48228
NDUFA9	4	0.0052483	0.018426	0.85999	36	2	0.59656
DLD	4	0.0052961	0.018566	0.85999	37	3	0.72665
ABHD3	4	0.0053115	0.018627	0.85999	38	2	0.37873
OXSM	4	0.0053923	0.018906	0.85999	39	2	-0.13237
PPOX	4	0.0058694	0.02048	0.903156	40	2	0.2261
HSD17B12	4	0.0062438	0.021607	0.903156	41	3	0.9184
ENTPD1	4	0.0063369	0.021898	0.903156	42	4	0.49295
HSD17B11	4	0.006427	0.022188	0.903156	43	2	0.0141
ASAH2B	4	0.0065664	0.022618	0.903156	44	4	0.87759
CYP7A1	4	0.0067889	0.023276	0.903156	45	4	0.58614
HAL	4	0.0069844	0.023896	0.903156	46	1	-0.58416
DGKQ	4	0.0070994	0.024225	0.903156	47	3	0.69083
CHST9	4	0.0071492	0.024437	0.903156	48	4	1.0299
NDUFC2	4	0.0075332	0.025609	0.903975	49	4	0.40216
PNLIPRP1	4	0.0075416	0.025648	0.903975	50	2	0.033402
NDUFV1	4	0.0078907	0.026859	0.903975	51	4	0.71027
OAS2	4	0.0079487	0.027032	0.903975	52	2	0.51027
ACO1	4	0.0079871	0.02715	0.903975	53	4	0.46812
ABHD12	4	0.0084457	0.028562	0.903975	54	4	0.51145
ASS1	4	0.0086552	0.029226	0.903975	55	2	0.14154
UEVLD	4	0.0087215	0.029421	0.903975	56	3	0.86805
NME3	4	0.0087553	0.029516	0.903975	57	3	0.67995
GANC	4	0.008966	0.030147	0.903975	58	2	0.67643
AASDH	4	0.0091206	0.030649	0.903975	59	4	0.50436
PIK3R1	4	0.0091515	0.030749	0.903975	60	4	0.76091
MIOX	4	0.0093038	0.031151	0.903975	61	4	0.53262
MTRR	4	0.0095581	0.031949	0.903975	62	4	0.89058
CYP17A1	4	0.009655	0.032301	0.903975	63	2	0.687
FBP2	4	0.0097679	0.032714	0.903975	64	3	0.35633
GALT	4	0.009976	0.033398	0.903975	65	2	0.38576
ADH1C	4	0.0101	0.033747	0.903975	66	4	0.69908
CA8	4	0.010324	0.034444	0.903975	67	2	0.51103
PDE7B	4	0.010398	0.034651	0.903975	68	3	0.63714
CYP20A1	4	0.010758	0.035884	0.91425	69	4	0.56983
ILVBL	4	0.01088	0.03628	0.91425	70	1	0.085794
NDUFA11	4	0.011104	0.037078	0.91425	71	3	0.62002
BHMT	4	0.011309	0.037765	0.91425	72	2	0.63657
PANK4	4	0.011331	0.037843	0.91425	73	4	0.33386
HPSE2	4	0.011435	0.03815	0.91425	74	2	0.32821
CBR4	4	0.011991	0.039869	0.91425	75	3	0.33034
ATP13A4	4	0.012062	0.040048	0.91425	76	3	0.82147

DGKG	4	0.012224	0.040522	0.91425	77	3	0.61418
ASNS	4	0.012361	0.040924	0.91425	78	3	0.65823
MGAT1	4	0.012546	0.041521	0.91425	79	2	0.17272
CRYZL1	4	0.012628	0.041722	0.91425	80	4	0.81028
PNPLA4	4	0.012852	0.042375	0.91425	81	3	0.62697
MOGAT3	4	0.012987	0.042794	0.91425	82	3	0.56453
PDE7A	4	0.0131	0.043095	0.91425	83	1	0.030866
GK5	4	0.013157	0.04329	0.91425	84	3	0.84061
NDUFS6	4	0.01356	0.044552	0.915159	85	4	0.4628
HYI	4	0.01365	0.044875	0.915159	86	4	0.3658
GALNT12	4	0.013655	0.044881	0.915159	87	2	0.13225
GALNTL6	4	0.014209	0.046499	0.9273	88	2	-0.001487 4
ST6GALNAC6	4	0.014218	0.046522	0.9273	89	4	0.41983
FOLH1	4	0.014748	0.048174	0.938305	90	2	0.22567
PLA2G10	4	0.014779	0.048252	0.938305	91	4	0.51911
NUDT19	4	0.015007	0.048894	0.938305	92	3	0.53543
PLA2G2F	4	0.015119	0.04919	0.938305	93	3	0.63078
PAH	4	0.015877	0.051506	0.957921	94	4	0.3052
THTPA	4	0.015902	0.051612	0.957921	95	4	0.40662
NDUFB4	4	0.016338	0.053029	0.957921	96	2	0.54512
ST8SIA2	4	0.016576	0.053666	0.957921	97	3	0.62979
CYP1A2	4	0.016583	0.053677	0.957921	98	3	0.58084
PIGY	4	0.016978	0.054882	0.957921	99	2	0.13467
PIGA	4	0.017009	0.055011	0.957921	100	4	0.44183
SORD	4	0.017014	0.055039	0.957921	101	3	0.57215
B3GNT4	4	0.017078	0.05524	0.957921	102	3	0.54733
CYP2S1	4	0.017531	0.056479	0.957921	103	2	0.18276
TGDS	4	0.017705	0.056886	0.957921	104	4	0.44454
PGS1	4	0.017749	0.056981	0.957921	105	2	0.44294
PIGM	4	0.017842	0.057238	0.957921	106	4	0.35964
HGLibA	50	0.018338	0.13219	0.980024	107	22	0.08457
CYP27C1	4	0.018412	0.059052	0.971242	108	3	0.73115
ACACA	4	0.018603	0.059615	0.971242	109	3	0.60545
PIK3CG	4	0.018636	0.059688	0.971242	110	3	0.42736
MICAL3	4	0.01879	0.060224	0.971242	111	4	0.46459
NAGS	4	0.019188	0.061317	0.979975	112	2	0.1886
PDE2A	4	0.01974	0.062908	0.980024	113	1	-0.43953
ABHD11	4	0.020014	0.063645	0.980024	114	3	0.56387
AKR1B10	4	0.020292	0.064437	0.980024	115	3	0.27559
GDPD2	4	0.020555	0.065185	0.980024	116	4	0.47077
B3GALT6	4	0.020687	0.065492	0.980024	117	3	0.5051
SAT2	4	0.02082	0.065983	0.980024	118	2	0.44856
CYB5R4	4	0.020843	0.066056	0.980024	119	1	0.12758
AADAT	4	0.020915	0.066285	0.980024	120	3	0.63841
FPGT	4	0.021094	0.066921	0.980024	121	2	0.50522

PCYOX1	4	0.021831	0.069153	0.980024	122	2	0.35689
HEXB	4	0.021946	0.069511	0.980024	123	2	0.45667
GALE	4	0.022392	0.070872	0.980024	124	2	0.38464
NDUFS8	4	0.022456	0.07109	0.980024	125	3	0.40946
GFOD2	4	0.022497	0.071252	0.980024	126	1	-0.38231
ATP13A5	4	0.022506	0.07128	0.980024	127	4	0.32481
ADSL	4	0.02258	0.071503	0.980024	128	2	0.51093
DECR2	4	0.022936	0.072374	0.980024	129	3	0.53162
GGT5	4	0.023033	0.072564	0.980024	130	3	0.55296
AMPD1	4	0.023047	0.072603	0.980024	131	1	-0.48022
NPR3	4	0.023436	0.07339	0.980024	132	3	0.70066
ABO	4	0.023532	0.073574	0.980024	133	3	0.59331
ALDOB	4	0.024016	0.074606	0.980024	134	3	0.64484
ALAS1	4	0.024209	0.075014	0.980024	135	3	0.7592
EXT2	4	0.024216	0.07503	0.980024	136	3	0.55749
B3GNT8	4	0.024821	0.076281	0.980024	137	3	0.5684
PSPH	4	0.025248	0.077095	0.980024	138	2	0.57312
PANK2	4	0.025797	0.078184	0.980024	139	3	0.61718
DHRS4	4	0.026901	0.080522	0.980024	140	2	0.44727
NDUFS3	4	0.027012	0.080757	0.980024	141	3	0.551
NADK	4	0.027335	0.081482	0.980024	142	3	0.57351
LDHAL6A	4	0.027444	0.081694	0.980024	143	2	0.55803
HLCS	4	0.027551	0.081945	0.980024	144	3	0.52505
MAT2B	4	0.027993	0.082811	0.980024	145	2	0.46273
NNT	4	0.028451	0.083715	0.980024	146	2	0.66071
POMT1	4	0.028541	0.083921	0.980024	147	1	-0.14237
PTDSS1	4	0.029089	0.084931	0.980024	148	1	-0.21482
HAAO	4	0.029637	0.086014	0.980024	149	2	-0.027551
ACSM5	4	0.029829	0.086394	0.980024	150	3	0.52653
ACAD8	4	0.030184	0.087125	0.980024	151	2	0.093867
PON2	4	0.030732	0.088235	0.980024	152	3	0.66631
PAPSS2	4	0.030794	0.088392	0.980024	153	2	0.3444
B3GLCT	4	0.030982	0.088782	0.980024	154	3	0.61827
PTGES3	4	0.031215	0.089313	0.980024	155	3	0.49564
NDUFA10	4	0.031279	0.089441	0.980024	156	2	0.35854
SDS	4	0.031447	0.089759	0.980024	157	2	0.39424
POMT2	4	0.031557	0.089938	0.980024	158	3	0.55052
PHYKPL	4	0.032821	0.09241	0.980024	159	3	0.47506
ST3GAL6	4	0.032919	0.092561	0.980024	160	2	-0.12032
PRPS1	4	0.033107	0.092929	0.980024	161	3	0.50349
IDH3B	4	0.033465	0.093677	0.980024	162	2	-0.047086
PIP4K2B	4	0.034477	0.09572	0.980024	163	3	0.5066
COX5B	4	0.034539	0.095826	0.980024	164	3	0.57139
RPE65	4	0.034557	0.095865	0.980024	165	2	-0.32922
GFPT1	4	0.034575	0.095887	0.980024	166	3	0.83044
PLCL2	4	0.034977	0.096529	0.980024	167	3	0.54657

CHST13	4	0.035102	0.096691	0.980024	168	1	-0.026739
CYP8B1	4	0.035377	0.097266	0.980024	169	2	0.4574
ASPA	4	0.035648	0.097818	0.980024	170	2	-0.10867
LTA4H	4	0.035839	0.098181	0.980024	171	3	0.49446
CROT	4	0.036071	0.09865	0.980024	172	2	0.094154
NDUFB8	4	0.036738	0.10007	0.980024	173	3	0.63391
GRHPR	4	0.037827	0.10226	0.980024	174	2	-0.26089
GPAT4	4	0.037831	0.10227	0.980024	175	3	0.61528
ENTPD6	4	0.03795	0.10248	0.980024	176	2	0.11901
GCH1	4	0.038371	0.10325	0.980024	177	1	-0.039978
FUT1	4	0.038737	0.10394	0.980024	178	3	0.44072
SPTLC1	4	0.038915	0.1043	0.980024	179	3	0.41583
POR	4	0.039145	0.10478	0.980024	180	3	0.48482
SOD1	4	0.039507	0.10535	0.980024	181	2	0.060488
WWOX	4	0.039748	0.10582	0.980024	182	3	0.50397
LCT	4	0.04002	0.10635	0.980024	183	3	0.42855
BCAT2	4	0.040637	0.10759	0.980024	184	3	0.50832
SOD3	4	0.041088	0.10837	0.980024	185	2	0.26704
HSD17B14	4	0.04119	0.10853	0.980024	186	3	0.55207
PLA2G2D	4	0.041214	0.10857	0.980024	187	2	0.33515
GALNT10	4	0.041329	0.10878	0.980024	188	3	0.47872
GSTP1	4	0.041461	0.10908	0.980024	189	3	0.53193
GALNT8	4	0.041677	0.10951	0.980024	190	3	0.61092
AGPAT2	4	0.042173	0.11039	0.980024	191	3	0.23753
SDHB	4	0.042239	0.11052	0.980024	192	3	0.71851
CHST4	4	0.042827	0.11181	0.980024	193	3	0.49367
ACAD9	4	0.043258	0.11269	0.980024	194	2	0.21542
CYP24A1	4	0.043799	0.11384	0.980024	195	3	0.32545
NDUFB1	4	0.043878	0.114	0.980024	196	3	0.44609
AK7	4	0.044341	0.11487	0.980024	197	1	-0.31681
RRM2B	4	0.044676	0.11556	0.980024	198	3	0.55083
LIPF	4	0.04536	0.117	0.980024	199	2	0.23771
ACSM3	4	0.045745	0.11774	0.980024	200	2	0.43096
GLUL	4	0.046132	0.11849	0.980024	201	2	0.30644
COX6B1	4	0.04652	0.11929	0.980024	202	2	0.20171
COX4I1	4	0.046779	0.11981	0.980024	203	2	0.4663
GALNT14	4	0.046896	0.12007	0.980024	204	3	0.47876
LIPG	4	0.047046	0.12036	0.980024	205	2	0.20312
SRD5A2	4	0.047122	0.12053	0.980024	206	3	0.52006
PIGO	4	0.0473	0.12081	0.980024	207	2	0.19027
FAHD2A	4	0.047431	0.121	0.980024	208	2	0.036849
AOC3	4	0.047575	0.12125	0.980024	209	3	0.37426
PIP5K1B	4	0.047586	0.12127	0.980024	210	1	-0.59789
FADS1	4	0.047823	0.12166	0.980024	211	2	0.32716
NDUFS7	4	0.047879	0.12176	0.980024	212	3	0.37211
MGLL	4	0.047955	0.12191	0.980024	213	3	0.4236

LPCAT2	4	0.048107	0.12211	0.980024	214	3	0.4117
CYP46A1	4	0.049744	0.12544	0.980024	215	2	0.50612
GDA	4	0.050284	0.12647	0.980024	216	1	-0.28884
ADCY6	4	0.05061	0.12709	0.980024	217	3	0.48145
NME1	4	0.051013	0.12773	0.980024	218	2	0.22126
UQCRFS1	4	0.051216	0.12805	0.980024	219	3	0.60775
NDUFB6	4	0.051361	0.12831	0.980024	220	2	-0.11336
PRPSAP1	4	0.051554	0.1287	0.980024	221	2	0.36796
NT5C2	4	0.051613	0.12882	0.980024	222	3	0.58419
COX7A2L	4	0.051899	0.1294	0.980024	223	3	0.20181
ACSF2	4	0.052171	0.12987	0.980024	224	3	0.60363
NAPRT	4	0.052412	0.13033	0.980024	225	3	0.37204
B4GALT5	4	0.052438	0.13038	0.980024	226	1	-0.33746
TK1	4	0.052572	0.13061	0.980024	227	3	0.52407
NDUFA8	4	0.052653	0.13077	0.980024	228	3	0.57496
FADS3	4	0.053513	0.13239	0.980024	229	3	0.43307
ZADH2	4	0.053875	0.13309	0.980024	230	2	0.13855
NAGPA	4	0.054013	0.13334	0.980024	231	3	0.63829
PMVK	4	0.054196	0.13371	0.980024	232	3	0.487
NDUFB2	4	0.055125	0.13547	0.980024	233	3	0.50977
B3GALT1	4	0.055662	0.13646	0.980024	234	2	0.2785
COX17	4	0.056198	0.13752	0.980024	235	2	0.1562
COLGALT2	4	0.056735	0.13858	0.980024	236	1	-0.1436
INPPL1	4	0.057271	0.13958	0.980024	237	2	0.18153
DHRS1	4	0.057873	0.14076	0.980024	238	3	0.37427
MDH2	4	0.058214	0.14136	0.980024	239	3	0.4586
HK1	4	0.058361	0.1417	0.980024	240	2	0.4149
MTHFS	4	0.058385	0.14174	0.980024	241	3	0.42159
ALOX12	4	0.059765	0.14419	0.980024	242	3	0.40976
SEPHS2	4	0.059793	0.14422	0.980024	243	2	0.30683
NIT2	4	0.059948	0.14455	0.980024	244	1	-0.48438
UQCRC2	4	0.060482	0.1456	0.980024	245	2	0.2253
XYLT2	4	0.060514	0.14566	0.980024	246	3	0.58792
NFS1	4	0.061017	0.14662	0.980024	247	2	0.13597
DERA	4	0.061551	0.14764	0.980024	248	1	-0.013553
AKR7A2	4	0.061606	0.14773	0.980024	249	3	0.38323
NDST2	4	0.062085	0.14854	0.980024	250	2	0.039418
COX8C	4	0.062113	0.14857	0.980024	251	3	0.3329
ABHD13	4	0.062619	0.14946	0.980024	252	1	0.031883
COQ5	4	0.062846	0.1498	0.980024	253	2	0.32024
CPOX	4	0.062851	0.1498	0.980024	254	3	0.45404
NDUFB5	4	0.06314	0.15035	0.980024	255	2	0.1154
OASL	4	0.063686	0.15134	0.980024	256	2	0.13426
COX7A2	4	0.064767	0.1534	0.980024	257	3	0.56064
GPT	4	0.064839	0.15352	0.980024	258	3	0.39296
NAT2	4	0.064916	0.15366	0.980024	259	2	0.35577

NDUFA4L2	4	0.065285	0.15439	0.980024	260	2	0.068009
DPYSL3	4	0.065512	0.15471	0.980024	261	2	0.40624
CHPF	4	0.065811	0.15524	0.980024	262	2	0.25353
MOCS3	4	0.065817	0.15524	0.980024	263	3	0.35214
MAT1A	4	0.066677	0.15686	0.980024	264	3	0.43108
FDX1	4	0.067013	0.15752	0.980024	265	2	0.22568
CTPS2	4	0.067315	0.15811	0.980024	266	3	0.50039
ACOT8	4	0.067466	0.15837	0.980024	267	2	0.15508
ENTPD7	4	0.067945	0.15922	0.980024	268	1	-0.16393
CYB5R3	4	0.068832	0.16098	0.980024	269	2	0.32491
ALDH16A1	4	0.068984	0.16123	0.980024	270	2	0.038016
HAS3	4	0.069007	0.16128	0.980024	271	2	0.30251
B3GNT6	4	0.069587	0.1623	0.980024	272	3	0.33307
PLA2G3	4	0.070069	0.16332	0.980024	273	2	0.35808
MPST	4	0.070362	0.16384	0.980024	274	3	0.49582
PON3	4	0.070541	0.16412	0.980024	275	3	0.4926
NUDT17	4	0.070599	0.16421	0.980024	276	2	0.15615
THEM4	4	0.07067	0.16431	0.980024	277	2	0.26765
PON1	4	0.071129	0.16522	0.980024	278	3	0.2681
ITPA	4	0.072189	0.16713	0.980024	279	1	-0.026003
PTER	4	0.072217	0.16718	0.980024	280	2	-0.14131
ISCA1	4	0.072473	0.16764	0.980024	281	3	0.46828
FOXRED1	4	0.072718	0.16803	0.980024	282	2	0.25417
GANAB	4	0.072765	0.16812	0.980024	283	3	0.36836
GDE1	4	0.073248	0.16901	0.980024	284	2	0.25298
ISCA2	4	0.073352	0.1692	0.980024	285	3	0.38085
CHPF2	4	0.07345	0.16944	0.980024	286	3	0.29322
ACMSD	4	0.073777	0.17003	0.980024	287	2	0.094712
IP6K2	4	0.07404	0.1704	0.980024	288	3	0.41401
AGXT2	4	0.074139	0.17056	0.980024	289	3	0.49825
FMO2	4	0.074305	0.17085	0.980024	290	1	0.011393
MTTP	4	0.074562	0.17131	0.980024	291	3	0.51388
LCAT	4	0.074834	0.17186	0.980024	292	2	0.11378
DCXR	4	0.074877	0.17196	0.980024	293	2	0.20791
ELOVL3	4	0.075362	0.17295	0.980024	294	2	0.24309
COX7A1	4	0.075508	0.17324	0.980024	295	2	0.15529
PCBD2	4	0.075891	0.17398	0.980024	296	1	-0.10969
PTGES2	4	0.076127	0.17446	0.980024	297	3	0.29781
CMAS	4	0.076419	0.17498	0.980024	298	1	-0.40159
CHST2	4	0.076528	0.17517	0.980024	299	3	0.46345
CYP39A1	4	0.076946	0.17599	0.980024	300	1	0.063422
MFNG	4	0.077255	0.17651	0.980024	301	2	0.18747
ASL	4	0.077474	0.1769	0.980024	302	1	-0.19874
ALDH18A1	4	0.078001	0.17793	0.980024	303	2	0.02393
ECHDC3	4	0.07835	0.17844	0.980024	304	3	0.32185
MAT2A	4	0.078528	0.17874	0.980024	305	2	0.24891

MOGAT1	4	0.079179	0.18001	0.980024	306	3	0.51092
MAN2B2	4	0.079475	0.18049	0.980024	307	3	0.42633
PLD2	4	0.079502	0.18055	0.980024	308	2	0.27948
DHRS9	4	0.079581	0.18068	0.980024	309	2	0.075002
FDX2	4	0.079825	0.18103	0.980024	310	2	0.29368
PRODH2	4	0.08061	0.18231	0.980024	311	3	0.34013
NAT10	4	0.080634	0.18235	0.980024	312	3	0.51145
GPX3	4	0.080818	0.18265	0.980024	313	3	0.30923
GCDH	4	0.081025	0.18302	0.980024	314	3	0.41646
ASAH1	4	0.08116	0.18328	0.980024	315	2	0.07598
PGM2L1	4	0.081337	0.18359	0.980024	316	3	0.45196
DBT	4	0.0821	0.18483	0.980024	317	2	-0.15732
B3GAT3	4	0.082211	0.18506	0.980024	318	1	-0.22095
DHDH	4	0.082383	0.18539	0.980024	319	3	0.3312
PYROXD1	4	0.082427	0.18546	0.980024	320	2	0.34029
CPT1A	4	0.082591	0.18578	0.980024	321	2	0.47776
EXT1	4	0.082736	0.18612	0.980024	322	1	-1.3473
COQ10B	4	0.083261	0.18718	0.980024	323	1	-0.37992
PDE8B	4	0.083965	0.18846	0.980024	324	3	0.35067
UQCRQ	4	0.084235	0.18898	0.980024	325	2	0.012259
CKMT2	4	0.08431	0.18906	0.980024	326	1	-0.10763
APRT	4	0.084816	0.18995	0.980024	327	3	0.34016
LPO	4	0.084834	0.18999	0.980024	328	3	0.48188
ASMT	4	0.085565	0.19118	0.980024	329	3	0.30744
AMT	4	0.085672	0.19133	0.980024	330	3	0.28664
UGT1A1	4	0.086406	0.19277	0.980024	331	2	0.3651
CKMT1B	4	0.087074	0.19383	0.980024	332	3	0.30277
AKR1D1	4	0.087452	0.19462	0.980024	333	2	0.20623
GSS	4	0.087975	0.19562	0.980024	334	1	-0.30774
POMGNT1	4	0.088052	0.19571	0.980024	335	3	0.41538
MCCC1	4	0.088063	0.19573	0.980024	336	3	0.41631
GGT6	4	0.088498	0.19658	0.980024	337	1	-0.40602
MCCC2	4	0.088904	0.19741	0.980024	338	3	0.47433
INPP5F	4	0.089021	0.19761	0.980024	339	1	-0.094561
NUDT16L1	4	0.089696	0.19879	0.980024	340	3	0.28024
COX6C	4	0.090065	0.19943	0.980024	341	2	0.39076
ESD	4	0.090587	0.20037	0.980024	342	3	0.33112
ATIC	4	0.091108	0.20123	0.980024	343	1	-0.14034
GDPD1	4	0.091358	0.20167	0.980024	344	3	0.39175
MGAT5	4	0.091616	0.2022	0.980024	345	2	0.29522
OTC	4	0.09163	0.20223	0.980024	346	1	-0.43047
TYR	4	0.092151	0.20302	0.980024	347	1	-0.057219
PFAS	4	0.092364	0.20335	0.980024	348	3	0.38171
PYGL	4	0.092476	0.20359	0.980024	349	3	0.36753
FN3K	4	0.092641	0.20378	0.980024	350	3	0.36092
NMNAT3	4	0.092925	0.20425	0.980024	351	3	0.37786

PLA2G2C	4	0.093155	0.20459	0.980024	352	3	0.38964
GK2	4	0.093193	0.20465	0.980024	353	3	0.34751
AKR1C1	4	0.09367	0.20547	0.980024	354	2	0.1698
ALDH6A1	4	0.093713	0.20559	0.980024	355	1	-0.3233
HSD17B7	4	0.094358	0.20679	0.980024	356	2	0.12547
ACACB	4	0.094753	0.20748	0.980024	357	2	0.20245
NEU3	4	0.095076	0.20807	0.980024	358	3	0.31735
PDE6H	4	0.095222	0.20833	0.980024	359	2	0.54741
DPM2	4	0.095532	0.20889	0.980024	360	3	0.32148
DDO	4	0.095914	0.20954	0.980024	361	2	0.30805
ALDH3A1	4	0.096564	0.21072	0.980024	362	3	0.37765
NAT6	4	0.096609	0.21079	0.980024	363	3	0.38151
GALK1	4	0.096831	0.21118	0.980024	364	2	0.087176
PDE8A	4	0.09735	0.21209	0.980024	365	2	0.016919
SULT1B1	4	0.097869	0.21307	0.980024	366	1	-0.76396
ALDH1B1	4	0.097949	0.2132	0.980024	367	3	0.46889
GGH	4	0.098387	0.21409	0.980024	368	2	0.029495
ARSG	4	0.099346	0.21584	0.980024	369	3	0.29463
PLCH1	4	0.099424	0.21595	0.980024	370	1	-0.47365
CA10	4	0.099582	0.21624	0.980024	371	2	-0.013813
GSTO1	4	0.099814	0.21667	0.980024	372	3	0.34021
ELOVL1	4	0.099934	0.21687	0.980024	373	2	0.3351
GAL3ST2	4	0.10028	0.2176	0.980024	374	3	0.3387
HSD17B1	4	0.10046	0.21781	0.980024	375	2	-0.15826
NSDHL	4	0.10158	0.21978	0.980024	376	3	0.44375
NAA60	4	0.10201	0.22049	0.980024	377	2	0.13839
AMY2A	4	0.10205	0.22056	0.980024	378	3	0.44799
FDPS	4	0.10206	0.22056	0.980024	379	2	0.30473
XYLT1	4	0.10241	0.2211	0.980024	380	3	0.33187
UGT2B15	4	0.10253	0.22134	0.980024	381	1	-0.27824
GPX2	4	0.10312	0.2223	0.980024	382	2	0.30076
TK2	4	0.10324	0.22255	0.980024	383	3	0.25658
PGAM1	4	0.1042	0.22414	0.980024	384	3	0.5605
MCEE	4	0.10444	0.22451	0.980024	385	3	0.35025
PECR	4	0.10459	0.22472	0.980024	386	2	0.24498
CA9	4	0.1048	0.22504	0.980024	387	3	0.32391
LIPJ	4	0.10511	0.22555	0.980024	388	2	-0.007748<br>6
HS3ST3B1	4	0.10562	0.22643	0.980024	389	2	0.30518
UPP1	4	0.10638	0.22772	0.980024	390	3	0.35649
DUOX1	4	0.10653	0.22799	0.980024	391	3	0.40283
DTYMK	4	0.10858	0.23162	0.980024	392	3	0.34948
AK5	4	0.1087	0.23181	0.980024	393	3	0.35618
SMS	4	0.10894	0.23221	0.980024	394	3	0.41383
TMLHE	4	0.10922	0.23264	0.980024	395	2	0.019343
MOCS2	4	0.10961	0.23326	0.980024	396	3	0.35547

LDHC	4	0.10974	0.23346	0.980024	397	2	0.28179
NT5C	4	0.11025	0.23427	0.980024	398	2	0.11908
SPR	4	0.11043	0.23457	0.980024	399	3	0.39928
DSE	4	0.1107	0.23503	0.980024	400	2	0.27359
LDHAL6B	4	0.11076	0.23512	0.980024	401	1	-0.18964
DPEP2	4	0.11125	0.23598	0.980024	402	2	0.10889
SDHC	4	0.11128	0.23601	0.980024	403	1	-1.0536
PDE4D	4	0.1123	0.23784	0.980024	404	3	0.33987
ADCY7	4	0.1123	0.23786	0.980024	405	2	0.06921
SDR39U1	4	0.11305	0.23909	0.980024	406	3	0.36172
HSD17B4	4	0.11333	0.23955	0.980024	407	1	-0.92403
ECHDC2	4	0.11355	0.2399	0.980024	408	3	0.37954
LYG1	4	0.11364	0.24004	0.980024	409	2	-0.029616
DPYSL2	4	0.11401	0.24067	0.980024	410	2	0.1331
ACSM4	4	0.11431	0.24118	0.980024	411	3	0.48222
PI4K2A	4	0.11435	0.24127	0.980024	412	3	0.22069
NDUFA4	4	0.11444	0.24137	0.980024	413	3	0.301
NDUFS4	4	0.11475	0.24193	0.980024	414	2	0.34876
GMPS	4	0.1152	0.24259	0.980024	415	3	0.45511
GNPNAT1	4	0.11533	0.24281	0.980024	416	3	0.28275
SMPD2	4	0.11558	0.2432	0.980024	417	3	0.29317
CYP2E1	4	0.11622	0.24416	0.980024	418	3	0.35165
NDUFAF1	4	0.11624	0.24419	0.980024	419	2	0.13314
PLA2G4A	4	0.11661	0.24476	0.980024	420	2	0.41587
DPEP3	4	0.11686	0.24523	0.980024	421	3	0.31025
NDUFS5	4	0.11736	0.24613	0.980024	422	2	0.42801
PIK3R2	4	0.11741	0.24627	0.980024	423	1	-0.27143
PIGZ	4	0.11788	0.24704	0.980024	424	3	0.22121
NDUFB10	4	0.11814	0.24746	0.980024	425	3	0.3946
GSTA1	4	0.11843	0.24795	0.980024	426	2	0.19361
MAN2A1	4	0.11894	0.24893	0.980024	427	2	0.045331
COX15	4	0.11943	0.24992	0.980024	428	3	0.3913
AK4	4	0.12034	0.25148	0.980024	429	3	0.24874
FH	4	0.12047	0.25169	0.980024	430	3	0.39936
COX11	4	0.12074	0.25214	0.980024	431	2	0.096676
DLAT	4	0.121	0.25262	0.980024	432	3	0.36048
CHST7	4	0.122	0.25445	0.980024	433	2	0.1458
GDPD5	4	0.1225	0.25534	0.980024	434	1	-0.039922
ATP13A3	4	0.12301	0.25622	0.980024	435	1	-0.45514
FADS2	4	0.12352	0.25703	0.980024	436	1	-0.62708
PLCXD3	4	0.12358	0.25715	0.980024	437	2	0.3995
GTDC1	4	0.12396	0.25782	0.980024	438	2	0.27821
SULT1A1	4	0.12403	0.25791	0.980024	439	2	0.062588
ASRGL1	4	0.12415	0.2581	0.980024	440	2	0.36068
ALDH7A1	4	0.12453	0.25867	0.980024	441	1	-0.1842
UGT2B28	4	0.12491	0.25928	0.980024	442	3	0.46165

PGD	4	0.12529	0.26003	0.980024	443	2	0.24483
PIK3C2B	4	0.12548	0.26027	0.980024	444	2	0.28949
LPCAT3	4	0.12615	0.26121	0.980024	445	3	0.24198
ASMTL	4	0.12656	0.26183	0.980024	446	2	0.11102
HADHA	4	0.12663	0.26195	0.980024	447	2	0.33665
GALC	4	0.12707	0.26266	0.980024	448	1	-0.23492
SRXN1	4	0.12759	0.26358	0.980024	449	2	0.17324
B3GNTL1	4	0.12776	0.26394	0.980024	450	3	0.30777
ACOX3	4	0.12803	0.26449	0.980024	451	3	0.44675
MANEAL	4	0.12808	0.26457	0.980024	452	1	0.023679
PTGES	4	0.12909	0.2662	0.980024	453	2	0.27441
GALNT4	4	0.12933	0.26662	0.980024	454	2	-0.003468<br>3
PIGV	4	0.12971	0.26721	0.980024	455	2	0.43929
KLB	4	0.12992	0.26743	0.980024	456	3	0.23861
GSTA3	4	0.1306	0.26847	0.980024	457	3	0.32301
HSD3B7	4	0.1306	0.26848	0.980024	458	1	-0.31409
FUT9	4	0.13068	0.26864	0.980024	459	2	0.34372
CA13	4	0.13126	0.2696	0.980024	460	2	0.21051
ARSE	4	0.13161	0.27018	0.980024	461	2	0.2671
CERS1	4	0.13169	0.27032	0.980024	462	3	0.26234
ELOVL7	4	0.13185	0.27062	0.980024	463	2	0.41803
ADH6	4	0.1321	0.27111	0.980024	464	3	0.23034
DHRS7B	4	0.13224	0.2714	0.980024	465	2	-0.072946
DOLK	4	0.13238	0.27164	0.980024	466	3	0.36118
ACER3	4	0.13312	0.27287	0.980024	467	1	-0.058736
GPAT3	4	0.13321	0.27299	0.980024	468	2	-0.37646
ALG1	4	0.13413	0.2745	0.980024	469	1	-0.29015
CYB561A3	4	0.13513	0.27605	0.980024	470	1	-0.18681
PNPO	4	0.13514	0.27606	0.980024	471	3	0.24819
ACSBG1	4	0.13517	0.27611	0.980024	472	2	0.10372
HGD	4	0.13536	0.27647	0.980024	473	2	0.20445
DHDDS	4	0.13555	0.27681	0.980024	474	3	0.36237
ENTPD5	4	0.13576	0.27715	0.980024	475	2	0.29399
PFKP	4	0.13614	0.27777	0.980024	476	1	-0.32276
PTGIS	4	0.13625	0.27794	0.980024	477	3	0.28953
ACSM2B	4	0.13664	0.27853	0.980024	478	2	0.26645
ENTPD2	4	0.13667	0.27855	0.980024	479	3	0.21876
GALNT3	4	0.13714	0.27925	0.980024	480	1	-0.59788
PLD6	4	0.13764	0.28006	0.980024	481	1	0.013374
BST1	4	0.13773	0.28022	0.980024	482	3	0.46111
PDE6G	4	0.13849	0.28147	0.980024	483	3	0.42815
QPRT	4	0.13871	0.28185	0.980024	484	2	0.31086
CSGALNACT1	4	0.13891	0.28214	0.980024	485	2	0.21026
DHRS13	4	0.13948	0.28307	0.980024	486	3	0.22361
PIGP	4	0.13965	0.28339	0.980024	487	1	-0.20418

KL	4	0.14015	0.2842	0.980024	488	1	-0.45845
CYP2C9	4	0.14033	0.28455	0.980024	489	3	0.20914
IMPA1	4	0.14047	0.28476	0.980024	490	3	0.35537
ADCY2	4	0.14061	0.28502	0.980024	491	3	0.28594
ABHD14A	4	0.14189	0.28689	0.980024	492	3	0.22122
CYP2C19	4	0.14189	0.28689	0.980024	493	2	0.322
LARGE2	4	0.14203	0.28716	0.980024	494	3	0.30219
GSTK1	4	0.14309	0.28905	0.980024	495	3	0.39246
ITPK1	4	0.14349	0.28969	0.980024	496	2	0.32214
PANK3	4	0.14364	0.29003	0.980024	497	1	-0.57226
CYP2D6	4	0.14369	0.2901	0.980024	498	2	0.043898
ETNK2	4	0.14389	0.29045	0.980024	499	3	0.44531
GNPAT	4	0.14414	0.29082	0.980024	500	1	-0.96761
LPIN3	4	0.14432	0.29108	0.980024	501	3	0.30039
SCD	4	0.14469	0.29155	0.980024	502	2	0.26475
CYP2A13	4	0.1449	0.29196	0.980024	503	3	0.38648
DHCR24	4	0.14514	0.29237	0.980024	504	1	-0.049388
FPGS	4	0.14533	0.29272	0.980024	505	3	0.22621
PNMT	4	0.14562	0.29316	0.980024	506	3	0.31461
PMM2	4	0.1457	0.29328	0.980024	507	2	0.088994
NUDT12	4	0.1463	0.29422	0.980024	508	3	0.43468
AHCYL1	4	0.14663	0.29484	0.980024	509	2	0.09143
ST6GALNAC2	4	0.14707	0.29549	0.980024	510	3	0.34927
MOCOS	4	0.14765	0.29641	0.980024	511	3	0.22251
NUDT14	4	0.14809	0.29719	0.980024	512	3	0.27252
AOX1	4	0.14812	0.29721	0.980024	513	1	-0.73192
CNDP1	4	0.14912	0.29874	0.980024	514	1	-0.35166
CHST6	4	0.14933	0.29908	0.980024	515	2	0.15445
MTR	4	0.14953	0.29934	0.980024	516	2	0.17935
PTGDS	4	0.1497	0.2996	0.980024	517	3	0.20104
CDIPT	4	0.15011	0.30032	0.980024	518	2	0.22863
UXS1	4	0.15058	0.30109	0.980024	519	3	0.27252
NOS3	4	0.15102	0.30179	0.980024	520	3	0.26799
LIPT1	4	0.15159	0.3027	0.980024	521	3	0.30495
ALG3	4	0.15176	0.30299	0.980024	522	3	0.27356
OXCT2	4	0.15177	0.303	0.980024	523	2	0.34305
DHRS2	4	0.15209	0.30349	0.980024	524	2	-0.0479
MBOAT7	4	0.15235	0.3039	0.980024	525	3	0.26441
GUCY1A3	4	0.15258	0.30422	0.980024	526	2	-0.052606
FAHD1	4	0.15299	0.3049	0.980024	527	2	0.055369
CA12	4	0.15308	0.30502	0.980024	528	1	-0.47333
ENO2	4	0.15309	0.30504	0.980024	529	3	0.2023
AGK	4	0.15339	0.30555	0.980024	530	3	0.35741
ST3GAL2	4	0.15384	0.30634	0.980024	531	3	0.18535
CYP4F22	4	0.15407	0.30664	0.980024	532	1	-0.41459
MTHFD2	4	0.15413	0.30672	0.980024	533	3	0.29808

GUCY2C	4	0.15456	0.30747	0.980024	534	2	-0.33161
COQ2	4	0.15518	0.30854	0.980024	535	3	0.22916
ALOX15	4	0.15566	0.30924	0.980024	536	2	0.21419
VKORC1L1	4	0.15604	0.30988	0.980024	537	2	0.15657
ALDH8A1	4	0.15653	0.31066	0.980024	538	3	0.1668
NDUFA5	4	0.15654	0.31067	0.980024	539	1	-0.2078
CYP27B1	4	0.15698	0.31132	0.980024	540	3	0.21529
LPL	4	0.15703	0.31142	0.980024	541	1	-0.0077646
ADH5	4	0.15728	0.31181	0.980024	542	3	0.27026
CYP1B1	4	0.15792	0.31287	0.980024	543	2	0.287
GGT7	4	0.15819	0.31325	0.980024	544	3	0.22909
COX7B	4	0.15851	0.3137	0.980024	545	2	0.26661
EDEM1	4	0.15854	0.3138	0.980024	546	2	0.33959
COQ6	4	0.15875	0.31418	0.980024	547	2	0.26061
PLCXD1	4	0.15894	0.31452	0.980024	548	3	0.3002
COQ7	4	0.15896	0.31454	0.980024	549	2	0.23105
CYP2R1	4	0.15949	0.31539	0.980024	550	1	-0.31297
NAALADL1	4	0.15998	0.31613	0.980024	551	1	-0.13586
ETNK1	4	0.15999	0.31614	0.980024	552	2	0.416
HMGCS1	4	0.1602	0.31648	0.980024	553	2	0.24634
SDR16C5	4	0.1604	0.31682	0.980024	554	2	0.097889
ACAA2	4	0.16047	0.31692	0.980024	555	1	-0.44483
NT5M	4	0.16097	0.3178	0.980024	556	1	-0.24095
DPM3	4	0.16107	0.31802	0.980024	557	3	0.26909
IPPK	4	0.16138	0.31861	0.980024	558	3	0.25036
GLUD1	4	0.16144	0.3187	0.980024	559	2	-0.010612
AANAT	4	0.16168	0.31906	0.980024	560	3	0.24356
UGT3A1	4	0.16186	0.31933	0.980024	561	2	0.43873
ACOT11	4	0.16195	0.31946	0.980024	562	2	-0.21236
FUT5	4	0.16207	0.31968	0.980024	563	2	-0.022808
AOAH	4	0.16229	0.31999	0.980024	564	3	0.31288
LYPLA2	4	0.16269	0.32071	0.980024	565	2	-0.48789
FA2H	4	0.16342	0.3219	0.980024	566	2	-0.071082
PLPP2	4	0.16383	0.32267	0.980024	567	3	0.19402
RPIA	4	0.16391	0.32282	0.980024	568	1	0.10109
PANK1	4	0.16478	0.32425	0.980024	569	2	0.4937
AGXT	4	0.16499	0.32459	0.980024	570	2	0.23842
HPDL	4	0.16584	0.32596	0.980024	571	3	0.29006
PIP5KL1	4	0.16615	0.32645	0.980024	572	3	0.15106
PLPP6	4	0.16635	0.32686	0.980024	573	1	-0.070249
PIK3C2A	4	0.16688	0.32731	0.980024	574	2	-0.10411
GGT1	4	0.16782	0.32823	0.980024	575	1	0.047432
XYLB	4	0.16831	0.32874	0.980024	576	2	0.35394
GALM	4	0.16877	0.32921	0.980024	577	2	0.15007
EPHX1	4	0.16928	0.32975	0.980024	578	1	-0.38493

B3GALT4	4	0.1694	0.32989	0.980024	579	2	0.26504
FBP1	4	0.16982	0.33032	0.980024	580	2	0.40725
SDHAF3	4	0.17026	0.33065	0.980024	581	1	-0.13211
PHYHD1	4	0.17046	0.33081	0.980024	582	2	0.02573
PLPP3	4	0.17067	0.33094	0.980024	583	2	0.21008
PIGN	4	0.17236	0.33258	0.980024	584	2	0.3768
ARSD	4	0.17321	0.33351	0.980024	585	2	0.21557
GCNT7	4	0.17366	0.33401	0.980024	586	1	-0.95652
PLA2G1B	4	0.17512	0.33541	0.980024	587	1	0.058653
SC5D	4	0.1756	0.33587	0.980024	588	2	-4.0138
CA11	4	0.17598	0.33628	0.980024	589	2	0.088883
CYP2J2	4	0.17609	0.33635	0.980024	590	1	-0.0061338
IMPA2	4	0.17641	0.33663	0.980024	591	2	0.098772
UCK1	4	0.17657	0.33676	0.980024	592	2	0.11014
ACE	4	0.17705	0.3372	0.980024	593	2	0.21052
PIGH	4	0.17706	0.3372	0.980024	594	2	0.071192
INPP4A	4	0.17754	0.33769	0.980024	595	2	-0.33344
MAOA	4	0.17769	0.33782	0.980024	596	2	0.16841
GNPDA2	4	0.1779	0.33803	0.980024	597	2	0.23347
CHIA	4	0.17899	0.33927	0.980024	598	1	0.14442
PNPLA2	4	0.1794	0.33962	0.980024	599	2	0.26401
TKFC	4	0.17947	0.33969	0.980024	600	2	0.14334
CERS4	4	0.17996	0.34016	0.980024	601	1	-0.4282
DHRS3	4	0.1814	0.34163	0.980024	602	1	-0.30109
ADCY5	4	0.18237	0.34262	0.980024	603	2	0.2835
ALDH4A1	4	0.18414	0.34432	0.980024	604	2	0.025232
CBR3	4	0.18429	0.34443	0.980024	605	1	0.079947
PCYOX1L	4	0.18477	0.34494	0.980024	606	1	-0.68732
INPP5A	4	0.18501	0.34516	0.980024	607	2	-0.15224
FTCD	4	0.18523	0.34534	0.980024	608	2	0.26161
NDUFA6	4	0.18544	0.34554	0.980024	609	2	0.27755
COLGALT1	4	0.18621	0.34618	0.980024	610	1	-0.31663
PLPPR4	4	0.18669	0.34665	0.980024	611	1	-0.20046
CPT1B	4	0.18717	0.34707	0.980024	612	1	-0.22239
PAM	4	0.18718	0.34707	0.980024	613	2	0.24773
DGKB	4	0.18765	0.34751	0.980024	614	1	0.10469
NME4	4	0.18805	0.34785	0.980024	615	2	0.11473
PDE3B	4	0.18813	0.34798	0.980024	616	1	-0.29595
SPHK1	4	0.18827	0.34814	0.980024	617	2	0.22788
HAS1	4	0.18861	0.34845	0.980024	618	2	-0.57434
FN3KRP	4	0.18957	0.34948	0.980024	619	1	-0.65159
NUDT9	4	0.19053	0.35034	0.980024	620	1	-0.026518
CA3	4	0.19132	0.35108	0.980024	621	2	0.48554
GLOD4	4	0.19176	0.35148	0.980024	622	2	-0.012676
MICAL1	4	0.19196	0.35167	0.980024	623	1	-0.1002

PI4K2B	4	0.19241	0.35211	0.980024	624	2	0.24244
ALDH1L2	4	0.19244	0.35214	0.980024	625	1	-0.54855
COQ9	4	0.19307	0.35276	0.980024	626	2	0.28411
MAN1B1	4	0.19373	0.35335	0.980024	627	2	0.36477
CYP2A6	4	0.19387	0.35345	0.980024	628	2	-0.035712
DPYSL5	4	0.19395	0.35352	0.980024	629	2	-0.26141
PNPLA1	4	0.19434	0.35387	0.980024	630	2	0.11962
ST3GAL1	4	0.19593	0.35537	0.980024	631	2	0.22554
NDUFA2	4	0.19625	0.35563	0.980024	632	2	0.311
PLA2G4F	4	0.19672	0.35615	0.980024	633	1	-0.28088
HMOX2	4	0.1972	0.35662	0.980024	634	2	-0.093462
CLYBL	4	0.19767	0.357	0.980024	635	1	0.03774
CYP2W1	4	0.19815	0.35747	0.980024	636	2	0.074965
ACOT2	4	0.19857	0.35789	0.980024	637	2	0.059944
GPX4	4	0.19902	0.35831	0.980024	638	2	-0.071799
SMPDL3B	4	0.1991	0.3584	0.980024	639	1	-0.056061
CBR1	4	0.19946	0.35869	0.980024	640	2	0.13845
DGKZ	4	0.20005	0.35932	0.980024	641	1	-0.32229
UGT2B10	4	0.20034	0.35959	0.980024	642	2	-0.12186
NME2	4	0.20052	0.35972	0.980024	643	2	0.19386
HEXA	4	0.20079	0.35995	0.980024	644	2	0.2639
PLPP4	4	0.20145	0.36056	0.980024	645	2	0.24962
PCBD1	4	0.2019	0.36099	0.980024	646	2	0.40135
DUT	4	0.20194	0.36102	0.980024	647	2	-0.008102
ISYNA1	4	0.20288	0.36189	0.980024	648	1	0.12578
IDH1	4	0.20323	0.36225	0.980024	649	2	0.12506
FAAH	4	0.20412	0.36308	0.980024	650	2	0.22275
NDUFB11	4	0.2043	0.36329	0.980024	651	2	0.060888
GSTM1	4	0.20477	0.36374	0.980024	652	1	-0.024835
GLYATL1	4	0.20635	0.36525	0.980024	653	2	0.26208
HMGCLL1	4	0.20666	0.36546	0.980024	654	1	-0.043376
PIP5K1A	4	0.20807	0.36679	0.980024	655	2	0.029521
NDUFB3	4	0.20901	0.36772	0.980024	656	1	-0.044961
LTC4S	4	0.20948	0.36819	0.980024	657	2	-0.005983 6
PIGU	4	0.21105	0.36983	0.980024	658	2	0.17948
COX4I2	4	0.2124	0.37126	0.980024	659	2	0.12502
PGM1	4	0.21417	0.37285	0.980024	660	1	0.017681
UAP1L1	4	0.2151	0.37382	0.980024	661	1	0.10121
NDUFV2	4	0.21557	0.3742	0.980024	662	1	0.13989
PRDX2	4	0.2165	0.37501	0.980024	663	1	-0.25941
ME3	4	0.21697	0.37551	0.980024	664	1	-0.36782
MOGAT2	4	0.21743	0.37601	0.980024	665	2	0.15438
LIPK	4	0.2179	0.37646	0.980024	666	1	-0.70349
PFKL	4	0.21837	0.37692	0.980024	667	1	0.082849
SGPP2	4	0.21883	0.37743	0.980024	668	1	-0.082201

UGT1A10	4	0.21941	0.37783	0.980024	669	2	0.32443
ADSSL1	4	0.22009	0.37847	0.980024	670	2	0.17747
ALDH5A1	4	0.22054	0.37892	0.980024	671	2	0.058969
CLC	4	0.221	0.37936	0.980024	672	2	0.1879
LPIN1	4	0.22116	0.37953	0.980024	673	1	-0.73639
GLRX	4	0.22209	0.38034	0.980024	674	1	-0.41617
CDA	4	0.22236	0.38059	0.980024	675	2	0.26618
ENPP1	4	0.22348	0.38168	0.980024	676	2	0.093741
CHST5	4	0.22441	0.38245	0.980024	677	2	0.026557
SDR42E1	4	0.22579	0.38393	0.980024	678	1	-0.74429
CHST8	4	0.22624	0.38433	0.980024	679	2	-0.39052
ASPG	4	0.22625	0.38434	0.980024	680	1	-0.14397
RENBP	4	0.22672	0.38488	0.980024	681	2	0.026565
MGST2	4	0.22807	0.38604	0.980024	682	2	0.16965
ADH7	4	0.2283	0.38626	0.980024	683	2	0.38766
PNLIPRP3	4	0.22899	0.38694	0.980024	684	2	0.27667
NUDT16	4	0.22922	0.38722	0.980024	685	2	0.19771
NDUFA13	4	0.2299	0.38787	0.980024	686	2	0.25172
DIO3	4	0.22994	0.38791	0.980024	687	2	0.10528
PDE4B	4	0.23013	0.38813	0.980024	688	2	0.21991
HYAL2	4	0.23105	0.38917	0.980024	689	2	0.19533
OGFOD1	4	0.2322	0.39025	0.980024	690	2	0.17584
RPE	4	0.23289	0.3909	0.980024	691	2	0.27038
EDEM3	4	0.23362	0.39163	0.980024	692	2	-0.17188
ECHS1	4	0.23408	0.39216	0.980024	693	1	0.009954
NDST4	4	0.23454	0.39259	0.980024	694	1	-0.25948
HS3ST1	4	0.23543	0.39338	0.980024	695	2	0.096934
HSD17B10	4	0.23591	0.39398	0.980024	696	2	0.080156
EXTL2	4	0.23728	0.39531	0.980024	697	2	0.069522
ALOXE3	4	0.23774	0.39566	0.980024	698	2	-0.093047
ABHD5	4	0.2382	0.39608	0.980024	699	1	-0.24674
FMO4	4	0.23866	0.39649	0.980024	700	1	-0.43842
NAT14	4	0.2389	0.39677	0.980024	701	2	0.17905
CKM	4	0.23911	0.39698	0.980024	702	2	0.18435
ACADS	4	0.23982	0.39764	0.980024	703	2	0.1973
PLCB4	4	0.24003	0.39787	0.980024	704	1	-0.20913
CYP3A7	4	0.24006	0.39791	0.980024	705	2	-0.36751
CYB5D1	4	0.24052	0.39827	0.980024	706	2	0.12312
GPX1	4	0.24094	0.39862	0.980024	707	2	0.17129
CYP4V2	4	0.24261	0.40025	0.980024	708	2	0.045698
ATP13A1	4	0.24276	0.4004	0.980024	709	2	0.22289
SMPD1	4	0.24321	0.40081	0.980024	710	1	-0.62179
GLUD2	4	0.24412	0.40168	0.980024	711	2	0.18252
IMPDH2	4	0.24424	0.40182	0.980024	712	2	0.12704
COQ10A	4	0.24503	0.40259	0.980024	713	2	-0.095627
UGCG	4	0.24564	0.40307	0.980024	714	2	-1.989

KDSR	4	0.24634	0.4036	0.980024	715	2	0.16908
FUT2	4	0.24774	0.40505	0.980024	716	2	-0.17036
IAH1	4	0.2482	0.4055	0.980024	717	1	-0.014361
UGT1A8	4	0.24844	0.4057	0.980024	718	2	-0.0048615
IDH3A	4	0.24867	0.40595	0.980024	719	2	0.10874
NME5	4	0.25001	0.4071	0.980024	720	1	-0.12455
MDH1B	4	0.25008	0.40715	0.980024	721	2	0.22024
HAGHL	4	0.25031	0.40737	0.980024	722	2	0.25681
BCHE	4	0.25046	0.40751	0.980024	723	1	-0.28748
LPCAT1	4	0.25078	0.40783	0.980024	724	2	0.14775
ADCY10	4	0.25091	0.40796	0.980024	725	2	0.16789
BCKDHB	4	0.25125	0.40828	0.980024	726	2	0.045589
BDH1	4	0.25172	0.40873	0.980024	727	2	0.072165
PYGM	4	0.25181	0.40885	0.980024	728	2	0.24174
CHID1	4	0.25361	0.41054	0.980024	729	2	0.1975
TKTL1	4	0.25618	0.41283	0.980024	730	2	-0.13656
A4GNT	4	0.25631	0.41296	0.980024	731	2	0.09371
HAGH	4	0.25688	0.41352	0.980024	732	2	0.17849
COX7C	4	0.25721	0.41383	0.980024	733	1	0.022062
PHGDH	4	0.2583	0.41484	0.980024	734	2	0.079971
HYAL1	4	0.25853	0.41503	0.980024	735	2	0.0026519
FMO1	4	0.259	0.4155	0.980024	736	1	-0.52363
AMACR	4	0.259	0.4155	0.980024	737	2	0.30748
INPP1	4	0.25924	0.41574	0.980024	738	2	0.1586
PDHX	4	0.26066	0.41703	0.980024	739	2	0.33035
PNPLA3	4	0.26113	0.41755	0.980024	740	2	0.14434
ABAT	4	0.26184	0.41822	0.980024	741	2	-0.036503
NOX1	4	0.26257	0.41879	0.980024	742	1	-0.72217
GMPR2	4	0.26346	0.41965	0.980024	743	2	0.041016
CANT1	4	0.26391	0.42016	0.980024	744	1	-0.30421
FUT10	4	0.26435	0.42059	0.980024	745	2	0.24079
PLCB2	4	0.26515	0.42135	0.980024	746	2	0.12132
ACSS3	4	0.26613	0.42217	0.980024	747	2	0.14635
PRODH	4	0.26657	0.4226	0.980024	748	1	-0.55183
NUDT1	4	0.26702	0.42311	0.980024	749	1	-0.037987
GALNT6	4	0.26791	0.42385	0.980024	750	1	-0.64623
PLCL1	4	0.26835	0.42429	0.980024	751	1	-0.5374
DGKD	4	0.26847	0.42439	0.980024	752	2	0.18433
CYP27A1	4	0.26919	0.42508	0.980024	753	2	0.058701
MSRB2	4	0.26942	0.42537	0.980024	754	2	0.13949
TM7SF2	4	0.26968	0.42559	0.980024	755	1	-0.080895
NDUFS2	4	0.271	0.42679	0.980024	756	2	0.19703
DIO1	4	0.27145	0.42724	0.980024	757	2	0.12165
PTS	4	0.27228	0.42803	0.980024	758	2	0.23063
GALNT1	4	0.27277	0.42841	0.980024	759	1	-0.34997

CES3	4	0.27365	0.42932	0.980024	760	1	-0.60212
GPI	4	0.27371	0.42938	0.980024	761	2	0.099091
B4GALT2	4	0.27395	0.42959	0.980024	762	2	0.029673
GCNT4	4	0.27442	0.43007	0.980024	763	2	0.21758
FUT11	4	0.27497	0.43062	0.980024	764	2	0.043607
PDE9A	4	0.27514	0.43075	0.980024	765	2	0.24243
CES2	4	0.27585	0.43131	0.980024	766	1	-0.11343
OLAH	4	0.2761	0.43155	0.980024	767	2	0.061346
SMPD4	4	0.27673	0.43219	0.980024	768	2	-0.014334
LIPC	4	0.27705	0.43254	0.980024	769	2	0.14278
TYMP	4	0.27761	0.43314	0.980024	770	2	0.13649
ELOVL6	4	0.27825	0.4337	0.980024	771	2	0.15835
COX10	4	0.28024	0.43557	0.980024	772	1	-0.33894
GMPPA	4	0.28112	0.43635	0.980024	773	1	0.0083818
PIK3R3	4	0.28155	0.43673	0.980024	774	1	-0.21791
NDUFB7	4	0.2816	0.4368	0.980024	775	2	0.19148
OXCT1	4	0.28208	0.43719	0.980024	776	2	0.28394
GPX8	4	0.28287	0.43791	0.980024	777	1	-0.11479
CYP26C1	4	0.28374	0.43875	0.980024	778	2	0.042748
PLA2G12A	4	0.28448	0.43953	0.980024	779	2	0.092373
DAO	4	0.28505	0.44003	0.980024	780	1	0.044371
BPGM	4	0.28548	0.44038	0.980024	781	1	-0.54139
IDI2	4	0.28592	0.4408	0.980024	782	1	-0.29217
ACOT4	4	0.28635	0.44123	0.980024	783	1	-0.3512
FECH	4	0.28679	0.44156	0.980024	784	1	-0.10207
COX6A2	4	0.28785	0.44261	0.980024	785	2	0.053908
GSTA4	4	0.28809	0.44284	0.980024	786	1	-0.33727
CES1	4	0.29098	0.44567	0.980024	787	2	0.18197
PDHA1	4	0.29112	0.44581	0.980024	788	2	-0.069647
ACADVL	4	0.29156	0.44622	0.980024	789	2	0.12833
ACYP1	4	0.29242	0.44704	0.980024	790	1	-0.20117
ENOX1	4	0.29285	0.4475	0.980024	791	1	-1.3519
ENTPD3	4	0.29329	0.44785	0.980024	792	1	-0.11867
AMDHD2	4	0.29436	0.449	0.980024	793	2	0.12271
IPMK	4	0.29484	0.44957	0.980024	794	2	-0.95381
ACLY	4	0.29501	0.44969	0.980024	795	2	-0.30086
ACADL	4	0.29629	0.45086	0.980024	796	2	0.20888
MUT	4	0.29653	0.45106	0.980024	797	2	0.16729
GYG2	4	0.29702	0.4515	0.980024	798	2	0.12047
AKR1C4	4	0.29716	0.45166	0.980024	799	1	-0.055629
PYCR3	4	0.29847	0.45299	0.980024	800	2	0.070041
PLA2G2A	4	0.29888	0.45339	0.980024	801	1	-0.63629
CA6	4	0.29944	0.45399	0.980024	802	2	-0.004117
GMDS	4	0.29974	0.45429	0.980024	803	1	-0.11411
NT5C3B	4	0.29992	0.45448	0.980024	804	2	0.11909
NMNAT1	4	0.30016	0.45472	0.980024	805	2	-0.20697

ACO2	4	0.30145	0.45592	0.980024	806	1	0.065229
PDSS2	4	0.30188	0.4564	0.980024	807	1	0.088021
DDAH1	4	0.30235	0.45682	0.980024	808	2	0.26826
ACSL3	4	0.3038	0.45814	0.980024	809	2	0.35029
IP6K1	4	0.30402	0.45833	0.980024	810	2	0.08046
SQOR	4	0.30487	0.45923	0.980024	811	2	0.20795
LIPE	4	0.30502	0.45937	0.980024	812	2	0.27655
HAO2	4	0.30573	0.46008	0.980024	813	2	0.052978
MSRA	4	0.30696	0.46111	0.980024	814	2	0.24571
PIK3R5	4	0.30785	0.46198	0.980024	815	2	0.20169
APEH	4	0.30891	0.463	0.980024	816	2	0.12023
COASY	4	0.3094	0.4635	0.980024	817	2	0.33328
GAL3ST3	4	0.30955	0.46365	0.980024	818	2	0.21387
COQ3	4	0.30988	0.464	0.980024	819	2	0.10163
MGAT2	4	0.30998	0.4641	0.980024	820	1	-0.049875
PPCDC	4	0.31013	0.46424	0.980024	821	2	0.21595
PIK3C3	4	0.31037	0.46445	0.980024	822	2	0.027453
INPP5E	4	0.31061	0.46467	0.980024	823	2	0.20893
FAH	4	0.31125	0.46533	0.980024	824	1	-0.16679
PLCZ1	4	0.31232	0.46635	0.980024	825	2	-0.03747
KYAT3	4	0.31252	0.46656	0.980024	826	1	-0.1867
ALG13	4	0.31305	0.46712	0.980024	827	2	0.21053
GAL3ST1	4	0.31336	0.46748	0.980024	828	1	-0.062968
ALDH3B2	4	0.31403	0.46811	0.980024	829	2	0.26768
FUT3	4	0.31463	0.46859	0.980024	830	1	-1.281
MME	4	0.31505	0.46898	0.980024	831	1	-0.086676
SCD5	4	0.31525	0.46911	0.980024	832	2	0.1703
PIK3C2G	4	0.31547	0.46927	0.980024	833	2	0.17994
LDHB	4	0.31549	0.46928	0.980024	834	2	0.31631
PIGK	4	0.31574	0.4695	0.980024	835	2	0.25755
GGTLC1	4	0.3159	0.46962	0.980024	836	2	-0.086256
PLA2G12B	4	0.31598	0.46971	0.980024	837	2	0.11568
B3GAT1	4	0.31758	0.47143	0.980024	838	2	0.12388
CYB5R2	4	0.31794	0.47171	0.980024	839	2	0.23242
ETFA	4	0.318	0.47177	0.980024	840	1	-0.38619
CHST11	4	0.31926	0.47284	0.980024	841	1	0.054999
SULT1E1	4	0.3194	0.47294	0.980024	842	2	0.17907
EBPL	4	0.31968	0.47317	0.980024	843	1	-0.93826
ALG9	4	0.3201	0.47356	0.980024	844	1	-0.20766
TKTL2	4	0.32014	0.4736	0.980024	845	2	-0.34936
CYP2B6	4	0.32052	0.47409	0.980024	846	2	0.095394
GPD1	4	0.32063	0.47421	0.980024	847	2	0.20474
SMPD3	4	0.32261	0.47616	0.980024	848	2	-0.22668
UGT1A5	4	0.3243	0.47784	0.980024	849	2	-0.0071893
ADPGK	4	0.32512	0.4785	0.980024	850	1	0.10589

CYP4F3	4	0.32602	0.47927	0.980024	851	2	-0.17889
UCKL1	4	0.32651	0.4797	0.980024	852	2	-0.25437
DGKA	4	0.3272	0.48046	0.980024	853	1	0.10074
PLCE1	4	0.32872	0.48182	0.980024	854	2	0.078569
HDC	4	0.32896	0.48209	0.980024	855	2	0.2519
CTPS1	4	0.33044	0.48351	0.980024	856	2	0.23865
MMAB	4	0.33301	0.48602	0.980024	857	1	-0.83026
NME6	4	0.33314	0.4862	0.980024	858	2	0.17342
MSRB1	4	0.33363	0.48664	0.980024	859	2	-0.0046794
PCK2	4	0.33384	0.48682	0.980024	860	2	0.16172
CYB5D2	4	0.33466	0.48769	0.980024	861	1	-0.04844
BPHL	4	0.33508	0.48814	0.980024	862	1	-0.18278
COX16	4	0.33755	0.49021	0.980024	863	2	0.082285
ST8SIA3	4	0.33757	0.49024	0.980024	864	2	-0.2387
CRYZ	4	0.33806	0.49072	0.980024	865	2	-0.37271
B4GALNT1	4	0.33837	0.49099	0.980024	866	1	-0.74417
HSD17B8	4	0.3396	0.49214	0.980024	867	2	0.16769
CS	4	0.34028	0.49275	0.980024	868	2	0.20306
ACSBG2	4	0.34083	0.49314	0.980024	869	1	-0.41476
UGT1A9	4	0.34288	0.49522	0.980024	870	2	-0.56256
DHRSX	4	0.34349	0.49578	0.980024	871	2	-0.069185
GCLM	4	0.34398	0.4962	0.980024	872	2	-0.018092
CA4	4	0.34423	0.49648	0.980024	873	2	0.21887
PGM5	4	0.34451	0.49676	0.980024	874	1	-0.071787
SRD5A1	4	0.34571	0.49806	0.980024	875	2	0.19072
LYZL4	4	0.34655	0.49888	0.980024	876	1	-0.65152
ALDH2	4	0.34696	0.49929	0.980024	877	2	-0.0029976
ALOX15B	4	0.34719	0.49953	0.980024	878	2	-0.0088528
NPR1	4	0.34744	0.49975	0.980024	879	2	-0.072304
ABHD2	4	0.34842	0.50069	0.980024	880	2	0.23298
ARSJ	4	0.3504	0.50255	0.980024	881	2	-0.087492
SULT4A1	4	0.35142	0.50342	0.980024	882	1	0.024621
LGSN	4	0.35183	0.50384	0.980024	883	1	-0.66939
MAN1C1	4	0.35188	0.50389	0.980024	884	2	0.26832
BDH2	4	0.35304	0.50487	0.980024	885	1	0.044026
PIGB	4	0.35362	0.50544	0.980024	886	2	-0.40574
ENO3	4	0.35385	0.50565	0.980024	887	1	-0.55045
BLVRB	4	0.35485	0.50647	0.980024	888	2	0.070824
GUSB	4	0.35506	0.5067	0.980024	889	1	-0.1239
ETFDH	4	0.35535	0.50698	0.980024	890	2	-0.20367
HSD17B13	4	0.35584	0.50745	0.980024	891	2	0.22688
MPO	4	0.35627	0.50786	0.980024	892	2	-0.28702
LDHD	4	0.35659	0.50817	0.980024	893	2	0.012378

GDPD4	4	0.35667	0.50825	0.980024	894	1	-0.2041
GK	4	0.35733	0.50884	0.980024	895	2	-0.091484
GSTCD	4	0.35747	0.50897	0.980024	896	1	0.11088
NME7	4	0.35828	0.50968	0.980024	897	1	-0.15263
GSTZ1	4	0.35857	0.50997	0.980024	898	2	0.22924
GYG1	4	0.35868	0.51008	0.980024	899	1	-0.38487
ACADSB	4	0.35908	0.51053	0.980024	900	2	-0.016485
PHOSPHO1	4	0.35956	0.51096	0.980024	901	2	0.093461
PRPS1L1	4	0.3598	0.51129	0.980024	902	2	0.03427
HS6ST1	4	0.3608	0.51229	0.980024	903	2	-0.013578
GPLD1	4	0.36104	0.51253	0.980024	904	2	0.28493
FHIT	4	0.36109	0.51261	0.980024	905	1	0.095666
IVD	4	0.36229	0.51374	0.980024	906	2	-0.081346
CYP21A2	4	0.36309	0.51456	0.980024	907	1	-0.36251
DDOST	4	0.36327	0.51479	0.980024	908	2	0.14552
G6PC	4	0.36388	0.51538	0.980024	909	1	-0.70555
AKR1C2	4	0.36402	0.51554	0.980024	910	2	0.45429
GLS	4	0.36428	0.51579	0.980024	911	1	0.032272
ACHE	4	0.36588	0.51743	0.980024	912	2	-0.16566
PLA2G15	4	0.36675	0.51838	0.980024	913	2	0.056331
CYP4X1	4	0.36724	0.51882	0.980024	914	2	-0.18018
CPT2	4	0.36848	0.51998	0.980024	915	2	0.090251
ACYP2	4	0.36866	0.52021	0.980024	916	1	-0.050466
PIK3CA	4	0.36972	0.52133	0.980024	917	2	-0.0045612
INPP4B	4	0.37024	0.52181	0.980024	918	1	-0.82014
UMPS	4	0.37064	0.5222	0.980024	919	1	-0.35829
NAT1	4	0.3732	0.52468	0.980024	920	2	0.14626
RDH13	4	0.37341	0.52486	0.980024	921	2	-0.0083837
ABHD4	4	0.37345	0.52487	0.980024	922	2	-0.30566
ADO	4	0.37419	0.52552	0.980024	923	2	-0.017846
PLCB3	4	0.3742	0.52553	0.980024	924	1	-0.40193
MTHFD1L	4	0.37498	0.52625	0.980024	925	1	-0.13071
SCLY	4	0.37538	0.52657	0.980024	926	1	-0.015083
SAT1	4	0.37593	0.52706	0.980024	927	2	0.24081
GLT8D1	4	0.37618	0.52738	0.980024	928	2	0.20786
GLYAT	4	0.37656	0.52773	0.980024	929	1	-0.38826
ATP13A2	4	0.38009	0.531	0.980024	930	1	-0.51103
PPAT	4	0.38048	0.53146	0.980024	931	1	-0.41654
NOX5	4	0.38065	0.53166	0.980024	932	2	0.1295
PLCB1	4	0.3809	0.53188	0.980024	933	2	-0.013908
DDAH2	4	0.38126	0.53219	0.980024	934	2	0.163
GNPTG	4	0.38204	0.53298	0.980024	935	1	-0.34342
PLCD4	4	0.38243	0.53334	0.980024	936	1	-0.13157
DHRS7	4	0.38289	0.53369	0.980024	937	2	0.24748

FDXR	4	0.38321	0.534	0.980024	938	1	-0.5205
GALNT13	4	0.38339	0.53415	0.980024	939	2	0.18757
GALNT7	4	0.38438	0.53505	0.980024	940	2	-0.029011
FUT7	4	0.38463	0.53531	0.980024	941	2	0.16884
CYP2F1	4	0.38538	0.53598	0.980024	942	2	-0.31091
CYCS	4	0.38588	0.53639	0.980024	943	2	0.13179
UGP2	4	0.38633	0.53682	0.980024	944	1	-0.75839
ARSF	4	0.38904	0.53933	0.980024	945	2	-0.16239
OAT	4	0.39011	0.54033	0.980024	946	2	-0.055882
GNMT	4	0.39036	0.54059	0.980024	947	2	-0.017076
ALDH9A1	4	0.3911	0.54129	0.980024	948	2	-0.025843
RDH5	4	0.39136	0.54157	0.980024	949	2	0.099391
B3GNT5	4	0.39235	0.54247	0.980024	950	2	0.013139
MGST1	4	0.39359	0.54372	0.980024	951	2	0.22606
NOX4	4	0.39367	0.54378	0.980024	952	1	0.15561
PDE11A	4	0.39409	0.54421	0.980024	953	2	0.18479
MSMO1	4	0.39434	0.54442	0.980024	954	2	-0.38882
HS6ST3	4	0.39444	0.54451	0.980024	955	1	0.17803
NAT8B	4	0.39583	0.54573	0.980024	956	2	0.11604
PTDSS2	4	0.39608	0.54597	0.980024	957	2	-0.25232
NUDT10	4	0.39633	0.54616	0.980024	958	2	-0.039265
DDT	4	0.39636	0.5462	0.980024	959	2	-0.004344
B3GAT2	4	0.39658	0.5464	0.980024	960	2	0.097452
NAGA	4	0.39683	0.5466	0.980024	961	2	0.091887
PRPSAP2	4	0.39708	0.54685	0.980024	962	2	-0.24676
DBH	4	0.39828	0.54807	0.980024	963	2	0.14544
ODC1	4	0.39832	0.54811	0.980024	964	2	0.15586
G6PC2	4	0.39857	0.54838	0.980024	965	2	-0.070488
GPX5	4	0.39956	0.54944	0.980024	966	2	0.15808
NUDT4	4	0.40057	0.55032	0.980024	967	1	-0.46801
ABHD12B	4	0.40081	0.55055	0.980024	968	2	0.15869
PDHB	4	0.40133	0.55113	0.980024	969	1	-0.25098
HKDC1	4	0.40172	0.55145	0.980024	970	1	-0.033245
ABHD1	4	0.40205	0.55181	0.980024	971	2	0.15363
SDR9C7	4	0.40248	0.55221	0.980024	972	1	-0.25914
AFMID	4	0.4028	0.55252	0.980024	973	2	0.02996
PMM1	4	0.40324	0.55287	0.980024	974	1	-0.44112
ADH1B	4	0.404	0.55354	0.980024	975	1	-0.47783
RBKS	4	0.40405	0.5536	0.980024	976	2	0.18378
HGSNAT	4	0.40454	0.55408	0.980024	977	2	-0.10785
ACOT6	4	0.40476	0.55432	0.980024	978	1	-0.10668
KHK	4	0.40479	0.55436	0.980024	979	2	0.17546
ADCY3	4	0.40529	0.55495	0.980024	980	2	0.14914
ADCY9	4	0.40579	0.5555	0.980024	981	2	0.18065
ACSL1	4	0.40628	0.55595	0.980024	982	2	-0.0097534

GPD2	4	0.40703	0.55669	0.980024	983	2	0.1224
TXNRD2	4	0.40741	0.55704	0.980024	984	2	0.16344
CYP2C18	4	0.40753	0.55716	0.980024	985	2	-0.23051
GSTA2	4	0.40817	0.55776	0.980024	986	2	-0.14663
GLA	4	0.40828	0.55786	0.980024	987	2	0.13195
GALNT17	4	0.40892	0.55841	0.980024	988	1	-0.3659
ACBD3	4	0.4093	0.55878	0.980024	989	1	-0.53964
SDHA	4	0.40952	0.559	0.980024	990	2	0.0067878
UGT2A1	4	0.41002	0.55942	0.980024	991	2	-0.032526
ENTPD8	4	0.41081	0.56021	0.980024	992	1	-0.10582
ALDH3A2	4	0.41102	0.56045	0.980024	993	2	-0.080889
LSS	4	0.41119	0.56068	0.980024	994	1	-0.43465
TH	4	0.41201	0.56142	0.980024	995	2	0.022267
TST	4	0.41269	0.56217	0.980024	996	1	-0.68182
RRM2	4	0.41326	0.56267	0.980024	997	2	-0.18306
CP	4	0.41382	0.56315	0.980024	998	2	0.036996
CRYM	4	0.41419	0.56351	0.980024	999	1	-0.098595
LPIN2	4	0.41525	0.56457	0.980024	1000	2	-0.066085
FLAD1	4	0.416	0.5652	0.980024	1001	2	0.12757
ADAD2	4	0.41682	0.56599	0.980024	1002	2	-0.18637
ADA	4	0.41699	0.56623	0.980024	1003	2	-0.46618
MGAT4C	4	0.41719	0.56647	0.980024	1004	1	-0.37877
SGPP1	4	0.41724	0.56651	0.980024	1005	2	0.048632
ACSS2	4	0.41756	0.56684	0.980024	1006	1	-0.23337
HSD3B2	4	0.41824	0.56751	0.980024	1007	2	0.23371
MARC1	4	0.41831	0.56759	0.980024	1008	1	-0.35977
PTGS1	4	0.41849	0.56777	0.980024	1009	2	0.076108
MMEL1	4	0.41898	0.56829	0.980024	1010	2	-0.038141
PLA2G16	4	0.42017	0.56947	0.980024	1011	1	-0.35651
DGUOK	4	0.42023	0.56951	0.980024	1012	2	0.058127
HS3ST2	4	0.42048	0.56982	0.980024	1013	2	-0.16251
PIGL	4	0.42098	0.57036	0.980024	1014	2	0.08438
CRYL1	4	0.42222	0.57158	0.980024	1015	2	0.13958
LIPM	4	0.42352	0.57277	0.980024	1016	1	-0.19198
ACSL6	4	0.42389	0.57321	0.980024	1017	2	0.094701
OAS3	4	0.42421	0.5736	0.980024	1018	2	-0.51418
HSD3B1	4	0.42426	0.57364	0.980024	1019	1	-0.38967
GCAT	4	0.42471	0.57412	0.980024	1020	2	-0.17439
FUT8	4	0.425	0.57438	0.980024	1021	1	-0.54263
HS3ST4	4	0.42537	0.57476	0.980024	1022	2	0.16635
MANBAL	4	0.42684	0.57625	0.980024	1023	1	0.039763
TXNRD1	4	0.42745	0.57677	0.980024	1024	2	0.25236
CERS2	4	0.42795	0.57731	0.980024	1025	1	-0.16188
ARSA	4	0.42894	0.57835	0.980024	1026	2	-0.028518
AGA	4	0.42942	0.57886	0.980024	1027	1	-0.084326
FASN	4	0.43043	0.57989	0.980024	1028	2	-0.088244

GALK2	4	0.43118	0.58058	0.980024	1029	2	0.14767
B4GALT7	4	0.43126	0.58068	0.980024	1030	1	0.11997
DHRS4L2	4	0.43167	0.58118	0.980024	1031	2	0.12342
GAD2	4	0.43192	0.58144	0.980024	1032	2	0.0057604
BLVRA	4	0.43273	0.58219	0.980024	1033	1	-0.56894
NIT1	4	0.43292	0.5824	0.980024	1034	2	0.095288
MANEA	4	0.43309	0.58259	0.980024	1035	1	-0.20901
PLCH2	4	0.43382	0.5833	0.980024	1036	2	0.13258
GRXCR2	4	0.43391	0.58342	0.980024	1037	2	0.027025
PNPLA6	4	0.43441	0.58394	0.980024	1038	2	0.20396
SDSL	4	0.43492	0.58428	0.980024	1039	1	-0.070952
HS6ST2	4	0.43565	0.58499	0.980024	1040	2	-0.012468
UGT3A2	4	0.43601	0.58525	0.980024	1041	1	-0.22296
IMPDH1	4	0.43615	0.58542	0.980024	1042	2	0.084214
G6PC3	4	0.4382	0.58736	0.980024	1043	1	-0.42178
CHKA	4	0.43856	0.58763	0.980024	1044	2	0.070245
GSTO2	4	0.44074	0.58976	0.980024	1045	1	-0.77507
ITPKA	4	0.44087	0.58992	0.980024	1046	2	0.1033
PAPSS1	4	0.4411	0.59013	0.980024	1047	1	-0.74559
PGM2	4	0.44137	0.5904	0.980024	1048	2	-0.043791
SCCPDH	4	0.44162	0.59067	0.980024	1049	2	-0.73621
FUCA1	4	0.44236	0.59133	0.980024	1050	2	0.17276
HS2ST1	4	0.44261	0.59163	0.980024	1051	2	-0.52742
DGKH	4	0.44291	0.592	0.980024	1052	1	-0.42537
AKR1A1	4	0.4436	0.59267	0.980024	1053	2	0.064012
CDS1	4	0.4441	0.59308	0.980024	1054	2	-0.25317
CYBA	4	0.44471	0.59363	0.980024	1055	1	-0.018887
AMPD3	4	0.44484	0.59376	0.980024	1056	2	0.23172
BHMT2	4	0.44687	0.5959	0.980024	1057	2	0.1664
CDO1	4	0.44757	0.59645	0.980024	1058	2	-0.021798
SRR	4	0.44881	0.5977	0.980024	1059	2	0.065378
ARSI	4	0.44903	0.59786	0.980024	1060	1	-0.39594
ABHD6	4	0.44906	0.59791	0.980024	1061	2	-0.069865
ARG1	4	0.44938	0.59815	0.980024	1062	1	-0.071241
PLCD3	4	0.45005	0.59883	0.980024	1063	2	0.12595
L2HGDH	4	0.4501	0.59887	0.980024	1064	1	-0.33151
CERS6	4	0.4503	0.59903	0.980024	1065	2	0.097971
UGT1A4	4	0.45081	0.59951	0.980024	1066	1	-0.18282
LIAS	4	0.45129	0.6	0.980024	1067	2	-0.10605
ADH1A	4	0.45153	0.60023	0.980024	1068	1	-0.55523
FAR1	4	0.45154	0.60025	0.980024	1069	2	0.12618
COMT	4	0.45179	0.60051	0.980024	1070	2	0.14501
IDI1	4	0.45229	0.60095	0.980024	1071	2	0.081337
PRDX4	4	0.45328	0.60214	0.980024	1072	2	0.037173
ME2	4	0.45367	0.60255	0.980024	1073	1	-0.29449
PIGF	4	0.45427	0.60313	0.980024	1074	2	-0.36662

PGK2	4	0.4565	0.60528	0.980024	1075	2	0.16182
GALNT15	4	0.45722	0.60593	0.980024	1076	2	-0.066507
CA1	4	0.45724	0.60594	0.980024	1077	2	-0.40163
SRD5A3	4	0.45774	0.60635	0.980024	1078	2	0.11764
CYBRD1	4	0.45823	0.60685	0.980024	1079	2	-0.11293
ACSM2A	4	0.45899	0.60765	0.980024	1080	1	-0.0564
PCYT1B	4	0.45922	0.60785	0.980024	1081	2	0.11379
UGT2B7	4	0.45972	0.60838	0.980024	1082	2	0.42557
CYP7B1	4	0.45997	0.60856	0.980024	1083	2	0.041667
CUBN	4	0.46096	0.60962	0.980024	1084	2	0.092716
TPK1	4	0.4612	0.60988	0.980024	1085	2	-0.067104
SRM	4	0.46145	0.61009	0.980024	1086	2	0.090181
PRG2	4	0.46146	0.6101	0.980024	1087	1	-0.67008
H6PD	4	0.4617	0.6103	0.980024	1088	2	0.008587
CKB	4	0.46181	0.6104	0.980024	1089	1	0.12891
MDH1	4	0.46393	0.61244	0.980024	1090	2	0.0279
CYP3A5	4	0.46427	0.61278	0.980024	1091	1	-1.1503
QDPR	4	0.46516	0.61372	0.980024	1092	2	-0.30647
PLB1	4	0.46541	0.61402	0.980024	1093	2	0.077876
NUDT11	4	0.4659	0.61441	0.980024	1094	2	-0.20377
GATM	4	0.46602	0.61458	0.980024	1095	1	-0.021717
GCLC	4	0.46615	0.61469	0.980024	1096	2	0.25334
ACSL4	4	0.46637	0.61494	0.980024	1097	1	-0.33183
ADCY4	4	0.4664	0.61496	0.980024	1098	2	0.1018
ENPP4	4	0.46714	0.61564	0.980024	1099	2	0.10508
EBP	4	0.46739	0.61591	0.980024	1100	2	0.031599
UGDH	4	0.46742	0.61594	0.980024	1101	1	-0.067789
GMPR	4	0.46812	0.61662	0.980024	1102	1	-0.27023
CHSY1	4	0.4701	0.6185	0.980024	1103	2	-0.52459
DGKE	4	0.47021	0.61861	0.980024	1104	1	-0.34013
DGKI	4	0.4709	0.61922	0.980024	1105	1	0.031909
INMT	4	0.47159	0.61981	0.980024	1106	2	0.17179
MPI	4	0.47195	0.6201	0.980024	1107	1	-0.02747
B3GNT7	4	0.47299	0.62115	0.980024	1108	1	-0.26521
GAD1	4	0.47331	0.62149	0.980024	1109	2	0.065105
PC	4	0.47472	0.62275	0.980024	1110	1	0.039637
INPP5D	4	0.47479	0.62283	0.980024	1111	2	-0.029797
GUCY1B3	4	0.47504	0.62301	0.980024	1112	2	0.18412
GLYATL2	4	0.47575	0.62373	0.980024	1113	1	-0.50336
SYNJ1	4	0.47603	0.62398	0.980024	1114	2	0.016007
HSD11B1L	4	0.47627	0.62417	0.980024	1115	2	0.17361
SCP2	4	0.47644	0.6243	0.980024	1116	1	-0.6537
COX6B2	4	0.47679	0.6247	0.980024	1117	1	-0.13808
ST6GALNAC1	4	0.47782	0.6258	0.980024	1118	1	-0.28149
IDH2	4	0.47874	0.62671	0.980024	1119	2	-0.028407
HS3ST6	4	0.47923	0.62719	0.980024	1120	2	0.093385

A4GALT	4	0.48071	0.6286	0.980024	1121	2	0.21868
HPD	4	0.48095	0.62887	0.980024	1122	2	-0.42686
UGT2B4	4	0.48194	0.62988	0.980024	1123	1	-0.23289
DHTKD1	4	0.48219	0.63012	0.980024	1124	2	0.16283
CYP4A11	4	0.48262	0.63057	0.980024	1125	1	-0.29852
PI4KB	4	0.48296	0.63089	0.980024	1126	1	-0.32647
PFKFB1	4	0.48317	0.63115	0.980024	1127	2	0.16031
CYP19A1	4	0.48331	0.63128	0.980024	1128	1	0.0013971
CEPT1	4	0.48342	0.63138	0.980024	1129	2	-0.37066
OGFOD2	4	0.48365	0.63169	0.980024	1130	1	-0.054786
HAS2	4	0.48399	0.63203	0.980024	1131	1	-0.29346
NEU1	4	0.48465	0.63254	0.980024	1132	2	-0.059023
AGPAT5	4	0.48467	0.63256	0.980024	1133	1	0.050345
PHYH	4	0.48501	0.63286	0.980024	1134	1	-0.30823
FUK	4	0.48563	0.63341	0.980024	1135	2	0.077657
UROC1	4	0.48735	0.63495	0.980024	1136	2	-0.15496
MGST3	4	0.48784	0.63541	0.980024	1137	2	-0.44673
DGAT2	4	0.48807	0.63564	0.980024	1138	1	0.11041
B4GALT1	4	0.48809	0.63565	0.980024	1139	2	0.054976
PIP4K2C	4	0.48833	0.63599	0.980024	1140	2	0.15504
GAMT	4	0.48875	0.63631	0.980024	1141	1	-0.13101
ENO1	4	0.48976	0.63714	0.980024	1142	1	-0.79372
ALAD	4	0.49103	0.63842	0.980024	1143	2	0.063198
NDUFAB1	4	0.49128	0.63861	0.980024	1144	2	0.20645
GLS2	4	0.49177	0.6391	0.980024	1145	2	0.17955
CES5A	4	0.49226	0.63968	0.980024	1146	2	0.15147
PDE6D	4	0.49251	0.63999	0.980024	1147	2	0.18386
ACADM	4	0.493	0.64046	0.980024	1148	2	0.17536
PLA2G4C	4	0.49347	0.64097	0.980024	1149	1	-0.041036
PDE10A	4	0.49422	0.64175	0.980024	1150	2	-0.12914
PTGR1	4	0.49482	0.64231	0.980024	1151	1	0.022266
PIK3R6	4	0.49549	0.64307	0.980024	1152	1	-1.2297
UPP2	4	0.49569	0.6432	0.980024	1153	2	0.020032
IMPAD1	4	0.49582	0.64333	0.980024	1154	1	-1.4201
PCYT1A	4	0.49618	0.64369	0.980024	1155	2	0.084901
ADCY8	4	0.49692	0.64444	0.980024	1156	2	0.092711
ACER1	4	0.4975	0.64514	0.980024	1157	1	-0.53893
UQCRH	4	0.49783	0.64553	0.980024	1158	1	-1.853
RDH10	4	0.49814	0.64587	0.980024	1159	2	0.12067
B3GNT9	4	0.49817	0.64587	0.980024	1160	1	-0.19179
PDE6B	4	0.49912	0.64686	0.980024	1161	2	0.11934
AGL	4	0.49917	0.6469	0.980024	1162	1	-0.22972
ABHD14B	4	0.4995	0.64723	0.980024	1163	1	-0.2641
ETNPPL	4	0.49961	0.64731	0.980024	1164	2	0.15524
ADK	4	0.49983	0.6475	0.980024	1165	1	0.030451
ACAT2	4	0.5001	0.64779	0.980024	1166	2	0.16831

MGAT3	4	0.50181	0.64946	0.980024	1167	2	-0.76742
HMGCR	4	0.50216	0.64986	0.980024	1168	1	-4.0223
ARSH	4	0.5023	0.64998	0.980024	1169	2	0.10932
ALOX5	4	0.50316	0.65078	0.980024	1170	1	0.070666
B4GAT1	4	0.50328	0.6509	0.980024	1171	2	0.023669
NUDT2	4	0.50352	0.65112	0.980024	1172	2	0.0024639
MTHFSD	4	0.50377	0.65137	0.980024	1173	2	-0.068038
DDHD1	4	0.50481	0.6524	0.980024	1174	1	-0.036188
ST8SIA1	4	0.50712	0.65346	0.980024	1175	1	-0.1281
PCK1	4	0.50943	0.65435	0.980024	1176	1	0.019281
CYP2C8	4	0.51238	0.65552	0.980024	1177	1	-0.10049
HNMT	4	0.51368	0.65595	0.980024	1178	1	-0.19594
GLOD5	4	0.51433	0.65619	0.980024	1179	1	-0.3792
CERKL	4	0.51726	0.6572	0.980024	1180	1	-0.14754
PGLS	4	0.51856	0.65775	0.980024	1181	1	0.025428
PIPOX	4	0.51953	0.65816	0.980024	1182	1	-0.10496
SULT2A1	4	0.52243	0.65923	0.980024	1183	1	-0.21763
ALDH1L1	4	0.52468	0.66013	0.980024	1184	1	0.13021
FRRS1	4	0.525	0.66026	0.980024	1185	1	-0.55907
RRM1	4	0.52564	0.66058	0.980024	1186	1	-0.56305
MLYCD	4	0.52756	0.66132	0.980024	1187	1	-0.034789
ADSS	4	0.52788	0.66144	0.980024	1188	1	-0.36233
PNPLA7	4	0.53011	0.66225	0.980024	1189	1	-0.086896
SGMS1	4	0.53233	0.66302	0.980024	1190	1	-0.2105
DEGS1	4	0.53265	0.66313	0.980024	1191	1	-0.58354
B3GALNT2	4	0.5336	0.66348	0.980024	1192	1	0.058932
SIAE	4	0.53423	0.66377	0.980024	1193	1	-0.98736
FXN	4	0.53738	0.66502	0.980024	1194	1	-0.35602
ENOPH1	4	0.53769	0.66513	0.980024	1195	1	-0.24952
ABHD10	4	0.53863	0.66551	0.980024	1196	1	0.073532
PLA2G2E	4	0.53895	0.66565	0.980024	1197	1	-0.32477
FUCA2	4	0.53957	0.6659	0.980024	1198	1	-0.1165
ENPP5	4	0.5427	0.66712	0.980024	1199	1	-0.44297
MBOAT4	4	0.54611	0.66851	0.980024	1200	1	-0.68297
HPGD	4	0.54859	0.66956	0.980024	1201	1	-0.10998
STS	4	0.5489	0.66973	0.980024	1202	1	-0.31712
ACOT9	4	0.55074	0.67045	0.980024	1203	1	0.010473
GOT2	4	0.55197	0.67093	0.980024	1204	1	0.058082
PAOX	4	0.55228	0.67105	0.980024	1205	1	-0.39888
NDUFA7	4	0.55259	0.67121	0.980024	1206	1	-0.2251
AACS	4	0.55381	0.67173	0.980024	1207	1	-0.20064
ENPP7	4	0.55473	0.67216	0.980024	1208	1	-0.42092
PIGG	4	0.55564	0.67254	0.980024	1209	1	-0.88322
CYP26B1	4	0.55595	0.6727	0.980024	1210	1	-0.72884
DOLPP1	4	0.55656	0.67301	0.980024	1211	1	0.20587
BAAT	4	0.55899	0.67414	0.980024	1212	1	-0.043914

CHIT1	4	0.56111	0.6751	0.980024	1213	1	-0.21817
CHI3L1	4	0.56261	0.67573	0.980024	1214	1	-1.3374
PIK3CB	4	0.56382	0.67623	0.980024	1215	1	-0.51337
ALG8	4	0.56622	0.6772	0.980024	1216	1	-0.30143
LRAT	4	0.56801	0.6781	0.980024	1217	1	-0.31079
MAN1A2	4	0.56891	0.67846	0.980024	1218	1	-0.40722
COMTD1	4	0.56921	0.67864	0.980024	1219	1	0.099857
SYNJ2	4	0.57158	0.67967	0.980024	1220	1	-0.12226
C1GALT1	4	0.57336	0.68046	0.980024	1221	1	-0.65398
ALDH1A2	4	0.57425	0.68083	0.980024	1222	1	0.0025076
PLCXD2	4	0.57778	0.68227	0.980024	1223	1	-0.49456
LYZL6	4	0.57807	0.68241	0.980024	1224	1	-0.26543
B3GNT2	4	0.57866	0.68266	0.980024	1225	1	0.022855
CNP	4	0.57983	0.68316	0.980024	1226	1	-0.28792
SARDH	4	0.581	0.6837	0.980024	1227	1	0.11479
PLCG1	4	0.58187	0.68403	0.980024	1228	1	-0.44914
PTGS2	4	0.58304	0.68453	0.980024	1229	1	-1.0147
GNPDA1	4	0.58362	0.68482	0.980024	1230	1	-0.087601
PDE6A	4	0.58565	0.68577	0.980024	1231	1	0.036731
GSTM2	4	0.58594	0.68589	0.980024	1232	1	-0.28021
GLO1	4	0.58738	0.68656	0.980024	1233	1	-0.235
CYB5RL	4	0.58767	0.68669	0.980024	1234	1	-0.31978
PLCG2	4	0.58911	0.68735	0.980024	1235	1	-0.058098
DHFR2	4	0.5934	0.68922	0.980024	1236	1	-0.82639
UGT1A7	4	0.59369	0.68932	0.980024	1237	1	-0.22063
GLRX5	4	0.59426	0.6895	0.980024	1238	1	-0.1048
GCK	4	0.59454	0.68965	0.980024	1239	1	-0.06333
SOAT1	4	0.59511	0.6899	0.980024	1240	1	-0.18006
FDFT1	4	0.59851	0.69138	0.980024	1241	1	-0.77756
ACSM1	4	0.59908	0.69162	0.980024	1242	1	0.092822
SHMT2	4	0.59936	0.69176	0.980024	1243	1	-0.029611
SMPDL3A	4	0.60274	0.69321	0.980024	1244	1	0.087283
NUDT22	4	0.60441	0.694	0.980024	1245	1	-0.041769
KMO	4	0.60859	0.69597	0.980024	1246	1	-0.5707
GFPT2	4	0.61191	0.69753	0.980024	1247	1	0.11146
RETSAT	4	0.61356	0.69833	0.980024	1248	1	-0.5494
GNE	4	0.61493	0.69888	0.980024	1249	1	-0.072867
TKT	4	0.6152	0.69902	0.980024	1250	1	0.011072
AOC1	4	0.61575	0.69927	0.980024	1251	1	-0.30671
EXTL1	4	0.61629	0.69947	0.980024	1252	1	-0.090625
DPAGT1	4	0.61766	0.7002	0.980024	1253	1	-0.12533
GALNT11	4	0.61956	0.70114	0.980024	1254	1	-1.2098
UGT2A3	4	0.62092	0.70178	0.980024	1255	1	-0.49494
PLPPR2	4	0.62119	0.70189	0.980024	1256	1	-0.11186
GAA	4	0.62254	0.70267	0.980024	1257	1	-0.14893
NDST1	4	0.62415	0.70347	0.980024	1258	1	-0.003444

							5
ST3GAL4	4	0.62469	0.70378	0.980024	1259	1	-0.11968
LARGE1	4	0.62577	0.70428	0.980024	1260	1	0.039896
CYP11A1	4	0.6263	0.70449	0.980024	1261	1	-0.3856
DPM1	4	0.6279	0.70526	0.980024	1262	1	0.0094381
CYP4F12	4	0.6287	0.70569	0.980024	1263	1	0.059632
CHI3L2	4	0.62924	0.706	0.980024	1264	1	-0.023864
GNS	4	0.6303	0.70652	0.980024	1265	1	0.02059
GLB1L	4	0.63083	0.70677	0.980024	1266	1	-2.2986
CPS1	4	0.63189	0.70732	0.980024	1267	1	-0.051908
ST8SIA5	4	0.63295	0.70777	0.980024	1268	1	-0.45926
ADAL	4	0.63321	0.70788	0.980024	1269	1	-0.13887
POFUT2	4	0.63453	0.70849	0.980024	1270	1	0.041741
NDUFS1	4	0.63506	0.70874	0.980024	1271	1	0.13162
SULF1	4	0.6369	0.70962	0.980024	1272	1	-0.52528
PFKM	4	0.63794	0.71014	0.980024	1273	1	0.012091
THNSL2	4	0.63977	0.71099	0.980024	1274	1	-1.0815
UROD	4	0.64055	0.71144	0.980024	1275	1	-0.41117
PLPP7	4	0.64185	0.71201	0.980024	1276	1	-0.046552
CYP1A1	4	0.64211	0.71215	0.980024	1277	1	-0.24191
MGAT5B	4	0.64289	0.71264	0.980024	1278	1	-0.10742
DHODH	4	0.64315	0.7128	0.980024	1279	1	0.063521
UGT8	4	0.64366	0.71312	0.980024	1280	1	-0.19683
PNPLA8	4	0.64495	0.7139	0.980024	1281	1	-0.76262
DPYSL4	4	0.64855	0.71575	0.980024	1282	1	-0.59277
GADL1	4	0.64957	0.71622	0.980024	1283	1	-0.26996
GLT8D2	4	0.6511	0.71691	0.980024	1284	1	-0.11853
GALNT2	4	0.6516	0.71716	0.980024	1285	1	-0.16674
DSEL	3	0.65401	0.66834	0.980024	1286	1	0.13074
MTHFR	4	0.65515	0.71894	0.980024	1287	1	0.065746
NAT8L	4	0.6554	0.71902	0.980024	1288	1	-0.61086
IDO2	4	0.65691	0.71979	0.980024	1289	1	-0.34791
AGMAT	4	0.65716	0.71988	0.980024	1290	1	-0.65933
COX8A	4	0.65766	0.7202	0.980024	1291	1	-0.50704
CHST10	4	0.65791	0.7203	0.980024	1292	1	0.0047091
PDXK	4	0.65891	0.72082	0.980024	1293	1	0.13351
GYS1	4	0.66016	0.72142	0.980024	1294	1	-0.14096
AUH	4	0.66041	0.72151	0.980024	1295	1	-0.11779
CA7	4	0.66215	0.72246	0.980024	1296	1	-0.32863
LYZ	4	0.6624	0.72255	0.980024	1297	1	0.10949
DAGLA	4	0.66314	0.72288	0.980024	1298	1	-0.27205
GLB1L3	4	0.66339	0.72302	0.980024	1299	1	0.070913
GSTM5	4	0.66463	0.72365	0.980024	1300	1	-0.25526
GAL3ST4	4	0.66512	0.72391	0.980024	1301	1	-0.1322
CYB5B	4	0.66537	0.724	0.980024	1302	1	-0.090689
NT5C1A	4	0.6666	0.72469	0.980024	1303	1	-0.288

COQ8A	4	0.66881	0.72593	0.980024	1304	1	-0.081199
GNPTAB	4	0.66978	0.72636	0.980024	1305	1	-0.20165
CSAD	4	0.67051	0.72682	0.980024	1306	1	-0.58917
NUDT8	4	0.67391	0.72867	0.980024	1307	1	-0.17275
HADHB	4	0.67512	0.72931	0.980024	1308	1	-0.41031
HPRT1	4	0.6756	0.72963	0.980024	1309	1	-0.022304
DHRS7C	4	0.67584	0.72975	0.980024	1310	1	-0.047568
PDE5A	4	0.67656	0.73016	0.980024	1311	1	-0.099054
DPEP1	4	0.6768	0.73027	0.980024	1312	1	-0.19566
TBXAS1	4	0.67752	0.73057	0.980024	1313	1	-0.13517
ACY3	4	0.67848	0.73115	0.980024	1314	1	-0.17254
PFKFB4	4	0.68229	0.73321	0.980024	1315	1	-0.15264
AHCYL2	4	0.68277	0.73347	0.980024	1316	1	0.093445
HK3	4	0.68348	0.7339	0.980024	1317	1	0.064682
NUDT6	4	0.68418	0.73422	0.980024	1318	1	0.13378
SULT1A3	4	0.68654	0.73561	0.980024	1319	1	-0.8684
HSD17B2	4	0.68677	0.73577	0.980024	1320	1	-0.025831
B3GNT3	4	0.68724	0.736	0.980024	1321	1	-0.10532
GCNT1	4	0.68818	0.73658	0.980024	1322	1	-0.58245
ENTPD4	4	0.68958	0.73735	0.980024	1323	1	-0.17679
AASDHPPT	4	0.69004	0.73762	0.980024	1324	1	-0.21401
DDC	4	0.69051	0.73781	0.980024	1325	1	-0.25056
MAN1A1	4	0.69144	0.73835	0.980024	1326	1	-0.14864
EDEM2	4	0.6919	0.73865	0.980024	1327	1	-0.28752
ACOXL	4	0.6926	0.73906	0.980024	1328	1	-0.38458
ALG5	4	0.69329	0.73947	0.980024	1329	1	-0.47289
PI4KA	4	0.69582	0.74087	0.980024	1330	1	-0.51021
ACBD4	4	0.69651	0.74122	0.980024	1331	1	0.036013
AS3MT	4	0.69742	0.74174	0.980024	1332	1	-0.78013
DDHD2	4	0.69834	0.74233	0.980024	1333	1	-0.1401
HMOX1	4	0.69879	0.74259	0.980024	1334	1	-0.64645
PFKFB2	4	0.70038	0.74353	0.980024	1335	1	0.17341
ABHD8	4	0.70106	0.74384	0.980024	1336	1	-0.32276
SULF2	4	0.70129	0.74395	0.980024	1337	1	-0.0088318
RDH14	4	0.70152	0.74407	0.980024	1338	1	0.0087995
SMOX	4	0.70197	0.74435	0.980024	1339	1	-0.7952
MTAP	4	0.70467	0.74589	0.980024	1340	1	-0.57697
RDH16	4	0.70534	0.74621	0.980024	1341	1	-0.11168
NDUFA3	4	0.70669	0.74698	0.980024	1342	1	-0.12179
DHFR	4	0.70802	0.74772	0.980024	1343	1	-0.71804
ADCY1	4	0.70891	0.74836	0.980024	1344	1	-0.35746
NADSYN1	4	0.70913	0.74852	0.980024	1345	1	-0.13878
MANBA	4	0.70958	0.74873	0.980024	1346	1	-0.22753
NDUFV3	4	0.71024	0.74912	0.980024	1347	1	-0.077689
ETFB	4	0.71091	0.74957	0.980024	1348	1	0.07115

ADA2	4	0.71113	0.74965	0.980024	1349	1	-0.08543
HS3ST3A1	4	0.71135	0.74977	0.980024	1350	1	-0.066357
GLRX3	4	0.71223	0.75025	0.980024	1351	1	-0.089218
SUCLG1	4	0.71267	0.75046	0.980024	1352	1	-0.29814
GLDC	4	0.71289	0.75063	0.980024	1353	1	-0.51638
KYNU	4	0.71333	0.7509	0.980024	1354	1	-0.39199
UPRT	4	0.71377	0.75113	0.980024	1355	1	-0.30196
NAGK	4	0.7153	0.75213	0.980024	1356	1	-0.039555
MINPP1	4	0.71552	0.75225	0.980024	1357	1	-0.1839
INPP5B	4	0.71747	0.75336	0.980024	1358	1	-0.39312
ACE2	4	0.71769	0.75347	0.980024	1359	1	-1.1607
CTBS	4	0.71813	0.7538	0.980024	1360	1	-0.87044
DAD1	4	0.71942	0.75457	0.980024	1361	1	-0.8869
OGDHL	4	0.72115	0.75558	0.980024	1362	1	0.018463
PDE6C	4	0.72222	0.75625	0.980024	1363	1	-0.30597
SATL1	4	0.72265	0.7566	0.980024	1364	1	-0.091079
PDE3A	4	0.7235	0.75713	0.980024	1365	1	-0.44686
GLB1	4	0.72542	0.75823	0.980024	1366	1	-0.87913
AK2	4	0.72775	0.75962	0.980024	1367	1	0.010221
PIGC	4	0.72923	0.76046	0.980024	1368	1	-0.20069
PCYT2	4	0.72986	0.76086	0.980024	1369	1	-1.3763
SUOX	4	0.73028	0.76116	0.980024	1370	1	-0.18983
OAS1	4	0.7307	0.76137	0.980024	1371	1	-0.47718
ASNSD1	4	0.73216	0.76223	0.980024	1372	1	-0.0027627
PIGT	4	0.73278	0.76253	0.980024	1373	1	-0.71698
NDUFA1	4	0.73692	0.76522	0.980024	1374	1	-0.30321
GAPDHS	4	0.73775	0.76573	0.980024	1375	1	-0.30735
GYS2	4	0.73918	0.76655	0.980024	1376	1	-0.36492
GDPD3	4	0.74041	0.76725	0.980024	1377	1	-0.25018
PDE4A	4	0.74183	0.76813	0.980024	1378	1	-0.47329
NUDT3	4	0.74284	0.76878	0.980024	1379	1	-0.032608
ALG14	4	0.74304	0.7689	0.980024	1380	1	-0.38047
PKLR	4	0.74345	0.7692	0.980024	1381	1	-0.15332
INPP5K	4	0.74365	0.76928	0.980024	1382	1	-0.0034407
HMGCL	4	0.74385	0.76945	0.980024	1383	1	-0.37649
MMACHC	4	0.74405	0.76955	0.980024	1384	1	0.015773
CRLS1	4	0.74466	0.76994	0.980024	1385	1	-0.041005
FUT6	4	0.74586	0.77069	0.980024	1386	1	-0.090458
ACSL5	4	0.74646	0.77115	0.980024	1387	1	0.083622
PCCA	4	0.74686	0.77141	0.980024	1388	1	-0.83261
ACBD7	4	0.74806	0.77225	0.980024	1389	1	-0.10081
PIGX	4	0.74866	0.77261	0.980024	1390	1	-0.29449
TPH1	4	0.74945	0.77321	0.980024	1391	1	0.12821
NDST3	4	0.74965	0.77333	0.980024	1392	1	-0.22154

HADH	4	0.75025	0.77371	0.980024	1393	1	-1.0758
AGPAT1	4	0.75104	0.77432	0.980024	1394	1	-0.35102
BCKDHA	4	0.75123	0.77446	0.980024	1395	1	-0.18595
RDH11	4	0.75163	0.77469	0.980024	1396	1	-0.10875
HCCS	4	0.75261	0.77537	0.980024	1397	1	-0.55724
AOC2	4	0.75281	0.77548	0.980024	1398	1	0.079101
UGT1A6	4	0.7532	0.7757	0.980024	1399	1	-0.61763
ST8SIA6	4	0.75379	0.7761	0.980024	1400	1	-0.19085
SGSH	4	0.75594	0.77746	0.980024	1401	1	-0.27402
GAPDH	4	0.7573	0.77827	0.980024	1402	1	0.011237
SOD2	4	0.75942	0.77979	0.980024	1403	1	-0.35426
TPO	4	0.75961	0.77994	0.980024	1404	1	-0.54637
NT5DC2	4	0.7598	0.78009	0.980024	1405	1	0.062208
B4GALT3	4	0.76019	0.78041	0.980024	1406	1	-0.3648
GCNT2	4	0.76057	0.78071	0.980024	1407	1	-0.11368
NOX3	4	0.76076	0.78087	0.980024	1408	1	-0.093139
GCNT3	4	0.76134	0.78129	0.980024	1409	1	-0.70026
NQO2	4	0.76229	0.78199	0.980024	1410	1	-0.24166
MVD	4	0.76305	0.78251	0.980024	1411	1	0.12671
MBOAT1	4	0.76382	0.78301	0.980024	1412	1	-0.15121
HMGCS2	4	0.76438	0.78335	0.980024	1413	1	-0.012728
PLA1A	4	0.76608	0.78455	0.980024	1414	1	-0.17251
PLD5	4	0.76646	0.78477	0.980024	1415	1	-0.79387
ECI1	4	0.76983	0.78718	0.980024	1416	1	-0.081382
CYP4F11	4	0.7702	0.78735	0.980024	1417	1	-0.23601
PTGR2	4	0.77076	0.78769	0.980024	1418	1	-0.98005
ST6GALNAC5	4	0.77095	0.78783	0.980024	1419	1	-0.45483
ST6GALNAC4	4	0.77113	0.78794	0.980024	1420	1	-0.054977
BCO1	4	0.77169	0.78834	0.980024	1421	1	-0.15958
GPD1L	4	0.77243	0.78894	0.980024	1422	1	-1.5302
KYAT1	4	0.77335	0.7896	0.980024	1423	1	-0.30619
DPYS	4	0.77353	0.78974	0.980024	1424	1	-0.091813
GSTM4	4	0.77536	0.79094	0.980024	1425	1	-0.65868
FMO3	4	0.77701	0.79208	0.980024	1426	1	-0.63803
AMD1	4	0.77737	0.79233	0.980024	1427	1	-0.11035
MCAT	4	0.77773	0.7926	0.980024	1428	1	0.055042
ACBD5	4	0.77791	0.7927	0.980024	1429	1	-0.20188
OGDH	4	0.77809	0.79284	0.980024	1430	1	-0.044262
RDH12	4	0.77828	0.79296	0.980024	1431	1	-0.66586
NAGLU	4	0.77846	0.79305	0.980024	1432	1	0.0036511
CHSY3	4	0.77972	0.79409	0.980024	1433	1	-0.012006
DHCR7	4	0.78116	0.79498	0.980024	1434	1	0.0086375
ALDOA	4	0.78187	0.79554	0.980024	1435	1	-0.42418
DCTD	4	0.78205	0.79568	0.980024	1436	1	-0.32335
SOAT2	4	0.78223	0.7958	0.980024	1437	1	-0.22622
PIK3CD	4	0.78259	0.79608	0.980024	1438	1	-0.031821

HAO1	4	0.78312	0.79643	0.980024	1439	1	-0.66959
ADHFE1	4	0.78383	0.79695	0.980024	1440	1	0.11284
PLA2G7	3	0.78468	0.78497	0.980024	1441	0	-0.40372
GFOD1	4	0.78472	0.79757	0.980024	1442	1	-0.40328
MGAM	4	0.78525	0.79796	0.980024	1443	1	-0.24189
PDE1B	4	0.78543	0.79806	0.980024	1444	1	0.13578
NANS	4	0.78613	0.79852	0.980024	1445	1	-0.80292
DPYD	4	0.78754	0.79945	0.980024	1446	1	-0.09257
RDH8	4	0.78806	0.79988	0.980024	1447	1	-0.64536
ACOT12	4	0.78841	0.80011	0.980024	1448	1	-0.23023
HK2	4	0.78998	0.80137	0.980024	1449	1	-0.28482
GLRX2	4	0.79016	0.80148	0.980024	1450	1	-0.36466
SULT6B1	4	0.79102	0.80223	0.980024	1451	1	-0.48356
MSRB3	4	0.7912	0.80238	0.980024	1452	1	-0.28577
ALG11	4	0.79172	0.80269	0.980024	1453	1	-0.25016
CTH	4	0.79241	0.80326	0.980038	1454	1	-0.73949
SULT1C2	4	0.79412	0.80453	0.980916	1455	1	-0.29051
MAN2C1	4	0.79617	0.80612	0.981177	1456	1	-0.12387
ENOSF1	4	0.79685	0.80665	0.981177	1457	1	-0.50188
POFUT1	4	0.79753	0.8072	0.981177	1458	1	0.0076481
CMBL	4	0.7977	0.80731	0.981177	1459	1	-0.44452
GALNT5	4	0.79837	0.80782	0.981177	1460	1	-0.49146
HS3ST5	4	0.79871	0.80806	0.981177	1461	1	-0.079027
CA5A	4	0.79972	0.80874	0.981326	1462	1	0.059103
GART	4	0.80089	0.80954	0.981629	1463	1	-0.050632
GLCE	4	0.80272	0.81091	0.982052	1464	1	-0.7579
ALG12	4	0.80288	0.81101	0.982052	1465	1	0.057888
SUCLG2	4	0.80355	0.81155	0.982052	1466	1	-0.21108
PIP5K1C	4	0.80716	0.81475	0.984882	1467	1	-0.25407
NT5E	4	0.80748	0.815	0.984882	1468	1	-0.35795
SULT1A2	4	0.80846	0.81585	0.985236	1469	1	-0.27352
G6PD	4	0.81024	0.81735	0.985983	1470	1	-0.19426
UPB1	4	0.81056	0.81758	0.985983	1471	1	-0.44476
CEL	4	0.812	0.81867	0.986559	1472	1	-0.23312
GUK1	4	0.8128	0.81934	0.986559	1473	1	-0.57732
SULT1C4	4	0.81391	0.8203	0.986559	1474	1	-0.07956
PLPP1	4	0.81486	0.821	0.986559	1475	1	-0.55556
ME1	4	0.81565	0.82157	0.986559	1476	1	-0.24486
ENPP3	4	0.81581	0.82171	0.986559	1477	1	-0.19684
MOCS1	4	0.81613	0.82195	0.986559	1478	1	-0.11648
ADH4	4	0.81754	0.82311	0.98698	1479	1	-0.69541
PIGQ	4	0.81925	0.82438	0.98698	1480	1	0.015919
NT5DC3	4	0.82018	0.82507	0.98698	1481	1	-0.52726
ECI2	4	0.82034	0.82525	0.98698	1482	1	-0.46131
PDXP	4	0.82049	0.8254	0.98698	1483	1	-0.27226
VKORC1	4	0.8208	0.82564	0.98698	1484	1	0.032077

AADAC	4	0.82341	0.82775	0.987857	1485	1	-0.39382
PGK1	4	0.82356	0.82786	0.987857	1486	1	0.029417
THNSL1	4	0.82569	0.82949	0.987857	1487	1	-0.51532
PGAM5	4	0.82644	0.83021	0.987857	1488	1	-0.29642
LIPN	4	0.82659	0.83032	0.987857	1489	1	-0.081784
CNDP2	4	0.82674	0.83044	0.987857	1490	1	-0.41161
EPX	4	0.82719	0.83077	0.987857	1491	1	-0.16433
NAT8	4	0.82824	0.83169	0.987857	1492	1	0.041135
CAD	4	0.82854	0.8319	0.987857	1493	1	-0.39969
CYP2A7	4	0.82914	0.83237	0.987857	1494	1	-0.038427
NNMT	4	0.82929	0.8325	0.987857	1495	1	-0.071042
CYP4B1	4	0.83092	0.83397	0.988944	1496	1	-0.15217
NUDT7	4	0.8321	0.835	0.989309	1497	1	-0.97948
MGAT4B	4	0.83254	0.83539	0.989309	1498	1	-0.21622
GOT1	4	0.83414	0.83677	0.990281	1499	1	-0.014617
LIPI	4	0.83545	0.83783	0.990875	1500	1	-0.32262
PLD3	4	0.83689	0.83899	0.99158	1501	1	-0.13817
GUCY1A2	4	0.83775	0.83985	0.991941	1502	1	-0.42096
B3GALT5	4	0.83975	0.84175	0.992536	1503	1	-0.38012
ARSB	4	0.83989	0.84192	0.992536	1504	1	-0.22577
EHHADH	4	0.84003	0.84203	0.992536	1505	1	-0.11649
CSGALNACT2	4	0.84159	0.84346	0.99334	1506	1	-0.40589
B4GALNT2	4	0.84243	0.84419	0.99334	1507	1	-0.087387
DGAT2L6	4	0.84424	0.84593	0.99334	1508	1	-0.1475
PLD4	4	0.84507	0.84673	0.99334	1509	1	-0.072287
HMBS	4	0.84521	0.84684	0.99334	1510	1	-0.20486
BPNT1	4	0.84535	0.84693	0.99334	1511	1	-0.18398
LYPLAL1	4	0.84576	0.84728	0.99334	1512	1	-0.54972
EPHX2	4	0.84618	0.84763	0.99334	1513	1	-0.63187
CYBB	4	0.84741	0.84888	0.99334	1514	1	-0.58038
ACER2	4	0.84782	0.84924	0.99334	1515	1	-0.038006
NUDT15	4	0.84891	0.85024	0.99334	1516	1	0.081299
PLCD1	4	0.85026	0.85147	0.99334	1517	1	0.0030873
MAN2A2	4	0.85147	0.85248	0.99334	1518	1	0.12114
ST6GAL1	4	0.85174	0.85278	0.99334	1519	1	-0.5973
LPCAT4	4	0.85201	0.85305	0.99334	1520	1	-0.12857
XDH	4	0.85268	0.85369	0.99334	1521	1	-0.71166
CA14	4	0.85308	0.85406	0.99334	1522	1	-0.40189
TPH2	4	0.85374	0.8547	0.99334	1523	1	-0.089764
NT5C1B	4	0.85427	0.85519	0.99334	1524	1	-0.67897
PGAM4	4	0.85466	0.8556	0.99334	1525	1	-0.46396
PRDX1	4	0.85506	0.85606	0.99334	1526	1	-0.75635
NUDT18	4	0.85519	0.85618	0.99334	1527	1	-0.38046
PGM3	4	0.85676	0.85773	0.99334	1528	1	-0.34766
HIBADH	4	0.85754	0.85855	0.99334	1529	1	-0.36902
PLA2G4E	4	0.85793	0.85897	0.99334	1530	1	0.11737

MBOAT2	4	0.85845	0.85941	0.99334	1531	1	-0.1626
UQCRB	4	0.85871	0.85963	0.99334	1532	1	0.063826
SHMT1	4	0.86	0.86086	0.99334	1533	1	-0.28923
GBGT1	4	0.86064	0.86139	0.99334	1534	0	-0.34986
FIG4	4	0.86102	0.86183	0.99334	1535	0	-0.74746
CH25H	4	0.86229	0.86312	0.99334	1536	0	-0.22439
PGAP1	4	0.8628	0.86362	0.99334	1537	0	-0.188
AK1	4	0.86292	0.86378	0.99334	1538	0	0.13742
UROS	4	0.86317	0.86402	0.99334	1539	0	-0.79035
DHRS4L1	4	0.86355	0.86441	0.99334	1540	0	-0.14743
COX19	4	0.86393	0.86481	0.99334	1541	0	-0.50759
AMPD2	4	0.86431	0.86522	0.99334	1542	0	-0.12751
DAGLB	4	0.86443	0.86533	0.99334	1543	0	-0.1055
DIO2	4	0.86481	0.86565	0.99334	1544	0	-0.36943
MMADHC	4	0.86493	0.86578	0.99334	1545	0	-0.4012
MTHFD2L	4	0.86741	0.86805	0.99334	1546	0	-0.55351
FAHD2B	4	0.86753	0.86814	0.99334	1547	0	-0.52765
SULT1C3	4	0.86839	0.86887	0.99334	1548	0	-0.5823
ST6GAL2	4	0.86864	0.86912	0.99334	1549	0	0.062791
PLA2G6	4	0.869	0.86944	0.99334	1550	0	-0.19098
TPMT	4	0.86925	0.86967	0.99334	1551	0	-0.19453
PYCR1	4	0.86937	0.86976	0.99334	1552	0	-0.226
PPCS	4	0.86985	0.87026	0.99334	1553	0	-0.51723
NMNAT2	4	0.87034	0.87074	0.99334	1554	0	-0.36077
A1CF	4	0.87143	0.87194	0.99334	1555	0	-0.22665
AASS	4	0.87179	0.87229	0.99334	1556	0	-0.59247
ASAH2	4	0.87191	0.87241	0.99334	1557	0	-0.0031929
DGAT1	4	0.87215	0.87269	0.99334	1558	0	-0.42867
NAALAD2	4	0.87274	0.87334	0.99334	1559	0	-0.34167
UST	4	0.8731	0.87375	0.99334	1560	0	-0.42509
D2HGDH	4	0.875	0.87571	0.99334	1561	0	-0.44548
B3GALNT1	4	0.87535	0.87606	0.99334	1562	0	-0.074366
PNPLA5	4	0.87558	0.87627	0.99334	1563	0	-0.62378
HSD11B2	4	0.87594	0.87671	0.99334	1564	0	-0.47165
GPHN	4	0.87617	0.87693	0.99334	1565	0	-0.0038371
BBOX1	4	0.8771	0.87782	0.99334	1566	0	-0.1937
FMO5	4	0.87791	0.87858	0.99334	1567	0	-0.48851
B4GALT6	4	0.87826	0.8789	0.99334	1568	0	-0.31278
NAALADL2	4	0.8793	0.87978	0.99334	1569	0	0.1211
ALG2	4	0.87953	0.88001	0.99334	1570	0	-0.85322
ACSF3	4	0.87998	0.88044	0.99334	1571	0	-0.36878
AK3	4	0.88044	0.88083	0.99334	1572	0	-0.90781
PDE1A	4	0.88214	0.8825	0.99334	1573	0	-0.67258
TSTA3	4	0.88259	0.88293	0.99334	1574	0	0.05016

SPTLC2	4	0.88304	0.88343	0.99334	1575	0	-0.31484
HSDL1	4	0.8836	0.88393	0.99334	1576	0	-0.20307
SPHK2	4	0.88382	0.88409	0.99334	1577	0	-0.42166
MICAL2	4	0.88438	0.88454	0.99334	1578	0	-0.2585
LDHA	4	0.8846	0.88473	0.99334	1579	0	-0.15257
CHST12	4	0.88504	0.8852	0.99334	1580	0	0.024491
NOS1	4	0.88614	0.88632	0.99334	1581	0	-0.26949
GALNT9	4	0.88702	0.88716	0.99334	1582	0	0.046607
AGPAT3	4	0.88746	0.88763	0.99334	1583	0	-0.20232
MECR	4	0.88789	0.88806	0.99334	1584	0	0.02496
ACOX2	4	0.88843	0.88856	0.99334	1585	0	-0.32645
DHRS11	4	0.88865	0.88874	0.99334	1586	0	0.0054794
HACL1	4	0.88951	0.88962	0.99334	1587	0	-0.039922
TPI1	4	0.89015	0.89033	0.99334	1588	0	-0.72028
SGMS2	4	0.89069	0.89082	0.99334	1589	0	-0.007493
FUT4	4	0.89111	0.89128	0.99334	1590	0	-0.32565
TDO2	4	0.89122	0.89139	0.99334	1591	0	-0.43494
CYB5A	4	0.89132	0.89148	0.99334	1592	0	-0.61131
PIGS	4	0.89185	0.89199	0.99334	1593	0	0.081926
SEPHS1	4	0.89291	0.89305	0.993897	1594	0	-0.36414
CDADC1	4	0.8952	0.89517	0.994989	1595	0	-4.6123
DCT	4	0.89673	0.89663	0.994989	1596	0	-0.62175
ALDH1A3	4	0.89694	0.89685	0.994989	1597	0	-0.10351
CYP26A1	4	0.89956	0.89959	0.994989	1598	0	-0.097833
LYPLA1	4	0.89986	0.8999	0.994989	1599	0	-0.097109
IDO1	4	0.90046	0.90043	0.994989	1600	0	-0.4574
AMY1A	4	0.90174	0.90164	0.994989	1601	0	-0.45607
NOS2	4	0.90184	0.90174	0.994989	1602	0	-0.57142
GBA2	4	0.90292	0.90273	0.994989	1603	0	-0.14216
CHPT1	4	0.90302	0.90286	0.994989	1604	0	-0.80883
ITPKC	4	0.9036	0.90341	0.994989	1605	0	-0.20905
CHDH	4	0.9037	0.90349	0.994989	1606	0	-0.19742
HIBCH	4	0.90399	0.90376	0.994989	1607	0	-0.23312
PNLIP	4	0.90466	0.90446	0.994989	1608	0	-0.43807
UGT1A3	4	0.90504	0.90482	0.994989	1609	0	-0.09864
PDHA2	4	0.90524	0.90501	0.994989	1610	0	-0.35171
ST6GALNAC3	4	0.90676	0.90633	0.994989	1611	0	-1.0133
CERS5	4	0.90732	0.90705	0.994989	1612	0	-0.54379
GPAM	4	0.90789	0.90771	0.994989	1613	0	-0.1347
PNLIPRP2	4	0.90863	0.90847	0.994989	1614	0	-0.44624
PLPP5	4	0.90956	0.90938	0.994989	1615	0	-0.60086
CAT	4	0.90984	0.90966	0.994989	1616	0	-0.60717
UGT2B11	4	0.91021	0.91009	0.994989	1617	0	-0.2203
HSD11B1	4	0.91039	0.91029	0.994989	1618	0	-0.6169
HYAL4	4	0.91158	0.9115	0.994989	1619	0	-0.20299
CBS	4	0.91185	0.91176	0.994989	1620	0	-0.11539

GMPPB	4	0.91239	0.91232	0.994989	1621	0	-0.92926
CERK	4	0.91257	0.91251	0.994989	1622	0	-0.51309
GPX6	4	0.91329	0.91328	0.994989	1623	0	-0.46585
ACOX1	4	0.91338	0.91336	0.994989	1624	0	0.010123
SDHD	4	0.91374	0.91366	0.994989	1625	0	-0.34276
ACOT1	4	0.91418	0.91403	0.994989	1626	0	-1.1312
ST3GAL5	4	0.91471	0.91459	0.994989	1627	0	-0.39321
PRDX5	4	0.91489	0.91478	0.994989	1628	0	-0.50561
NUDT21	4	0.91498	0.91486	0.994989	1629	0	-0.9534
DEGS2	4	0.91577	0.91563	0.994989	1630	0	-0.47511
LCTL	4	0.91594	0.91576	0.994989	1631	0	-0.003545<br>3
PAICS	4	0.91603	0.91582	0.994989	1632	0	-0.13352
GALNS	4	0.91612	0.91591	0.994989	1633	0	-0.13937
CYP4F8	4	0.91708	0.91693	0.99505	1634	0	-0.16087
PIP4K2A	4	0.91725	0.91708	0.99505	1635	0	-0.57901
MVK	4	0.91794	0.91779	0.995068	1636	0	-0.10641
GSTA5	4	0.91837	0.91822	0.995068	1637	0	-0.22508
ACY1	4	0.91913	0.91893	0.995228	1638	0	-0.48516
LIPH	4	0.92174	0.92154	0.99576	1639	0	-0.63871
TYMS	4	0.92215	0.92196	0.99576	1640	0	-0.24875
CYP3A43	4	0.92248	0.92228	0.99576	1641	0	-0.51484
B4GALT4	4	0.92264	0.9225	0.99576	1642	0	-0.28009
ALOX12B	4	0.92379	0.92353	0.99576	1643	0	-0.50531
B3GALT2	4	0.92395	0.92378	0.99576	1644	0	-0.38836
ELOVL2	4	0.92468	0.92456	0.99576	1645	0	-0.78704
CYP2U1	4	0.92675	0.92659	0.99576	1646	0	-0.4526
EXTL3	4	0.92706	0.92687	0.99576	1647	0	-0.019772
THEM5	4	0.92722	0.92703	0.99576	1648	0	-0.91332
GGPS1	4	0.92785	0.92765	0.99576	1649	0	-0.47161
PRDX6	4	0.92816	0.92805	0.99576	1650	0	-0.097772
AMDHD1	4	0.92893	0.92878	0.99576	1651	0	-0.50052
GUCY2F	4	0.92978	0.92958	0.99576	1652	0	-0.59714
GSR	4	0.93174	0.93149	0.99576	1653	0	-0.39947
AKR1C3	4	0.93264	0.93245	0.99576	1654	0	-0.16671
MAOB	4	0.93323	0.93299	0.99576	1655	0	-0.13021
LPGAT1	4	0.93338	0.9331	0.99576	1656	0	-0.3233
ACAD10	4	0.93345	0.93319	0.99576	1657	0	-0.24578
HSD17B6	4	0.93411	0.93387	0.99576	1658	0	-0.25798
FAAH2	4	0.9344	0.93422	0.99576	1659	0	-0.62803
IDH3G	4	0.93447	0.93429	0.99576	1660	0	-0.7354
DCK	4	0.93469	0.93454	0.99576	1661	0	-0.47215
NUDT5	4	0.93483	0.93467	0.99576	1662	0	-0.51388
SPTLC3	4	0.9349	0.93471	0.99576	1663	0	-0.30895
GALNT16	4	0.93584	0.93557	0.99576	1664	0	-0.42233
ENOX2	4	0.93683	0.93637	0.99576	1665	0	-0.12308

AGPAT4	4	0.93732	0.93674	0.99576	1666	0	-0.44845
PPA2	4	0.93739	0.9368	0.99576	1667	0	-1.0769
HYAL3	4	0.93816	0.93762	0.99576	1668	0	-0.41444
GALNT18	4	0.9392	0.93859	0.99576	1669	0	-0.27403
PDSS1	4	0.93975	0.93914	0.99576	1670	0	-0.33855
DHRS12	4	0.94083	0.94024	0.99576	1671	0	-0.80571
GLB1L2	4	0.9419	0.94133	0.99576	1672	0	-0.91391
COX18	4	0.94203	0.94141	0.99576	1673	0	-0.2986
IYD	4	0.94295	0.94237	0.99576	1674	0	-0.25308
MAN2B1	4	0.94463	0.94413	0.99576	1675	0	-0.39535
NEU4	4	0.94469	0.94418	0.99576	1676	0	-0.36072
NDUFA12	4	0.9452	0.94479	0.99576	1677	0	-0.20837
DUOX2	4	0.94558	0.9452	0.99576	1678	0	-0.27464
IDUA	4	0.94577	0.94534	0.99576	1679	0	-0.35013
INPP5J	4	0.94615	0.94575	0.99576	1680	0	-0.71122
ACAA1	4	0.94659	0.94616	0.99576	1681	0	-0.64192
ELOVL4	4	0.94696	0.94659	0.99576	1682	0	-0.45115
PPA1	4	0.94727	0.94693	0.99576	1683	0	-0.34365
NPR2	4	0.94758	0.94726	0.99576	1684	0	-0.25972
GBE1	4	0.94806	0.94784	0.99576	1685	0	-0.22049
ST3GAL3	4	0.94813	0.9479	0.99576	1686	0	-0.55848
BCO2	4	0.95005	0.94993	0.99576	1687	0	-1.123
SULT2B1	4	0.95034	0.95024	0.99576	1688	0	-0.22297
CYP3A4	4	0.95075	0.95059	0.99576	1689	0	-0.16725
CHST14	4	0.95093	0.95077	0.99576	1690	0	-0.7033
ARSK	4	0.9511	0.95095	0.99576	1691	0	-0.20928
NUDT13	4	0.95116	0.95101	0.99576	1692	0	-0.89304
CHST1	4	0.95163	0.95151	0.99576	1693	0	-0.27681
PTEN	4	0.95191	0.95182	0.99576	1694	0	-0.86862
BCAT1	4	0.95203	0.95194	0.99576	1695	0	-0.69315
MTHFD1	4	0.95209	0.95199	0.99576	1696	0	-0.32763
DECR1	4	0.95283	0.95285	0.99576	1697	0	-0.17758
CYP4A22	4	0.95339	0.95341	0.99576	1698	0	-0.66474
PRPS2	4	0.95367	0.95366	0.99576	1699	0	-0.60368
ITPKB	4	0.95571	0.95576	0.996044	1700	0	-0.35307
CYB561	4	0.95587	0.95589	0.996044	1701	0	-0.28107
LNPEP	4	0.95592	0.95597	0.996044	1702	0	-0.34331
GPX7	4	0.95614	0.95618	0.996044	1703	0	-0.3052
ELOVL5	4	0.95683	0.95685	0.996162	1704	0	-0.62393
ENPP6	4	0.95757	0.95766	0.99623	1705	0	-0.28954
PHOSPHO2	4	0.9582	0.95821	0.99623	1706	0	-0.26345
TAT	4	0.95932	0.95928	0.99623	1707	0	-0.40048
ST8SIA4	4	0.96083	0.9609	0.99623	1708	0	-0.5472
NDOR1	4	0.96097	0.96102	0.99623	1709	0	-0.2003
HPSE	4	0.96166	0.96165	0.99623	1710	0	-4.2176
NT5DC1	4	0.96195	0.96188	0.99623	1711	0	-0.18382

CYB5R1	4	0.962	0.96193	0.99623	1712	0	-0.39841
ACAT1	4	0.96333	0.96331	0.99623	1713	0	-0.52897
BTD	4	0.96384	0.96384	0.99623	1714	0	-0.3911
GLYCTK	4	0.96389	0.96386	0.99623	1715	0	-0.62403
UCK2	4	0.96435	0.9643	0.99623	1716	0	-0.15165
HSD17B3	4	0.96463	0.96457	0.99623	1717	0	-0.59018
GSTM3	4	0.96526	0.96515	0.99623	1718	0	-0.5496
FAR2	4	0.96544	0.96534	0.99623	1719	0	-0.35154
GBA	4	0.96716	0.96713	0.996733	1720	0	-0.74978
ACBD6	4	0.96835	0.96836	0.996733	1721	0	-0.59557
TREH	4	0.96885	0.96885	0.996733	1722	0	-0.22691
NDUFAF2	4	0.96894	0.96893	0.996733	1723	0	-0.41642
SQLE	4	0.97012	0.97003	0.996733	1724	0	-0.80411
IDS	4	0.97088	0.97076	0.996733	1725	0	-0.46068
CDS2	4	0.97185	0.97175	0.996733	1726	0	-0.69389
CYP11B2	4	0.97216	0.97202	0.996733	1727	0	-0.24507
ALG10	4	0.97269	0.97262	0.996733	1728	0	-0.76946
UAP1	4	0.97284	0.97279	0.996733	1729	0	-0.41402
TALDO1	4	0.97303	0.97292	0.996733	1730	0	-1.0679
SGPL1	4	0.97495	0.97483	0.996733	1731	0	-1.0891
HEXDC	4	0.97509	0.97498	0.996733	1732	0	-0.24494
ALDH1A1	4	0.97643	0.97631	0.996733	1733	0	-1.3319
PFKFB3	4	0.97667	0.97658	0.996733	1734	0	-0.22228
ACOT7	4	0.9773	0.97717	0.996733	1735	0	-0.7709
DGKK	4	0.9774	0.97731	0.996733	1736	0	-0.68206
PEMT	4	0.97746	0.97737	0.996733	1737	0	-0.34117
SI	4	0.97753	0.97741	0.996733	1738	0	-0.79678
COX5A	4	0.97769	0.97757	0.996733	1739	0	-0.20816
B4GALNT4	4	0.9783	0.97819	0.996733	1740	0	-0.22666
ECHDC1	4	0.97914	0.9791	0.996733	1741	0	-0.43361
ALDOC	4	0.97938	0.97936	0.996733	1742	0	-0.41587
CRAT	4	0.97981	0.97977	0.996733	1743	0	-0.59554
IP6K3	4	0.97988	0.97988	0.996733	1744	0	-0.42498
GLTP	4	0.98112	0.98111	0.997416	1745	0	-0.54525
ACSS1	4	0.98267	0.98256	0.997844	1746	0	-0.26887
CA5B	4	0.98278	0.98266	0.997844	1747	0	-0.79246
AGPS	4	0.98333	0.98322	0.997845	1748	0	-0.78384
NT5C3A	4	0.98419	0.98427	0.998339	1749	0	-0.55966
LIPA	4	0.98587	0.98595	0.998687	1750	0	-0.91674
GGTLC2	4	0.98597	0.98602	0.998687	1751	0	-0.47637
B4GALNT3	4	0.98633	0.98638	0.998687	1752	0	-0.37574
ALAS2	4	0.98675	0.98686	0.998687	1753	0	-0.76933
PDE1C	4	0.98809	0.98817	0.999433	1754	0	-0.95348
AKR1B1	4	0.98867	0.98874	0.999444	1755	0	-0.67812
NANP	4	0.99112	0.99111	0.999712	1756	0	-0.59953
ARG2	4	0.99135	0.99132	0.999712	1757	0	-0.51046

ADI1	4	0.99151	0.99143	0.999712	1758	0	-0.95922
PLA2G5	4	0.99226	0.99223	0.999712	1759	0	-0.48069
CYP4F2	4	0.99261	0.99264	0.999712	1760	0	-0.93673
FOXRED2	4	0.99279	0.99279	0.999712	1761	0	-0.66324
PLD1	4	0.99352	0.99345	0.999712	1762	0	-1.0542
AKR7A3	4	0.99529	0.99529	0.999712	1763	0	-1.0133
PKM	4	0.99584	0.99586	0.999712	1764	0	-1.043
LYZL1	4	0.99653	0.99657	0.999712	1765	0	-0.58902
ENPP2	4	0.997	0.9971	0.999712	1766	0	-1.2182
NQO1	4	0.99724	0.99732	0.999712	1767	0	-0.57283
CERS3	4	0.99775	0.99779	0.999712	1768	0	-1.919
ALG10B	4	0.99779	0.99784	0.999712	1769	0	-1.761
LBR	4	0.99793	0.99798	0.999712	1770	0	-0.64364
AZIN2	4	0.99811	0.99816	0.999712	1771	0	-0.97077
PDE4C	4	0.99857	0.99859	0.999712	1772	0	-0.75565
NPL	4	0.9997	0.99967	0.999997	1773	0	-1.2949
SUCLA2	4	0.99999	1	0.999997	1774	0	-2.7666
