# Supplementary material for: The AHCY–adenosine complex rewires mRNA methylation to enhance fatty acid biosynthesis and tumorigenesis
Source: Cell Res. 2026 Jan 19;36(2):152–72. doi: 10.1038/s41422-025-01213-5 (PMC12848013; doi:10.1038/s41422-025-01213-5)
Supplement: Supplementary file 12 — Supplementary information, Table S2 [file 41422_2025_1213_MOESM12_ESM.pdf]

Table S2. Ranked list of genes identified in the positive screen performed in HEK293T cells expressing the SSm6A reporter using the metabolic enzyme-related gene gRNA library.

Item	Description
**ID**	Gene or miRNA IDs.
**Num**	The number of targeting sgRNAs for this gene in the library.
**Pos|score**	The RRA lo value of this gene in positive selection.
**Pos|p-value**	The raw p-value of this gene for permutation test in positive selection.
**Pos|fdr**	The false discovery rate of this gene in positive selection.
**Pos|rank**	The ranking of this gene in positive selection.
**Pos|goodsgrna**	The number of "good" sgRNAs (sgRNAs whose ranking is below the selected alpha cutoff) in positive selection.
**Pos|lfc**	The log fold change of this gene in positive selection.

ID	Num	Pos|score	Pos|p-value	Pos|fdr	Pos|rank	Pos|goodsgrna	Pos|lfc
ACSS1	4	8.94E-05	0.0003265	0.579208	1	4	0.80834
CDA	4	0.00028	0.0010018	0.766364	2	1	-0.12493
ASNSD1	4	0.00038475	0.0014092	0.766364	3	4	0.92755
MCEE	4	0.0004985	0.0018278	0.766364	4	2	0.39264
AMY2B	4	0.00064382	0.0024306	0.766364	5	3	1.3488
CERS2	4	0.0008074	0.003078	0.766364	6	3	0.90614
DSEL	4	0.00083984	0.003212	0.766364	7	2	0.072688
DCXR	4	0.00096106	0.0035915	0.766364	8	4	0.70046
PIK3CB	4	0.0012836	0.0048026	0.766364	9	4	1.2113
ASAH2B	4	0.0013994	0.0052435	0.766364	10	3	1.215
COX6B1	4	0.0014068	0.0052547	0.766364	11	3	0.90692
COX7A1	4	0.0018196	0.0066555	0.766364	12	3	0.74761
PIK3C2G	4	0.0019588	0.0070853	0.766364	13	2	0.41111
CA8	4	0.001964	0.0071076	0.766364	14	4	0.62098
CP	4	0.0021844	0.0078778	0.766364	15	3	1.0266
ALDH3A1	4	0.0022616	0.0081569	0.766364	16	3	0.64794
CYP2C9	4	0.0025179	0.0089717	0.766364	17	2	0.47426
B3GNT2	4	0.002778	0.0098256	0.766364	18	3	0.89667
LIPT1	4	0.0028041	0.0098926	0.766364	19	4	1.0428
GK2	4	0.0029903	0.010523	0.766364	20	3	1.1512
CYP2D6	4	0.0030767	0.010841	0.766364	21	3	0.8974
GGT5	4	0.0031307	0.011026	0.766364	22	3	0.98836
DECR1	4	0.0031979	0.011193	0.766364	23	2	0.61078
LTA4H	4	0.0033244	0.01164	0.766364	24	3	0.84826
COLGALT2	4	0.0033955	0.011863	0.766364	25	3	0.88635
PDXK	4	0.0036355	0.012778	0.766364	26	2	0.25355
OXCT2	4	0.0037449	0.013102	0.766364	27	2	0.61288
SDR9C7	4	0.0038734	0.013442	0.766364	28	3	1.0938
PLPP7	4	0.003992	0.013766	0.766364	29	2	0.709
GYS2	4	0.0041185	0.014246	0.766364	30	3	0.92825
FMO5	4	0.004161	0.014363	0.766364	31	2	0.65954

GK5	4	0.0041939	0.014464	0.766364	32	1	-0.076485
HGSNAT	4	0.0042964	0.014754	0.766364	33	4	0.62088
LRAT	4	0.004353	0.014921	0.766364	34	4	0.69461
EBPL	4	0.0045362	0.015485	0.766364	35	4	0.59068
PDE3B	4	0.0045535	0.015552	0.766364	36	3	0.79882
ALDH16A1	4	0.0047521	0.016171	0.775355	37	2	-0.01722
ELOVL6	4	0.0050563	0.017288	0.803884	38	2	0.64403
GSR	4	0.0051635	0.017673	0.803884	39	4	0.78091
AHCYL2	4	0.00531	0.018147	0.804827	40	3	0.6928
NAGK	4	0.0055858	0.019079	0.825525	41	4	0.39252
INPP4A	4	0.0058677	0.0199	0.839627	42	3	0.76042
PLCL2	4	0.0059839	0.020352	0.839627	43	2	0.59249
CA10	4	0.0061557	0.020915	0.843272	44	4	0.77995
NSDHL	4	0.0064252	0.021758	0.857756	45	2	0.31552
UGT2B10	4	0.0068227	0.02302	0.887753	46	2	0.93647
PFAS	4	0.0074392	0.024923	0.916852	47	4	0.70249
**AHCY**	**4**	**0.007486**	**0.025157**	**0.916852**	**48**	**3**	**0.63343**
CYC1	4	0.0075395	0.025325	0.916852	49	1	-0.35085
CDO1	4	0.0080963	0.027127	0.952339	50	2	0.2162
NDUFB9	4	0.0081784	0.027378	0.952339	51	3	0.93599
ACER2	4	0.0085913	0.028779	0.956134	52	3	0.86995
ST3GAL4	4	0.0086528	0.029003	0.956134	53	2	0.11917
GLRX3	4	0.0087167	0.029203	0.956134	54	2	0.35947
PLCXD1	4	0.0092934	0.030945	0.956134	55	4	0.62555
B4GALNT3	4	0.0093775	0.031218	0.956134	56	4	0.62834
GCAT	4	0.0094216	0.031363	0.956134	57	3	0.77875
POR	4	0.009685	0.032134	0.956134	58	4	0.4374
NDUFB8	4	0.009906	0.032809	0.956134	59	2	0.65118
GALNT13	4	0.010035	0.033222	0.956134	60	2	0.59973
ACADSB	4	0.010321	0.034165	0.956134	61	2	0.24954
HSDL1	4	0.010362	0.034299	0.956134	62	2	0.15889
PLA2G15	4	0.010505	0.034701	0.956134	63	4	0.7687
ACSBG2	4	0.010877	0.035795	0.956134	64	2	0.19747
HNMT	4	0.01117	0.03676	0.956134	65	3	0.87998
CYP1B1	4	0.011432	0.037609	0.956134	66	1	-0.074004
PNPLA3	4	0.011928	0.038976	0.956134	67	3	0.64933
CHST14	4	0.011987	0.03916	0.956134	68	2	0.24699
B3GALT5	4	0.012117	0.039517	0.956134	69	3	0.69987
DHRS4L2	4	0.012542	0.040863	0.956134	70	2	-0.020124
NDUFB4	4	0.012677	0.04137	0.956134	71	4	0.53368
PIGX	4	0.012869	0.041934	0.956134	72	4	0.63337
MGAT4C	4	0.012932	0.042163	0.956134	73	2	0.36854
CTBS	4	0.013005	0.042302	0.956134	74	3	0.91659
CHST8	4	0.013106	0.042576	0.956134	75	4	0.59067
PLPPR2	4	0.013112	0.042609	0.956134	76	3	0.59722
CYP4F12	4	0.013193	0.042861	0.956134	77	4	0.35533

G6PC2	4	0.013215	0.042888	0.956134	78	4	0.5541
GUSB	4	0.013597	0.044133	0.956134	79	3	0.59655
CHKA	4	0.013651	0.044312	0.956134	80	2	0.057046
G6PC	4	0.013973	0.04535	0.956134	81	2	-0.092247
ACAA2	4	0.014205	0.04602	0.956134	82	1	-1.1162
UGT1A1	4	0.014759	0.047861	0.956134	83	2	0.34958
NDST2	4	0.01481	0.047995	0.956134	84	4	0.46871
HS3ST2	4	0.014895	0.048347	0.956134	85	2	0.24078
COX4I2	4	0.015256	0.049469	0.956134	86	3	0.65915
FHIT	4	0.015313	0.049631	0.956134	87	1	-0.22592
GOT2	4	0.015765	0.050864	0.956134	88	3	0.5978
COQ10B	4	0.015845	0.051087	0.956134	89	3	0.8155
ECHDC3	4	0.015862	0.051132	0.956134	90	3	0.59971
NDUFAF1	4	0.015867	0.051149	0.956134	91	3	0.50095
PDE1C	4	0.01601	0.051673	0.956134	92	4	0.44701
FMO4	4	0.016248	0.052388	0.956134	93	2	0.28415
DGKZ	4	0.016417	0.052879	0.956134	94	4	0.31139
UGT8	4	0.01642	0.05289	0.956134	95	3	0.62159
MARC1	4	0.016546	0.053202	0.956134	96	4	0.43742
A4GNT	4	0.016937	0.054347	0.956134	97	4	0.2451
UGP2	4	0.016973	0.054453	0.956134	98	2	0.6456
LGSN	4	0.01704	0.054637	0.956134	99	3	0.6959
ALOX5	4	0.017319	0.055357	0.956134	100	3	0.64603
MOCS2	4	0.017335	0.055385	0.956134	101	4	0.24628
PCYT1A	4	0.017403	0.055563	0.956134	102	2	0.20938
NAA60	4	0.017904	0.056735	0.956134	103	4	0.56642
TPK1	4	0.017979	0.056998	0.956134	104	3	0.81837
DPYS	4	0.018078	0.057204	0.956134	105	2	0.50548
ABHD12	4	0.018346	0.057952	0.956134	106	4	0.44114
MCAT	4	0.018374	0.058019	0.956134	107	4	0.25487
GCNT3	4	0.018631	0.058839	0.956134	108	1	-0.69574
PDE4A	4	0.019183	0.060369	0.956134	109	3	0.34514
CMAS	4	0.019314	0.060737	0.956134	110	4	0.64647
NADK	4	0.019378	0.060927	0.956134	111	4	0.91536
TK1	4	0.019466	0.06115	0.956134	112	2	0.39811
PNPO	4	0.019645	0.06173	0.956134	113	3	0.65883
ASPA	4	0.019731	0.061965	0.956134	114	2	0.51415
PLCE1	4	0.019735	0.061982	0.956134	115	2	0.34083
IMPA2	4	0.020286	0.063528	0.965808	116	2	0.2406
DLD	4	0.020355	0.06374	0.965808	117	3	0.80475
AK1	4	0.020537	0.064242	0.965808	118	3	0.45729
B3GLCT	4	0.020838	0.065219	0.968947	119	2	0.27958
ILVBL	4	0.021053	0.065911	0.968947	120	4	0.30387
PTGIS	4	0.021115	0.066089	0.968947	121	4	0.27936
UQCRFS1	4	0.021389	0.066837	0.970495	122	2	0.13162
PKLR	4	0.02194	0.068026	0.970495	123	2	0.13526

HPGD	4	0.022491	0.069142	0.970495	124	2	0.22764
DGKI	4	0.022822	0.069745	0.970495	125	3	0.61931
SGPL1	4	0.023015	0.07018	0.970495	126	3	0.85797
GFPT2	4	0.023041	0.070231	0.970495	127	1	-0.33203
PNPLA5	4	0.023799	0.071687	0.970495	128	3	0.49228
PANK1	4	0.024141	0.072446	0.970495	129	2	-0.056688
ACSL4	4	0.024294	0.072764	0.970495	130	2	0.50231
GAL3ST2	4	0.024587	0.07335	0.970495	131	3	0.59869
NUDT18	4	0.024691	0.073563	0.970495	132	1	-0.41138
NUDT16	4	0.025277	0.074774	0.970495	133	2	0.27576
D2HGDH	4	0.02579	0.075817	0.970495	134	2	0.23839
FUT8	4	0.026888	0.077916	0.970495	135	2	-0.062539
DGUOK	4	0.026937	0.078005	0.970495	136	3	0.63846
PDSS1	4	0.027367	0.078926	0.970495	137	3	0.78218
GDPD5	4	0.027985	0.080199	0.970495	138	3	0.4101
CHST10	4	0.028129	0.080528	0.970495	139	3	0.5376
HPSE2	4	0.02846	0.081119	0.970495	140	3	0.55529
ENTPD3	4	0.028533	0.081298	0.970495	141	3	0.46749
MTAP	4	0.028751	0.081717	0.970495	142	3	0.58463
PDE6D	4	0.028856	0.081962	0.970495	143	2	0.41771
CHID1	4	0.029067	0.082398	0.970495	144	3	0.56201
GMDS	4	0.029081	0.082442	0.970495	145	2	-0.0065075
LPCAT2	4	0.030176	0.084608	0.970495	146	2	-0.016778
XYLB	4	0.030436	0.085132	0.970495	147	3	0.6252
SULT2B1	4	0.030723	0.085757	0.970495	148	2	-0.0082992
PCK2	4	0.03127	0.086818	0.970495	149	2	0.45242
NAGPA	4	0.03165	0.087622	0.970495	150	3	0.52467
CYP4F22	4	0.032076	0.088615	0.970495	151	3	0.58584
DIO1	4	0.03209	0.088621	0.970495	152	3	0.56451
CPOX	4	0.032364	0.089156	0.970495	153	2	0.26448
DHRS3	4	0.032421	0.089246	0.970495	154	3	0.63673
NDUFA3	4	0.032495	0.089391	0.970495	155	3	0.54206
PNPLA8	4	0.032532	0.089447	0.970495	156	2	0.58699
GUCY2F	4	0.032643	0.089647	0.970495	157	2	0.32819
DHFR2	4	0.03328	0.090803	0.970495	158	3	0.72877
UGCG	4	0.033341	0.090881	0.970495	159	3	1.0186
PLCH2	4	0.033456	0.091154	0.970495	160	2	0.192
CYB5R4	4	0.033763	0.091724	0.970495	161	2	0.51097
NME2	4	0.034002	0.092215	0.970495	162	1	-0.19667
GAA	4	0.034326	0.092985	0.970495	163	3	0.55599
BCO2	4	0.034785	0.093755	0.970495	164	3	0.98546
DAD1	4	0.035264	0.094799	0.970495	165	3	0.50363
ELOVL5	4	0.035638	0.095647	0.970495	166	2	0.57131
B3GNT6	4	0.035708	0.095742	0.970495	167	3	0.50524
SRD5A1	4	0.036091	0.096546	0.970495	168	3	0.44642
BCAT2	4	0.036284	0.096987	0.970495	169	2	0.32521

UGT1A3	4	0.036634	0.097673	0.970495	170	2	0.34504
PON1	4	0.036728	0.097829	0.970495	171	2	0.053522
CHST7	4	0.037272	0.098895	0.970495	172	2	0.088605
VKORC1	4	0.03836	0.10111	0.970495	173	3	0.36611
ACE	4	0.038645	0.10168	0.970495	174	3	0.65329
GSTCD	4	0.038904	0.10226	0.970495	175	2	-0.075426
PGAM1	4	0.039176	0.10272	0.970495	176	3	0.6458
SOD1	4	0.039245	0.10282	0.970495	177	2	0.43846
ENTPD2	4	0.039446	0.10328	0.970495	178	3	0.47677
ECHDC1	4	0.039447	0.10328	0.970495	179	2	0.36027
PTS	4	0.039486	0.10335	0.970495	180	2	0.36985
FBP1	4	0.039728	0.1038	0.970495	181	3	0.60177
FTCD	4	0.039991	0.1043	0.970495	182	3	0.47554
PRG2	4	0.040534	0.10528	0.970495	183	1	-0.68143
PLA2G6	4	0.04058	0.10534	0.970495	184	3	0.65139
LIPA	4	0.040947	0.10602	0.970495	185	3	0.54374
DAO	4	0.041193	0.1065	0.970495	186	2	0.30728
PDE7B	4	0.041439	0.10694	0.970495	187	2	0.13406
B3GNT7	4	0.041619	0.10732	0.970495	188	2	0.08028
FAAH2	4	0.041809	0.10773	0.970495	189	2	0.2219
PDE3A	4	0.042555	0.109	0.970495	190	2	0.081645
FUT1	4	0.042704	0.10929	0.970495	191	2	0.24301
FDXR	4	0.042913	0.10967	0.970495	192	3	0.45687
DBT	4	0.043246	0.11033	0.970495	193	3	0.52431
ACBD5	4	0.043413	0.11075	0.970495	194	3	0.46255
KYNU	4	0.043787	0.11146	0.970495	195	1	-0.18055
ENTPD6	4	0.04487	0.11361	0.970495	196	1	-0.22828
SULF1	4	0.045374	0.11455	0.970495	197	3	0.43054
GGTLC1	4	0.045411	0.11462	0.970495	198	1	0.10421
OAS3	4	0.045447	0.1147	0.970495	199	3	0.46726
POFUT2	4	0.046492	0.11688	0.970495	200	3	0.43292
TALDO1	4	0.046935	0.11773	0.970495	201	3	0.85064
CYP20A1	4	0.047033	0.11794	0.970495	202	3	0.46036
GLA	4	0.047275	0.11841	0.970495	203	2	0.34358
MVD	4	0.047462	0.11874	0.970495	204	3	0.34979
CA2	4	0.047573	0.11893	0.970495	205	3	0.53294
IDI1	4	0.04769	0.11913	0.970495	206	3	0.48483
FXN	4	0.048652	0.121	0.970495	207	2	-0.005823
TKTL1	4	0.049066	0.12167	0.970495	208	3	0.41336
LIPH	4	0.049192	0.12198	0.970495	209	1	0.1039
RENBP	4	0.049731	0.12293	0.970495	210	1	-0.54538
ALG8	4	0.050182	0.12392	0.970495	211	3	0.62325
EPX	4	0.050808	0.12512	0.970495	212	1	-0.25628
CA13	4	0.051122	0.12572	0.970495	213	2	0.38249
LYZL1	4	0.051662	0.12686	0.970495	214	2	0.22403
B3GAT3	4	0.051732	0.12694	0.970495	215	3	0.39072

GCSH	4	0.051797	0.12713	0.970495	216	3	0.73864
C1GALT1	4	0.051811	0.12715	0.970495	217	3	0.7275
NQO2	4	0.051885	0.12733	0.970495	218	3	0.3259
GPD1L	4	0.051933	0.12743	0.970495	219	2	0.48452
VKORC1L1	4	0.052451	0.12834	0.970495	220	3	0.46211
GALNT14	4	0.052477	0.12838	0.970495	221	2	0.19585
NUDT19	4	0.052612	0.12864	0.970495	222	3	0.40354
GALK2	4	0.052887	0.12913	0.970495	223	3	0.52609
TYR	4	0.052961	0.12928	0.970495	224	3	0.60141
SULT1A3	4	0.053572	0.1304	0.970495	225	3	0.62852
HS3ST6	4	0.053583	0.13042	0.970495	226	3	0.38541
NEU3	4	0.053991	0.13118	0.970495	227	3	0.44171
ABAT	4	0.054954	0.13299	0.970495	228	2	0.41417
SCD	4	0.05511	0.13326	0.970495	229	1	-0.37938
MAN1C1	4	0.055646	0.13429	0.970495	230	2	0.10197
DOLK	4	0.056183	0.13516	0.970495	231	2	0.30386
CHPF	4	0.056392	0.13555	0.970495	232	3	0.42764
HPRT1	4	0.056632	0.13599	0.970495	233	3	0.63723
TSTA3	4	0.057255	0.13725	0.970495	234	3	0.42465
COX19	4	0.057488	0.13776	0.970495	235	3	0.57343
SGSH	4	0.058326	0.13938	0.970495	236	2	0.026581
RFK	4	0.05833	0.13939	0.970495	237	2	0.37224
NUDT10	4	0.058683	0.14006	0.970495	238	3	0.44657
MME	4	0.059286	0.14131	0.970495	239	3	0.63226
AADAT	4	0.059331	0.14138	0.970495	240	3	0.66207
CYP2W1	4	0.059397	0.14155	0.970495	241	2	0.25444
EDEM2	4	0.06005	0.14278	0.970495	242	2	0.55679
ABHD1	4	0.060766	0.14421	0.970495	243	3	0.40283
PRPSAP1	4	0.060854	0.14433	0.970495	244	3	0.5349
ATP13A4	4	0.060917	0.14446	0.970495	245	3	0.65537
CPT1B	4	0.061	0.14462	0.970495	246	2	0.56348
CYP1A2	4	0.061382	0.14537	0.970495	247	3	0.46664
LPCAT3	4	0.061534	0.14565	0.970495	248	1	0.11414
ADSL	4	0.061935	0.14636	0.970495	249	2	0.46518
OTC	4	0.062179	0.1468	0.970495	250	3	0.58581
GALNT17	4	0.062357	0.14713	0.970495	251	3	0.57172
TKT	4	0.062374	0.14718	0.970495	252	2	0.39936
NDUFA9	4	0.06252	0.1474	0.970495	253	2	0.36315
AASS	4	0.062602	0.14754	0.970495	254	1	-0.047006
PGLS	4	0.062714	0.14775	0.970495	255	3	0.37793
PRDX4	4	0.062893	0.14812	0.970495	256	3	0.48772
MBOAT2	4	0.063135	0.14855	0.970495	257	2	0.32382
CYBB	4	0.063669	0.14947	0.970495	258	2	0.126
GSTM2	4	0.064437	0.15106	0.970495	259	2	0.22847
SPTLC1	4	0.064607	0.15144	0.970495	260	3	0.49849
HMGCS2	4	0.064698	0.15158	0.970495	261	3	0.46331

MAN1B1	4	0.064734	0.15165	0.970495	262	2	0.26559
PTGES3	4	0.065031	0.15222	0.970495	263	2	0.28206
FUT4	4	0.065267	0.15266	0.970495	264	2	0.01744
ACAD9	4	0.065329	0.15278	0.970495	265	3	0.50357
CES5A	4	0.065887	0.15376	0.970495	266	3	0.4147
NDUFA5	4	0.066348	0.15457	0.970495	267	3	0.70699
NOS2	4	0.066441	0.15472	0.970495	268	3	0.40834
GCLM	4	0.066863	0.15553	0.970495	269	2	0.18032
GDPD2	4	0.067395	0.15649	0.970495	270	3	0.38082
ECH1	4	0.067885	0.15744	0.970495	271	2	0.33633
ALDH3A2	4	0.067926	0.15748	0.970495	272	2	0.32654
PLD1	4	0.068457	0.15844	0.970495	273	2	0.098353
PTGDS	4	0.069519	0.16032	0.970495	274	2	0.23582
ENTPD8	4	0.069713	0.16067	0.970495	275	2	0.095556
DSE	4	0.069724	0.1607	0.970495	276	3	0.45365
DGKK	4	0.069914	0.16105	0.970495	277	3	0.52524
GCLC	4	0.07005	0.16131	0.970495	278	2	-0.10035
HSD17B12	4	0.070487	0.16202	0.970495	279	3	0.63609
ACACB	4	0.07058	0.16217	0.970495	280	2	-0.069399
ST6GAL2	4	0.070634	0.16228	0.970495	281	3	0.34879
PLPP5	4	0.070679	0.16236	0.970495	282	3	0.56597
HSD17B6	4	0.07111	0.16312	0.970495	283	2	0.078257
FUT5	4	0.071251	0.16339	0.970495	284	2	0.28081
CYP24A1	4	0.071448	0.1638	0.970495	285	3	0.65417
PPA2	4	0.07164	0.16413	0.970495	286	1	-3.6529
RETSAT	4	0.071715	0.16425	0.970495	287	2	0.21546
HYAL4	4	0.07187	0.16451	0.970495	288	2	0.49151
NT5DC2	4	0.071932	0.16462	0.970495	289	3	0.46861
ARSI	4	0.072025	0.1648	0.970495	290	2	0.39508
DDHD2	4	0.072169	0.16507	0.970495	291	1	-0.21449
SMPDL3B	4	0.072515	0.16579	0.970495	292	3	0.54553
UGT2B15	4	0.072699	0.16612	0.970495	293	1	-0.070234
GAPDHS	4	0.072807	0.1663	0.970495	294	3	0.48646
RPE65	4	0.073228	0.16714	0.970495	295	2	0.38043
AKR7A2	4	0.073296	0.16726	0.970495	296	3	0.38483
SMPD4	4	0.073757	0.16815	0.970495	297	2	0.38037
DGKG	4	0.073787	0.1682	0.970495	298	3	0.48326
AUH	4	0.074378	0.16919	0.970495	299	3	0.42291
ALG2	4	0.07527	0.17088	0.970495	300	3	0.3973
FH	4	0.075342	0.17102	0.970495	301	2	-0.14118
PRPS1	4	0.07537	0.17107	0.970495	302	3	0.48684
CHST11	4	0.07547	0.17129	0.970495	303	3	0.36756
ADA2	4	0.07587	0.17208	0.970495	304	1	-1.6964
CYP4B1	4	0.076738	0.17374	0.970495	305	2	0.29636
DPYD	4	0.076772	0.17379	0.970495	306	3	0.46104
PLA2G1B	4	0.077276	0.17473	0.970495	307	3	0.32783

BDH1	4	0.077376	0.17491	0.970495	308	2	0.27072
CAT	4	0.077453	0.17503	0.970495	309	1	-0.0684
ATP13A1	4	0.07798	0.176	0.970495	310	2	-3.8334
ABHD14A	4	0.077985	0.17602	0.970495	311	3	0.37952
HIBCH	4	0.078507	0.17698	0.970495	312	3	0.24266
MPST	4	0.079033	0.17788	0.970495	313	1	-0.042657
CYB5D1	4	0.0793	0.17837	0.970495	314	3	0.37593
DPM1	4	0.07956	0.17883	0.970495	315	2	0.32763
GALM	4	0.079946	0.17949	0.970495	316	2	0.42115
GAL3ST3	4	0.080032	0.17969	0.970495	317	3	0.3025
PIGB	4	0.080269	0.18013	0.970495	318	2	0.24911
ADCY2	4	0.080445	0.18049	0.970495	319	3	0.3552
FMO2	4	0.080612	0.18075	0.970495	320	1	-0.16067
KDSR	4	0.081138	0.18166	0.970495	321	3	0.29731
HMGCL	4	0.08138	0.18214	0.970495	322	3	0.53508
MAOA	4	0.081663	0.18264	0.970495	323	3	0.46533
CA4	4	0.081901	0.18303	0.970495	324	3	0.38877
PON3	4	0.082058	0.18333	0.970495	325	2	0.16551
SMPD3	4	0.082385	0.18405	0.970495	326	2	0.31411
UROC1	4	0.082549	0.18434	0.970495	327	3	0.50874
DHRS11	4	0.082712	0.18464	0.970495	328	2	0.39386
NDUFV1	4	0.083238	0.18559	0.970495	329	3	0.22568
DPEP3	4	0.083372	0.18586	0.970495	330	3	0.33287
ADCY9	4	0.083478	0.18608	0.970495	331	3	0.32787
ALDH1A2	4	0.083795	0.18669	0.970495	332	3	0.42288
BCAT1	4	0.084287	0.18757	0.970495	333	2	0.21082
SRR	4	0.084811	0.18846	0.970495	334	3	0.1977
PLCG1	4	0.085335	0.18948	0.970495	335	2	0.26359
ACMSD	4	0.085822	0.19042	0.970495	336	3	0.32232
DHRS7	4	0.085859	0.19047	0.970495	337	2	0.45742
ASL	4	0.085929	0.19061	0.970495	338	3	0.49335
HSD17B8	4	0.086014	0.19079	0.970495	339	2	0.19932
A4GALT	4	0.086383	0.19153	0.970495	340	2	0.23055
CHSY3	4	0.087768	0.19402	0.970495	341	3	0.58933
POMGNT1	4	0.087952	0.19433	0.970495	342	2	0.27587
COX4I1	4	0.088474	0.19527	0.970495	343	2	0.32169
GLYAT	4	0.088523	0.19537	0.970495	344	2	0.58097
GDPD3	4	0.089189	0.19647	0.970495	345	3	0.43535
INPP5F	4	0.089519	0.19712	0.970495	346	3	0.30855
MOGAT3	4	0.089849	0.19772	0.970495	347	3	0.37126
PCYT2	4	0.090041	0.19802	0.970495	348	1	-0.40098
MTHFD1	4	0.090734	0.19917	0.970495	349	3	0.40948
GANAB	4	0.090845	0.19938	0.970495	350	3	0.30977
HSD17B13	4	0.090956	0.19959	0.970495	351	3	0.50866
BDH2	4	0.091084	0.19988	0.970495	352	2	0.2374
AKR1C3	4	0.091229	0.20017	0.970495	353	2	0.18284

PLA2G2C	4	0.091289	0.20026	0.970495	354	3	0.36111
DHRSX	4	0.091605	0.20075	0.970495	355	1	-0.56758
PIGM	4	0.091911	0.20127	0.970495	356	2	0.29776
LPGAT1	4	0.092081	0.20151	0.970495	357	2	0.36191
CYB5RL	4	0.092647	0.20252	0.970495	358	1	-0.32309
SDR42E1	4	0.093108	0.20332	0.970495	359	2	0.33297
CANT1	4	0.093192	0.20345	0.970495	360	3	0.43457
ALOX5AP	4	0.094139	0.20525	0.970495	361	2	0.42987
HAGH	4	0.094208	0.20536	0.970495	362	1	-0.044249
AGPAT3	4	0.094728	0.20636	0.970495	363	1	-0.0045901
AK7	4	0.095347	0.20754	0.970495	364	2	0.30449
HMGCS1	4	0.095689	0.20811	0.970495	365	3	0.37555
PNPLA6	4	0.095693	0.20811	0.970495	366	2	0.40031
HEXB	4	0.095767	0.20825	0.970495	367	1	-0.92686
ST8SIA4	4	0.096262	0.2091	0.970495	368	3	0.47985
TDO2	4	0.096805	0.21008	0.970495	369	1	-0.2871
B4GALNT1	4	0.096908	0.21031	0.970495	370	2	0.22997
ALDH7A1	4	0.097067	0.21052	0.970495	371	3	0.31875
ARSB	4	0.097876	0.21186	0.970495	372	3	0.48721
CYP3A7	4	0.098304	0.21265	0.970495	373	2	0.51402
NDUFS3	4	0.098339	0.21269	0.970495	374	3	0.28945
PI4K2A	4	0.098361	0.21272	0.970495	375	1	-1.272
PANK4	4	0.098655	0.2133	0.970495	376	2	-0.036084
SULT6B1	4	0.099884	0.21535	0.970495	377	2	0.27506
OXA1L	4	0.10021	0.21597	0.970495	378	3	0.57609
PIGY	4	0.10094	0.21726	0.970495	379	2	0.28681
MPI	4	0.10147	0.21824	0.970495	380	2	0.12185
B4GAT1	4	0.10174	0.2187	0.970495	381	3	0.40671
ACOT11	4	0.10198	0.21914	0.970495	382	1	0.048102
PGK1	4	0.10218	0.21958	0.970495	383	2	0.35972
ACAT1	4	0.10254	0.22031	0.970495	384	2	-0.16177
GALNT5	4	0.10317	0.22139	0.970495	385	3	0.38126
ETFDH	4	0.10325	0.22152	0.970495	386	2	0.26144
OGFOD1	4	0.10353	0.22195	0.970495	387	3	0.42178
HYAL1	4	0.10436	0.22337	0.970495	388	3	0.40207
PPA1	4	0.10456	0.22367	0.970495	389	1	-0.42276
PON2	4	0.1046	0.22374	0.970495	390	3	0.45237
GCH1	4	0.10504	0.22449	0.970495	391	2	0.35867
PPOX	4	0.10508	0.22457	0.970495	392	1	-0.89821
NUDT14	4	0.1054	0.22512	0.970495	393	2	0.29283
PTER	4	0.10611	0.2264	0.970495	394	1	-0.74275
CYP2C8	4	0.10611	0.22641	0.970495	395	2	0.31698
ACSL1	4	0.10648	0.22709	0.970495	396	2	0.2667
SULT1C4	4	0.10666	0.22737	0.970495	397	3	0.50146
PLA1A	4	0.1069	0.2278	0.970495	398	3	0.42886
CYP3A43	4	0.10727	0.22842	0.970495	399	3	0.51102

AGPAT2	4	0.10738	0.22862	0.970495	400	2	0.31742
PANK2	4	0.10788	0.22945	0.970495	401	3	0.31784
ENPP7	4	0.108	0.22971	0.970495	402	3	0.46238
SPHK2	4	0.10874	0.23085	0.970495	403	3	0.48478
ABHD14B	4	0.10899	0.23124	0.970495	404	3	0.43213
CTPS1	4	0.10911	0.23151	0.970495	405	3	0.47222
NDUFA1	4	0.10919	0.23167	0.970495	406	3	0.31341
CYP51A1	4	0.10923	0.23172	0.970495	407	3	0.35629
BST1	4	0.10971	0.23248	0.970495	408	2	-0.5333
ALAS1	4	0.10992	0.23282	0.970495	409	2	0.29286
ENPP5	4	0.11022	0.23332	0.970495	410	1	-0.59801
PIGT	4	0.11047	0.23375	0.970495	411	2	0.096334
PLD3	4	0.11047	0.23375	0.970495	412	3	0.39523
PIGU	4	0.11073	0.23431	0.970495	413	2	-0.039833
NME6	4	0.11109	0.23492	0.970495	414	3	0.32742
CES1	4	0.11157	0.23582	0.970495	415	2	-0.0065763
TPH1	4	0.11159	0.23586	0.970495	416	3	0.47067
HCCS	4	0.11171	0.23607	0.970495	417	3	0.48364
COX11	4	0.11176	0.23615	0.970495	418	2	0.05102
GSTM3	4	0.11184	0.23631	0.970495	419	3	0.31971
COQ2	4	0.11227	0.237	0.970495	420	2	0.21767
INPP5J	4	0.11248	0.23734	0.970495	421	2	0.18939
IVD	4	0.11285	0.23796	0.970495	422	2	0.099401
ENPP4	4	0.11381	0.23952	0.970495	423	2	0.26947
CNP	4	0.11433	0.24038	0.970495	424	2	-0.1096
EXTL3	4	0.11435	0.24042	0.970495	425	3	0.29377
LYPLA2	4	0.1147	0.24097	0.970495	426	2	0.090894
ST3GAL3	4	0.11483	0.24122	0.970495	427	1	-0.18105
ALDH6A1	4	0.11534	0.2421	0.970495	428	2	0.53504
CYP2J2	4	0.11575	0.24269	0.970495	429	3	0.34284
COX6A2	4	0.11585	0.24288	0.970495	430	2	0.24988
NEU4	4	0.116	0.24316	0.970495	431	3	0.26757
ESD	4	0.11636	0.24366	0.970495	432	2	0.10782
CDIPT	4	0.11639	0.2437	0.970495	433	3	0.31597
XYLT2	4	0.11687	0.24456	0.970495	434	2	0.30828
ACOT6	4	0.11693	0.24466	0.970495	435	2	0.28049
CMBL	4	0.1173	0.2452	0.970495	436	3	0.47346
NT5C1A	4	0.11738	0.24536	0.970495	437	2	-0.18828
RDH11	4	0.11741	0.2454	0.970495	438	3	0.32376
ASAH2	4	0.11786	0.24614	0.970495	439	2	0.024718
UGT2A3	4	0.11805	0.24651	0.970495	440	3	0.38457
MAN2B2	4	0.11818	0.24665	0.970495	441	3	0.41888
HSD17B4	4	0.1184	0.24703	0.970495	442	2	0.10598
RDH16	4	0.11891	0.24801	0.970495	443	1	-0.33295
GSTM1	4	0.11896	0.2481	0.970495	444	3	0.40578
UGDH	4	0.11909	0.24838	0.970495	445	3	0.38104

IAH1	4	0.11942	0.24896	0.970495	446	1	0.11513
PIK3CG	4	0.11947	0.24905	0.970495	447	3	0.32461
NPR3	4	0.11992	0.24984	0.970495	448	3	0.39738
GLRX5	4	0.11993	0.24985	0.970495	449	2	0.15307
MANBAL	4	0.11999	0.24995	0.970495	450	3	0.38005
PLCD4	4	0.1203	0.25048	0.970495	451	2	0.20711
NDUFS5	4	0.12044	0.2507	0.970495	452	2	0.05974
PI4KA	4	0.12077	0.25131	0.970495	453	3	0.40939
UQCRQ	4	0.12105	0.25179	0.970495	454	2	0.11722
XDH	4	0.12117	0.25195	0.970495	455	3	0.32917
PRPSAP2	4	0.12143	0.2524	0.970495	456	2	-0.020031
ENOX2	4	0.12182	0.25298	0.970495	457	3	0.50265
ETNK1	4	0.12196	0.25325	0.970495	458	2	0.36225
DDHD1	4	0.12219	0.25366	0.970495	459	2	0.407
COQ6	4	0.12238	0.25401	0.970495	460	2	0.043075
SULT2A1	4	0.12247	0.25418	0.970495	461	2	-0.66944
B3GALNT1	4	0.12261	0.25439	0.970495	462	3	0.32268
CYP2A7	4	0.12353	0.25595	0.970495	463	3	0.29201
UPRT	4	0.12379	0.25643	0.970495	464	3	0.23864
FBP2	4	0.1239	0.25661	0.970495	465	2	0.32906
GALC	4	0.12419	0.25705	0.970495	466	3	0.32548
HPSE	4	0.1245	0.25754	0.970495	467	2	-0.13155
ASPG	4	0.12459	0.25766	0.970495	468	3	0.26877
SCD5	4	0.12472	0.25784	0.970495	469	3	0.35068
AMD1	4	0.12485	0.25803	0.970495	470	3	0.35176
SRXN1	4	0.12504	0.25832	0.970495	471	2	0.31596
DHODH	4	0.12512	0.25844	0.970495	472	3	0.27602
GPHN	4	0.12551	0.25914	0.970495	473	3	0.48693
PNMT	4	0.12561	0.25931	0.970495	474	3	0.37156
ENTPD5	4	0.12657	0.26089	0.97223	475	2	-0.26003
PDHX	4	0.12699	0.26157	0.97223	476	3	0.29813
CERS3	4	0.12703	0.26164	0.97223	477	2	-0.065342
CROT	4	0.12753	0.26248	0.97223	478	2	-0.028222
NUDT9	4	0.12754	0.26251	0.97223	479	2	0.099053
ALDH18A1	4	0.12807	0.26339	0.973463	480	3	0.3532
FUT10	4	0.12855	0.26411	0.974095	481	1	-0.63851
ABHD12B	4	0.12915	0.26507	0.975607	482	3	0.43921
PDE7A	4	0.13118	0.26864	0.975829	483	3	0.26827
RDH14	4	0.13146	0.26916	0.975829	484	3	0.39023
ATIC	4	0.13158	0.26936	0.975829	485	3	0.26498
CNDP1	4	0.13187	0.26984	0.975829	486	3	0.33201
FOXRED1	4	0.13208	0.27025	0.975829	487	2	0.14366
INPP5K	4	0.13255	0.2711	0.975829	488	3	0.34473
SDHC	4	0.13258	0.27112	0.975829	489	1	-0.87903
CH25H	4	0.13338	0.27251	0.975829	490	3	0.41946
DBH	4	0.13412	0.27385	0.975829	491	2	0.26614

PLA2G2F	4	0.13549	0.27588	0.975829	492	2	0.31757
COX5B	4	0.13573	0.27624	0.975829	493	3	0.22852
CYB5R1	4	0.1361	0.27683	0.975829	494	2	0.23974
ABHD3	4	0.13761	0.27927	0.975829	495	1	-0.37439
OXCT1	4	0.13811	0.28024	0.975829	496	2	0.28078
BPNT1	4	0.13825	0.2805	0.975829	497	3	0.34185
PGK2	4	0.13825	0.2805	0.975829	498	2	0.14268
PDE10A	4	0.13839	0.28073	0.975829	499	3	0.31163
PMVK	4	0.13861	0.2811	0.975829	500	2	0.24033
GMPPA	4	0.13944	0.28242	0.975829	501	2	0.2622
ADSSL1	4	0.13964	0.28281	0.975829	502	2	0.23745
PRODH2	4	0.1398	0.28309	0.975829	503	3	0.27939
GLUD2	4	0.13994	0.28334	0.975829	504	3	0.36444
AOC3	4	0.14008	0.28356	0.975829	505	3	0.243
HSD17B1	4	0.14011	0.28365	0.975829	506	2	0.10252
ACAD8	4	0.14061	0.28446	0.975829	507	1	0.10142
LDHA	4	0.14093	0.28496	0.975829	508	3	0.25159
FUCA1	4	0.14107	0.2853	0.975829	509	3	0.37145
GCNT4	4	0.14193	0.28667	0.975829	510	3	0.2865
CNDP2	4	0.1425	0.28769	0.975829	511	3	0.31778
B4GALT2	4	0.14261	0.28797	0.975829	512	3	0.22819
PLA2G2D	4	0.14262	0.28801	0.975829	513	2	-0.00903
KLB	4	0.14293	0.28852	0.975829	514	3	0.2063
GTDC1	4	0.14307	0.28873	0.975829	515	3	0.34925
PLA2G4C	4	0.14322	0.28899	0.975829	516	2	-0.020268
HGLibA	50	0.14329	0.52297	0.990873	517	22	0.12482
DHRS4L1	4	0.14361	0.28967	0.975829	518	1	-0.22971
GLCE	4	0.14364	0.28974	0.975829	519	3	0.35413
PI4KB	4	0.14407	0.29045	0.975829	520	3	0.32252
FAHD2B	4	0.1441	0.29046	0.975829	521	2	0.2008
PFKFB2	4	0.14442	0.29102	0.975829	522	2	0.098412
ACYP1	4	0.14482	0.29164	0.975829	523	2	0.35128
ECHDC2	4	0.1451	0.29214	0.975829	524	2	0.058981
PDSS2	4	0.14537	0.29267	0.975829	525	3	0.37518
B3GAT2	4	0.1456	0.29297	0.975829	526	1	-0.28529
MANEA	4	0.14603	0.29365	0.975829	527	2	0.11919
PIP5K1C	4	0.14609	0.29379	0.975829	528	3	0.38257
TPMT	4	0.1461	0.2938	0.975829	529	1	-0.57867
ARG1	4	0.14624	0.29402	0.975829	530	3	0.29956
CPS1	4	0.14653	0.29446	0.975829	531	3	0.27561
NT5C	4	0.14659	0.29456	0.975829	532	2	0.17421
B4GALT5	4	0.14663	0.29467	0.975829	533	2	0.19075
STS	4	0.14667	0.29472	0.975829	534	3	0.25282
ALG10	4	0.14683	0.29498	0.975829	535	2	0.25029
FADS3	4	0.14711	0.29547	0.975829	536	3	0.26928
SQOR	4	0.14754	0.29611	0.975829	537	3	0.17233

ACAD11	4	0.14804	0.29702	0.975829	538	2	0.26
PIGH	4	0.14808	0.29707	0.975829	539	1	-0.46497
DUOX1	4	0.14827	0.29731	0.975829	540	3	0.29434
FDX2	4	0.14885	0.29821	0.975829	541	2	-0.067217
CHST5	4	0.14908	0.29858	0.975829	542	1	-0.099209
BCKDHB	4	0.14915	0.29868	0.975829	543	3	0.39978
FUT2	4	0.14959	0.2994	0.975829	544	3	0.2021
LARGE2	4	0.14966	0.29948	0.975829	545	2	0.15717
PCBD2	4	0.14987	0.29979	0.975829	546	2	-0.50671
CHST1	4	0.15068	0.30099	0.977194	547	2	0.023358
THTPA	4	0.15129	0.3021	0.977194	548	2	0.0082049
GALNT11	4	0.1518	0.30291	0.977194	549	3	0.39522
LYPLA1	4	0.15209	0.30336	0.977194	550	3	0.35261
CBR1	4	0.15231	0.30372	0.977194	551	2	0.22133
PC	4	0.15254	0.30405	0.977194	552	3	0.18435
PLD5	4	0.15254	0.30406	0.977194	553	1	-0.22675
ST6GALNAC6	4	0.15298	0.30486	0.977987	554	3	0.25185
SYNJ1	4	0.15353	0.30581	0.978444	555	1	-0.40946
SCCPDH	4	0.15372	0.30614	0.978444	556	3	0.37639
ST6GALNAC5	4	0.15402	0.30666	0.978444	557	3	0.33387
B3GALT2	4	0.15462	0.30751	0.979389	558	3	0.35851
PLCL1	4	0.15517	0.30845	0.980615	559	2	0.43121
XYLT1	4	0.156	0.3099	0.982157	560	2	0.23711
SPHK1	4	0.1565	0.31077	0.982157	561	2	-0.014101
ASMT	4	0.15732	0.31212	0.982157	562	3	0.27506
MAN2C1	4	0.15748	0.31236	0.982157	563	2	-0.12834
ALDH2	4	0.15867	0.31446	0.982157	564	3	0.26251
HAS1	4	0.15883	0.31476	0.982157	565	3	0.22921
DGKE	4	0.15896	0.315	0.982157	566	1	0.089841
PLA2G4F	4	0.15909	0.3152	0.982157	567	2	0.28163
B4GALNT4	4	0.15913	0.31529	0.982157	568	3	0.20052
UGT2A1	4	0.15945	0.31585	0.982157	569	1	-1.32
PRDX1	4	0.15991	0.31659	0.982157	570	2	0.23502
UST	4	0.16019	0.31701	0.982157	571	3	0.2754
GPD2	4	0.16092	0.318	0.982157	572	2	0.17898
BPGM	4	0.16095	0.31804	0.982157	573	2	0.32685
INPP1	4	0.16157	0.31865	0.982157	574	2	-0.091406
NIT2	4	0.1619	0.31893	0.982157	575	1	-0.52955
A1CF	4	0.1624	0.31936	0.982157	576	1	-0.42781
SCP2	4	0.16324	0.32017	0.982157	577	2	0.15388
LIPI	4	0.16338	0.3203	0.982157	578	1	-0.11489
LYZL6	4	0.16386	0.32079	0.982157	579	2	0.089233
ACSS3	4	0.16484	0.32187	0.982157	580	2	0.10459
IP6K1	4	0.16533	0.3224	0.982157	581	1	-0.20226
SI	4	0.16554	0.32255	0.982157	582	2	0.20772
MTHFD2	4	0.16575	0.32279	0.982157	583	2	0.057014

HSD11B1L	4	0.16722	0.3243	0.982157	584	2	0.22014
LYPLAL1	4	0.16778	0.32492	0.982157	585	1	0.07424
MTR	4	0.16785	0.32501	0.982157	586	2	0.038188
LPIN2	4	0.16924	0.32645	0.982157	587	2	0.13436
CHSY1	4	0.16973	0.32691	0.982157	588	2	0.29081
DHRS13	4	0.1707	0.32783	0.982157	589	1	0.025634
PIGC	4	0.17119	0.3282	0.982157	590	2	0.048345
POFUT1	4	0.17122	0.32825	0.982157	591	2	0.24279
SGPP2	4	0.17186	0.32885	0.982157	592	2	0.36568
GADL1	4	0.17292	0.32974	0.982157	593	2	0.30398
PLPP1	4	0.17355	0.33035	0.982157	594	2	0.56011
NDUFA8	4	0.17362	0.33037	0.982157	595	2	0.14972
ACSF2	4	0.1741	0.33091	0.982157	596	1	-0.50788
SMPD2	4	0.17507	0.33179	0.982157	597	2	0.084037
COQ10A	4	0.17632	0.33293	0.982157	598	2	0.10858
ADK	4	0.17701	0.33356	0.982157	599	1	-0.38946
PNPLA2	4	0.1776	0.33411	0.982157	600	2	0.28278
NT5C3B	4	0.17867	0.33512	0.982157	601	2	0.28288
SCLY	4	0.17889	0.33533	0.982157	602	2	0.36913
LYZ	4	0.17943	0.33591	0.982157	603	2	-0.09339
ACSF3	4	0.17991	0.33638	0.982157	604	1	-0.23698
B3GALT1	4	0.17996	0.33645	0.982157	605	2	0.37468
ALOX15	4	0.18018	0.33664	0.982157	606	2	0.28934
NAT14	4	0.18039	0.33687	0.982157	607	2	0.072966
CUBN	4	0.18039	0.33687	0.982157	608	2	0.21308
ENPP3	4	0.18087	0.33741	0.982157	609	2	-0.0082446
NMNAT2	4	0.18125	0.3378	0.982157	610	2	-0.11994
PIGZ	4	0.18168	0.33825	0.982157	611	2	0.053259
AGPS	4	0.18232	0.33899	0.982157	612	1	-0.31091
CYB561A3	4	0.18319	0.33968	0.982157	613	2	0.076817
AGPAT1	4	0.18328	0.33977	0.982157	614	1	-0.24338
ALOX15B	4	0.18362	0.34009	0.982157	615	2	0.13922
DPEP1	4	0.18569	0.34202	0.982157	616	1	-0.040599
HK3	4	0.18665	0.343	0.982157	617	1	-0.12779
ENPP6	4	0.18808	0.34425	0.982157	618	1	-0.010149
DAGLB	4	0.18952	0.34557	0.982157	619	1	0.13174
CYCS	4	0.1897	0.34571	0.982157	620	2	-0.18009
ACACA	4	0.19048	0.34641	0.982157	621	1	-0.1877
ARSK	4	0.19143	0.3475	0.982157	622	2	-0.11865
PLPP6	4	0.1921	0.34805	0.982157	623	2	0.19789
SIAE	4	0.19286	0.34876	0.982157	624	1	-0.13919
PLCXD2	4	0.19298	0.34889	0.982157	625	2	0.22907
ADA	4	0.19382	0.34966	0.982157	626	2	-0.34927
PDE6C	4	0.19429	0.35017	0.982157	627	1	-0.039033
ARG2	4	0.19452	0.35038	0.982157	628	2	0.16468
SUCLA2	4	0.19628	0.35209	0.982157	629	2	0.21026

ST6GALNAC2	4	0.19715	0.35293	0.982157	630	1	-0.19232
PDE12	4	0.19826	0.3539	0.982157	631	2	-0.074407
HS3ST5	4	0.19848	0.35409	0.982157	632	2	0.10564
ALDOC	4	0.19937	0.35494	0.982157	633	2	0.17249
NDST3	4	0.19981	0.35534	0.982157	634	2	0.28624
RRM2B	4	0.19999	0.35552	0.982157	635	1	-0.01391
ENTPD4	4	0.20025	0.35574	0.982157	636	2	0.33174
ALAS2	4	0.20047	0.35591	0.982157	637	1	0.17459
SMPD1	4	0.20047	0.35591	0.982157	638	2	-0.0092741
DMGDH	4	0.20136	0.35667	0.982157	639	2	0.24923
NDUFB11	4	0.20189	0.35716	0.982157	640	1	-0.064866
LPCAT1	4	0.20269	0.35795	0.982157	641	2	-0.063541
CERS4	4	0.20291	0.35812	0.982157	642	2	0.20128
IDH1	4	0.20313	0.35832	0.982157	643	2	0.26177
DDAH2	4	0.2033	0.35849	0.982157	644	1	-0.19038
GMPS	4	0.20378	0.35896	0.982157	645	1	-0.29117
ACE2	4	0.2038	0.35896	0.982157	646	2	0.33038
OAT	4	0.20472	0.35982	0.982157	647	1	-0.066206
AMT	4	0.20603	0.36112	0.982157	648	2	0.090043
LPL	4	0.20613	0.36121	0.982157	649	1	-0.65383
PDE8A	4	0.20661	0.36168	0.982157	650	1	-1.2099
ECI2	4	0.20759	0.36272	0.982157	651	2	0.32092
GANC	4	0.20802	0.36312	0.982157	652	1	-0.48199
LIAS	4	0.20849	0.36357	0.982157	653	2	-0.055498
PDE9A	4	0.20916	0.36421	0.982157	654	2	0.3443
PMM1	4	0.20943	0.36449	0.982157	655	1	-0.39215
EBP	4	0.21073	0.36574	0.982157	656	2	0.3604
ACOT9	4	0.2114	0.36635	0.982157	657	2	0.3296
MBOAT4	4	0.21177	0.36667	0.982157	658	1	-0.59367
NDST4	4	0.21224	0.36704	0.982157	659	1	-0.38006
ITPKA	4	0.21365	0.3683	0.982157	660	2	0.21577
HADHA	4	0.21411	0.36869	0.982157	661	2	-0.088341
IDO2	4	0.21546	0.37003	0.982157	662	2	-0.019947
PFKM	4	0.21551	0.3701	0.982157	663	2	-0.14096
NME3	4	0.21568	0.37027	0.982157	664	2	0.27676
ALDH1A1	4	0.21598	0.3705	0.982157	665	1	-0.15362
CYP2C19	4	0.21704	0.37152	0.982157	666	2	0.16621
ADCY10	4	0.21738	0.37201	0.982157	667	2	0.27721
RBKS	4	0.21862	0.37317	0.982157	668	2	-0.058705
FAR2	4	0.21885	0.3733	0.982157	669	2	0.1794
CYP2F1	4	0.21908	0.37346	0.982157	670	2	0.27874
ARSE	4	0.21924	0.37362	0.982157	671	2	0.13174
ADCY1	4	0.2193	0.37369	0.982157	672	2	0.080299
ADCY8	4	0.21953	0.37388	0.982157	673	2	0.24253
ADCY4	4	0.21971	0.37404	0.982157	674	1	-0.49508
IYD	4	0.21998	0.37425	0.982157	675	2	0.12101

ARSJ	4	0.22017	0.37443	0.982157	676	1	-0.19851
PFKFB4	4	0.2211	0.37534	0.982157	677	1	-0.35062
ACOX3	4	0.22203	0.37618	0.982157	678	1	-0.34725
SULT1A2	4	0.22317	0.3772	0.982157	679	2	0.23127
GRXCR2	4	0.22388	0.37786	0.982157	680	1	0.12848
BHMT2	4	0.22408	0.37802	0.982157	681	2	0.367
PGD	4	0.22454	0.3784	0.982157	682	2	0.31374
FECH	4	0.22522	0.37901	0.982157	683	2	0.064778
RDH8	4	0.22527	0.37905	0.982157	684	2	0.1836
QPRT	4	0.22573	0.37946	0.982157	685	1	0.071805
PDE4B	4	0.22712	0.38079	0.982157	686	1	-0.053997
PPCDC	4	0.22758	0.38135	0.982157	687	1	-0.18913
ADH7	4	0.22804	0.38183	0.982157	688	2	-0.19142
PRPS2	4	0.22842	0.38218	0.982157	689	2	0.068984
GAMT	4	0.22911	0.38283	0.982157	690	2	-0.05752
ACOT4	4	0.22943	0.38321	0.982157	691	1	0.13166
ACSL5	4	0.22989	0.3837	0.982157	692	2	-0.42152
PIGK	4	0.23072	0.38451	0.982157	693	2	-0.21889
ADH4	4	0.23081	0.38461	0.982157	694	1	-0.5856
MSRB2	4	0.23127	0.38508	0.982157	695	2	0.037259
DDAH1	4	0.23265	0.38648	0.982157	696	1	-0.76608
COASY	4	0.23302	0.38686	0.982157	697	2	0.0003766<br>9
KL	4	0.2331	0.38695	0.982157	698	2	0.084237
FDPS	4	0.23348	0.38731	0.982157	699	2	0.28104
HMBS	4	0.23356	0.3874	0.982157	700	1	0.10921
ALOXE3	4	0.23371	0.38755	0.982157	701	2	0.07795
HSDL2	4	0.23448	0.38827	0.982592	702	1	-0.28247
ST8SIA1	4	0.23671	0.39044	0.985458	703	2	0.088818
GLUL	4	0.23677	0.39052	0.985458	704	1	-0.13266
AOC2	4	0.23809	0.3918	0.985638	705	2	0.11508
PSPH	4	0.23833	0.39206	0.985638	706	2	0.20162
SOAT2	4	0.23948	0.39319	0.985638	707	2	0.14846
ABHD2	4	0.24111	0.39462	0.985638	708	2	0.16653
NUDT7	4	0.24133	0.39485	0.985638	709	1	0.27499
PIK3C2B	4	0.24134	0.39486	0.985638	710	2	0.16798
DHTKD1	4	0.2427	0.39614	0.985638	711	1	-0.23111
HPD	4	0.24315	0.39654	0.985638	712	1	-0.20518
NQO1	4	0.24361	0.39699	0.985638	713	2	-0.19652
UROD	4	0.24483	0.39818	0.985638	714	2	0.30328
GALNS	4	0.24497	0.39831	0.985638	715	1	-0.60753
RDH13	4	0.24633	0.39962	0.985638	716	2	0.10779
MGAM	4	0.24669	0.4	0.985638	717	2	0.22113
PYROXD1	4	0.24678	0.40007	0.985638	718	1	-0.73021
FUCA2	4	0.24723	0.40048	0.985638	719	2	-0.37508
IP6K2	4	0.24769	0.40094	0.985638	720	2	0.10649

NME1	4	0.24859	0.40172	0.985638	721	1	-0.30007
ALDH4A1	4	0.24879	0.4019	0.985638	722	2	0.18982
PDE6H	4	0.24949	0.4025	0.985638	723	1	-0.59533
ODC1	4	0.25043	0.40347	0.985638	724	2	-0.38715
FUK	4	0.25113	0.4041	0.985638	725	2	-0.082972
GPAT3	4	0.2513	0.40429	0.985638	726	2	0.21044
INMT	4	0.25137	0.40434	0.985638	727	2	0.22207
PCCB	4	0.2522	0.4052	0.985638	728	1	-0.023324
MMACHC	4	0.25254	0.40547	0.985638	729	2	0.1704
GGTLC2	4	0.25301	0.40589	0.985638	730	2	0.051284
DCK	4	0.25418	0.40704	0.985638	731	2	0.37035
LIPG	4	0.25442	0.40731	0.985638	732	2	0.30605
OGDH	4	0.25559	0.4084	0.985638	733	2	0.11576
GALNTL6	4	0.25606	0.40886	0.985638	734	2	0.16686
MTHFR	4	0.25625	0.40909	0.985638	735	1	-0.033301
ACYP2	4	0.25714	0.41002	0.985638	736	2	0.2545
ENOX1	4	0.25759	0.41053	0.985638	737	1	-0.35615
HSD17B10	4	0.25771	0.41062	0.985638	738	2	0.22926
MOGAT2	4	0.25794	0.41083	0.985638	739	2	0.23547
COX15	4	0.25804	0.41093	0.985638	740	2	0.22296
WWOX	4	0.25893	0.41176	0.985638	741	2	0.029728
ABO	4	0.25959	0.41242	0.985638	742	2	0.052326
LCTL	4	0.26027	0.41315	0.985638	743	2	0.25539
PLA2G12A	4	0.26054	0.41341	0.985638	744	2	0.31469
PFKL	4	0.26101	0.41385	0.985638	745	2	0.15131
BCHE	4	0.26148	0.4143	0.985638	746	2	0.31642
RDH10	4	0.26251	0.41527	0.985638	747	1	-0.34287
ITPKB	4	0.26314	0.41592	0.985638	748	2	-0.59635
CRYL1	4	0.26337	0.41611	0.985638	749	2	0.14545
HS3ST3A1	4	0.2634	0.41615	0.985638	750	2	0.20289
H6PD	4	0.26503	0.41763	0.985988	751	2	0.35647
SULT4A1	4	0.26518	0.41782	0.985988	752	1	-0.40635
ACADL	4	0.26562	0.41829	0.985988	753	1	-0.33744
CYP46A1	4	0.26606	0.41879	0.985988	754	2	0.12288
FUT6	4	0.26693	0.4196	0.985988	755	2	0.035731
MAOB	4	0.26695	0.41963	0.985988	756	1	-0.05716
CBS	4	0.26811	0.42059	0.986461	757	2	0.21143
FAR1	4	0.26883	0.42129	0.986461	758	2	0.28904
ADAD2	4	0.26906	0.4215	0.986461	759	2	0.047157
SHMT2	4	0.27005	0.42246	0.986727	760	2	-0.11895
GALNT16	4	0.2705	0.42293	0.986727	761	1	-0.020085
NEU1	4	0.27094	0.42338	0.986727	762	1	-0.13721
HMGCR	4	0.27138	0.42384	0.986727	763	2	0.10708
GSTA4	4	0.27226	0.42466	0.987355	764	1	-0.010551
CERKL	4	0.27491	0.42753	0.990873	765	1	-0.14983
GSTM4	4	0.27535	0.428	0.990873	766	1	-1.3954

COX16	4	0.27669	0.42945	0.990873	767	2	0.25424
SOD3	4	0.27693	0.42971	0.990873	768	2	0.20918
PLCH1	4	0.2771	0.42989	0.990873	769	2	0.10506
ADCY7	4	0.27754	0.43034	0.990873	770	1	-0.1857
DGAT1	4	0.27974	0.43242	0.990873	771	1	-0.34456
GAL3ST4	4	0.28004	0.43269	0.990873	772	2	-0.39907
ACSL6	4	0.28061	0.43321	0.990873	773	1	-0.28631
PLA2G7	4	0.28171	0.43432	0.990873	774	2	-0.25455
DHRS9	4	0.28236	0.43505	0.990873	775	1	-0.32431
HAAO	4	0.28267	0.43531	0.990873	776	2	-0.0071773
SAT2	4	0.28323	0.43587	0.990873	777	1	-0.18815
CYP2A6	4	0.28454	0.43714	0.990873	778	2	-0.14585
MGST2	4	0.28483	0.43739	0.990873	779	2	-0.42336
PISD	4	0.28541	0.43793	0.990873	780	2	-0.16177
NT5DC3	4	0.28585	0.43834	0.990873	781	2	0.048881
LYG1	4	0.28672	0.4392	0.990873	782	1	-0.42919
PIGN	4	0.287	0.4395	0.990873	783	2	0.19674
PDXP	4	0.28715	0.43968	0.990873	784	1	0.033124
ALDH1L1	4	0.28724	0.43977	0.990873	785	2	-0.37786
GLRX	4	0.28772	0.44026	0.990873	786	2	0.25441
FIG4	4	0.28889	0.44148	0.990873	787	1	-1.1257
PGAM2	4	0.28916	0.44172	0.990873	788	2	0.24946
KHK	4	0.28932	0.44189	0.990873	789	1	-0.32447
DECR2	4	0.2894	0.44195	0.990873	790	2	0.25322
LPCAT4	4	0.29013	0.44263	0.990873	791	2	0.22172
GDPD1	4	0.29105	0.44345	0.990873	792	1	-0.35919
PDE6A	4	0.29302	0.44551	0.990873	793	2	0.052526
ATP13A5	4	0.29321	0.44573	0.990873	794	2	0.14102
GPX6	4	0.29537	0.44775	0.990873	795	1	-0.37875
ST3GAL6	4	0.2958	0.44819	0.990873	796	1	-0.27333
PTGR2	4	0.2964	0.44871	0.990873	797	2	-0.071964
DHRS12	4	0.29752	0.44975	0.990873	798	1	-0.14605
NPL	4	0.29785	0.45007	0.990873	799	2	-0.19113
LPO	4	0.29838	0.45058	0.990873	800	2	-0.074147
PGM3	4	0.29955	0.45183	0.990873	801	2	-0.21937
ADCY5	4	0.30052	0.45272	0.990873	802	2	0.21789
BLVRA	4	0.30052	0.45273	0.990873	803	2	0.13376
SDR16C5	4	0.30124	0.45338	0.990873	804	2	0.30826
PHYH	4	0.30181	0.4539	0.990873	805	1	-0.28927
IMPA1	4	0.30266	0.45471	0.990873	806	1	-0.21291
PLA2G4E	4	0.3027	0.45475	0.990873	807	2	0.16341
UGT1A10	4	0.30294	0.45502	0.990873	808	2	0.35766
PI4K2B	4	0.30343	0.45553	0.990873	809	2	0.031791
DAGLA	4	0.30391	0.45597	0.990873	810	2	-0.020909
CYP2A13	4	0.30395	0.45601	0.990873	811	1	0.023846
MGLL	4	0.30437	0.45639	0.990873	812	2	0.18506

NAT8B	4	0.30586	0.45792	0.990873	813	2	0.07713
SGMS1	4	0.3061	0.4581	0.990873	814	2	-0.057018
ACER1	4	0.30732	0.45919	0.990873	815	2	0.092684
NUDT12	4	0.3078	0.45967	0.990873	816	2	0.0057423
GCNT1	4	0.30877	0.46061	0.990873	817	2	-0.015268
PRPS1L1	4	0.3099	0.46159	0.990873	818	1	0.017104
HMGCLL1	4	0.30999	0.46172	0.990873	819	2	0.097236
EPHX2	4	0.31075	0.4624	0.990873	820	1	-0.2855
DGAT2L6	4	0.31117	0.46289	0.990873	821	2	0.17177
PLPP2	4	0.31194	0.46359	0.990873	822	2	-0.13239
DHRS4	4	0.31202	0.46367	0.990873	823	1	-0.30366
PIGS	4	0.31243	0.46407	0.990873	824	2	0.20583
COX18	4	0.31365	0.46534	0.990873	825	2	0.335
NDST1	4	0.31455	0.46638	0.990873	826	1	-0.09069
DDOST	4	0.31498	0.4668	0.990873	827	2	0.21414
FLAD1	4	0.3156	0.46743	0.990873	828	2	0.14813
AMDHD2	4	0.31666	0.46861	0.990873	829	2	0.28786
CRYZL1	4	0.31731	0.46919	0.990873	830	2	0.35323
NDUFA4L2	4	0.3178	0.46959	0.990873	831	2	0.16943
ALG3	4	0.31834	0.47013	0.990873	832	1	-0.81446
PLA2G12B	4	0.31878	0.47058	0.990873	833	2	-0.3524
NUDT21	4	0.31951	0.47133	0.990873	834	2	-0.26664
HAGHL	4	0.3196	0.4714	0.990873	835	1	-0.15765
ACOT8	4	0.32098	0.47273	0.990873	836	2	0.12883
GYG2	4	0.32171	0.47347	0.990873	837	2	0.044123
LSS	4	0.32254	0.47424	0.990873	838	1	0.092408
ITPKC	4	0.32379	0.47546	0.990873	839	1	-0.35751
ACOXL	4	0.32421	0.47608	0.990873	840	1	-0.67242
NOX1	4	0.32462	0.47649	0.990873	841	1	-0.10981
ENO3	4	0.32504	0.47686	0.990873	842	1	-0.1761
PIGV	4	0.32539	0.47721	0.990873	843	2	0.13933
NDUFB1	4	0.32612	0.47795	0.990873	844	2	0.15526
GSTA3	4	0.32712	0.47898	0.990873	845	1	-0.57211
ALG1	4	0.33029	0.48191	0.990873	846	2	0.23786
CERS6	4	0.33054	0.48218	0.990873	847	2	0.23852
BCO1	4	0.33086	0.48247	0.990873	848	1	-4.2315
NADSYN1	4	0.33169	0.48321	0.990873	849	2	0.24351
ABHD11	4	0.33252	0.48392	0.990873	850	2	0.18025
DPYSL2	4	0.33293	0.48431	0.990873	851	1	-0.041067
CHST13	4	0.33373	0.48515	0.990873	852	2	-0.3601
ACY1	4	0.33376	0.48516	0.990873	853	2	-0.15594
GATM	4	0.33398	0.48541	0.990873	854	2	0.015097
SDS	4	0.33417	0.4856	0.990873	855	1	-0.71959
AOC1	4	0.335	0.48638	0.990873	856	2	0.12275
CYP17A1	4	0.3357	0.48707	0.990873	857	2	0.22579
FRRS1	4	0.33619	0.48751	0.990873	858	2	0.26892

PIP5K1B	4	0.33623	0.48757	0.990873	859	1	0.0075076
LARGE1	4	0.33644	0.48773	0.990873	860	2	0.15102
SULF2	4	0.33669	0.48799	0.990873	861	2	0.23059
GMPR2	4	0.33693	0.4882	0.990873	862	2	0.04225
ENO1	4	0.33829	0.48955	0.990873	863	1	-0.052823
CRYM	4	0.3387	0.48998	0.990873	864	1	-0.01489
NT5DC1	4	0.3389	0.49026	0.990873	865	2	0.077374
ADCY3	4	0.33952	0.49072	0.990873	866	1	-0.05631
B3GAT1	4	0.33993	0.49118	0.990873	867	1	-0.18587
PAH	4	0.34075	0.49212	0.990873	868	1	-0.98808
GFOD2	4	0.34087	0.4922	0.990873	869	2	0.1162
B4GALT4	4	0.3426	0.49395	0.990873	870	2	0.096813
DERA	4	0.34321	0.49464	0.990873	871	1	-0.093245
MMADHC	4	0.34402	0.49542	0.990873	872	1	-1.8836
DPYSL3	4	0.34457	0.49587	0.990873	873	2	0.23401
COX7A2	4	0.34484	0.49619	0.990873	874	1	-0.19112
MBOAT7	4	0.34525	0.49656	0.990873	875	1	-0.066663
PLCB1	4	0.34606	0.49742	0.990873	876	1	-0.80891
ALDH9A1	4	0.34728	0.49864	0.990873	877	2	0.16372
GNMT	4	0.3481	0.49948	0.990873	878	1	-0.11857
NDUFA2	4	0.34877	0.50009	0.990873	879	2	0.12822
NDUFC1	4	0.34951	0.50077	0.990873	880	2	0.29089
IDS	4	0.34972	0.50093	0.990873	881	2	-0.61869
PYGM	4	0.35093	0.50214	0.990873	882	1	0.064671
CYP2E1	4	0.35134	0.50261	0.990873	883	2	0.092467
GSTA2	4	0.35211	0.50327	0.990873	884	2	0.34272
HS6ST1	4	0.35336	0.50437	0.990873	885	1	-1.0167
GALNT1	4	0.35371	0.50468	0.990873	886	2	0.17049
ST6GALNAC4	4	0.35421	0.50528	0.990873	887	2	-0.054823
ACOT7	4	0.35445	0.50548	0.990873	888	2	0.16493
ALG13	4	0.35538	0.50644	0.990873	889	1	-0.13084
PAICS	4	0.35569	0.50677	0.990873	890	2	-0.38267
NMNAT3	4	0.35668	0.50787	0.990873	891	2	0.13565
MGST3	4	0.35693	0.50808	0.990873	892	2	0.17995
ETFB	4	0.35718	0.5083	0.990873	893	2	-1.0867
UQCRB	4	0.35841	0.50945	0.990873	894	2	0.36628
GNS	4	0.3586	0.50959	0.990873	895	1	-0.68296
AMY2A	4	0.3594	0.51055	0.990873	896	1	-0.1157
PRDX6	4	0.35941	0.51056	0.990873	897	2	0.21493
CHST6	4	0.3599	0.51101	0.990873	898	2	0.23016
AACS	4	0.3606	0.51179	0.990873	899	1	-0.058802
NAALADL1	4	0.361	0.51213	0.990873	900	2	0.15621
HAS2	4	0.36163	0.51275	0.990873	901	2	0.20867
PDE1A	4	0.363	0.51409	0.990873	902	1	-0.25831
DGKD	4	0.36312	0.51419	0.990873	903	2	0.20342
PLA2G5	4	0.3634	0.51446	0.990873	904	1	0.09616

NDUFB2	4	0.3638	0.51482	0.990873	905	2	0.21166
PIK3CA	4	0.36499	0.51606	0.990873	906	2	0.13658
ETNPPL	4	0.3651	0.51622	0.990873	907	2	0.15859
INPP5B	4	0.36634	0.51751	0.990873	908	2	0.12377
AK3	4	0.36738	0.51853	0.990873	909	1	-0.76106
EXT2	4	0.36778	0.5189	0.990873	910	1	-0.056756
GPI	4	0.36818	0.51931	0.990873	911	1	-0.40577
COQ7	4	0.36833	0.51946	0.990873	912	2	0.2014
GBA	4	0.36858	0.51965	0.990873	913	2	-0.056938
NMNAT1	4	0.36937	0.52032	0.990873	914	1	-0.12526
COQ8A	4	0.37016	0.52113	0.990873	915	1	-0.24346
PAPSS1	4	0.37106	0.522	0.990873	916	2	0.049446
ABHD5	4	0.37156	0.52246	0.990873	917	2	-0.41191
COX7C	4	0.37205	0.52297	0.990873	918	2	0.06802
OLAH	4	0.3728	0.52363	0.990873	919	2	-0.2559
TXNRD2	4	0.37304	0.52386	0.990873	920	2	0.17492
PFKFB1	4	0.37332	0.52407	0.990873	921	1	-0.16358
AKR1C1	4	0.37404	0.52473	0.990873	922	2	-0.12378
ME2	4	0.37411	0.52478	0.990873	923	1	-4.0745
IMPDH1	4	0.37429	0.52493	0.990873	924	2	-0.25004
ACSM4	4	0.3745	0.5251	0.990873	925	1	-0.53289
POMT1	4	0.37529	0.52591	0.990873	926	1	-0.000775
PDE4C	4	0.37553	0.52619	0.990873	927	2	0.11401
PDE11A	4	0.37568	0.52633	0.990873	928	1	-0.55445
CBR3	4	0.37627	0.5269	0.990873	929	2	0.10213
ACHE	4	0.37686	0.52751	0.990873	930	1	-1.1434
PRODH	4	0.37702	0.52772	0.990873	931	2	-0.47586
DHDH	4	0.37843	0.5291	0.990873	932	1	-0.20622
SMS	4	0.38	0.53053	0.990873	933	1	-1.1228
GLDC	4	0.38078	0.53133	0.990873	934	1	-0.16317
SRD5A2	4	0.38124	0.53175	0.990873	935	2	0.20929
ACOT1	4	0.38149	0.53197	0.990873	936	2	0.10484
FDX1	4	0.38156	0.53205	0.990873	937	1	-0.64405
GNE	4	0.38199	0.5325	0.990873	938	2	-0.043137
PDHA2	4	0.38234	0.53284	0.990873	939	1	0.13567
AMY1A	4	0.38312	0.53352	0.990873	940	1	-0.06332
LIPK	4	0.38373	0.5341	0.990873	941	2	-0.12687
ELOVL7	4	0.38429	0.53463	0.990873	942	1	0.031332
ETNK2	4	0.38572	0.536	0.990873	943	2	-0.30142
CHST9	4	0.38585	0.53612	0.990873	944	1	-0.20587
SUCLG2	4	0.38621	0.53646	0.990873	945	2	0.090117
ETFA	4	0.38624	0.53647	0.990873	946	1	-0.12745
DLAT	4	0.38771	0.53788	0.990873	947	2	0.11876
GPX2	4	0.38796	0.53819	0.990873	948	2	-0.13913
GSTZ1	4	0.38818	0.53835	0.990873	949	1	-0.20933
PHYKPL	4	0.38856	0.53882	0.990873	950	1	-0.59091

UQCRC1	4	0.38895	0.53914	0.990873	951	1	-0.081182
GSTK1	4	0.3892	0.53939	0.990873	952	2	-0.1349
HPDL	4	0.38934	0.53958	0.990873	953	1	-0.11745
ACOX1	4	0.38945	0.53967	0.990873	954	2	-0.048931
BCKDHA	4	0.3905	0.54073	0.990873	955	1	-0.28605
LDHC	4	0.39069	0.54098	0.990873	956	2	0.11015
NDUFS2	4	0.39088	0.54119	0.990873	957	1	-0.068867
GK	4	0.39144	0.54168	0.990873	958	2	0.20393
CYP26A1	4	0.39243	0.54275	0.990873	959	2	-0.090048
GPX5	4	0.3932	0.54357	0.990873	960	2	-0.10217
PLCZ1	4	0.39442	0.54474	0.990873	961	2	0.23063
UQCRC2	4	0.39592	0.54609	0.990873	962	2	-0.15915
ALAD	4	0.39627	0.54643	0.990873	963	1	-0.022525
ACSBG1	4	0.39641	0.54658	0.990873	964	2	-0.36732
GALNT2	4	0.39791	0.54813	0.990873	965	2	0.049529
CHPT1	4	0.39816	0.54837	0.990873	966	2	-0.2073
HS6ST3	4	0.39915	0.54934	0.990873	967	2	0.27772
ACAD10	4	0.39933	0.54951	0.990873	968	1	0.028136
MAN1A2	4	0.40015	0.55037	0.990873	969	2	0.011223
ALDOA	4	0.40048	0.55073	0.990873	970	1	-0.3981
NDUFS6	4	0.40086	0.55097	0.990873	971	1	-0.14577
CSGALNACT2	4	0.40189	0.55202	0.990873	972	2	0.23403
PLCD3	4	0.40289	0.55293	0.990873	973	2	0.11845
CYP27A1	4	0.40413	0.55407	0.990873	974	2	0.16751
ST3GAL1	4	0.40429	0.55418	0.990873	975	2	-0.1037
MFNG	4	0.40513	0.55504	0.990873	976	2	0.089151
ATP13A3	4	0.4058	0.55575	0.990873	977	2	-3.2777
NDUFB10	4	0.40618	0.5561	0.990873	978	1	-0.15047
ISCA2	4	0.40656	0.55645	0.990873	979	2	-0.17785
GALNT7	4	0.40694	0.55682	0.990873	980	1	-0.051673
OGFOD2	4	0.40732	0.55714	0.990873	981	1	-0.14397
SHMT1	4	0.4077	0.55757	0.990873	982	1	-0.078875
MDH2	4	0.40808	0.55787	0.990873	983	1	-0.63401
SAT1	4	0.40811	0.55792	0.990873	984	2	-0.05753
THEM4	4	0.40911	0.55893	0.990873	985	2	0.1794
CYP27C1	4	0.40921	0.55902	0.990873	986	2	0.20683
UAP1L1	4	0.4101	0.55996	0.990873	987	2	0.0070816
ENPP2	4	0.4106	0.56037	0.990873	988	2	0.081004
MSRB3	4	0.41072	0.56045	0.990873	989	1	0.14881
GSTO1	4	0.41185	0.56146	0.990873	990	1	-0.035987
NAT1	4	0.41185	0.56146	0.990873	991	2	0.12356
OXSM	4	0.41222	0.56181	0.990873	992	1	-0.45149
CYP4F3	4	0.41297	0.56255	0.990873	993	1	0.0027723
GLO1	4	0.41309	0.56272	0.990873	994	2	-0.22084
MTHFSD	4	0.41335	0.56309	0.990873	995	1	-0.13344
CHI3L1	4	0.41372	0.56352	0.990873	996	2	0.099612

DCT	4	0.41434	0.56418	0.990873	997	2	0.0027519
SULT1C3	4	0.41447	0.56434	0.990873	998	2	0.21806
ELOVL4	4	0.41522	0.56507	0.990873	999	1	-0.29875
ACADS	4	0.41583	0.56564	0.990873	1000	2	0.12868
QDPR	4	0.41784	0.56745	0.990873	1001	1	-0.56838
CYP7B1	4	0.41821	0.56785	0.990873	1002	1	-5.1067
CYB561	4	0.41857	0.56821	0.990873	1003	2	0.12923
HAL	4	0.41882	0.56839	0.990873	1004	2	0.17272
UPB1	4	0.41931	0.56889	0.990873	1005	2	0.076989
TREH	4	0.42031	0.57001	0.990873	1006	2	-0.042635
AFMID	4	0.42056	0.57021	0.990873	1007	2	0.054967
LDHB	4	0.42354	0.57312	0.990873	1008	2	0.012141
GCDH	4	0.42453	0.57413	0.990873	1009	1	0.0337
IPMK	4	0.4249	0.57448	0.990873	1010	1	-0.73723
ISYNA1	4	0.42528	0.57489	0.990873	1011	2	-0.0099307
NDUFB6	4	0.42603	0.57564	0.990873	1012	2	0.19071
ARSA	4	0.42675	0.57628	0.990873	1013	1	-0.26268
PDE4D	4	0.42712	0.57662	0.990873	1014	1	-0.23792
CSGALNACT1	4	0.42785	0.57736	0.990873	1015	2	0.22563
ST8SIA3	4	0.42802	0.57748	0.990873	1016	2	0.19205
CSAD	4	0.42822	0.57768	0.990873	1017	1	-0.12964
NDUFB3	4	0.42852	0.57797	0.990873	1018	2	0.29771
CYP2U1	4	0.42877	0.57826	0.990873	1019	2	-0.047044
CYP2B6	4	0.42969	0.57908	0.990873	1020	1	-0.48553
ADH6	4	0.43175	0.58093	0.990873	1021	2	0.18108
CA9	4	0.43225	0.58143	0.990873	1022	2	-0.03391
GSS	4	0.43226	0.58145	0.990873	1023	1	-1.0082
ATP13A2	4	0.4325	0.58169	0.990873	1024	2	0.20757
SPR	4	0.43263	0.58183	0.990873	1025	1	-0.67648
ENTPD7	4	0.43299	0.58217	0.990873	1026	2	-0.15357
ADO	4	0.43336	0.58258	0.990873	1027	1	0.06294
OASL	4	0.43482	0.58383	0.990873	1028	1	-0.19543
CYP26B1	4	0.43519	0.58412	0.990873	1029	1	0.18592
ALDH3B1	4	0.43523	0.58416	0.990873	1030	2	-0.10284
AGL	4	0.43628	0.58513	0.990873	1031	1	-0.36202
ST8SIA5	4	0.43647	0.58534	0.990873	1032	2	-0.29263
PIK3R1	4	0.43672	0.5856	0.990873	1033	2	0.15359
CRAT	4	0.43722	0.58604	0.990873	1034	2	-0.6202
NDUFS7	4	0.43747	0.58627	0.990873	1035	2	-0.082847
PPCS	4	0.4381	0.58692	0.990873	1036	1	-0.019193
ACO2	4	0.43821	0.58702	0.990873	1037	2	0.15354
B4GALT3	4	0.43846	0.58728	0.990873	1038	2	0.079307
NDUFA10	4	0.43896	0.58779	0.990873	1039	2	0.14506
UGT1A7	4	0.43919	0.58797	0.990873	1040	1	0.020891
ALG10B	4	0.4392	0.58798	0.990873	1041	2	-0.061449
MICAL3	4	0.43955	0.58832	0.990873	1042	1	-0.42461

TBXAS1	4	0.44045	0.58914	0.990873	1043	2	-0.079118
PIK3R4	4	0.4407	0.58936	0.990873	1044	2	0.2146
NDUFA13	4	0.44144	0.59001	0.990873	1045	2	0.1403
ISCA1	4	0.44318	0.59158	0.990873	1046	2	0.021395
NDUFB5	4	0.44343	0.59182	0.990873	1047	2	0.11337
DPM2	4	0.44353	0.5919	0.990873	1048	1	0.10077
PNPLA4	4	0.44417	0.59245	0.990873	1049	2	0.17781
GALNT4	4	0.44426	0.59251	0.990873	1050	1	-0.0011753
FASN	4	0.44467	0.59288	0.990873	1051	2	-0.067725
NUDT8	4	0.44498	0.59314	0.990873	1052	2	0.12352
DHRS1	4	0.44516	0.59331	0.990873	1053	2	0.085249
GNPDA1	4	0.44606	0.59418	0.990873	1054	1	-0.33653
G6PC3	4	0.44713	0.59528	0.990873	1055	1	0.089378
PIK3C3	4	0.44893	0.59709	0.990873	1056	1	0.10738
LPIN1	4	0.44913	0.59736	0.990873	1057	2	-0.69258
HMOX2	4	0.44938	0.59761	0.990873	1058	2	0.13509
AGPAT5	4	0.44964	0.59786	0.990873	1059	1	0.22619
CES2	4	0.44988	0.5981	0.990873	1060	2	-0.31439
PIGL	4	0.45013	0.59839	0.990873	1061	2	0.0086528
NPR2	4	0.45036	0.59861	0.990873	1062	1	0.13761
CYP4F2	4	0.45037	0.59861	0.990873	1063	2	0.15663
COQ5	4	0.45136	0.59968	0.990873	1064	2	0.15087
NDUFB7	4	0.45214	0.60038	0.990873	1065	1	-0.021281
CYP1A1	4	0.45286	0.60111	0.990873	1066	1	-1.6599
MPO	4	0.45335	0.60164	0.990873	1067	2	-0.2561
ALDH8A1	4	0.45357	0.6019	0.990873	1068	2	0.19118
ACER3	4	0.4536	0.60192	0.990873	1069	2	-0.0002203<br>5
DPM3	4	0.45434	0.60263	0.990873	1070	2	0.052743
SDHA	4	0.45483	0.60309	0.990873	1071	2	-0.28959
NUDT16L1	4	0.4557	0.60389	0.990873	1072	1	-0.023085
HSD17B11	4	0.45641	0.60459	0.990873	1073	1	-0.41156
NOX5	4	0.45756	0.60562	0.990873	1074	2	0.10598
DUOX2	4	0.45805	0.60602	0.990873	1075	2	0.15171
HKDC1	4	0.4588	0.60678	0.990873	1076	2	-0.026082
PIGQ	4	0.45889	0.60684	0.990873	1077	1	0.0082169
TPO	4	0.46028	0.60814	0.990873	1078	2	0.14723
CA5B	4	0.46065	0.60853	0.990873	1079	1	-0.078942
PLA2G4D	4	0.46078	0.60868	0.990873	1080	2	-0.48573
DPEP2	4	0.46206	0.60988	0.990873	1081	1	0.03197
ACBD6	4	0.4635	0.61125	0.990873	1082	2	-0.068992
FPGT	4	0.46375	0.61159	0.990873	1083	2	0.10118
PIP4K2A	4	0.46548	0.61328	0.990873	1084	2	0.073242
B3GALT4	4	0.46622	0.61403	0.990873	1085	2	-0.05503
PCCA	4	0.46627	0.61407	0.990873	1086	1	-0.32303
HAS3	4	0.46662	0.61439	0.990873	1087	1	-0.0043247

SATL1	4	0.46696	0.61479	0.990873	1088	2	0.18302
ALG11	4	0.46697	0.61481	0.990873	1089	1	-0.057688
PTGR1	4	0.46745	0.61535	0.990873	1090	2	-0.95715
SOAT1	4	0.46837	0.61641	0.990873	1091	1	-0.14882
CDS1	4	0.46968	0.61763	0.990873	1092	2	0.25135
HGD	4	0.47091	0.61892	0.990873	1093	2	0.24939
NUDT6	4	0.47116	0.6192	0.990873	1094	2	0.10117
AMACR	4	0.47239	0.62034	0.990873	1095	2	0.028589
ACO1	4	0.47254	0.62047	0.990873	1096	1	-0.090054
NDUFS1	4	0.47288	0.62071	0.990873	1097	1	-0.07347
FAHD1	4	0.47412	0.62188	0.990873	1098	2	0.033929
ACY3	4	0.47535	0.62314	0.990873	1099	2	0.20847
MAT2A	4	0.47584	0.62366	0.990873	1100	2	-0.02494
HSD3B7	4	0.47634	0.62406	0.990873	1101	2	0.092447
ADSS	4	0.47634	0.62407	0.990873	1102	1	-0.15561
MAN2A1	4	0.47737	0.62499	0.990873	1103	1	-0.13823
ENOSF1	4	0.47757	0.62517	0.990873	1104	2	-0.49967
HSD11B1	4	0.47772	0.62533	0.990873	1105	1	-0.040384
MIOX	4	0.47806	0.62564	0.990873	1106	1	-0.24759
COX6B2	4	0.47855	0.62609	0.990873	1107	2	0.15578
PTGES	4	0.4788	0.6263	0.990873	1108	2	-0.021095
GNPDA2	4	0.48115	0.62864	0.990873	1109	1	0.022506
MTRR	4	0.48218	0.62967	0.990873	1110	1	-0.67009
GPX7	4	0.48225	0.62972	0.990873	1111	2	0.16228
ADCY6	4	0.48252	0.63	0.990873	1112	1	0.017961
TPI1	4	0.48323	0.63066	0.990873	1113	2	0.05395
NME7	4	0.48372	0.63112	0.990873	1114	2	0.15516
UGT2B28	4	0.48495	0.63242	0.990873	1115	2	0.36435
PDE2A	4	0.48661	0.63399	0.990873	1116	1	-0.58541
ASNS	4	0.48692	0.63422	0.990873	1117	2	0.14122
PFKP	4	0.48716	0.63445	0.990873	1118	2	0.16272
B4GALNT2	4	0.48741	0.63472	0.990873	1119	2	0.021755
NUDT11	4	0.48796	0.63521	0.990873	1120	1	-0.33438
LCT	4	0.48913	0.63636	0.990873	1121	2	0.1613
ST8SIA2	4	0.49011	0.63727	0.990873	1122	2	0.13935
ECHS1	4	0.49033	0.63743	0.990873	1123	1	-0.19762
COX6C	4	0.49067	0.63781	0.990873	1124	1	-0.47073
NAALADL2	4	0.49085	0.638	0.990873	1125	2	0.076837
ADPGK	4	0.49109	0.63825	0.990873	1126	2	0.079991
PANK3	4	0.49134	0.6385	0.990873	1127	2	-0.073543
TMLHE	4	0.49135	0.63852	0.990873	1128	1	-0.34976
PMM2	4	0.49232	0.63943	0.990873	1129	2	0.10678
CHPF2	4	0.49452	0.64169	0.990873	1130	2	0.099707
B3GNT3	4	0.49471	0.64189	0.990873	1131	1	-0.50217
LTC4S	4	0.4955	0.64264	0.990873	1132	2	0.087422
DEGS2	4	0.49839	0.64394	0.990873	1133	1	-0.2607

ENTPD1	4	0.49939	0.64432	0.990873	1134	1	-0.38286
DHRS7B	4	0.50172	0.64527	0.990873	1135	1	0.13395
NDUFC2	4	0.50371	0.64603	0.990873	1136	1	0.017625
FADS2	4	0.50438	0.64626	0.990873	1137	1	-0.16212
PRDX5	4	0.50537	0.64659	0.990873	1138	1	-0.17503
OAS2	4	0.5057	0.64673	0.990873	1139	1	-0.62823
NDUFA11	4	0.50833	0.64778	0.990873	1140	1	-0.16541
LDHD	4	0.51063	0.64872	0.990873	1141	1	-0.80506
COQ3	4	0.51129	0.64905	0.990873	1142	1	-0.17273
G6PD	4	0.51227	0.64948	0.990873	1143	1	-0.17316
MTHFD1L	4	0.51292	0.64976	0.990873	1144	1	-0.14044
PIP5K1A	4	0.5139	0.65018	0.990873	1145	1	-0.083231
NME4	4	0.5152	0.65067	0.990873	1146	1	-0.088155
PIP4K2B	4	0.51618	0.65117	0.990873	1147	1	-0.059354
GDE1	4	0.5165	0.6513	0.990873	1148	1	-0.10326
MDH1	4	0.51683	0.6514	0.990873	1149	1	-0.059019
FAHD2A	4	0.51748	0.65166	0.990873	1150	1	-0.2829
GLB1	4	0.5178	0.6518	0.990873	1151	1	0.1349
SMOX	4	0.5191	0.65234	0.990873	1152	1	-0.55916
ABHD8	4	0.51942	0.65243	0.990873	1153	1	-0.34419
GLT8D2	4	0.52007	0.65269	0.990873	1154	1	-0.15094
CYP39A1	4	0.52103	0.65311	0.990873	1155	1	-0.85805
AKR1B10	4	0.522	0.65352	0.990873	1156	1	-0.037518
NDUFA4	4	0.52264	0.65376	0.990873	1157	1	-0.21313
SUCLG1	4	0.52489	0.65458	0.990873	1158	1	-0.13467
NAALAD2	4	0.52585	0.65494	0.990873	1159	1	-0.34062
CKB	4	0.52617	0.65509	0.990873	1160	1	-0.11332
GALNT6	4	0.52873	0.65607	0.990873	1161	1	-0.096501
UCK2	4	0.52904	0.65621	0.990873	1162	1	0.033616
COX8C	4	0.53159	0.65733	0.990873	1163	1	-0.21285
HYI	4	0.53254	0.65768	0.990873	1164	1	-0.1636
B3GNTL1	4	0.5357	0.65909	0.990873	1165	1	-0.17595
AGMAT	4	0.53632	0.65929	0.990873	1166	1	-0.2801
NDUFS4	4	0.53695	0.65956	0.990873	1167	1	-0.42655
ADH1A	4	0.53727	0.65971	0.990873	1168	1	-0.7349
PIGF	4	0.53758	0.65983	0.990873	1169	1	-1.1714
CERS5	4	0.53978	0.66077	0.990873	1170	1	0.11571
HAO1	4	0.54103	0.66134	0.990873	1171	1	0.17083
ST3GAL2	4	0.54507	0.66314	0.990873	1172	1	-0.054886
HIBADH	4	0.54693	0.66395	0.990873	1173	1	-0.62385
DDO	4	0.54786	0.66433	0.990873	1174	1	-0.44826
PIK3R6	4	0.55125	0.66567	0.990873	1175	1	-0.40934
AKR7A3	4	0.55644	0.66801	0.990873	1176	1	-0.40324
DPYSL5	4	0.55675	0.66812	0.990873	1177	1	-0.039673
UGT3A2	4	0.55736	0.6684	0.990873	1178	1	-0.14658
PDE8B	4	0.55948	0.66939	0.990873	1179	1	-0.14941

GBE1	4	0.5625	0.67063	0.990873	1180	1	-0.35736
ACSM3	4	0.56461	0.67163	0.990873	1181	1	0.066076
FN3KRP	4	0.56551	0.67209	0.990873	1182	1	0.21542
CYB5R3	4	0.5667	0.67254	0.990873	1183	1	-0.257
CYP8B1	4	0.5673	0.67283	0.990873	1184	1	-0.44202
ST6GAL1	4	0.5676	0.67291	0.990873	1185	1	-0.62005
ALOX12	4	0.5682	0.67317	0.990873	1186	1	-0.66224
AZIN2	4	0.5685	0.67329	0.990873	1187	1	-0.089657
ACBD7	4	0.56879	0.67343	0.990873	1188	1	-0.20488
HSD3B2	4	0.57028	0.67403	0.990873	1189	1	-0.22319
AGXT2	4	0.57058	0.67416	0.990873	1190	1	0.13284
SPTLC2	4	0.57147	0.67459	0.990873	1191	1	-0.39056
COX17	4	0.57236	0.67496	0.990873	1192	1	-0.58429
DDT	4	0.57295	0.67525	0.990873	1193	1	-0.96044
PEMT	4	0.57413	0.67583	0.990873	1194	1	-0.30164
FMO3	4	0.5759	0.67664	0.990873	1195	1	-0.065842
PAPSS2	4	0.57678	0.67695	0.990873	1196	1	-0.18659
FADS1	4	0.57708	0.6771	0.990873	1197	1	-0.39227
MGAT4A	4	0.57796	0.67747	0.990873	1198	1	-0.78941
CA7	4	0.57913	0.67799	0.990873	1199	1	0.11675
ST3GAL5	4	0.57942	0.67806	0.990873	1200	1	-0.67247
CHST4	4	0.58001	0.6783	0.990873	1201	1	-0.32628
GAD2	4	0.58205	0.67919	0.990873	1202	1	0.10217
ALG14	4	0.58263	0.67948	0.990873	1203	1	-0.19552
AGXT	4	0.5835	0.67982	0.990873	1204	1	-0.096258
GLB1L2	4	0.58379	0.67994	0.990873	1205	1	-0.10563
GBA2	4	0.58669	0.6812	0.990873	1206	1	-0.29159
CYP4X1	4	0.58813	0.68183	0.990873	1207	1	-0.43604
CYP2S1	4	0.59186	0.6835	0.990873	1208	1	-0.15937
B3GALT6	4	0.59215	0.68365	0.990873	1209	1	-0.20628
ACSL3	4	0.593	0.6841	0.990873	1210	1	-0.56512
INPPL1	4	0.59443	0.68463	0.990873	1211	1	0.073546
MTHFD2L	4	0.59557	0.6851	0.990873	1212	1	-3.4061
SMPDL3A	4	0.59727	0.68583	0.990873	1213	1	-0.6679
ALDH5A1	4	0.5984	0.68636	0.990873	1214	1	-0.54672
UMPS	4	0.59868	0.68653	0.990873	1215	1	-0.45882
ALDH1A3	4	0.59925	0.68683	0.990873	1216	1	-0.29011
GUCY1B3	4	0.60178	0.68807	0.990873	1217	1	-0.53717
ABHD10	4	0.60234	0.68831	0.990873	1218	1	-0.15689
PLCB4	4	0.60346	0.68888	0.990873	1219	1	-0.44129
FOXRED2	4	0.60374	0.68902	0.990873	1220	1	-0.058097
PLA2G2E	4	0.60402	0.68918	0.990873	1221	1	-0.045349
MOCOS	4	0.6043	0.6893	0.990873	1222	1	-0.12473
SGMS2	4	0.60597	0.69009	0.990873	1223	1	-0.0985
CDS2	4	0.60653	0.69033	0.990873	1224	1	-0.18846
NDUFA12	4	0.60709	0.69062	0.990873	1225	1	-0.0092265

PTEN	4	0.60764	0.69082	0.990873	1226	1	-0.58292
MBOAT1	4	0.60986	0.69176	0.990873	1227	1	-0.2223
CHST12	4	0.61096	0.69224	0.990873	1228	1	-0.20061
MCCC2	4	0.61207	0.69275	0.990873	1229	1	-0.10239
MICAL2	4	0.61234	0.69289	0.990873	1230	1	-0.095662
PTDSS1	4	0.61344	0.69341	0.990873	1231	1	0.058673
PGM2	4	0.61399	0.69364	0.990873	1232	1	-2.0711
NDUFAB1	4	0.62377	0.69844	0.990873	1233	1	-0.25616
UPP2	4	0.62511	0.69916	0.990873	1234	1	-1.0434
CERS1	4	0.62725	0.70032	0.990873	1235	1	-0.13061
GNPNAT1	4	0.62779	0.70057	0.990873	1236	1	-0.5886
MDH1B	4	0.62992	0.70157	0.990873	1237	1	-0.19557
CYP4F8	4	0.63072	0.70197	0.990873	1238	1	-0.59033
CPT1C	4	0.63125	0.7023	0.990873	1239	1	-0.28904
GALT	4	0.6323	0.70282	0.990873	1240	1	-0.14428
GLT1D1	4	0.63257	0.70296	0.990873	1241	1	-0.1626
MAN1A1	4	0.63442	0.7039	0.990873	1242	1	-0.55194
GART	4	0.6352	0.70439	0.990873	1243	1	-0.019467
CEL	4	0.63652	0.70503	0.990873	1244	1	-0.15229
GGPS1	4	0.63939	0.70649	0.990873	1245	1	0.091855
GLYCTK	4	0.64017	0.70687	0.990873	1246	1	-0.40772
ARSD	4	0.64147	0.70754	0.990873	1247	1	-0.1399
CS	4	0.64329	0.70849	0.990873	1248	1	-0.49916
MSRB1	4	0.6438	0.70879	0.990873	1249	1	-0.31975
HSD17B3	4	0.64586	0.70994	0.990873	1250	1	-0.10107
NAT2	4	0.64689	0.7104	0.990873	1251	1	-0.039107
GUCY1A2	4	0.64715	0.71057	0.990873	1252	1	-0.052229
CRLS1	4	0.64945	0.71179	0.990873	1253	1	-0.24311
NANP	4	0.65072	0.71242	0.990873	1254	1	-0.48664
NAGA	4	0.65123	0.7127	0.990873	1255	1	-0.4799
MMEL1	4	0.65225	0.71318	0.990873	1256	1	-0.052079
CA11	4	0.65276	0.71344	0.990873	1257	1	-0.31673
HMOX1	4	0.65377	0.71386	0.990873	1258	1	-0.16262
MOCS3	4	0.65528	0.71465	0.990873	1259	1	-0.52088
NAT8	4	0.65654	0.71539	0.990873	1260	1	-0.26752
CYP3A5	4	0.65704	0.71566	0.990873	1261	1	-0.18201
PLA2G2A	4	0.65779	0.71606	0.990873	1262	1	-0.37779
CYP2R1	4	0.65855	0.71642	0.990873	1263	1	-0.20408
AKR1D1	4	0.6588	0.71658	0.990873	1264	1	0.10001
PTDSS2	4	0.66029	0.71746	0.990873	1265	1	-0.1174
HEXA	4	0.66129	0.71799	0.990873	1266	1	-0.10967
ALDH3B2	4	0.66204	0.71835	0.990873	1267	1	-0.16402
ME1	4	0.66327	0.71892	0.990873	1268	1	0.10768
IDO1	4	0.66352	0.71907	0.990873	1269	1	-0.21928
IP6K3	4	0.66377	0.71915	0.990873	1270	1	-0.35645
NT5E	4	0.66402	0.71929	0.990873	1271	1	-0.24385

MANEAL	4	0.66426	0.71946	0.990873	1272	1	-0.40181
ACSM2A	4	0.66451	0.71962	0.990873	1273	1	-0.015777
CYP4V2	4	0.66574	0.72032	0.990873	1274	1	0.003806
KMO	4	0.66697	0.72099	0.990873	1275	1	-0.42429
ALG12	4	0.66795	0.7215	0.990873	1276	1	-0.48391
DGKB	4	0.66869	0.72188	0.990873	1277	1	-0.56348
PNLIPRP3	4	0.66967	0.72229	0.990873	1278	1	-0.3933
LIPF	4	0.67015	0.72255	0.990873	1279	1	-0.02141
PIK3CD	4	0.67137	0.72314	0.990873	1280	1	0.11192
ALDH1L2	4	0.67234	0.72377	0.990873	1281	1	-0.24123
SDHAF3	4	0.67355	0.72437	0.990873	1282	1	-0.25132
ACBD4	4	0.675	0.7253	0.990873	1283	1	-0.85821
GYG1	4	0.67645	0.72614	0.990873	1284	1	0.10422
ASS1	4	0.67765	0.7266	0.990873	1285	1	0.15984
DHCR24	4	0.67812	0.72682	0.990873	1286	1	-0.59409
AASDH	4	0.6786	0.72713	0.990873	1287	1	-0.59516
PIP4K2C	4	0.67884	0.72726	0.990873	1288	1	-0.12371
AASDHPPT	4	0.67908	0.72741	0.990873	1289	1	-0.56638
PAOX	4	0.68194	0.72891	0.990873	1290	1	-0.13069
CA12	4	0.68217	0.72907	0.990873	1291	1	-0.37263
CRYZ	4	0.68383	0.73001	0.990873	1292	1	-0.056843
PCYT1B	4	0.68454	0.7304	0.990873	1293	1	-0.036935
PYCR2	4	0.68595	0.73116	0.990873	1294	1	-0.14953
ACSM1	4	0.68618	0.73128	0.990873	1295	1	0.084054
PAM	4	0.68712	0.73184	0.990873	1296	1	-0.30207
SEPHS1	4	0.68923	0.7329	0.990873	1297	1	-0.36719
RDH5	4	0.69016	0.73343	0.990873	1298	1	-0.51691
NT5C3A	4	0.69132	0.73413	0.990873	1299	1	0.08147
UEVLD	4	0.69155	0.73423	0.990873	1300	1	-0.386
COMTD1	4	0.69201	0.73456	0.990873	1301	1	0.0042884
DIO3	4	0.6934	0.73534	0.990873	1302	1	-0.26384
CAD	4	0.69409	0.73575	0.990873	1303	1	-0.14744
NAT10	4	0.69432	0.73586	0.990873	1304	1	-3.9626
ADI1	4	0.69455	0.73602	0.990873	1305	1	-0.019368
DPAGT1	4	0.69524	0.73647	0.990873	1306	1	-0.062361
GLS2	4	0.69616	0.73702	0.990873	1307	1	-0.090292
HADHB	4	0.69662	0.73738	0.990873	1308	1	0.14704
SUOX	4	0.69708	0.7377	0.990873	1309	1	-0.84478
HSD17B7	4	0.69731	0.73786	0.990873	1310	1	-0.030018
NUDT1	4	0.69753	0.73799	0.990873	1311	1	-0.16015
GSTP1	4	0.69799	0.73822	0.990873	1312	1	-0.063337
GGT6	4	0.69867	0.73869	0.990873	1313	1	0.028764
CHIT1	4	0.70072	0.73995	0.990873	1314	1	-0.016237
TK2	4	0.70095	0.74005	0.990873	1315	1	0.13481
NOX3	4	0.70117	0.74015	0.990873	1316	1	0.11164
NUDT15	4	0.70208	0.7407	0.990873	1317	1	-0.44132

THNSL1	4	0.7032	0.74143	0.990873	1318	1	-0.081786
DGKQ	4	0.70343	0.74154	0.990873	1319	1	-0.10297
CA14	4	0.70365	0.74167	0.990873	1320	1	-0.10902
SYNJ2	4	0.7041	0.74193	0.990873	1321	1	-0.16003
MVK	4	0.70455	0.7422	0.990873	1322	1	-0.22578
PNLIPRP2	4	0.70478	0.74233	0.990873	1323	1	-0.26122
RPIA	4	0.70545	0.74281	0.990873	1324	1	-0.053493
DDC	4	0.70634	0.74339	0.990873	1325	1	-0.020124
FDFT1	4	0.70701	0.74379	0.990873	1326	1	-0.49261
GLYATL1	4	0.70768	0.74413	0.990873	1327	1	0.0080708
B4GALT6	4	0.70879	0.74494	0.990873	1328	1	0.030537
PTGES2	4	0.70924	0.74519	0.990873	1329	1	-0.29358
ACSS2	4	0.71035	0.74585	0.990873	1330	1	-0.014425
GALK1	4	0.71145	0.74654	0.990873	1331	1	-0.26285
GLT8D1	4	0.71189	0.74681	0.990873	1332	1	-0.37854
NAT8L	4	0.71321	0.74752	0.990873	1333	1	-0.036716
DUT	4	0.71387	0.74791	0.990873	1334	1	-0.21019
TYMS	4	0.71518	0.74872	0.990873	1335	1	-0.79808
MINPP1	4	0.7154	0.74883	0.990873	1336	1	-0.10668
GSTA5	4	0.71562	0.74891	0.990873	1337	1	-0.48623
MGAT1	4	0.71692	0.74977	0.990873	1338	1	-0.17998
INPP5A	4	0.71725	0.74999	0.990873	1339	1	-0.39385
UXS1	4	0.71725	0.74999	0.990873	1340	1	-0.35048
PDHB	4	0.71757	0.75019	0.990873	1341	1	-0.40243
PLD4	4	0.71822	0.75054	0.990873	1342	1	-0.29864
ABHD13	4	0.71974	0.75152	0.990873	1343	1	0.091418
CYB5R2	4	0.71995	0.75165	0.990873	1344	1	-0.1331
PYCR3	4	0.72167	0.75274	0.990873	1345	1	-0.53171
IDH2	4	0.72253	0.7533	0.990873	1346	1	-0.43071
NUDT5	4	0.72296	0.75357	0.990873	1347	1	-0.045972
MGAT5	4	0.72381	0.75405	0.990873	1348	1	-0.23094
CYP4A22	4	0.72424	0.75428	0.990873	1349	1	-0.57363
CA5A	4	0.7253	0.75503	0.990873	1350	1	-0.37912
ACAA1	4	0.72637	0.75564	0.990873	1351	1	-0.34801
DGKA	4	0.72721	0.75611	0.990873	1352	1	-0.074337
GLB1L	4	0.72869	0.75692	0.990873	1353	1	0.13359
ABHD6	4	0.7289	0.75706	0.990873	1354	1	-0.59386
GALNT9	4	0.72974	0.75756	0.990873	1355	1	0.023427
NUDT2	4	0.73225	0.7591	0.990873	1356	1	-0.20226
PKM	4	0.73267	0.75934	0.990873	1357	1	-0.79263
ARSH	4	0.73308	0.75957	0.990873	1358	1	-0.08283
TKFC	4	0.7335	0.75981	0.990873	1359	1	-0.39094
PRDX3	4	0.73536	0.76086	0.990873	1360	1	-0.18221
COX8A	4	0.73763	0.76238	0.990873	1361	1	-0.13781
PGAM4	4	0.73825	0.7627	0.990873	1362	1	-0.61848
PHGDH	4	0.73988	0.76377	0.990873	1363	1	-0.20083

GGH	4	0.74009	0.76391	0.990873	1364	1	-0.18361
NUDT3	4	0.74273	0.76568	0.990873	1365	1	0.03347
GALNT3	4	0.74293	0.7658	0.990873	1366	1	-0.48058
CYP2C18	4	0.74313	0.76596	0.990873	1367	1	0.018704
COX10	4	0.74333	0.76608	0.990873	1368	1	-0.028559
SGPP1	4	0.74434	0.76672	0.990873	1369	1	-0.108
CLC	4	0.74454	0.76683	0.990873	1370	1	-0.12104
CYB5B	4	0.74615	0.76795	0.990873	1371	1	-0.23939
NDUFA6	4	0.74735	0.7687	0.990873	1372	1	-0.13332
SARDH	4	0.74755	0.76891	0.990873	1373	1	-0.3959
POMT2	4	0.75132	0.77129	0.990873	1374	1	-0.24801
CYP11A1	4	0.75151	0.77145	0.990873	1375	1	-0.41315
GPX1	4	0.7521	0.77183	0.990873	1376	1	0.11642
LIPE	4	0.75328	0.77268	0.990873	1377	1	-0.46887
SULT1B1	4	0.75348	0.77281	0.990873	1378	1	0.080728
ELOVL2	4	0.75426	0.77328	0.990873	1379	1	-0.41149
NIT1	4	0.75465	0.77359	0.990873	1380	1	-0.64233
ENOPH1	4	0.75485	0.7737	0.990873	1381	1	0.042238
ZADH2	4	0.75504	0.77386	0.990873	1382	1	-0.63038
APEH	4	0.75524	0.77395	0.990873	1383	1	0.12979
TKTL2	4	0.75737	0.77518	0.990873	1384	1	-1.3949
PLD2	4	0.75795	0.77558	0.990873	1385	1	-0.48426
LBR	4	0.75873	0.77604	0.990873	1386	1	-0.11088
HS3ST4	4	0.75911	0.77635	0.990873	1387	1	0.0095089
AGK	4	0.76122	0.77797	0.990873	1388	1	-0.31714
MSMO1	4	0.76141	0.77806	0.990873	1389	1	0.035234
GALNT12	4	0.76161	0.77818	0.990873	1390	1	-0.58136
AMPD2	4	0.76332	0.7794	0.990873	1391	1	-0.30428
GSTO2	4	0.76351	0.7795	0.990873	1392	1	-0.17475
GPX3	4	0.7654	0.78077	0.990873	1393	1	-0.15027
ELOVL1	4	0.76616	0.78123	0.990873	1394	1	-0.1583
UAP1	4	0.76691	0.78184	0.990873	1395	1	-0.27072
PDE5A	4	0.76729	0.78211	0.990873	1396	1	-1.5234
AKR1C4	4	0.7686	0.78302	0.990873	1397	1	0.21378
GPX8	4	0.76916	0.78339	0.990873	1398	1	0.076362
AS3MT	4	0.76972	0.78372	0.990873	1399	1	-0.823
HDC	4	0.7699	0.78389	0.990873	1400	1	-0.063126
GPT	4	0.77046	0.78425	0.990873	1401	1	-0.50841
PCYOX1	4	0.77083	0.78447	0.990873	1402	1	-0.37142
GPD1	4	0.77286	0.78588	0.990873	1403	1	0.12998
PTGS1	4	0.77378	0.78661	0.990873	1404	1	-0.19251
GLUD1	4	0.77598	0.78825	0.990873	1405	1	0.021386
TST	4	0.77635	0.78847	0.990873	1406	1	-0.47908
SULT1C2	4	0.77689	0.78883	0.990873	1407	1	-0.33817
PGM5	4	0.7787	0.79007	0.990873	1408	1	-0.057374
PLA2G3	4	0.77961	0.79076	0.990873	1409	1	-0.028778

PRDX2	4	0.77979	0.79089	0.990873	1410	1	-0.37146
AOAH	4	0.78051	0.79147	0.990873	1411	1	-0.50977
AADAC	4	0.78158	0.79226	0.990873	1412	1	-0.494
NPR1	4	0.78265	0.79301	0.990873	1413	1	0.049542
LIPN	4	0.78301	0.79332	0.990873	1414	1	-0.35197
PIP5KL1	4	0.78319	0.79342	0.990873	1415	1	-0.24033
CPT2	4	0.78337	0.79354	0.990873	1416	1	-0.72012
ALG9	4	0.78372	0.79385	0.990873	1417	1	-0.50202
HS3ST3B1	4	0.7839	0.79401	0.990873	1418	1	-0.35165
AKR1C2	4	0.78408	0.79414	0.990873	1419	1	-0.13311
LIPC	4	0.78514	0.79491	0.990873	1420	1	0.14238
GFPT1	4	0.78531	0.795	0.990873	1421	1	-0.14274
CYP27B1	4	0.78584	0.79546	0.990873	1422	1	-0.43193
CYP3A4	4	0.78602	0.79557	0.990873	1423	1	-0.21246
DHCR7	4	0.78655	0.79608	0.990873	1424	1	-0.31729
DGKH	4	0.78725	0.79668	0.990873	1425	1	-0.6202
PIK3R3	4	0.78743	0.79682	0.990873	1426	1	0.10346
GLOD4	4	0.78778	0.79705	0.990873	1427	1	-0.023124
HYAL3	4	0.78865	0.79771	0.990954	1428	1	-0.32645
NAPRT	4	0.7897	0.79864	0.990954	1429	1	0.019343
RPE	4	0.78987	0.7988	0.990954	1430	1	-0.17024
GUK1	4	0.79074	0.79952	0.991154	1431	1	-0.021202
UGT1A8	4	0.79178	0.80037	0.99152	1432	1	0.072043
PLCD1	4	0.79401	0.80212	0.991841	1433	1	-0.34248
FUT7	4	0.79452	0.8025	0.991841	1434	1	-0.48694
FOLH1	4	0.79538	0.80322	0.991841	1435	1	0.048044
GCNT2	4	0.79572	0.80342	0.991841	1436	1	-0.30702
PYCR1	4	0.79589	0.80352	0.991841	1437	1	-0.29605
GALNT15	4	0.79708	0.80448	0.991841	1438	1	0.014687
PHYHD1	4	0.79742	0.80472	0.991841	1439	1	-0.4634
B3GALNT2	4	0.79792	0.80514	0.991841	1440	1	-0.27701
PECR	4	0.7986	0.80566	0.991841	1441	1	-0.1836
HEXDC	4	0.80011	0.80683	0.992229	1442	1	-0.078267
UGT1A9	4	0.80044	0.8071	0.992229	1443	1	0.0043846
ITPA	4	0.80178	0.80818	0.992343	1444	1	-0.87518
ACLY	4	0.80194	0.80831	0.992343	1445	1	0.18955
PLPPR4	4	0.8041	0.81022	0.993212	1446	1	-0.86743
CYP4A11	4	0.80443	0.81049	0.993212	1447	1	0.053861
MAN2B1	4	0.80525	0.81109	0.993212	1448	1	-0.16079
GYS1	4	0.80607	0.81173	0.993212	1449	1	-0.20121
HS3ST1	4	0.80639	0.81202	0.993212	1450	1	-0.055068
DOLPP1	4	0.80737	0.81282	0.993212	1451	1	-1.3496
NT5C1B	4	0.80754	0.81293	0.993212	1452	1	-0.06451
ALDH1B1	4	0.80948	0.8145	0.994443	1453	1	-0.77231
AGA	4	0.81077	0.81566	0.994836	1454	1	-0.57801
SRD5A3	4	0.81189	0.81667	0.994836	1455	1	-0.16906

THNSL2	4	0.81221	0.81691	0.994836	1456	1	-0.18985
PLD6	4	0.81349	0.81805	0.994836	1457	1	0.020682
ASAH1	4	0.81396	0.81854	0.994836	1458	1	-0.52378
ALG6	4	0.81428	0.81883	0.994836	1459	1	-0.25899
MAN2A2	4	0.81507	0.81951	0.994836	1460	1	-0.34282
NUDT4	4	0.81539	0.81977	0.994836	1461	1	0.034062
GDA	4	0.81555	0.81987	0.994836	1462	1	-1.8185
ST6GALNAC3	4	0.8168	0.82086	0.995354	1463	1	-0.29793
HADH	4	0.81774	0.82171	0.995702	1464	1	-0.2375
NDUFV3	4	0.82007	0.82364	0.99621	1465	1	-0.40406
PLCXD3	4	0.82146	0.82483	0.99621	1466	1	-0.15348
ACBD3	4	0.82391	0.82694	0.99621	1467	1	-0.59999
GMPR	4	0.82406	0.82706	0.99621	1468	1	0.063747
FPGS	4	0.82437	0.82729	0.99621	1469	1	-0.57489
CEPT1	4	0.82467	0.82758	0.99621	1470	1	-0.36009
ADHFE1	4	0.82497	0.82782	0.99621	1471	1	-0.027232
ENO2	4	0.82513	0.82795	0.99621	1472	1	0.077936
SDR39U1	4	0.82603	0.82876	0.99621	1473	1	-0.19139
B3GNT8	4	0.82633	0.82902	0.99621	1474	1	0.093237
LDHAL6A	4	0.82784	0.83043	0.99621	1475	1	0.010197
CYP4Z1	4	0.82814	0.83064	0.99621	1476	1	-0.27231
FUT11	4	0.82843	0.83088	0.99621	1477	1	0.016305
IDH3A	4	0.82992	0.83214	0.99621	1478	1	-0.17154
PNLIPRP1	4	0.83037	0.83253	0.99621	1479	1	-0.16782
HSD17B2	4	0.83066	0.83275	0.99621	1480	1	-0.099597
HAO2	4	0.83096	0.833	0.99621	1481	1	-0.27125
FAAH	4	0.83125	0.83319	0.99621	1482	1	-0.16625
PIGG	4	0.83243	0.83425	0.99621	1483	1	-0.37578
NT5C2	4	0.83272	0.83451	0.99621	1484	1	-0.33216
BHMT	4	0.83287	0.83464	0.99621	1485	1	0.14455
EPHX1	4	0.83331	0.83503	0.99621	1486	1	-0.66865
UCK1	4	0.83375	0.83547	0.99621	1487	1	-1.1291
RRM1	4	0.83404	0.83575	0.99621	1488	1	-0.85574
SQLE	4	0.83664	0.83796	0.99621	1489	1	-0.19084
ARSF	4	0.83836	0.83959	0.99621	1490	1	-0.61073
MECR	4	0.83993	0.84094	0.99621	1491	1	-0.3141
ACOX2	4	0.84007	0.84105	0.99621	1492	1	-0.23733
KYAT3	4	0.84035	0.84136	0.99621	1493	1	-0.76139
ECI1	4	0.84106	0.84197	0.99621	1494	1	0.057814
NOS3	4	0.84204	0.84287	0.99621	1495	1	-0.04977
FAH	4	0.844	0.84464	0.99621	1496	1	-0.21755
PGS1	4	0.84456	0.8452	0.99621	1497	1	-0.16949
PLCG2	4	0.84497	0.84557	0.99621	1498	1	-0.092616
GALE	4	0.84621	0.84688	0.99621	1499	1	0.031601
CYP21A2	4	0.84704	0.84764	0.99621	1500	1	0.10461
GUCY2C	4	0.84717	0.8478	0.99621	1501	1	-0.36628

FUT3	4	0.84731	0.84793	0.99621	1502	1	-0.47024
MGST1	4	0.84745	0.84805	0.99621	1503	1	-0.31405
GSTA1	4	0.84772	0.84825	0.99621	1504	1	-0.98158
EHHADH	4	0.84813	0.84867	0.99621	1505	1	-0.37614
CYB5A	4	0.84989	0.85053	0.99621	1506	1	0.099228
ADH1B	4	0.85043	0.85111	0.99621	1507	1	-0.19069
ASMTL	4	0.85097	0.85164	0.99621	1508	1	-0.01027
PFKFB3	4	0.85124	0.85186	0.99621	1509	1	-0.27942
CHST3	4	0.85164	0.85223	0.99621	1510	1	-0.26539
UGT3A1	4	0.85191	0.85249	0.99621	1511	1	-0.3368
UGT2B4	4	0.85204	0.85262	0.99621	1512	1	-0.66774
PNPLA7	4	0.85218	0.85275	0.99621	1513	1	0.015509
ASRGL1	4	0.85324	0.85377	0.99621	1514	1	-0.61459
SORD	4	0.85404	0.85455	0.99621	1515	1	-0.12225
PDE6G	4	0.8543	0.85478	0.99621	1516	1	-0.56641
LIPM	4	0.85443	0.85486	0.99621	1517	1	-0.77589
CPT1A	4	0.85535	0.85582	0.99621	1518	1	-0.41101
CHI3L2	4	0.85549	0.85597	0.99621	1519	0	-0.45469
AMDHD1	4	0.85562	0.85605	0.99621	1520	0	-0.85717
PIK3C2A	4	0.85575	0.8562	0.99621	1521	0	-0.74916
CYP26C1	4	0.85653	0.85704	0.99621	1522	0	-0.24295
GSTM5	4	0.85679	0.85729	0.99621	1523	0	0.091462
ACSM5	4	0.85718	0.85764	0.99621	1524	0	-0.0046562
MTHFS	4	0.8577	0.85812	0.99621	1525	0	0.028205
SC5D	4	0.85796	0.85838	0.99621	1526	0	-0.87182
UGT1A6	4	0.85874	0.85914	0.99621	1527	0	-1.0763
GLTP	4	0.85887	0.85928	0.99621	1528	0	-0.24807
PHOSPHO2	4	0.85925	0.85969	0.99621	1529	0	-0.12325
GGT7	4	0.86003	0.8605	0.99621	1530	0	-0.31477
FA2H	4	0.86054	0.86096	0.99621	1531	0	-0.10914
PYGB	4	0.86143	0.86172	0.99621	1532	0	-0.28838
ACOT2	4	0.86194	0.86232	0.99621	1533	0	-0.27446
CYP11B2	4	0.86244	0.86283	0.99621	1534	0	-0.0091713
IMPAD1	4	0.8627	0.86315	0.99621	1535	0	-0.1997
CYB5D2	4	0.8632	0.86373	0.99621	1536	0	-0.051263
MTTP	4	0.86345	0.86389	0.99621	1537	0	-0.87261
SULT1A1	4	0.86371	0.8641	0.99621	1538	0	-0.50162
CTH	4	0.86396	0.86435	0.99621	1539	0	-0.080335
HK1	4	0.86446	0.8648	0.99621	1540	0	-0.36548
GPX4	4	0.86583	0.86617	0.996322	1541	0	-0.10296
AK4	4	0.86595	0.86631	0.996322	1542	0	-0.42928
DGAT2	4	0.8667	0.86703	0.996322	1543	0	-0.38655
NAGLU	4	0.86682	0.86715	0.996322	1544	0	-0.19714
GMPPB	4	0.86817	0.86844	0.99688	1545	0	-0.61321
GOT1	4	0.86927	0.86948	0.99688	1546	0	-0.71198
HYAL2	4	0.86951	0.86971	0.99688	1547	0	-0.18329

NUDT13	4	0.87048	0.87068	0.99688	1548	0	-0.2752
ITPK1	4	0.87097	0.87114	0.99688	1549	0	-0.46787
SDSL	4	0.87121	0.87145	0.99688	1550	0	-0.20056
AMPD3	4	0.87169	0.872	0.99688	1551	0	0.067818
ALOX12B	4	0.87181	0.87213	0.99688	1552	0	-0.18405
PDE1B	4	0.87318	0.87345	0.99688	1553	0	-0.99859
CHDH	4	0.87372	0.8739	0.99688	1554	0	0.062183
PLCB3	4	0.8749	0.87513	0.99688	1555	0	-0.31569
GBGT1	4	0.87596	0.87621	0.99688	1556	0	-0.051109
PNLIP	4	0.87666	0.87694	0.99688	1557	0	-0.15228
CHST2	4	0.87701	0.87727	0.99688	1558	0	-0.29921
THEM5	4	0.87782	0.87814	0.99688	1559	0	-0.3733
MGAT3	4	0.87794	0.87824	0.99688	1560	0	-0.08326
AMPD1	4	0.87805	0.87834	0.99688	1561	0	-0.22799
UROS	4	0.87909	0.87932	0.99688	1562	0	-1.2651
COX7A2L	4	0.88	0.88018	0.99688	1563	0	-0.12288
AK5	4	0.88023	0.88041	0.99688	1564	0	-0.2491
TGDS	4	0.88069	0.88093	0.99688	1565	0	-0.55819
PYGL	4	0.88092	0.88114	0.99688	1566	0	-0.12888
UGT1A4	4	0.88103	0.88126	0.99688	1567	0	-0.40877
GDPD4	4	0.88114	0.88135	0.99688	1568	0	-0.0071124
GAL3ST1	4	0.88193	0.88199	0.99688	1569	0	-0.042643
PTGS2	4	0.8825	0.88263	0.99688	1570	0	-0.52632
NANS	4	0.88306	0.88322	0.99688	1571	0	-0.040798
B3GNT4	4	0.88506	0.88525	0.99688	1572	0	-0.42743
CHIA	4	0.88693	0.88706	0.99688	1573	0	-0.1525
UGT2B7	4	0.88758	0.88769	0.99688	1574	0	-0.51378
GGT1	4	0.88796	0.88813	0.99688	1575	0	-0.31135
EXTL1	4	0.88813	0.88828	0.99688	1576	0	-0.050877
AKR1A1	4	0.88834	0.88851	0.99688	1577	0	-0.31457
GRHPR	4	0.88845	0.88859	0.99688	1578	0	-0.16901
NME5	4	0.88867	0.88883	0.99688	1579	0	-3.284
ALG5	4	0.89198	0.89235	0.99688	1580	0	-0.15063
PLA2G4A	4	0.8924	0.89268	0.99688	1581	0	-0.78666
B3GNT9	4	0.8925	0.89283	0.99688	1582	0	-0.24723
FMO1	4	0.89324	0.89352	0.99688	1583	0	-0.71903
GUCY2D	4	0.89344	0.8937	0.99688	1584	0	-1.2505
CBR4	4	0.89417	0.89443	0.99688	1585	0	-1.7044
SOD2	4	0.89428	0.89455	0.99688	1586	0	-0.13855
UPP1	4	0.89449	0.89475	0.99688	1587	0	-0.7155
HS6ST2	4	0.89459	0.89485	0.99688	1588	0	-0.5045
SEPHS2	4	0.89572	0.8958	0.99688	1589	0	-0.62845
GLB1L3	4	0.89685	0.89693	0.99688	1590	0	-0.09219
IMPDH2	4	0.89695	0.89703	0.99688	1591	0	-0.44715
PIGW	4	0.89807	0.89814	0.99688	1592	0	-1.211
COQ9	4	0.89817	0.89824	0.99688	1593	0	-0.46

DIO2	4	0.89827	0.89835	0.99688	1594	0	-0.12139
IPPK	4	0.89837	0.8985	0.99688	1595	0	-0.053343
DHDDS	4	0.89917	0.89934	0.99688	1596	0	-0.24404
DCTD	4	0.89958	0.89978	0.99688	1597	0	-0.49791
TXNRD1	4	0.90136	0.90157	0.99688	1598	0	-0.47568
PLPP4	4	0.90195	0.90219	0.99688	1599	0	-0.47929
MCCC1	4	0.90244	0.90269	0.99688	1600	0	-0.038358
HK2	4	0.90283	0.9031	0.99688	1601	0	-0.2593
MLYCD	4	0.90371	0.90393	0.99688	1602	0	-0.050995
LPIN3	4	0.90515	0.90547	0.99688	1603	0	-0.74223
L2HGDH	4	0.9061	0.9065	0.99688	1604	0	-0.41111
CYP4F11	4	0.90648	0.90682	0.99688	1605	0	0.010048
SPTLC3	4	0.90696	0.90724	0.99688	1606	0	-0.46751
RRM2	4	0.90724	0.90755	0.99688	1607	0	-0.95092
GALNT18	4	0.90761	0.90794	0.99688	1608	0	-0.72575
UQCRH	4	0.9079	0.90821	0.99688	1609	0	-1.216
PCK1	4	0.9092	0.90965	0.99688	1610	0	-0.82643
HS2ST1	4	0.90948	0.90993	0.99688	1611	0	-0.44593
MSRA	4	0.91003	0.91051	0.99688	1612	0	-0.35082
COLGALT1	4	0.91031	0.91074	0.99688	1613	0	-1.2958
AANAT	4	0.91049	0.91095	0.99688	1614	0	-0.17525
LYZL4	4	0.91113	0.91162	0.99688	1615	0	-0.21816
PIGO	4	0.91122	0.91175	0.99688	1616	0	-0.47884
MAT2B	4	0.91131	0.91182	0.99688	1617	0	-0.30967
HLCS	4	0.91168	0.91214	0.99688	1618	0	-0.34331
COX6A1	4	0.91177	0.91221	0.99688	1619	0	-0.02092
PNPLA1	4	0.91231	0.9127	0.99688	1620	0	-0.54689
TPH2	4	0.91525	0.9155	0.99688	1621	0	-0.00073146
NUDT17	4	0.91534	0.91561	0.99688	1622	0	-0.081249
NOS1	4	0.91586	0.91611	0.99688	1623	0	-0.36152
NUDT22	4	0.91613	0.91633	0.99688	1624	0	-0.3499
NNT	4	0.91656	0.91674	0.99688	1625	0	-0.27802
UGT1A5	4	0.91751	0.9178	0.99688	1626	0	-0.17196
GNPAT	4	0.91769	0.91797	0.99688	1627	0	-1.2168
TH	4	0.9188	0.91894	0.99688	1628	0	-0.31653
HSD11B2	4	0.91939	0.91955	0.99688	1629	0	-0.079209
COX7B	4	0.91965	0.91979	0.99688	1630	0	-1.403
CKMT1B	4	0.92041	0.92062	0.99688	1631	0	-0.48872
MAT1A	4	0.92074	0.92092	0.99688	1632	0	-0.1237
BTD	4	0.92141	0.9216	0.99688	1633	0	-0.37927
PIK3R2	4	0.92199	0.92212	0.99688	1634	0	-0.48455
ACSM2B	4	0.9224	0.92255	0.99688	1635	0	-0.52715
PCYOX1L	4	0.92248	0.92266	0.99688	1636	0	-0.23592
GUCY1A3	4	0.92289	0.92303	0.99688	1637	0	-0.10547
DHRS7C	4	0.92298	0.92311	0.99688	1638	0	-0.38907

B4GALT1	4	0.92306	0.92319	0.99688	1639	0	-0.18582
PLA2G16	4	0.92314	0.92328	0.99688	1640	0	-0.13475
PHOSPHO1	4	0.9233	0.92344	0.99688	1641	0	-0.10113
NT5M	4	0.92444	0.92453	0.99688	1642	0	-0.31529
HACL1	4	0.9246	0.92472	0.99688	1643	0	-1.0951
NAT6	4	0.925	0.92511	0.99688	1644	0	-0.065162
CES3	4	0.92564	0.92564	0.99688	1645	0	-0.22437
B4GALT7	4	0.92588	0.92593	0.99688	1646	0	-0.33784
PGAM5	4	0.9273	0.92741	0.99688	1647	0	-0.20455
AGPAT4	4	0.92754	0.92761	0.99688	1648	0	-0.82382
IDUA	4	0.92769	0.92777	0.99688	1649	0	-0.47887
GPLD1	4	0.92801	0.92803	0.99688	1650	0	-0.30923
COX5A	4	0.92886	0.92891	0.99688	1651	0	-0.18814
PGM2L1	4	0.9297	0.92974	0.99688	1652	0	-0.79024
NDUFS8	4	0.92978	0.92978	0.99688	1653	0	-0.56282
ACADM	4	0.92986	0.92986	0.99688	1654	0	-1.0831
ARSG	4	0.93001	0.93001	0.99688	1655	0	-0.20504
PGM1	4	0.93367	0.93377	0.998745	1656	0	-0.28267
CA1	4	0.93403	0.93414	0.998745	1657	0	-0.37643
DHRS2	4	0.94036	0.94049	0.998745	1658	0	-0.050728
INPP4B	4	0.94056	0.94069	0.998745	1659	0	-0.455
DPYSL4	4	0.94076	0.94088	0.998745	1660	0	-0.83435
LIPJ	4	0.94183	0.94196	0.998745	1661	0	-0.20664
EXTL2	4	0.94189	0.94205	0.998745	1662	0	-0.86452
PIGP	4	0.94249	0.94265	0.998745	1663	0	-0.63548
KYAT1	4	0.94321	0.94344	0.998745	1664	0	-0.21323
CYP7A1	4	0.94366	0.94385	0.998745	1665	0	-0.26337
UGT2B11	4	0.94386	0.94402	0.998745	1666	0	-0.46284
ME3	4	0.94463	0.94473	0.998745	1667	0	-0.35612
NDOR1	4	0.94488	0.94497	0.998745	1668	0	-0.17693
FUT9	4	0.94596	0.94598	0.998745	1669	0	-0.35977
ENPP1	4	0.94652	0.94657	0.998745	1670	0	-0.74643
B3GNT5	4	0.94702	0.94712	0.998745	1671	0	-0.29201
EXT1	4	0.9472	0.94731	0.998745	1672	0	-1.227
MMAB	4	0.94745	0.94765	0.998745	1673	0	-0.065592
GCNT7	4	0.94763	0.94785	0.998745	1674	0	-0.1785
OGDHL	4	0.94776	0.94798	0.998745	1675	0	-0.48851
CERK	4	0.94794	0.94811	0.998745	1676	0	-0.11981
SDHD	4	0.948	0.94815	0.998745	1677	0	-0.92686
NAGS	4	0.94806	0.94824	0.998745	1678	0	-0.62804
ST8SIA6	4	0.94831	0.94848	0.998745	1679	0	-0.30838
NFS1	4	0.94903	0.94919	0.998745	1680	0	-0.54894
GALNT10	4	0.94975	0.94987	0.998745	1681	0	-0.53909
AK2	4	0.94981	0.94995	0.998745	1682	0	-0.15899
CA3	4	0.95016	0.95035	0.998745	1683	0	-0.19756
CKMT2	4	0.9511	0.95134	0.998745	1684	0	-0.47057

TAT	4	0.95133	0.95152	0.998745	1685	0	-0.30112
DEGS1	4	0.95145	0.95165	0.998745	1686	0	-0.38713
PGAP1	4	0.9518	0.95202	0.998745	1687	0	-0.14353
GAD1	4	0.95367	0.95371	0.998745	1688	0	-0.41873
CLYBL	4	0.95389	0.95391	0.998745	1689	0	-0.44653
PPAT	4	0.95395	0.95398	0.998745	1690	0	-0.19273
HSD17B14	4	0.95434	0.95431	0.998745	1691	0	-0.77272
CDADC1	4	0.9545	0.95444	0.998745	1692	0	-0.52503
NDUFA7	4	0.9557	0.95563	0.998745	1693	0	-0.39952
CA6	4	0.9564	0.95631	0.998745	1694	0	-0.26115
ELOVL3	4	0.95656	0.95648	0.998745	1695	0	-1.0124
GPAM	4	0.95767	0.95762	0.998745	1696	0	-0.41142
BPHL	4	0.95871	0.95848	0.998745	1697	0	-0.63586
BLVRB	4	0.95937	0.95907	0.998745	1698	0	-0.3625
TYMP	4	0.95952	0.95918	0.998745	1699	0	-0.38313
PDHA1	4	0.95997	0.95972	0.998745	1700	0	-0.63097
PIK3R5	4	0.96003	0.95977	0.998745	1701	0	-0.40787
GNPTG	4	0.96013	0.95986	0.998745	1702	0	-0.20106
GCK	4	0.96018	0.95989	0.998745	1703	0	-0.30673
NOX4	4	0.96022	0.95994	0.998745	1704	0	-0.43434
OAS1	4	0.96121	0.96114	0.998745	1705	0	-0.39703
IDI2	4	0.96175	0.96163	0.998745	1706	0	-0.80802
LNPEP	4	0.96261	0.96236	0.998745	1707	0	-0.37533
CYP19A1	4	0.96309	0.96277	0.998745	1708	0	-0.36145
COMT	4	0.96346	0.96316	0.998745	1709	0	-0.44004
MGAT4B	4	0.9636	0.96332	0.998745	1710	0	-0.30488
GLS	4	0.96601	0.96558	0.998745	1711	0	-0.38858
ALDOB	4	0.9661	0.96571	0.998745	1712	0	-0.62991
INPP5E	4	0.96659	0.96625	0.998745	1713	0	-0.39905
PLCB2	4	0.96702	0.96669	0.998745	1714	0	-0.52482
UCKL1	4	0.96754	0.96721	0.998745	1715	0	-0.43402
GLYATL2	4	0.96914	0.96884	0.998745	1716	0	-0.33614
PDE6B	4	0.97048	0.97037	0.998745	1717	0	-0.59073
ADH5	4	0.97115	0.97101	0.998745	1718	0	-0.31433
SRM	4	0.97127	0.97113	0.998745	1719	0	-0.64498
SDHB	4	0.97181	0.97167	0.998745	1720	0	-1.2972
MICAL1	4	0.97204	0.97188	0.998745	1721	0	-0.31288
DTYMK	4	0.97215	0.97198	0.998745	1722	0	-0.3586
IDH3B	4	0.9731	0.97301	0.998745	1723	0	-0.47788
DHFR	4	0.97324	0.97317	0.998745	1724	0	-0.62115
CTPS2	4	0.97339	0.97332	0.998745	1725	0	-0.36615
PIGA	4	0.97365	0.97356	0.998745	1726	0	-1.0354
BAAT	4	0.9743	0.9742	0.998745	1727	0	-1.399
GLOD5	4	0.97466	0.97457	0.998745	1728	0	-0.47615
PLPP3	4	0.97547	0.97542	0.998745	1729	0	-0.57182
ST6GALNAC1	4	0.97592	0.97586	0.998745	1730	0	-0.64309

PCBD1	4	0.97693	0.97686	0.998745	1731	0	-0.84442
LCAT	4	0.9781	0.97788	0.998745	1732	0	-0.96112
ACADVL	4	0.97854	0.97839	0.998745	1733	0	-0.60155
APRT	4	0.97929	0.97914	0.998745	1734	0	-0.68977
SULT1E1	4	0.98034	0.98026	0.998745	1735	0	-3.689
GLRX2	4	0.98045	0.98038	0.998745	1736	0	-7.3538
CYBRD1	4	0.98092	0.9809	0.998745	1737	0	-0.89264
PLB1	4	0.98117	0.98113	0.998745	1738	0	-0.51184
MGAT2	4	0.98174	0.98176	0.998745	1739	0	-0.35267
GPAT4	4	0.9834	0.9834	0.998745	1740	0	-0.62728
NDUFAF2	4	0.98459	0.98457	0.998745	1741	0	-0.89067
GFOD1	4	0.98466	0.98467	0.998745	1742	0	-0.74391
MGAT5B	4	0.98468	0.98468	0.998745	1743	0	-0.85563
CYBA	4	0.98524	0.98526	0.998745	1744	0	-0.44596
AOX1	4	0.98564	0.9856	0.998745	1745	0	-0.77504
MOCS1	4	0.9865	0.98657	0.998745	1746	0	-0.36722
MOGAT1	4	0.98657	0.98661	0.998745	1747	0	-0.53855
ABHD4	4	0.98687	0.98703	0.998745	1748	0	-0.56567
GAPDH	4	0.9872	0.98729	0.998745	1749	0	-0.6032
AHCYL1	4	0.98755	0.98762	0.998745	1750	0	-0.45128
TM7SF2	4	0.9878	0.98786	0.998745	1751	0	-1.1319
ADAL	4	0.98793	0.988	0.998745	1752	0	-0.77629
MANBA	4	0.98849	0.98856	0.998745	1753	0	-3.4783
RDH12	4	0.98908	0.98909	0.998745	1754	0	-0.55262
CKM	4	0.98931	0.98929	0.998745	1755	0	-0.57728
HSD3B1	4	0.98982	0.98981	0.998745	1756	0	-1.2427
ADH1C	4	0.99018	0.99021	0.998745	1757	0	-1.102
EDEM3	4	0.99052	0.99057	0.998745	1758	0	-0.87923
GALNT8	4	0.99054	0.99058	0.998745	1759	0	-0.66719
PLA2G10	4	0.9913	0.99141	0.998745	1760	0	-1.063
LDHAL6B	4	0.9915	0.99158	0.998745	1761	0	-1.0067
IDH3G	4	0.99232	0.99237	0.998745	1762	0	-0.93523
GNPTAB	4	0.99253	0.99255	0.998745	1763	0	-0.60113
ACAT2	4	0.99351	0.99349	0.998931	1764	0	-0.98949
ACOT12	4	0.99461	0.99453	0.998931	1765	0	-1.1661
NDUFV2	4	0.99518	0.99509	0.998931	1766	0	-1.9616
NNMT	4	0.99543	0.9953	0.998931	1767	0	-0.65093
BBOX1	4	0.99632	0.99636	0.998931	1768	0	-0.92395
MUT	4	0.99749	0.99745	0.998931	1769	0	-0.65871
FN3K	4	0.99754	0.9975	0.998931	1770	0	-0.45744
PIPOX	4	0.99789	0.99787	0.998931	1771	0	-0.58187
EDEM1	4	0.99818	0.99814	0.998931	1772	0	-1.0972
INPP5D	4	0.99838	0.99837	0.998931	1773	0	-0.89027
AKR1B1	4	0.99947	0.99946	0.999461	1774	0	-1.0116
