# Supplementary material for: The AHCY–adenosine complex rewires mRNA methylation to enhance fatty acid biosynthesis and tumorigenesis
Source: Cell Res. 2026 Jan 19;36(2):152–72. doi: 10.1038/s41422-025-01213-5 (PMC12848013; doi:10.1038/s41422-025-01213-5)
Supplement: Supplementary file 13 — Supplementary information, Table S3 [file 41422_2025_1213_MOESM13_ESM.pdf]

Table S3. Sequences of the biotin-m6A motif and the biotin-ACACA fragments containing the GGAC m6A motif.

Name	Sequence (5' to 3')	Modification
Control	AUUGUCAACAGCAGC	3'-Biotin
Biotin-m6A	AUUGUCA(m6A)CAGCAGC	3'-Biotin
Biotin-CDS1	CCGCAGCAATAAGAATGTTTGGGGATATTTCAGTGTTGCTGCTGCAGGGGG(m6A)CTTCATGAATTTGCTGAT	3'-Biotin
Biotin-3'UTR-1	CAGTGAAGGCTTATGTTTGGG(m6A)CAATAATAAGGATCTGGCGGAGTGGCTAGAGAAACAGCTGACAGAGGA	3'-Biotin
Biotin-3'UTR-2	AGTCCTCCACTCCCTGCACAGG(m6A)CTGAGAAGGCAATGAAAGGTAC	3'-Biotin
Biotin-3'UTR-3	TAGATAGATAGTCTGTTTGTTTTGAGG(m6A)CTTGGAAAGTTGTTCCTATGAAGCCTGGAGCTTGGATGGTTT	3'-Biotin
