# Supplementary material for: The AHCY–adenosine complex rewires mRNA methylation to enhance fatty acid biosynthesis and tumorigenesis
Source: Cell Res. 2026 Jan 19;36(2):152–72. doi: 10.1038/s41422-025-01213-5 (PMC12848013; doi:10.1038/s41422-025-01213-5)
Supplement: Supplementary file 14 — Supplementary information, Table S4 [file 41422_2025_1213_MOESM14_ESM.pdf]

Table S4. Molecular dynamics simulation and binding free energy calculation for AHCY dimer-perturbing peptides by the MM/GBSA method.

Name	Sequence	EVDW	EELE	EGB	ESURF	ΔH
AA 1#	VTKSKFDNLYGCRESLIDGIKRAT	-47.80±0.96	-322.69±8.71	345.81±9.21	-7.44±0.13	-32.13±1.45
AA 2#	TKSKFDNLYGCRESLIDGIKRAT	-42.20±1.93	-270.76±9.13	290.45±6.98	-7.01±0.21	-29.52±1.44
AA 3#	KSKFDNLYGCRESLIDGIKRAT	-42.83±1.19	-253.81±8.68	277.54±7.44	-7.27±0.09	-26.38±1.02
AA 4#	SKFDNLYGCRESLIDGIKRAT	-33.41±1.26	-200.44±8.88	214.82±8.22	-5.29±0.25	-24.32±1.90
AA 5#	KFDNLYGCRESLIDGIKRAT	-37.28±1.43	-210.77±7.28	235.13±7.63	-6.05±0.19	-18.98±1.32
AA 6#	FDNLYGCRESLIDGIKRAT	-41.32±0.98	-176.09±5.29	187.06±4.73	-7.20±0.12	-37.55±1.26
AA 7#	DNLYGCRESLIDGIKRAT	-43.12±0.85	-235.30±4.26	246.60±3.58	-7.47±0.10	-39.29±1.15
AA 8#	NLYGCRESLIDGIKRAT	-33.05±1.13	-250.87±8.67	260.23±6.93	-5.99±0.10	-29.69±1.57
AA 9#	LYGCRESLIDGIKRAT	-26.25±1.35	-220.79±9.96	228.18±9.03	-4.76±0.24	-23.62±1.65
AA 10#	YGCRESLIDGIKRAT	-32.75±2.15	-195.86±6.98	208.28±7.08	-6.05±0.29	-26.38±1.96
AA 11#	GCRESLIDGIKRAT	-25.12±1.18	-268.61±6.53	280.51±6.13	-4.93±0.16	-18.14±1.25
