# Supplementary material for: The AHCY–adenosine complex rewires mRNA methylation to enhance fatty acid biosynthesis and tumorigenesis
Source: Cell Res. 2026 Jan 19;36(2):152–72. doi: 10.1038/s41422-025-01213-5 (PMC12848013; doi:10.1038/s41422-025-01213-5)
Supplement: Supplementary file 15 — Supplementary information, Table S5 [file 41422_2025_1213_MOESM15_ESM.pdf]

Table S5. The primer sequences used for construction of the mutants.

Mutant	Primer	Sequence (5' to 3')
AHCY-H55A	Forward	GCCATGACCGTGGAGACGGCCGTCC
	Reverse	TCTCCACGGTCATGGCCAGGCAGCCAGC
AHCY-E156A	Forward	CACCACGACTGGGGTCCACAAC
	Reverse	CCAGTCGTGGTGTCCTCAGAGATGC
AHCY-K426R	Forward	GGCCGGATCACTACCGCTAC
	Reverse	GTAGTGATCCGGCCTGAAGGGGC
AHCY-D245A-ABI	Forward	CCCCCATCAACGCACTGCAGGC
	Reverse	CGTTGATGGGGGCAATCTCGGTGATGATG
ADA 2DA-PYL1	Forward 1	TCAAGTCCACCCTGGACACTG
	Reverse 1	GTTGAGCGAGTAGTTAGCCTGG
	Forward 2	ACAGCTGCACCGCTCATCTTCAAGTCCACCCTGGACACTG
	Reverse 2	AGATGAGCGGTGCAGCTGTGTTGAGCGAGTAGTTAGCCTGG
AHCY-E59A	Forward	CGACGGCCGTCCTCATTGAGACCCTC
	Reverse	GACGGCCGTCGCCACGGTCATG
AHCY-T157A	Forward	GCCACGACTGGGGTCCACAACCTC
	Reverse	CCCAGTCGTGGCCTCCTCAGAGATGC
AHCY Δ388-397	Forward 1	AAGTTGACCAAGCTAACTGAGAAGCAAG
	Reverse 1	GGGCAGGAAATGAACCCCAACG
	Forward 2	CACCTGGGCAAGCTGAATGTGAAGTTGACCAAGCTAACTGAGAAGCAAG
	Reverse 2	CACATTCAGCTTGCCCAGGTGGGGCAGGAAATGAACCCCAACG
AHCY-Y193H	Forward	CATGGCTGCCGGGAGTCCCT
	Reverse	CCCGGCAGCCGTAGAGGTTGTCA
AHCY-Y193K	Forward	AAAGGCTGCCGGGAGTCCCT
	Reverse	CCCGGCAGCCTTTGAGGTTGTCA
AHCY-Y193F	Forward	TTGGCTGCCGGGAGTCCCT
	Reverse	CCGGCAGCCAAAGAGGTTGTCA
AHCY Δ1-183	Forward 1	ACCAAGAGCAAGTTTGACAACCTC
	Reverse 1	GGAACAGAACTTCCAGATCCGATTTTGG
	Forward 2	AGGGGCCCCTGGGATCCACCAAGAGCAAGTTTGACAACCTC
	Reverse 2	GGATCCCAGGGGCCCCTGGAACAGAACTTCCAGATCCGATTTTGG
AHCY Δ184-356	Forward 1	ATGAGTAACTCCTTCACCAACCAGG
	Reverse 1	GATGGCAGGCACCTTGAGGAT
	Forward 2	AATGTCAATGACTCCGTCATGAGTAACTCCTTCACCAACCAGG
	Reverse 2	GACGGAGTCATTGACATTGATGGCAGGCACCTTGAGGAT
AHCY Δ357-390	Forward 1	GAGGCAGTGGCTGAAGCCCAC
	Reverse 1	CATGGCACAACCCAGGTTGACCAG
	Forward 2	GGCCACCCCAGCTTCGTGGAGGCAGTGGCTGAAGCCCAC
	Reverse 2	CACGAAGCTGGGGTGGCCCATGGCACAACCCAGGTTGACCAG
AHCY Δ391-431	Forward 1	TAAGCGGCCGCATCGTGACTG
	Reverse 1	AAATGAACCCCAACGGGGTACTTG
	Forward 2	CCTGCCCAAGAAGCTGGATTAAGCGGCCGCATCGTGACTG
	Reverse 2	ATCCAGCTTCTTGGGCAGGAAATGAACCCCAACGGGGTACTTG

AHCY-D11A	Forward	CCATCGGCCTGGCTGCCTGG
	Reverse	CCAGGCCGATGGCGGCGACTTTGTAG
AHCY-T158N	Forward	GAGGAGACCAACACTGGGGTCCACAACCTCTACAAG
	Reverse	GTGGACCCCAGTGTTGGTCTCCTCAGAGATGCCTCG
AHCY-H162N	Forward	ACGACTGGGGTCAACAACCTCTACAAGA
	Reverse	TGACCCCAGTCGTGGTCTCCTCAG
AHCY-D208A	Forward	GCGTCATGATCGCCGGCAAGGTAG
	Reverse	GCGATCATGACGCCAGTCGCTCTCTTGATG
AHCY-E243A	Forward	CGATTGACCCCATCAACGCACTGCAG
	Reverse	TGATGGGGTCAATCGCGGTGATGATGACGC
AHCY-D245A	Forward	CCCCCATCAACGCACTGCAGGC
	Reverse	TGCGTTGATGGGGGCAATCTCGGTGATG
AHCY-Q251A	Forward	TGGCTGCCATGGAGGGCTATGAGG
	Reverse	CCATGGCAGCCAGCAGTGCGTTGATG
AHCY-C278A	Forward	GCCATAGATATTATCCTTGGCCGGCACTTTGA
	Reverse	CCAAGGATAATATCTATGGCCCCAGTCGTGGTGACAAAGA
AHCY-D280A	Forward	GCTATTATCCTTGGCCGGCACTTTGAGCAG
	Reverse	CGGCCAAGGATAATAGCTATGCACCCAGTC
FTO-Q86A	Forward	AGATCTTTGCCTGCGATCCTAACCAGGTCCCGA
	Reverse	GCACACTGTGTTTTGGCCGGTTCA
FTO-Q306A	Forward	CAAAACACAGTGTGCGTGGGTGGCATTGAGATC
	Reverse	GCCATCCTCATTGGTAATCCAGGCTGC
FTO-R96A	Forward	GCCATCCTCATTGGTAATCCAGGCTGC
	Reverse	ACCAATGAGGATGGCAGATACCGGAGTGAGCAGATC
FTO-Y108A	Forward	GCCCTGAACACCAGGCTCTTTACGGTC
	Reverse	CTGGTGTTCAGGGCCTTGTAGGTGCAGCCTG
FTO-E234A	Forward	CAAATCTGGTGGACAGGTCAGCGG
	Reverse	TGTCCACCAGATTTGCATCATGATGCCAGCTC
AHCY Δ184-187	Forward 1	CGGGAGTCCCTCATAGATGGCATC
	Reverse 1	GGAGTCATTGACATTGATGGCAGGC
	Forward 2	AAGTTTGACAACCTCTATGGCTGCCGGGAGTCCCTCATAGATGGCATC
	Reverse 2	GCAGCCATAGAGGTTGTCAAACTTGGAGTCATTGACATTGATGGCAGGC
AHCY Δ190-207	Forward 1	TAGCGGTGGTAGCAGGCTATGG
	Reverse 1	AAACTTGCTCTTGGTGACGGAGTC
	Forward 2	GATGTGATGATTGCCGGCAAGGTAGCGGTGGTAGCAGGCTATGG
	Reverse 2	CCTTGCCGGCAATCATCACATCAAACTTGCTCTTGGTGACGGAGTC
