# Supplementary material for: The AHCY–adenosine complex rewires mRNA methylation to enhance fatty acid biosynthesis and tumorigenesis
Source: Cell Res. 2026 Jan 19;36(2):152–72. doi: 10.1038/s41422-025-01213-5 (PMC12848013; doi:10.1038/s41422-025-01213-5)
Supplement: Supplementary file 16 — Supplementary information, Table S6 [file 41422_2025_1213_MOESM16_ESM.pdf]

Table S6. Sense and antisense shRNA oligonucleotides.

shRNA	Primer	Sequence (5' to 3')
AHCY-shRNA1	Forward	CCGGCGGGCCACAGATGTGATGATTCTCGAGAATCATCACATCTGTGGCCCGTTTTTG
	Reverse	AATTCAAAAACGGGCCACAGATGTGATGATTCTCGAGAATCATCACATCTGTGGCCCG
AHCY-shRNA2	Forward	CCGGCACAGGCTGTATTGACATCATCTCGAGATGATGTCAATACAGCCTGTGTTTTTG
	Forward	AATTCAAAAACACAGGCTGTATTGACATCATCTCGAGATGATGTCAATACAGCCTGTG
CYC1-shRNA1	Forward	CCGGCCAGGGAAGCTGTTCGACTATCTCGAGATAGTCGAACAGCTTCCCTGGTTTTTG
	Reverse	AATTCAAAAACCAGGGAAGCTGTTCGACTATCTCGAGATAGTCGAACAGCTTCCCTGG
CYC1-shRNA2	Forward	CCGGGCTGTTCGACTATTTCCCAAACTCGAGTTTGGGAAATAGTCGAACAGCTTTTTG
	Forward	AATTCAAAAAGCTGTTCGACTATTTCCCAAACTCGAGTTTGGGAAATAGTCGAACAGC
AMY2B-shRNA1	Forward	CCGGGCGTTCCAAGATTGCCGAATACTCGAGTATTCGGCAATCTTGGAACGCTTTTTG
	Reverse	AATTCAAAAAGCGTTCCAAGATTGCCGAATACTCGAGTATTCGGCAATCTTGGAACGC
AMY2B-shRNA2	Forward	CCGGTGAATCATCTCATTGACATTGCTCGAGCAATGTCAATGAGATGATTCATTTTTG
	Forward	AATTCAAAAATGAATCATCTCATTGACATTGCTCGAGCAATGTCAATGAGATGATTCA
ASAH2B-shRNA1	Forward	CCGGACGCATTATCTGCTTACATTCCTCGAGGAATGTAAGCAGATAATGCGTTTTTTG
	Reverse	AATTCAAAAAACGCATTATCTGCTTACATTCCTCGAGGAATGTAAGCAGATAATGCGT
ASAH2B-shRNA2	Forward	CCGGGGAAGTTGCTGAAGTTATATTCTCGAGAATATAACTTCAGCAACTTCCTTTTTG
	Forward	AATTCAAAAAGGAAGTTGCTGAAGTTATATTCTCGAGAATATAACTTCAGCAACTTCC
MAT2A-shRNA1	Forward	CCGGTTTGGAGGACGTACGTAATAACTCGAGTTATTACGTACGTCCTCCAAATTTTTG
	Reverse	AATTCAAAAATTTGGAGGACGTACGTAATAACTCGAGTTATTACGTACGTCCTCCAAA
MAT2A-shRNA2	Forward	CCGGAGCAGTTGTGCCTGCGAAATACTCGAGTATTTCGCAGGCACAACTGCTTTTTTG
	Reverse	AATTCAAAAAAGCAGTTGTGCCTGCGAAATACTCGAGTATTTCGCAGGCACAACTGCT
FTO-shRNA1	Forward	CCGGCGGTTCACAACCTCGGTTTAGCTCGAGCTAAACCGAGGTTGTGAACCGTTTTTG
	Reverse	AATTCAAAAACGGTTCACAACCTCGGTTTAGCTCGAGCTAAACCGAGGTTGTGAACCG
FTO-shRNA2	Forward	CCGGTCGCATGGCAGCAAGCTAAATCTCGAGATTTAGCTTGCTGCCATGCGATTTTTG
	Reverse	AATTCAAAAATCGCATGGCAGCAAGCTAAATCTCGAGATTTAGCTTGCTGCCATGCGA
ALKBH5-shRNA1	Forward	CCGGGAAAGGCTGTTGGCATCAATACTCGAGTATTGATGCCAACAGCCTTTCTTTTTG
	Reverse	AATTCAAAAAGAAAGGCTGTTGGCATCAATACTCGAGTATTGATGCCAACAGCCTTTC
ALKBH5-shRNA2	Forward	CCGGGAAAGGCTGTTGGCATCAATACTCGAGTATTGATGCCAACAGCCTTTCTTTTTG
	Reverse	AATTCAAAAAGAAAGGCTGTTGGCATCAATACTCGAGTATTGATGCCAACAGCCTTTC
METTL3-shRNA1	Forward	CCGGGCTGCACTTCAGACGAATTATCTCGAGATAATTCGTCTGAAGTGCAGCTTTTTG
	Reverse	AATTCAAAAAGCTGCACTTCAGACGAATTATCTCGAGATAATTCGTCTGAAGTGCAGC
METTL3-shRNA2	Forward	CCGGGCTGCACTTCAGACGAATTATCTCGAGATAATTCGTCTGAAGTGCAGCTTTTTG
	Reverse	AATTCAAAAAGCTGCACTTCAGACGAATTATCTCGAGATAATTCGTCTGAAGTGCAGC
METTL14-shRNA1	Forward	CCGGGAAAGGCTGTTGGCATCAATACTCGAGTATTGATGCCAACAGCCTTTCTTTTTG
	Reverse	AATTCAAAAAGAAAGGCTGTTGGCATCAATACTCGAGTATTGATGCCAACAGCCTTTC
METTL14-shRNA2	Forward	CCGGAGGATGAGTTAATAGCTAAATCTCGAGATTTAGCTATTAACTCATCCTTTTTTG
	Reverse	AATTCAAAAAAGGATGAGTTAATAGCTAAATCTCGAGATTTAGCTATTAACTCATCCT
ADA-shRNA1	Forward	CCGGAGAAGACCATGATCTCAATAGCTCGAGCTATTGAGATCATGGTCTTCTTTTTTG
	Reverse	AATTCAAAAAAGAAGACCATGATCTCAATAGCTCGAGCTATTGAGATCATGGTCTTCT
ADA-shRNA2	Forward	CCGGTGAACCCTATGTGTCCATTTCCTCGAGGAAATGGACACATAGGGTTCATTTTTG
	Reverse	AATTCAAAAATGAACCCTATGTGTCCATTTCCTCGAGGAAATGGACACATAGGGTTCA
ADORA2A-shRNA1	Forward	CCGGCCTAAGGGAAGGAGATCTTTACTCGAGTAAAGATCTCCTTCCCTTAGGTTTTTG
	Reverse	AATTCAAAAACCTAAGGGAAGGAGATCTTTACTCGAGTAAAGATCTCCTTCCCTTAGG

ADORA2A-shRNA2	Forward	CCGGTGCTCATGCTGGGTGTCTATTCTCGAGAATAGACACCCAGCATGAGCATTTTTG
	Reverse	AATTCAAAAATGCTCATGCTGGGTGTCTATTCTCGAGAATAGACACCCAGCATGAGCA
ADORA2B-shRNA1	Forward	CCGGGCTAATATGTATGTGTCAGTACTCGAGTACTGACACATACATATTAGCTTTTTG
	Reverse	AATTCAAAAAGCTAATATGTATGTGTCAGTACTCGAGTACTGACACATACATATTAGC
ADORA2B-shRNA2	Forward	CCGGGCTAATATGTATGTGTCAGTACTCGAGTACTGACACATACATATTAGCTTTTTG
	Reverse	AATTCAAAAAGCTAATATGTATGTGTCAGTACTCGAGTACTGACACATACATATTAGC
CD39-shRNA1	Forward	CCGGCCCAGATAATGCTCTGCAATTCTCGAGAATTGCAGAGCATTATCTGGGTTTTTG
	Reverse	AATTCAAAAACCCAGATAATGCTCTGCAATTCTCGAGAATTGCAGAGCATTATCTGGG
CD39-shRNA2	Forward	CCGGGCACCAAGAGACACCCGTTTACTCGAGTAAACGGGTGTCTCTTGGTGCTTTTTG
	Reverse	AATTCAAAAAGCACCAAGAGACACCCGTTTACTCGAGTAAACGGGTGTCTCTTGGTGC
CD73-shRNA1	Forward	CCGGGCACTGGGAAATCATGAATTTCTCGAGAAATTCATGATTTCCCAGTGCTTTTTG
	Reverse	AATTCAAAAAGCACTGGGAAATCATGAATTTCTCGAGAAATTCATGATTTCCCAGTGC
CD73-shRNA2	Forward	CCGGGCACTGGGAAATCATGAATTTCTCGAGAAATTCATGATTTCCCAGTGCTTTTTG
	Reverse	AATTCAAAAAGCACTGGGAAATCATGAATTTCTCGAGAAATTCATGATTTCCCAGTGC
