# Supplementary material for: The AHCY–adenosine complex rewires mRNA methylation to enhance fatty acid biosynthesis and tumorigenesis
Source: Cell Res. 2026 Jan 19;36(2):152–72. doi: 10.1038/s41422-025-01213-5 (PMC12848013; doi:10.1038/s41422-025-01213-5)
Supplement: Supplementary file 17 — Supplementary information, Table S7 [file 41422_2025_1213_MOESM17_ESM.pdf]

Table S7. Primer sequences used for qPCR, PCR, and CLIP/RIP-qPCR analysis.

Gene	Primer	Sequence (5' to 3')
18S (qPCR)	Forward	CTACCACATCCAAGGAAGCA
	Reverse	TTTTTCGTCACTACCTCCCCG
ACTIN (qPCR)	Forward	GTGACGTTGACATCCGTAAAGA
	Reverse	GCCGGACTCATCGTACTC
GAPDH (qPCR)	Forward	GGAGCGAGATCCCTCCAAAAT
	Forward	GGCTGTTGTCATACTTCTCAGG
AHCY (qPCR)	Forward	ATTCCGGTGTATGCCTGGAAG
	Reverse	GAGATGCCTCGGATGCCTG
MAT2A (qPCR)	Forward	ATGAACGGACAGCTCAACGG
	Reverse	CCAGCAAGAAGGATCATTCCAG
ALKBH5 (qPCR)	Forward	CGGCGAAGGCTACACTTACG
	Reverse	CCACCAGCTTTTGGATCACCA
FTO (qPCR)	Forward	ACTTGGCTCCCTTATCTGACC
	Reverse	TGTGCAGTGTGAGAAAGGCTT
METTL3 (qPCR)	Forward	TTGTCTCCAACCTTCCGTAGT
	Reverse	CCAGATCAGAGAGGTGGTGTAG
METTL14 (qPCR)	Forward	AGTGCCGACAGCATTGGTG
	Reverse	GGAGCAGAGGTATCATAGGAAGC
CYC1 (qPCR)	Forward	CCATCTACACAGAAGTCTTGGAG
	Reverse	GCGTTTTCGATGGTCATGCTCTG
AMY2B (qPCR)	Forward	GATAATGGGAGCAACCAAGTGGC
	Reverse	CAGTATGTGCCAGCAGGAAGAC
ASAH2B (qPCR)	Forward	GAGGCTACTTCAACATCGTGGC
	Reverse	TAGATTCCAGGCTGGGCAGTGT
ADA (qPCR)	Forward	CTGCTGAACGTCATTGGCATGG
	Reverse	GGCGATCCTTTTGATAGCCTCC
ADORA2A (qPCR)	Forward	CGCTCCGGTACAATGGCTT
	Reverse	TTGTTCCAACCTAGCATGGGA
ADORA2B (qPCR)	Forward	TGCACTGACTTCTACGGCTG
	Reverse	GGTCCCCGTGACCAAACTT
CD39 (qPCR)	Forward	AGGTGCCTATGGCTGGATTAC
	Reverse	CCAAAGCTCCAAAGGTTTCCT
CD73 (qPCR)	Forward	GCCTGGGAGCTTACGATTTTG
	Reverse	TAGTGCCCTGGTACTGGTCG
ACACA (qPCR)	Forward	ATGTCTGGCTTGCACCTAGTA
	Reverse	CCCCAAAGCGAGTAACAAATTCT
RAB10 (qPCR)	Forward	CTGCTCCTGATCGGGGATTC
	Reverse	TGATGGTGTGAAATCGCTCCT
PPP2CB (qPCR)	Forward	CTGAACGAGAACCAAGTGCG
	Reverse	ACGAACCTCTTGCACATTTGA
IGF1R (qPCR)	Forward	TCGACATCCGCAACGACTATC
	Reverse	CCAGGGCGTAGTTGTAGAAGAG

PIK3CD (qPCR)	Forward	AAGGAGGAGAATCAGAGCGTT
	Reverse	GAAGAGCGGCTCATACTGGG
GAS6 (qPCR)	Forward	GGTAGCTGAGTTTGACTTCCG
	Reverse	GACAGCATCCCTGTTGACCTT
BCL2L1 (qPCR)	Forward	GAGCTGGTGGTTGACTTTCTC
	Reverse	TCCATCTCCGATTCAGTCCCT
IL3RA (qPCR)	Forward	ACGAAGGAAGATCCAAACCCA
	Reverse	GCATAGAATAGTCGGCGTCTTA
CSF1 (qPCR)	Forward	TGGCGAGCAGGAGTATCAC
	Reverse	AGGTCTCCATCTGACTGTCAAT
FGFR1 (qPCR)	Forward	CCCGTAGCTCCATATTGGACA
	Reverse	TTTGCCATTTTTCAACCAGCG
COL4A2 (qPCR)	Forward	TTATGCACTGCCTAAAGAGGAGC
	Reverse	CCCTTAACTCCGTAGAAACCAAG
COL6A1 (qPCR)	Forward	ACAGTGACGAGGTGGAGATCA
	Reverse	GATAGCGCAGTCGGTGTAGG
COL6A2 (qPCR)	Forward	GACTCCACCGAGATCGACCA
	Reverse	CTTGTAGCACTCTCCGTAGGC
SPP1 (qPCR)	Forward	CTCCATTGACTCGAACGACTC
	Reverse	CAGGTCTGCGAAACTTCTTAGAT
FLNB (qPCR)	Forward	GTGAACAAACGCATCGGCAA
	Reverse	ACCAGACCCAAGATGAGCTTC
ACACB (qPCR)	Forward	CAAGCCGATCACCAAGAGTAAA
	Reverse	CCCTGAGTTATCAGAGGCTGG
ACLY (qPCR)	Forward	TCGGCCAAGGCAATTTCAGAG
	Reverse	CGAGCATACTTGAACCGATTCT
FASN (qPCR)	Forward	AAGGACCTGTCTAGGTTTGATGC
	Reverse	TGGCTTCATAGGTGACTTCCA
GPAM (qPCR)	Forward	GATGTAAGCACACAAGTGAGGA
	Reverse	TCCGACTCATTAGGCTTTCTTTC
MCAT (qPCR)	Forward	CGCGGTCTGCTCAACTACC
	Reverse	CACTAGGGCTGCAAACTCTCC
OLAH (qPCR)	Forward	CCTGGCATCGCATTCCCAA
	Reverse	GGTACGTTAGAGGTGCAACTTC
OXSM (qPCR)	Forward	CAATATCCAGATTGCATAGGCGA
	Reverse	CGATCCCAAACCAGGTGAGTT
SCD (qPCR)	Forward	GCCCCTCTACTTGGAAGACGA
	Reverse	AAGTGATCCCATACAGGGCTC
MLYCD (qPCR)	Forward	ACGTCCGGGAAATGAATGGG
	Reverse	GTAACCCGTTCTAGGTTCAGGA
FABP1 (qPCR)	Forward	GGAGGAATGTGAGCTGGAGACA
	Reverse	TATGTCGCCGTTGAGTTCGGTC
FABP5 (qPCR)	Forward	TGAAGGAGCTAGGAGTGGGAA
	Reverse	TGCACCATCTGTAAAGTTGCAG
CD36 (qPCR)	Forward	GGCTGTGACCGGAACTGTG
	Reverse	AGGTCTCCAACTGGCATTAGAA

SLC27A1 (qPCR)	Forward	GGGGCAGTGTCTCATCTATGG
	Reverse	CCGATGTACTGAACCACCGT
SLC27A2 (qPCR)	Forward	TTTCCGCCATCTACACAGTCC
	Reverse	CGTAGGTGAGAGTCTCGTCG
FADS1 (qPCR)	Forward	CTACCCCGCGCTACTTCAC
	Reverse	CGGTCGATCACTAGCCACC
FADS2 (qPCR)	Forward	TGACCGCAAGGTTTACAACAT
	Reverse	AGGCATCCGTTGCATCTTCTC
ACSL3 (qPCR)	Forward	GCCGAGTGGATGATAGCTGC
	Reverse	ATGGCTGGACCTCCTAGAGTG
ACSL4 (qPCR)	Forward	CATCCCTGGAGCAGATACTCT
	Reverse	TCACTTAGGATTTCCCTGGTCC
ELOVL1 (qPCR)	Forward	TTATTCTCCGAAAGAAAGACGGG
	Reverse	ATGACATGCACGGAAGAGTTTAT
ELOVL3 (qPCR)	Forward	CTGTTCCAGCCCTATAACTTCG
	Reverse	GAATGAGGTTGCCCAATACTCC
ELOVL5 (qPCR)	Forward	TAACAGGAGTATGGGAAGGCA
	Reverse	ACCAGAGGACACGGATAATCTT
ELOVL6 (qPCR)	Forward	AACGAGCAAAGTTTGAACTGAGG
	Reverse	TCGAAGAGCACCGAATATACTGA
ELOVL7 (qPCR)	Forward	GCCTTCAGTGATCTTACATCGAG
	Reverse	AGGACATGAGGAGCCAATCTT
DGAT1 (qPCR)	Forward	TATTGCGGCCAATGTCTTTGC
	Reverse	CACTGGAGTGATAGACTCAACCA
PNPLA2 (qPCR)	Forward	GGCTTCCTCGGCGTCTACTA
	Reverse	TTTACCAGGTTGAAGGAGGGG
CPT1A (qPCR)	Forward	TCCAGTTGGCTTATCGTGGTG
	Reverse	TCCAGAGTCCGATTGATTTTTGC
CPT2 (qPCR)	Forward	CATACAAGCTACATTTCGGGACC
	Reverse	AGCCCGGAGTGTCTTCAGAA
ACACA (RIP-qPCR)	Forward	CGCTGGTTTGTGGAAGTGGA
	Reverse	CTGCTCGCTGAGTGGGTGAT
SCD (RIP-qPCR)	Forward	GCCCCTCTACTTGGAAGACGA
	Reverse	AAGTGATCCCATACAGGGCTC
Ahcy flox/flox (fl/fl)-5'arm	Forward	GTGAAGTCAAGGATTCTCTTCCC
	Reverse	AAGTGCTTGCTTATGGGTGCT
Ahcy flox/flox (fl/fl)-3'arm	Forward	GTAACACTTGCTTCCAGTGCCTG
	Reverse	GAACAAGGAGAGAGGTGATGTGG
pVillin-KI (RT-PCR, Intestine)	Forward	CCTCCTCTCCTGACTACTCCCAGTC
	Reverse	TCACAGAAACCATATGGCGCTCC
pVillin-WT (RT-PCR, Intestine)	Forward	CAGCAAAACCTGGCTGTGGATC
	Reverse	ATGAGCCACCATGTGGGTGTC
