# Supplementary material for: The AHCY–adenosine complex rewires mRNA methylation to enhance fatty acid biosynthesis and tumorigenesis
Source: Cell Res. 2026 Jan 19;36(2):152–72. doi: 10.1038/s41422-025-01213-5 (PMC12848013; doi:10.1038/s41422-025-01213-5)
Supplement: Supplementary file 21 — Video legends [file 41422_2025_1213_MOESM21_ESM.pdf]

Supplementary information, Video S1-3:

Supplementary information, Video S1. Representative movie showing the ADO response of the AHCY-based ADO sensor to ADO (Sensor C1). Representative traces and group analysis of fluorescence changes in Sensor C1-expressing cells in response to 100 μM ADO. Scale bars, 10 μm.

Supplementary information, Video S2. Representative movie showing the response of the transient regulating Sensor C1 to ADO and ABA in an ABA-triggered ABI/PYL1 system. Images of sensor fluorescence before and after the addition of 100 μM ADO followed by 3 mM ABA in HEK293 cells expressing ABI-Sensor C1 and ADA-PYL1. Scale bars, 10 μm.

Supplementary information, Video S3. Representative movie showing the response of the transient regulating GRABAdo.1 to ADO and ABA in an ABA-triggered ABI/PYL1 system. Images of sensor fluorescence before and after the addition of 100 μM ADO followed by 3 mM ABA in HEK293 cells expressing ABI-GRABAdo.1 and ADA-PYL1. Scale bars, 20 μm.
